# Differential miRNA Expressions Linking Environmental Risk Factors to Triple-Negative Breast Cancer Stages at Diagnosis

**DOI:** 10.3390/cancers17162618

**Published:** 2025-08-11

**Authors:** Amjila Bam, Yawen Hu, Xiaocheng Wu, Meng Luo, Nubaira Rizvi, Luis Del Valle, Arnold H. Zea, Fokhrul Hossain, Denise Moore Danos, Jovanny Zabaleta, Augusto Ochoa, Lucio Miele, Edward Trapido, Qingzhao Yu

**Affiliations:** 1 School of Public Health, LSU-Health Sciences Center, New Orleans, LA 70112, USA; abam@lsuhsc.edu (A.B.); xwu@lsuhsc.edu (X.W.); nrizvi@lsuhsc.edu (N.R.); ddanos@lsuhsc.edu (D.M.D.); etrapi@lsuhsc.edu (E.T.); 2 School of Medicine, LSU-Health Sciences Center, New Orleans, LA 70112, USA; yhu2@lsuhsc.edu (Y.H.); mluo2@lsuhsc.edu (M.L.); ldelva@lsuhsc.edu (L.D.V.); azea@lsuhsc.edu (A.H.Z.); fhossa@lsuhsc.edu (F.H.); jzabal@lsuhsc.edu (J.Z.); aochoa@lsuhsc.edu (A.O.); lmiele@lsuhsc.edu (L.M.)

**Keywords:** breast cancer, TNBC, TNBC stage, environmental risk factors, miRNA, TMM normalization, UpSet plot, pathway analysis, functional enrichment analysis

## Abstract

**Background/Objectives:** Triple negative breast cancer (TNBC) is an aggressive, molecularly heterogeneous subtype of breast cancer, accounting for approximately 10–15% of all cases. While reproductive and metabolic factors contribute to breast cancer development, growing concerns about environmental exposures, alongside biological and socio-cultural influences, underscore the need for targeted prevention strategies across diverse populations. Despite increasing evidence linking biological, socioeconomic, and environmental factors to TNBC outcomes, the molecular mechanisms underlying these relationships remain poorly understood. Micro-RNAs (miRNAs), which regulate gene expression and play critical roles in cancer development, have emerged as potential mediators between environmental exposures and TNBC progression. The goal of this research is to identify environmental risk factors that directly relate to TNBC stages and enhance understanding of the mechanisms underlying how miRNAs link environmental exposures to TNBC stages. **Methods:** In this study, we analyzed 434 Formalin-Fixed, Paraffin-Embedded (FFPE) tumor samples from 434 women diagnosed with TNBC between 2009 and 2019, encompassing diverse cancer stages (184 cases from early stage and 250 cases from advanced stage), racial backgrounds, and socioeconomic statuses. The sequencing data were linked with the Louisiana Tumor Registry data and the Environmental Justice index. **Results:** A total of 348 unique miRNAs were identified as differentially expressed across environmental risk factors statistically associated with TNBC stage, adjusting for plate effects. An UpSet plot revealed 44 miRNAs commonly differentially expressed across TNBC stages and multiple environmental exposures. At least one differentially expressed (DE) miRNA was shared between the TNBC stage and each environmental factor, with many associated with receptor-negative and aggressive breast cancer subtypes. **Conclusions:** These findings highlight potential biological pathways through which exposures may drive the TNBC progression and contribute to disparities in outcomes.

## Simple Summary

Triple negative breast cancer (TNBC) is a fast-growing and hard-to-treat form of breast cancer. Researchers are increasingly concerned that environmental conditions, like pollution or poor living environments, may play a role in who develops this disease and how severe it becomes. In this study, we analyzed tumor samples from 434 women with TNBC to explore how environmental factors might influence the stage at which the cancer is diagnosed. We focused on molecules called microRNAs, which can control how genes behave in the body and may help explain how the environment affects cancer. Our findings revealed a group of microRNAs that are linked to both cancer prognosis and environmental exposures. These results help us better understand how external factors might drive cancer progression and could lead to more personalized prevention and treatment efforts in the future, especially for communities more affected by environmental challenges.

## 1. Introduction

Breast cancer is a malignant condition characterized by the uncontrolled growth of abnormal cells within the milk-producing lobules or ducts of the breast, leading to tumor formation. According to the latest update (February 2024) from the National Library of Medicine (NLM), and the key statistics for breast cancer by the American Cancer Society, breast cancer is the most frequently diagnosed cancer among women and 30% of all new cancers each year in the United States, respectively. Globally, it represents over 10% of all new cancer cases each year and is also the second leading cause of cancer-related deaths in women. Triple negative breast cancer (TNBC) is an aggressive, molecularly heterogeneous subtype of breast cancer, known for its rapid growth and high potential to spread. It accounts for approximately 10–15% of all breast cancer cases. TNBC is characterized by immunohistochemically negative staining for estrogen receptor α (ER), progesterone receptors (PR), and a lack of genomic amplification of Erb-B2 receptor tyrosine kinase 2 (ERBB2), leading to overexpression of the human epidermal growth factor receptor 2 (HER2) protein, which limits the effectiveness of targeted therapies. While reproductive factors, estrogen, and progesterone play key roles in ER+ breast cancer development, risk factors for TNBC are less well understood. Type 2 diabetes is among them [PMID: 33415093; PMID: 35725146]. Growing concerns about environmental exposures, alongside biological and socio-cultural influences, continue to drive research and policy efforts, as they likely contribute to unexplained risks and disparities, underscoring the need for targeted prevention strategies across diverse populations [1,2]. 

Among U.S. women diagnosed with invasive breast cancer, early-stage diagnosis rates differ by race and ethnicity. While biological factors partly explain these disparities, growing evidence suggests that physical or built environmental risk factors, as well as their regulation through microRNAs, may also contribute to differences in tumor aggressiveness and delayed diagnosis, particularly in underserved populations [3,4]. TNBC, especially the basal-like subtype, is more commonly observed in African American (AA) women than in non-Hispanic White (NHW), Hispanic, or Asian women [5]. AA women are also more likely to receive a TNBC diagnosis at a younger age and more advanced stage, with a higher risk of early metastasis compared to NHW women [6,7]. Patients with obesity are more likely to be diagnosed with larger primary breast tumors and at a more advanced stage of the disease [8,9]. Specifically, studies, including the 2008 Carolina Breast Cancer Study, suggest a potential link between obesity and higher TNBC incidence in African American women, though findings remain complex due to inconsistent obesity measurements and racial/ethnic variability [10]. Metabolic imbalance, resulting in pre-diabetes and diabetes, may be the link between obesity and TNBC risk [PMID: 35725146]. Despite inconsistent findings, there is some evidence that other biological factors, like increased blood pressure, may be linked to higher rates of breast cancer, more advanced stages of TNBC, and related deaths [11,12,13]. Lactation at a younger age is recognized as a protective biological factor against breast cancer, with early-age breastfeeding associated with a lower risk compared to initiating lactation at an older age [14,15]. Cigarette smoking may contribute to more advanced stages of TNBC by promoting carcinogen accumulation and DNA damage in breast tissue, particularly through mechanisms involving lipophilic transport, DNA adduct formation, and p53 gene mutations, although current evidence remains limited and inconclusive [16]. Socioeconomic factors like insurance, marital status, and neighborhood income affect breast cancer outcomes, with uninsured and unmarried patients more likely to be diagnosed at later stages [17,18,19,20]. Apart from pre-existing biological vulnerability and socioeconomic vulnerability, TNBC can also be linked to various physical environmental burdens. Long-term exposure to PM2.5 air pollution has been linked to larger tumor size at diagnosis, a higher likelihood of developing TNBC, and increased mortality from cancers, especially breast and lung cancer, indicating its role in poorer outcomes and thus suggesting that sustained exposure to particulate matter may influence cancer development and progression by promoting cell growth, invasiveness, and the potential for metastasis [21,22]. Despite growing evidence connecting biological, socioeconomic, and environmental factors to TNBC incidence and outcomes, the underlying molecular mechanisms driving these associations remain poorly understood. 

MicroRNAs, which regulate gene expression and play critical roles in cancer development and progression, have emerged as promising biomarkers that may mediate the effects of environmental exposures on breast cancer outcomes. miRNAs are small, non-coding RNA molecules, typically 22 to 25 nucleotides long, that play a crucial role in regulating gene expression by silencing or degrading target messenger RNAs (mRNAs), thereby influencing key biological processes. Recognized as a distinct class of biological regulators nearly a decade after their discovery in the early 1990s, miRNAs have revolutionized molecular biology, with their dysregulated expression now linked to a wide range of human diseases [23,24,25]. Research has shown that more than 3000 miRNAs are associated with tumor initiation and progression, highlighting their crucial role in cancer biology [26]. Furthermore, in TNBC, numerous miRNAs, including miR-125b, miR-20a, miR-145, and miR-655, have been implicated in key processes driving disease progression, such as epithelial-to-mesenchymal transition (EMT), a central mechanism by which cancer cells acquire migratory and invasive properties. Other research has highlighted the critical role of circulating miRNAs in TNBC, identifying several as major contributors to tumorigenesis and as promising candidates for diagnostic, prognostic, and therapeutic targeting [27]. In addition, numerous miRNAs have been recognized for their tumor-suppressive functions, including the inhibition of cancer cell proliferation, invasion, and migration. According to Xu et al. [26], other tumor-suppressive miRNAs like miR-199a-5p, miR-200b, and miR-96 also play key roles in limiting TNBC progression by inhibiting cell movement and metastatic potential.

However, little is known about how specific miRNAs may link long-term environmental exposures, such as air pollution or metabolic stressors, to the aggressive nature and poor prognosis of TNBC. While previous studies have largely focused on descriptive associations, few have explored potential molecular mediators that bridge environmental injustice and TNBC progression. This study aims to identify environmental risk factors directly associated with TNBC stage at diagnosis and to investigate how miRNAs may mediate the impact of these exposures, thereby improving our understanding of the biological mechanisms linking environment and disease progression. 

For this study, we analyzed 434 FFPE tumor samples from 434 women diagnosed with TNBC between 2009 and 2019 (184 cases from early stage and 250 cases from advanced stage). These samples represented a wide range of cancer stages, tumor aggressiveness, racial and ethnic backgrounds, underlying health conditions, residential environments, and socioeconomic statuses. A key strength of our study lies in the integration of high-resolution Environmental Justice Index (EJI) data with miRNA profiles, enabling us to quantify the influence of structural and environmental disadvantages on tumor biology. The Louisiana Tumor Registry (LTR) facilitated the collection and integration of these samples with data from the SEER cancer registry. Using high-throughput sequencing, we profiled miRNA expressions to identify differentially expressed miRNAs. We also predicted and examined the key genes (e.g., *SRRM2*, *HUWE1*, *KMT2D*) and biological pathways regulated by these DE miRNAs, including the MAPK signaling pathway, cell cycle, Wnt signaling pathway, PI3K-Akt signaling pathway, breast cancer pathway, apoptosis, TGF-beta signaling, p53 signaling, Hedgehog signaling, Notch signaling, and estrogen signaling pathways. Notably, several miRNAs, such as hsa-miR-20b-5p, hsa-miR-7-5p, hsa-miR-1-3p, and 41 others, were found to regulate genes involved in these pathways.

To our knowledge, this is the first study to use miRNA sequencing to investigate environment-gene interactions in the context of TNBC, leveraging a large, population-based sample of 434 FFPE cases enriched with environmental justice metrics. We selected miRNAs due to their molecular stability in FFPE tissue, which makes them ideal for retrospective studies. Moreover, miRNAs are promising candidates for early detection, risk stratification, and therapeutic targeting, reinforcing their value in guiding future TNBC prognosis and precision intervention efforts.

## 2. Methods

### 2.1. Dataset and Measurements

To investigate the relationship between specific miRNAs, outcomes, and environmental risk factors, we analyzed 434 FFPE tissue samples of 134 early-stage and 250 advanced-stage TNBC cases. The samples were collected from 434 patients diagnosed with TNBC between 2009 and 2019. The clinical data were obtained from the LTR, a National Cancer Institute’s SEER (Surveillance, Epidemiology and End Results) and National Program of Central Cancer Registries (NPCR)-affiliated cancer registry that collects comprehensive statewide data on cancer incidence from medical records and linkages with other sources of data. This includes detailed information on morphology, grade, and behavior, as well as anatomical location, stage at diagnosis, treatment modalities, and patient outcomes, such as date of last contact, vital status and cause of death. This study examined one primary outcome variable: TNBC cancer stage, classified as early stage (localized) or advanced stage (regional or distant). Environmental risk factors were the exposure variables sourced from the 2022 Environmental Justice Index (EJI). The EJI serves as the nation’s first place-based metric that quantifies the combined effects of environmental hazards on health outcomes, while integrating measures of social vulnerability, including income, race, ethnicity, national origin, Tribal affiliation, and disability [28]. Drawing from multiple federal and public data sources, including the U.S. Census Bureau, Environmental Protection Agency, Mine Safety and Health Administration, Geological Survey, Department of Transportation, Centers for Disease Control and Prevention, and OpenStreetMap, the EJI provides a comprehensive ranking of environmental injustice and its health-related effects at the census tract level across the United States. The EJI framework encompasses three domains: environmental burden (including metrics related to air pollution, toxic sites, built environment, transportation, and water pollution), social vulnerability (covering racial/ethnic minority status, socioeconomic indicators, household composition, and housing characteristics), and health vulnerability (reflecting the burden of pre-existing chronic conditions) Centers for Disease Control and Prevention and Agency for Toxic Substances Disease Registry [28]. A total of 434 FFPE samples were obtained from major pathology laboratories for tissue collection (using convenience sampling) and subsequent miRNA sequencing. The sequencing data were linked with the SEER cancer registry and environmental data by LTR. All procedures and sample collections were approved by the Institutional Review Board (IRB) at LSU Health-New Orleans.

### 2.2. miRNA Sequencing

After processing 434 FFPE TNBC tissue blocks through standard storage, selection, and cutting procedures, total RNA was extracted using the Quick-RNA FFPE Miniprep Kit (Cat. No. R1008, Zymo Research, Irvine, CA, USA) according to the manufacturer’s protocol. DNase I treatment was performed to eliminate DNA contamination. RNA yield and quality were assessed using a NanoDrop spectrophotometer (Thermo Fisher Scientific, Waltham, MA, USA) and Agilent Bioanalyzer (Agilent Technologies, Santa Clara, CA, USA), and only samples with an RNA integrity number (RIN) ≥ 8 were selected for miRNA library preparation. For miRNA library preparation, 100 ng of high-quality total RNA per sample was used with the QIAseq miRNA Library Kit (Cat. No. 331502, Qiagen, Germantown, MD, USA) and QIAseq miRNA 96 Index Kit (Cat. No. 331565, Qiagen, Germantown, MD, USA), following the manufacturer’s protocol. Library concentrations were measured using the Qubit 4.0 Fluorometer (Thermo Fisher Scientific, Waltham, MA, USA), and libraries were pooled in equimolar ratios. Pooled libraries were quantified by real-time PCR and sequenced on the Illumina (San Diego, CA, USA) NextSeq 2000 Sequencing System (75 bp single read), with PhiX Control v3 added as a 1% internal standard [4]. 

### 2.3. miRNA Normalization

Normalization of miRNA counts is essential for comparing miRNA expression levels across different samples or conditions, to account for variations in sample input, miRNA quality, and ensure that the measured expression levels reflect biological differences rather than technical artifacts. For this study, the Trimmed Mean of *M*-values (TMM) normalization method, proposed by Robinson et al. [29], was used for normalizing the miRNA counts. This approach operates under the assumption that the majority of genes are not differentially expressed and that the overall expression levels across samples are comparable [29,30]. TMM calculates *M*-values, which represent log fold changes, and assigns weights based on the inverse variances, using the delta method applied to binomial distributions. If a reference sample is not specified, in our case, the sample with an upper quartile of counts-per-million closest to the average upper quartile across all samples is automatically chosen. 

### 2.4. Differentially Expressed miRNA Among Environmental Risk Factors

To identify differentially expressed miRNAs associated with environmental risk factors, TMM-normalized miRNA count data were analyzed using the edgeR version 4.6.2 package in R. We examined multiple environmental justice-related variables such as census tract level racial demographics, socioeconomic indicators like poverty and unemployment rates, and individual health metrics including blood pressure, that were found to be significantly associated with the stage at diagnosis of triple-negative breast cancer (except for racial factors, which were not significant in our dataset but were included in the study due to their established relevance in prior research) [2,3,4,6,7,31]. Batch effects (sequencing plate) were adjusted in the model. In parallel, DE analysis was also conducted to identify miRNAs that were differentially expressed between early and advanced stages of TNBC. To control false positives due to multiple comparisons, we applied the Benjamini-Hochberg procedure, and miRNAs were considered significant if they met an FDR-adjusted *p*-value threshold of <0.05. To uncover potential miRNAs at the intersection of environmental influences and disease progression, an UpSet plot was generated in R, using UpSetR version 1.4.0 package, highlighting miRNAs commonly linked to both environmental risk factors and TNBC stage. UpSetR version 1.4.0 visualizes set intersections using a matrix layout, where each row corresponds to a set and each column represents a specific intersection, with the lowest black circle in each column highlighting the column-specific relationships [32,33,34]. This integrative approach helps in revealing miRNA signatures that may mediate the biological impact of environmental disparities on TNBC outcomes.

### 2.5. Pathway Analysis

Pathway Analysis (PA), also referred to as functional enrichment analysis, is a bioinformatics technique used to interpret high-throughput biological data, such as RNA-seq and microarrays, by pinpointing groups of functionally related genes (pathways) that show statistical changes under experimental conditions. The primary aim of PA tools is to examine data from high-throughput technologies, identifying significant clusters of related genes that differ between case samples and a control group. PA methods have been instrumental for researchers in uncovering the biological functions of candidate genes, which are then selected to develop new cancer therapies, minimizing harm to healthy cells [35,36]. 

In our study, after identifying DE miRNAs from TNBC samples, using an FDR-adjusted *p*-value threshold of <0.05, we explored their potential biological impact by retrieving experimentally validated target genes using the multiMiR version 1.30.0 package in R, which integrates data from resources such as miRecords, miRTarBase and TarBase. To understand the functional relevance of these target genes, we conducted gene ontology (GO) enrichment analysis using the clusterProfiler version 4.16.0 and org.Hs.eg.db version 3.21.0 packages to identify significantly enriched biological processes, molecular functions, and cellular components. Additionally, to investigate the involvement of these genes in known signaling and metabolic pathways, we performed KEGG (Kyoto Encyclopedia of Genes and Genomes) pathway enrichment analysis using the *enrichKEGG* function from the clusterProfiler version 4.16.0 package. This comprehensive integrative analysis enabled us to highlight key regulatory pathways and functional networks potentially modulated by DE miRNAs in TNBC, offering insights into their roles in cancer pathogenesis.

### 2.6. Statistical Analysis

To assess the statistical association between TNBC diagnostic stages and various environmental risk factors, we applied either Chi-squared tests or logistic regression, as appropriate. Subsequently, clinical patient data were integrated with normalized miRNA expression data to identify differentially expressed miRNAs, accounting for potential plate or batch effects using the Genewise Negative Binomial Generalized Linear Models with Quasi-Dispersion Estimation (*glmQLFit*). All statistical analyses and data visualizations were conducted using R (version 4.5.0).

## 3. Results

### 3.1. Data Preprocessing 

Preprocessing was necessary to ensure accurate differential expression analysis. Thus, following initial data cleaning, miRNAs with consistently low expression across the 434 FFPE samples were removed to improve the reliability of downstream analyses. This filtering step retained 2283 miRNAs for further study. To ensure comparability across samples and to mitigate technical variation, TMM normalization was applied to the filtered count data. For detailed information on preprocessing steps, refer to Hu et al. [4].

### 3.2. Association Between Environmental Risk Factors and TNBC Stage

Using Chi-squared tests and logistic regression, we assessed the association between TNBC diagnostic stages and a set of environmental risk factors. Several community level factors including indicator of total poverty status (Total_PovStatus), percentage of people who are unemployed (EP_UNEMP), percentage of civilian noninstitutionalized population with a disability estimate and their percentile rank (EP_DISABL and EPL_DISABL), domain consisting of proximity to high volume roads, proximity to railways, and proximity to airports and their percentile ranks (SPL_EBM_THEME4 and RPL_EBM_DOM4), domain consisting of ozone, PM2.5, air toxics cancer risk, and diesel particulate matter and their percentile ranks (SPL_EBM_THEME1 and RPL_EBM_DOM1), domain consisting of percentage of individuals who are a racial/ethnic minority and their percentile ranks (SPL_SVM_DOM1 and RPL_SVM_DOM1), domain consisting of English language proficiency, aged 65 or older, aged 17 or younger, and civilian with a disability and their percentile ranks (SPL_SVM_DOM3 and RPL_SVM_DOM3), percentage of individuals with raw high blood pressures values and their percentile ranks (EP_BPHIGH and EPL_BPHIGH), percentile rank of percentage of individuals with diabetes (EPL_DIABETES), percentile rank of percentage of minority persons (EPL_MINRTY) and FormTot (total concentration of Hazardous Air Pollutant Formaldehyde considered as lifetime cancer risk from inhalation of air toxics by National Air Toxics Assessment) were found to be significantly associated with TNBC stage (Table 1). These variables were subsequently incorporated into our differential expression analysis. Race was the only factor included in this study, despite not showing a significant association with TNBC stages in our dataset, due to its well-established relevance in previous research.

### 3.3. Differential Expression Analysis of miRNAs

In total, 348 unique miRNAs were identified as differentially expressed across environmental risk factors and found to be statistically associated with TNBC stage, while adjusting for plate effects. Additionally, DE analysis was conducted independently to compare miRNA expressions between early and advanced TNBC stages, resulting in 69 differentially expressed miRNAs. The overall result is presented in Supplementary Tables S1 to S19 in the Supplementary Information Section.

### 3.4. Shared miRNAs Across Risk Factors and Disease Stage

**Figure 1:**
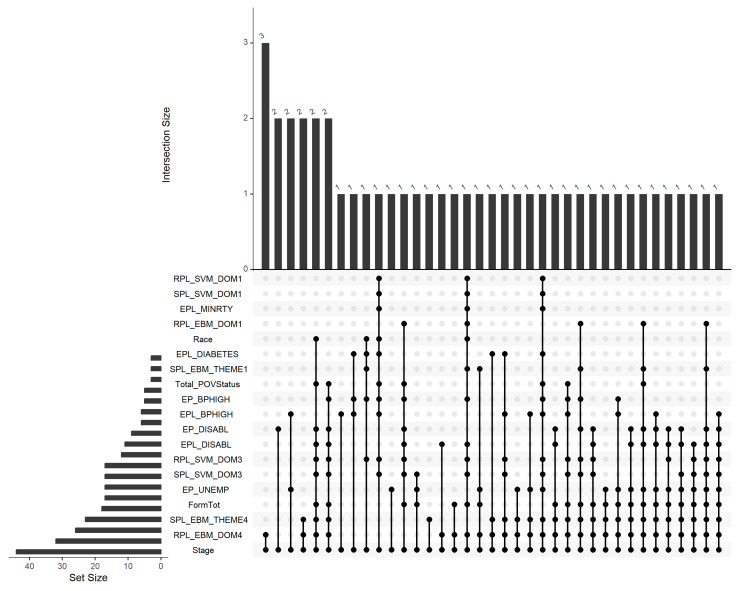
UpSet plot displaying 44 differentially expressed miRNAs shared between TNBC stage and various environmental risk factors. The horizontal bars on the bottom left indicate the total number of DE miRNAs associated with each environmental variable that are also shared with the TNBC stage. The matrix layout below the main bar plot uses dark-filled circles to represent combinations of variables that share one or more DE miRNAs. The vertical bars on the top quantify the number of DE miRNAs common to each specific combination of variables indicated by the corresponding filled circles.

To explore commonalities among DE miRNAs associated with different environmental exposures and TNBC stage, we generated an UpSet plot (Figure 1). The plot illustrates 44 distinct miRNAs that were consistently found to be differentially expressed across different stages of TNBC as well as in association with multiple environmental risk factors. This visualization revealed that the maximum number of miRNAs commonly differentially expressed across RPL_EBM_DOM4 and TNBC stages is 3. There were 2 DE miRNAs shared across TNBC stage and EP_DISABL; 2 DE miRNAs shared across TNBC stage, EP_UNEMP and EPL_BPHIGH; 2 DE miRNAs shared across TNBC stage, RPL_EBM_DOM4 and SPL_EBM_THEME4; 2 DE miRNAs were shared between TNBC stage, RPL_EBM_DOM4, SPL_EBM_THEME4, FormTot, SPL_SVM_DOM3, RPL_SVM_DOM3, EPL_DISABL, EP_DISABL, Total_PovStatus and race; 2 DE miRNAs were shared between TNBC stage, RPL_EBM_DOM4, SPL_EBM_THEME4, FormTot, SPL_SVM_DOM3, RPL_SVM_DOM3, EPL_DISABL, EP_DISABL, EP_BPHIGH and Total_PovStatus. Notably, at least one DE miRNA was shared between the TNBC stage and each individual environmental risk factor, highlighting potential miRNAs of high relevance to both biological progression and environmental disparity in TNBC.

### 3.5. Functional and Pathway Enrichment Analysis

To understand the biological significance of the 44 differentially expressed miRNAs, we conducted functional and pathway enrichment analyses. These analyses identified a total of 12 pathways related to TNBC stage at diagnosis, potentially regulated by the DE miRNAs through their corresponding target genes. Comprehensive details of these enriched pathways, including their adjusted *p*-values and the number of associated target genes, are presented in Table 2. We identified a total of 15,478 target genes (approximately 62% of the transcriptome) that are potentially regulated by the differentially expressed miRNAs. Detailed information on the specific miRNAs linked to each of these genes is provided in Supplementary Table S20 in the Supplementary Material (including only target genes with 10 or more differentially expressed miRNAs), offering insights into the regulatory interactions underlying TNBC-related molecular mechanisms. Out of the 44 differentially expressed miRNAs, all were recognized as mature miRNAs. Among them, hsa-miR-20b-5p showed the broadest influence, targeting 10,424 genes, followed by hsa-miR-7-5p with 7801 gene targets and hsa-miR-1-3p with 6542 targets. Other highly connected miRNAs included hsa-miR-320a-3p (6329 genes), hsa-miR-148a-3p (5910), hsa-miR-335-5p (4946), hsa-miR-138-5p (3928), hsa-miR-574-5p (3766), hsa-miR-335-3p (3602), hsa-miR-671-5p (3290), hsa-miR-500a-3p (3261) and hsa-miR-361-5p (3219). Several additional mature miRNAs were associated with targeting between 1500 and 3000 genes, such as hsa-miR-2110 (2246), hsa-miR-769-5p (2046), hsa-miR-147b-3p (1989), hsa-miR-589-5p (1917), hsa-miR-133a-3p (1633), hsa-miR-629-5p (1616), and hsa-miR-342-5p (1591). Others, such as hsa-miR-320d, hsa-miR-378i, hsa-miR-339-5p, hsa-miR-9-3p, and hsa-miR-455-3p, targeted between 1100 and 1500 genes. The remaining mature miRNAs, including hsa-miR-425-3p, hsa-miR-1275, hsa-miR-361-3p, hsa-miR-1307-3p, hsa-miR-188-5p, hsa-miR-190b-5p, hsa-miR-887-3p, hsa-miR-3615, hsa-miR-29c-5p, and hsa-miR-1307-5p and hsa-miR-874-3p, showed fewer targets, ranging from several hundred to just over a thousand. At the lower end, hsa-miR-4516 targeted 208 genes, hsa-miR-1468-5p 203, hsa-miR-3168 197, hsa-miR-675-3p 125 and hsa-miR-551b-3p 108. The least connected mature miRNAs were hsa-miR-7704, hsa-miR-4497, hsa-miR-4791 and hsa-miR-12136, targeting 62, 44, 39 and 16 genes, respectively.

These 44 shared miRNAs were significantly associated with key aspects of breast cancer progression, including conditions such as progesterone receptor-negative breast cancer, estrogen receptor-negative breast cancer, HER2-negative breast cancer, ductal carcinoma of the breast, and invasive breast carcinoma, which are consistent with the subtype of breast cancer we studied. Gene *SRRM2* was targeted by 31 miRNAs, representing the highest number of miRNA interactions observed, followed by two genes (*HUWE1* and *KMT2D*), which were each targeted by 30 miRNAs. Additionally, a total of 1065 genes were targeted by at least 15 of the 44 differentially expressed miRNAs. KEGG pathway analysis of the 15,478 genes targeted by the 44 differentially expressed miRNAs revealed significant enrichment in several key biological pathways. These include the MAPK signaling pathway (KEGG pathway ID: hsa04010, adjusted *p*-value = 1.195 × 10^−18^), cell cycle (KEGG pathway ID: hsa04110, adjusted *p*-value = 4.5244 × 10^−17^), Wnt signaling pathway (KEGG pathway ID: hsa04310, adjusted *p*-value = 9.300 × 10^−14^), PI3K-Akt signaling pathway (KEGG pathway ID: hsa04151, adjusted *p*-value = 6.328 × 10^−12^), breast cancer (KEGG pathway ID: hsa05224, adjusted *p*-value = 2.839 × 10^−10^), apoptosis (KEGG pathway ID: hsa04210, adjusted *p*-value = 9.172 × 10^−10^), TGF-beta signaling pathway (KEGG pathway ID: hsa04350, adjusted *p*-value = 3.307 × 10^−8^), p53 signaling pathway (KEGG pathway ID: hsa04115, adjusted *p*-value = 5.913 × 10^−8^), hedgehog signaling pathway (KEGG pathway ID: hsa04340, adjusted *p*-value = 1.605 × 10^−6^), notch signaling pathway (KEGG pathway ID: hsa04330, adjusted *p*-value = 2.071 × 10^−6^), apoptosis-multiple species (KEGG pathway ID: hsa04215, adjusted *p*-value = 1.536 × 10^−5^), and estrogen signaling pathway (1.758 × 10^−5^). Among the 15478 genes targeted by the 44 differentially expressed miRNAs, pathway enrichment analysis revealed that the MAPK signaling pathway 278 genes, cell cycle includes 154 targeted genes, Wnt signaling pathway 164 genes, PI3K-Akt signaling pathway 314 genes, breast cancer pathway 137 genes, apoptosis 127 genes, TGF-beta signaling pathway 102 genes, p53 signaling pathway 72 genes, hedgehog signaling pathway 54 genes, notch signaling pathway 59 genes, apoptosis-multiple species 32 genes, and estrogen signaling pathway 120 genes.

## 4. Discussions

In this study, we analyzed miRNA expression profiles from 434 FFPE breast tissue samples collected from patients diagnosed with early or advanced stages of triple-negative breast cancer. The goal was to identify differentially expressed miRNAs associated with various environmental risk factors and TNBC stage at diagnosis. After removing miRNAs that were either entirely zero or missing across all samples and based on the filtering process of a software called Qiagen (details are provided in Hu et al. [4]), a total of 2283 miRNAs were retained for analysis. TMM normalization was applied to the filtered dataset, following the exclusion of low-count miRNAs from the DGE list.

Among the retained miRNAs, several were identified as significantly differentially expressed across environmental variables and TNBC stages:

- 8 miRNAs were associated with census tract-level racial groups (Supplementary Table S1).
- 102 and 196 miRNAs were linked to domains involving proximity to high-volume roads, railways, and airports, as well as their percentile ranks (Supplementary Tables S12 and S2, respectively).
- 40 and 56 miRNAs were associated with the percentage of individuals with high blood pressure values and their percentile ranks (Supplementary Tables S3 and S9, respectively).
- 22 and 19 miRNAs were associated with domains involving ozone, PM2.5, air toxics cancer risk, diesel particulate matter, and their respective percentile ranks (Supplementary Tables S8 and S4).
- 21 miRNAs were linked to the percentage of unemployed individuals (Supplementary Table S5).
- 82 miRNAs were associated with the community-level indicator of total poverty status (Supplementary Table S6).
- 102 and 126 miRNAs were associated with domains comprising English language proficiency, age groups (65+, ≤17), civilian disability status, and their percentile ranks (Supplementary Tables S7 and S10, respectively).
- 69 miRNAs were significantly associated with TNBC stage (Supplementary Table S11).
- 4 miRNAs each were associated with the percentile rank of minority population percentage and domains involving racial/ethnic minority representation and their percentile ranks (Supplementary Tables S13–S15).
- 27 and 24 miRNAs were associated with the percentage and percentile rank of the civilian noninstitutionalized population with disabilities (Supplementary Tables S17 and S16, respectively).
- 12 miRNAs were linked to the percentile rank of individuals with diabetes (Supplementary Table S18).
- 69 miRNAs were associated with the total concentration of hazardous air pollutant formaldehyde, considered as lifetime cancer risk from inhalation of air toxics by the National Air Toxics Assessment (Supplementary Table S19).

In total, 348 unique DE miRNAs were identified across all comparisons, with 44 of them found to be commonly differentially expressed between TNBC stage and one or more environmental risk indicators. These 44 shared miRNAs were significantly associated with critical aspects of breast cancer progression, such as receptor-negative subtypes, including progesterone receptor-negative, estrogen receptor-negative, and HER2-negative breast cancers, as well as ductal carcinoma and invasive breast carcinoma. Among these, hsa-miR-20b-5p and hsa-miR-148a-3p have been previously implicated in TNBC progression and breast cancer prognosis. Specifically, hsa-miR-20b-5p was associated with favorable survival outcomes and exhibited the broadest regulatory influence, targeting over 10,000 genes, suggesting its potential as a biomarker of better prognosis or even a therapeutic target. Likewise, hsa-miR-148a-3p, also linked to improved prognosis, targets over 5900 genes, indicating its likely role in widespread transcriptomic regulation [37]. In addition, hsa-miR-7-5p has been shown to act as a tumor suppressor in breast cancer, including TNBC, where it inhibits cell proliferation and migration by targeting oncogenes such as *EGFR* and *IRS1*. Its established role in promoting apoptosis further supports its relevance as a candidate biomarker and potential therapeutic agent [38,39]. Notably, one gene was targeted by 31 and two genes were each targeted by 30 of these miRNAs, highlighting their potential importance as regulatory hubs. Additionally, 1066 genes were found to be targeted by at least 15 of these miRNAs, indicating strong coordinated regulation across multiple genes involved in cancer-related pathways.

Pathway analysis of the 15,478 genes targeted by the 44 miRNAs revealed their widespread involvement in several essential biological signaling pathways. These pathways play central roles in regulating cell proliferation, programmed cell death, DNA damage response, and immune signaling processes that are often dysregulated in cancer. For instance, the MAPK, PI3K-Akt, and Wnt signaling pathways are known to drive tumor growth, survival, and metastasis and are often activated in TNBC [35,40,41]. The TGF-beta and notch pathways are involved in epithelial-mesenchymal transition and stemness, which can contribute to tumor progression and resistance to therapy in TNBC [42,43]. The p53 signaling pathway, frequently disrupted in TNBC due to high TP53 mutation rates, plays a central role in maintaining genomic stability and inducing apoptosis through effectors like BAX and PUMA, and its dysregulation, often in coordination with PI3K-Akt and EGFR pathways, contributes to unchecked proliferation, genomic instability, and therapy resistance in aggressive TNBC tumors [44,45]. Additionally, the involvement of estrogen-related signaling pathways underscores the hormone-responsive nature of breast tissue. It provides further evidence of how dysregulation in these pathways could contribute to the development of aggressive TNBC subtypes.

Altogether, the enrichment of these pathways suggests that the miRNAs shared between TNBC stage and environmental risk factors likely contribute to TNBC progression by organizing changes in gene networks involved in growth control, apoptosis, and tumor-environment interactions. These findings provide deeper insights into how specific environmental exposures, such as air pollution, proximity to transportation infrastructure, socioeconomic stressors, and community-level health risks, may impact the molecular landscape of TNBC. By identifying differentially expressed miRNAs commonly associated with both environmental risk factors and TNBC stages, this study highlights potential biological pathways through which these exposures may contribute to tumor progression. Such mechanisms may also help explain disparities in breast cancer outcomes across different populations, particularly those disproportionately affected by environmental burdens.

## 5. Conclusions

In this study, we identified key microRNAs that play a significant mediating role between environmental risk factors and the progression of TNBC. Additionally, we uncovered biological pathways that help explain these associations. This study has some limitations. Since all patients in this study are residents of Louisiana, the findings may not be fully generalizable to the broader U.S. population due to regional differences in environmental exposures, demographics, and healthcare access. Louisiana is known for its unique environmental burdens, such as high levels of industrial pollution, and a distinct demographic composition that may influence both EJI distributions and miRNA expression patterns. Additionally, while the Environmental Justice Index (EJI) is a nationally designed metric, its behavior and impact may vary when applied within a single state context. To improve the broader relevance and applicability of our findings, future work will incorporate samples from multiple states across diverse geographic regions. This will help provide a more comprehensive understanding of how environmental and social stressors influence triple-negative breast cancer diagnosis and progression, thereby enhancing the generalizability of our results. Another limitation is that the samples were obtained through convenience sampling from large hospitals and pathology laboratories, which may not accurately reflect patients treated in smaller or rural healthcare settings. This could introduce bias due to differences in patient demographics, tumor subtypes, and clinical management across facilities. In particular, miRNA expression profiles may be influenced by tumor subtype enrichment or treatment availability, which could affect the generalizability of our molecular findings. While this limitation is partly mitigated by our focus on environmental risk factors across a diverse patient population, future studies should incorporate more systematic and geographically diverse sampling strategies to validate these results. The third limitation of this study is the temporal mismatch between the EJI data collection and the time of TNBC stage diagnoses. Since EJI scores are based on environmental and demographic data aggregated for 2022, while TNBC diagnoses in this study span from 2009 to 2019, there may be discrepancies between the environmental conditions at the time of diagnosis and those captured by the EJI. This temporal gap could influence the accuracy of associations observed between environmental exposures and cancer outcomes. Most EJI indicators are based on data collected within the last five years, and while precise quantification of historical variability is limited due to data constraints, the overall ranking of census tracts tends to be consistent for these structural factors. Additionally, many indicators include estimation uncertainty, such as margins of error from the U.S. Census, which are not incorporated into EJI scores [28]. Therefore, small differences in EJI rankings should be interpreted with caution. To improve temporal precision and reduce exposure misclassification, future studies should incorporate time-specific environmental data when available. As part of future research, we plan to collect longitudinal environmental risk data and align the timing of these exposures with the timing of TNBC diagnoses.

Our study concentrated solely on TNBC stage at diagnosis as the primary outcome variable, because stage is a strong predictor of progression and mortality. However, future research could consider additional cancer-related outcomes such as tumor aggressiveness (e.g., tumor grade, tumor size, lymphovascular invasion, intratumoral necrosis, and basal-like subtypes), survival, and other relevant indicators. Furthermore, to better understand how miRNAs mediate the relationship between environmental risk factors and TNBC stage, we plan to expand our work by incorporating mediation analysis.

In conclusion, this study uncovered stage-specific miRNA expression patterns in TNBC tumors across diverse racial groups, environmental exposures, and clinical profiles, revealing key regulatory genes and pathways potentially influenced by these miRNAs. The results contribute to a deeper understanding of TNBC biology and may inform future efforts in improving early detection, guiding intervention strategies, and enhancing patient-specific care plans.

## Figures and Tables

**Table 1:** Association between environmental risk factors and TNBC stages.

	Early Stage (*n* = 184) Counts (Row Percentage, %)	Advanced Stage (*n* = 250) Counts (Row Percentage, %)	*p*-Value *
**Race**			0.111
African American	104 (46.0)	122 (54.0)	
Caucasian American	80 (38.5)	128 (61.5)	
**EPL_MINRTY**			0.048
EPL_MINRTY_1 (≤0.54)	80 (48.5)	85 (51.5)	
EPL_MINRTY_2 (>0.54)	104 (38.8)	164 (61.2)	
**SPL_SVM_DOM1**			0.048
SPL_SVM_DOM1_1 (≤0.54)	80 (48.5)	85 (51.5)	
SPL_SVM_DOM1_2 (>0.54)	104 (38.8)	164 (61.2)	
**RPL_SVM_DOM1**			0.048
RPL_SVM_DOM1_1 (≤0.54)	80 (48.5)	85 (51.5)	
RPL_SVM_DOM1_2 (>0.54)	104 (38.8)	164 (61.2)	
**Total_PovStatus**			0.047
Total_PovStatus_1 (<2350)	33 (52.4)	30 (47.6)	
Total_PovStatus_2 (≥2350, <6250)	91 (36.8)	156 (63.2)	
Total_PovStatus_3 (≥6250)	33 (45.8)	39 (54.2)	
Total_PovStatus_4 (missing)	27 (51.9)	25 (48.1)	
**EPL_DIABETES**			0.033
EPL_DIABETES_1 (≤0.44)	43 (53.1)	38 (46.9)	
EPL_DIABETES_2 (>0.44)	141 (40.1)	211 (58.9)	
**RPL_EBM_DOM1**			0.025
RPL_EBM_DOM1_1 (<0.24)	18 (62.1)	11 (37.9)	
RPL_EBM_DOM1_2 (≥0.24, <0.52)	62 (36.5)	108 (63.5)	
RPL_EBM_DOM1_3 (≥0.52)	104 (44.4)	130 (55.6)	
**EP_BPHIGH**			0.031
EP_BPHIGH_1 (<35)	48 (53.3)	42 (46.7)	
EP_BPHIGH_2 (≥35, <39)	34 (37.0)	58 (63.0)	
EP_BPHIGH_3 (≥39, <44)	56 (46.3)	65 (53.7)	
EP_BPHIGH_4 (≥44)	46 (35.4)	84 (64.6)	
**SPL_EBM_THEME1**			0.022
SPL_EBM_THEME1_1 (<1.26)	19 (57.6)	14 (42.4)	
SPL_EBM_THEME1_2 (≥1.26, <1.9)	56 (35.0)	104 (65.0)	
SPL_EBM_THEME1_3 (≥1.9)	109 (45.4)	131 (54.6)	
**SPL_SVM_DOM3**			0.019
SPL_SVM_DOM3_1 (<1.75)	53 (51.5)	50 (48.5)	
SPL_SVM_DOM3_2 (≥1.75, <2)	38 (38.0)	62 (62.0)	
SPL_SVM_DOM3_3 (≥2, <2.25)	49 (49.0)	51 (51.0)	
SPL_SVM_DOM3_4 (≥2.25)	44 (33.8)	86 (66.2)	
**EP_UNEMP**			0.018
EP_UNEMP_1 (≤7)	93 (37.5)	155 (62.5)	
EP_UNEMP_2 (>7)	90 (48.9)	94 (51.1)	
**FormTot**			0.009
FormTot_1 (≤2500)	123 (45.4)	148 (54.6)	
FormTot_2 (>2500)	34 (30.6)	77 (69.4)	
FormTot_3 (missing)	27 (51.9)	25 (48.1)	
**SPL_EBM_THEME4**			0.006
SPL_EBM_THEME4_1 (<1)	83 (48.0)	90 (52.0)	
SPL_EBM_THEME4_2 (≥1, <1.6)	77 (44.3)	97 (55.7)	
SPL_EBM_THEME4_3 (≥1.6)	24 (27.9)	62 (72.1)	
**RPL_EBM_DOM4**			0.006
RPL_EBM_DOM4_1 (<0.48)	82 (47.7)	90 (52.3)	
RPL_EBM_DOM4_2 (≥0.48, <0.63)	22 (38.6)	35 (61.4)	
RPL_EBM_DOM4_3 (≥0.63, <0.81)	56 (48.3)	60 (51.7)	
RPL_EBM_DOM4_4 (≥0.81)	24 (27.3)	64 (72.7)	
**EPL_DISABL**			0.005
EPL_DISABL_1 (≤0.54)	74 (52.1)	68 (47.9)	
EPL_DISABL_2 (>0.54)	110 (37.8)	181 (62.2)	
**EP_DISABL**			0.004
EP_DISABL_1 (≤13)	71 (53.0)	64 (47.4)	
EP_DISABL_2 (>13)	113 (38.0)	185 (62.1)	
**RPL_SVM_DOM3**			0.004
RPL_SVM_DOM3_1 (<0.35)	65 (50.0)	65 (50.0)	
RPL_SVM_DOM3_2 (≥0.35, <0.51)	29 (36.3)	51 (63.8)	
RPL_SVM_DOM3_3 (≥0.51, <0.74)	53 (50.5)	52 (49.5)	
RPL_SVM_DOM3_4 (≥0.74)	37 (31.4)	81 (68.6)	
**EPL_BPHIGH**			0.003
EPL_BPHIGH_1 (≤0.65)	46 (57.5)	34 (42.5)	
EPL_BPHIGH_2 (>0.65)	138 (39.1)	215 (60.9)	

* Represents the *p*-value obtained from a Likelihood Ratio Test (LRT), conducted by comparing a full model including the variable of interest (each environmental risk factor) with a nested model excluding it, to assess their significance in relation to TNBC stage at diagnosis.

**Table 2:** KEGG pathway enrichment of 15,478 genes.

KEGG Pathway ID	Description Pathway	*p*-Adjust *	Gene Counts
has04010	MAPK signaling	1.195 × 10^−18^	278
has04110	Cell cycle	4.524 × 10^−17^	154
has04310	Wnt signaling	9.300 × 10^−14^	164
has04151	PI3K-Akt signaling	6.328 × 10^−12^	314
has05224	Breast cancer	2.839 × 10^−10^	137
has04210	Apoptosis	9.172 × 10^−10^	127
has04350	TGF-beta signaling	3.307 × 10^−8^	102
has04115	p53 signaling	5.913 × 10^−8^	72
has04340	Hedgehog signaling	1.605 × 10^−6^	54
has04330	Notch signaling pathway	2.071 × 10^−6^	59
has04215	Apoptosis—multiple species	1.536 × 10^−5^	32
has04915	Estrogen signaling pathway	1.758 × 10^−5^	120

* Represents the *p*-value obtained after adjustment using the Benjamini-Hochberg (BH) method, which controls the false discovery rate (FDR) to account for multiple testing and reduce the likelihood of false positives.

## Data Availability

The data that supports the findings of this study is restrictedly accessible by request from the Louisiana Tumor Registry and will be released without IRB approval. For inquiries regarding the data, researchers are encouraged to reach out to Lauren S. Maniscalco at lspiza@lsuhsc.edu for further details on the LTR’s data release policies.

## Supplementary Materials

The following supporting information can be downloaded at: https://www.mdpi.com/article/10.3390/cancers17162618/s1, Supplementary Table S1: Differentially expressed miRNAs among racial groups; Supplementary Table S2: Differentially expressed miRNAs among RPL_EBM_DOM4 groups; Supplementary Table S3: Differentially expressed miRNAs among EP_BPHIGH groups; Supplementary Table S4: Differentially expressed miRNAs among RPL_EBM_DOM1 groups; Supplementary Table S5: Differentially expressed miRNAs among EP_UNEMP groups; Supplementary Table S6: Differentially expressed miRNAs among Total_PovStatus groups; Supplementary Table S7: Differentially expressed miRNAs among SPL_SVM_DOM3 groups; Supplementary Table S8: Differentially expressed miRNAs among SPL_EBM_THEME1 groups; Supplementary Table S9: Differentially expressed miRNAs among EPL_BPHIGH groups; Supplementary Table S10: Differentially expressed miRNAs among RPL_SVM_DOM3 groups; Supplementary Table S11: Differentially expressed miRNAs among TNBC stages; Supplementary Table S12: Differentially expressed miRNAs among SPL_EBM_THEME4 groups; Supplementary Table S13: Differentially expressed miRNAs among EPL_MINRTY groups; Supplementary Table S14: Differentially expressed miRNAs among SPL_SVM_DOM1 groups; Supplementary Table S15: Differentially expressed miRNAs among RPL_SVM_DOM1 groups; Supplementary Table S16: Differentially expressed miRNAs among EPL_DISABL groups; Supplementary Table S17: Differentially expressed miRNAs among EP_DISABL groups; Supplementary Table S18: Differentially expressed miRNAs among EPL_DIABETES groups; Supplementary Table S19: Differentially expressed miRNAs among FormTot groups; Supplementary Table S20: Differentially expressed miRNAs linked to each of the target genes (including only target genes with 15 or more differentially expressed miRNAs).

## Abbreviations

The following abbreviations are used in this manuscript:

TNBC	Triple Negative Breast Cancer
miRNA	Micro Ribonucleic Acid
LTR	Louisiana Tumor Registry
FFPE	Formalin-Fixed, Paraffin-Embedded
DE	Differentially Expressed
NLM	National Library of Medicine
ER	Estrogen Receptor
PR	Progesterone Receptor
HER2	Human Epidermal Growth Factor Receptor 2
ERBB2	Erb-B2 Receptor Tyrosine Kinase 2
TMM	Trimmed Mean of M-values
KEGG	Kyoto Encyclopedia of Genes and Genomes
GO	Gene Ontology
PA	Pathway Analysis
FDR	False Discovery Rate
BH	Benjamini-Hochberg
RIN	RNA Integrity Number
