# Supplementary material for: Differential miRNA Expressions Linking Environmental Risk Factors to Triple-Negative Breast Cancer Stages at Diagnosis
Source: Cancers (Basel). 2025 Aug 11;17(16):2618. doi: 10.3390/cancers17162618 (PMC12385157; doi:10.3390/cancers17162618)
Supplement: Supplementary file 1 [file cancers-17-02618-s001.zip › cancers-3755658-supplementary.pdf]

Supplementary Information

Supplementary Table S1. Differentially expressed miRNAs among racial groups

miRNA	logFC	FDR p-value
hsa-miR-483-3p	-0.852	0.000
hsa-miR-675-3p	-0.688	0.005
hsa-miR-335-3p	-0.444	0.007
hsa-miR-9-3p	-0.838	0.010
hsa-miR-100-5p	-0.366	0.012
hsa-miR-146b-5p	-0.255	0.016
hsa-miR-320d	-0.635	0.016
hsa-miR-320c	-0.609	0.021

Supplementary Table S2. Differentially expressed miRNAs among RPL_EBM_DOM4 groups

miRNA	logFC	FDR p-value	miRNA	logFC	FDR p-value
hsa-miR-517a-3p	1.226	0	hsa-miR-425-5p	-0.373	0.049
hsa-miR-122-5p	-1.975	0.001	hsa-miR-489-3p	0.703	0.049
hsa-miR-21-3p	0.83	0.001	hsa-miR-31681	3.426	0
hsa-miR-187-3p	1.162	0.001	hsa-miR-517a-3p2	-1.714	0
hsa-miR-449a	1.294	0.001	hsa-miR-320c1	1.872	0
hsa-miR-30d-3p	0.614	0.004	hsa-miR-320d1	1.827	0
hsa-miR-190b-5p	0.858	0.008	hsa-miR-44881	3.064	0
hsa-miR-519c-5p	1.064	0.016	hsa-miR-45161	2.315	0
hsa-miR-675-3p	-0.894	0.036	hsa-miR-370-3p1	1.937	0
hsa-miR-9983-3p	1.139	0.039	hsa-miR-45081	2.5	0
hsa-miR-133a-3p	4.151	0	hsa-miR-127-3p2	1.066	0
hsa-miR-1-3p	3.496	0	hsa-miR-382-5p1	1.019	0
hsa-miR-206	4.489	0	hsa-miR-409-3p2	1.016	0.001
hsa-miR-499a-5p	1.104	0	hsa-miR-44921	2.026	0.001
hsa-miR-95-3p	0.505	0	hsa-miR-39601	2.133	0.001
hsa-miR-378i	0.621	0.002	hsa-miR-671-5p1	1.221	0.001
hsa-miR-138-5p	-0.84	0.015	hsa-miR-769-5p2	0.854	0.002
hsa-miR-654-3p	0.409	0.03	hsa-miR-1307-3p1	1.044	0.003
hsa-miR-376b-5p	0.419	0.048	hsa-miR-337-3p1	0.798	0.003
hsa-miR-378a-5p	0.382	0.048	hsa-miR-1307-5p1	1.021	0.004
hsa-miR-323a-3p	0.447	0.048	hsa-miR-77041	1.169	0.008
hsa-miR-1307-3p	-0.588	0.048	hsa-miR-320a-3p	0.967	0.008
hsa-miR-361-3p	-0.273	0.048	hsa-miR-574-5p	0.55	0.01
hsa-miR-653-3p	0.504	0.048	hsa-miR-21-3p2	-0.694	0.012
hsa-miR-20b-5p	0.471	0.048	hsa-miR-1275	1.019	0.013
hsa-miR-769-5p	-0.459	0.048	hsa-miR-320b	0.841	0.013
hsa-miR-3168	2.496	0	hsa-miR-338-3p	-0.51	0.013
hsa-miR-4488	3.177	0	hsa-miR-874-3p	0.899	0.015
hsa-miR-196a-3p	1.336	0	hsa-miR-196a-3p1	0.927	0.017
hsa-miR-320d	1.35	0	hsa-miR-127-5p	0.583	0.022

hsa-miR-4516	1.928	0	hsa-miR-2062	2.334	0.028
hsa-miR-582-3p	1.067	0	hsa-miR-425-3p1	0.467	0.031
hsa-miR-320c	1.228	0	hsa-miR-363-3p	-0.641	0.037
hsa-miR-370-3p	1.582	0	hsa-miR-12901	0.831	0.041
hsa-miR-122-5p1	-2.03	0	hsa-miR-519a-3p	-1.216	0.047
hsa-miR-4492	2.101	0	hsa-miR-519c-5p2	-0.99	0.049
hsa-miR-4508	2.067	0	hsa-miR-31682	2.915	0
hsa-miR-3960	1.78	0	hsa-miR-320d2	1.892	0
hsa-miR-33a-5p	0.743	0.001	hsa-miR-320c2	1.836	0
hsa-miR-127-3p	0.664	0.002	hsa-miR-133a-3p2	-3.683	0
hsa-miR-409-3p	0.721	0.002	hsa-miR-44882	3.452	0
hsa-miR-184	1.069	0.002	hsa-miR-1-3p2	-3.23	0
hsa-miR-4662a-5p	0.767	0.002	hsa-miR-2063	-4.225	0
hsa-miR-671-5p	0.848	0.002	hsa-miR-122-5p3	-2.491	0
hsa-miR-382-5p	0.647	0.003	hsa-miR-671-5p2	1.454	0
hsa-miR-187-3p1	0.834	0.005	hsa-miR-44922	2.381	0
hsa-miR-584-5p	0.541	0.009	hsa-miR-769-5p3	0.981	0
hsa-miR-483-3p	-0.785	0.009	hsa-miR-1307-5p2	1.171	0
hsa-miR-1246	0.75	0.009	hsa-miR-45082	2.167	0
hsa-miR-30d-3p1	0.472	0.009	hsa-miR-45162	1.784	0
hsa-miR-3196	1.215	0.01	hsa-miR-196a-3p2	1.109	0
hsa-miR-425-3p	0.391	0.013	hsa-miR-1307-3p2	1.031	0
hsa-miR-222-3p	-0.318	0.013	hsa-miR-425-3p2	0.585	0
hsa-miR-769-5p1	0.522	0.014	hsa-miR-582-3p1	0.938	0
hsa-miR-219a-5p	0.6	0.016	hsa-miR-39602	1.936	0
hsa-miR-151a-3p	0.277	0.017	hsa-miR-320b1	0.918	0
hsa-miR-33b-5p	0.713	0.018	hsa-miR-339-5p	0.545	0
hsa-miR-542-3p	0.488	0.025	hsa-miR-33a-5p1	0.855	0
hsa-miR-1290	0.648	0.027	hsa-miR-151a-3p1	0.397	0
hsa-miR-4791	0.612	0.027	hsa-miR-77042	1.158	0
hsa-miR-196a-5p	0.529	0.027	hsa-miR-483-3p2	-0.978	0.001
hsa-miR-1307-5p	0.614	0.028	hsa-miR-370-3p2	1.293	0.001
hsa-miR-145-5p	-0.477	0.028	hsa-miR-12751	1.028	0.001
hsa-let-7b-5p	-0.355	0.029	hsa-miR-574-5p1	0.532	0.001
hsa-miR-449a1	0.741	0.039	hsa-miR-449a3	1.115	0.001
hsa-miR-148b-5p	0.349	0.039	hsa-miR-499a-5p2	-0.861	0.001
hsa-let-7c-5p	-0.466	0.042	hsa-miR-4662a-5p1	0.824	0.001
hsa-miR-7704	0.723	0.044	hsa-miR-191-3p	0.706	0.001
hsa-miR-190b-5p1	0.534	0.047	hsa-miR-320a-3p1	0.846	0.001
hsa-miR-378d	0.438	0.047	hsa-miR-187-3p3	0.965	0.002
hsa-miR-2061	6.559	0	hsa-miR-47911	0.806	0.002
hsa-miR-133a-3p1	4.386	0	hsa-miR-129-5p	1.126	0.003
hsa-miR-1-3p1	4.014	0	hsa-miR-874-3p1	0.786	0.005
hsa-miR-122-5p2	2.436	0	hsa-miR-132-3p	-0.412	0.006
hsa-miR-449a2	-1.667	0	hsa-miR-20b-5p1	-0.631	0.006
hsa-miR-517a-3p1	-1.18	0	hsa-miR-33b-5p1	0.805	0.007
hsa-miR-187-3p2	-1.292	0	hsa-miR-31961	1.262	0.007
hsa-miR-21-3p1	-0.814	0.001	hsa-miR-425-5p1	0.373	0.01

hsa-miR-519c-5p1	-1.25	0.005	hsa-miR-589-5p	0.477	0.011
hsa-miR-337-3p	0.753	0.005	hsa-miR-95-3p1	-0.431	0.011
hsa-miR-487b-3p	0.563	0.012	hsa-miR-12902	0.739	0.012
hsa-miR-499a-5p1	0.889	0.012	hsa-miR-3615	0.79	0.013
hsa-miR-147b-3p	-1.002	0.016	hsa-miR-219a-5p1	0.623	0.013
hsa-miR-9983-3p1	-1.275	0.028	hsa-miR-500a-3p	0.377	0.013
hsa-miR-337-5p	0.502	0.028	hsa-miR-335-5p	-0.46	0.016
hsa-miR-329-3p	0.675	0.028	hsa-miR-653-3p1	-0.582	0.024
hsa-miR-483-3p1	0.841	0.036	hsa-miR-10b-5p	-0.368	0.028
hsa-miR-127-3p1	0.651	0.037	hsa-miR-4443	0.917	0.029
hsa-miR-376b-5p1	0.559	0.038	hsa-miR-2110	0.695	0.029
hsa-miR-12136	-0.395	0.039	hsa-miR-2355-3p	0.541	0.042
hsa-miR-30d-3p2	-0.497	0.043	hsa-miR-378i1	-0.497	0.044
hsa-miR-154-5p	0.453	0.043	hsa-miR-769-3p	0.599	0.045
hsa-miR-190b-5p2	-0.704	0.047	hsa-miR-887-3p	0.562	0.045
hsa-miR-409-3p1	0.672	0.047	hsa-miR-1261	-0.504	0.048
hsa-miR-654-3p1	0.49	0.047	hsa-miR-330-5p	0.506	0.048
hsa-miR-375-3p	-0.83	0.047	hsa-miR-130a-3p	-0.353	0.048
hsa-miR-432-5p	0.596	0.048	hsa-miR-615-3p	0.494	0.048
hsa-miR-323a-3p1	0.57	0.048	hsa-miR-12461	0.636	0.049

Supplementary Table S3. Differentially expressed miRNAs among EP_BPHIGH groups

miRNA	logFC	FDR p-value	miRNA	logFC	FDR p-value
hsa-miR-206	-5.299	0	hsa-miR-93-5p	0.42	0.009
hsa-miR-9-3p	1.692	0	hsa-miR-517a-3p1	-0.905	0.009
hsa-miR-370-3p	-1.485	0.001	hsa-miR-9-3p1	1.202	0.009
hsa-miR-517a-3p	-1.095	0.001	hsa-miR-1275	-0.9	0.016
hsa-miR-1-3p	-2.177	0.001	hsa-miR-4791	0.788	0.016
hsa-miR-127-3p	-0.757	0.006	hsa-miR-455-3p	0.539	0.018
hsa-miR-133a-3p	-1.981	0.006	hsa-miR-25-3p	0.322	0.023
hsa-miR-409-3p	-0.823	0.006	hsa-miR-181b-5p	0.329	0.024
hsa-miR-122-5p	-1.613	0.019	hsa-miR-675-3p	0.783	0.035
hsa-miR-92b-3p	0.426	0.024	hsa-miR-516a-5p	-1.254	0.035
hsa-miR-9-5p	1.182	0.026	hsa-miR-551b-3p	-0.719	0.004
hsa-miR-378a-3p	-0.57	0.026	hsa-miR-93-5p1	0.404	0.035
hsa-miR-193b-5p	-0.912	0.027	hsa-miR-2061	5.229	0
hsa-miR-1468-5p	-0.921	0.03	hsa-miR-1-3p1	1.624	0.019
hsa-miR-1283	-1.217	0.03	hsa-miR-190b-5p1	0.786	0.019
hsa-miR-335-3p	-0.552	0.03	hsa-miR-2062	5.976	0
hsa-miR-382-5p	-0.648	0.032	hsa-miR-133a-3p1	2.807	0
hsa-miR-190b-5p	1.013	0	hsa-miR-1-3p2	2.33	0
hsa-miR-12831	-1.61	0.003	hsa-miR-3168	1.452	0.014
hsa-miR-122-5p1	-1.859	0.005	hsa-miR-370-3p1	1.14	0.028

Supplementary Table S4. Differentially expressed miRNAs among RPL_EBM_DOM1 groups

miRNA	logFC	FDR p-value
hsa-miR-517a-3p	-1.918	0
hsa-miR-515-5p	-2.106	0
hsa-miR-526b-5p	-1.843	0
hsa-miR-675-3p	1.683	0.002
hsa-miR-509-3p	-1.5	0.003
hsa-miR-675-5p	1.972	0.008
hsa-miR-1307-5p	1.207	0.036
hsa-miR-671-5p	1.297	0.04
hsa-miR-515-5p1	-2.099	0
hsa-miR-517a-3p1	-1.768	0
hsa-miR-526b-5p1	-1.95	0
hsa-miR-509-3p1	-1.996	0
hsa-miR-122-5p	3.246	0
hsa-miR-217-5p	-1.606	0
hsa-miR-122-5p1	1.718	0
hsa-miR-3168	-1.604	0
hsa-miR-675-5p1	-0.926	0.001
hsa-miR-1468-5p	0.702	0.026
hsa-miR-196b-5p	-0.344	0.03

Supplementary Table S5. Differentially expressed miRNAs among EP_UNEMP groups

miRNA	logFC	FDR p-value
hsa-miR-769-5p	-0.722	0
hsa-miR-671-5p	-0.84	0.001
hsa-miR-1307-5p	-0.712	0.002
hsa-miR-1307-3p	-0.742	0.002
hsa-miR-190b-5p	0.583	0.003
hsa-miR-339-5p	-0.354	0.004
hsa-miR-425-3p	-0.34	0.007
hsa-miR-574-5p	-0.336	0.009
hsa-miR-1275	-0.677	0.011
hsa-miR-2110	-0.581	0.015
hsa-miR-4497	-0.653	0.016
hsa-miR-589-5p	-0.342	0.021
hsa-miR-629-5p	-0.236	0.021
hsa-miR-330-5p	-0.433	0.021
hsa-miR-874-3p	-0.547	0.037
hsa-miR-320a-3p	-0.53	0.042
hsa-miR-3615	-0.543	0.043
hsa-miR-29c-5p	-0.286	0.046
hsa-miR-769-3p	-0.464	0.046
hsa-miR-342-5p	-0.319	0.046
hsa-miR-378d	-0.35	0.046

Supplementary Table S6. Differentially expressed miRNAs among Total_PovStatus groups

miRNA	logFC	FDR p-value	miRNA	logFC	FDR p-value
hsa-miR-133a-3p	-2.273	0	hsa-miR-379-5p	-0.393	0.045
hsa-miR-206	-4.787	0	hsa-miR-590-3p	0.544	0.045
hsa-miR-1-3p	-2.06	0	hsa-miR-455-5p	0.533	0.046
hsa-miR-376a-3p	-0.493	0.002	hsa-miR-1246	0.702	0.046
hsa-miR-137-3p	-1.136	0.002	hsa-miR-133a-3p1	-0.116	0
hsa-miR-1468-5p	1.135	0.003	hsa-miR-137-3p1	-1.501	0.008
hsa-miR-337-5p	-0.51	0.003	hsa-miR-203a-3p	1.434	0.023
hsa-miR-4662a-5p	0.884	0.003	hsa-miR-449a	1.329	0.037
hsa-miR-487b-3p	-0.524	0.003	hsa-miR-133a-3p2	-2.197	0
hsa-miR-1283	1.617	0.003	hsa-miR-122-5p	2.812	0
hsa-miR-329-3p	-0.666	0.004	hsa-miR-133a-3p3	2.157	0
hsa-miR-551a	1.157	0.004	hsa-miR-2061	4.704	0
hsa-miR-381-3p	-0.514	0.004	hsa-miR-499a-5p	0.955	0.001
hsa-miR-9-3p	1.284	0.004	hsa-miR-1-3p1	2.333	0.001
hsa-miR-516a-5p	1.577	0.007	hsa-miR-122-5p1	-1.68	0.002
hsa-miR-582-3p	0.858	0.007	hsa-miR-203a-3p1	1.192	0.002
hsa-miR-154-5p	-0.444	0.01	hsa-miR-455-5p1	-0.751	0.003
hsa-miR-542-5p	-0.49	0.011	hsa-miR-181b-5p	-0.379	0.003
hsa-miR-299-3p	-0.503	0.011	hsa-miR-455-3p	-0.578	0.005
hsa-miR-519a-3p	1.291	0.011	hsa-miR-449a1	1.03	0.007
hsa-miR-4488	1.739	0.011	hsa-miR-320c	-0.944	0.012
hsa-miR-337-3p	-0.563	0.011	hsa-miR-9-3p1	-1.122	0.013
hsa-miR-299-5p	-0.505	0.012	hsa-miR-320d	-0.918	0.018
hsa-miR-4791	0.764	0.016	hsa-miR-9-5p1	-1.048	0.036
hsa-miR-665	-0.634	0.016	hsa-miR-44881	-1.505	0.045
hsa-miR-654-3p	-0.456	0.016	hsa-miR-5771	-0.856	0.049
hsa-miR-376b-5p	-0.491	0.017	hsa-miR-181d-5p	-0.436	0.049
hsa-miR-494-3p	-0.479	0.018	hsa-miR-133a-3p4	0.076	0
hsa-miR-934	1.189	0.019	hsa-miR-122-5p2	1.999	0
hsa-miR-146a-3p	0.884	0.021	hsa-miR-519a-3p1	-2.028	0
hsa-miR-1307-5p	0.742	0.021	hsa-miR-12831	-2.11	0
hsa-miR-519c-5p	0.965	0.025	hsa-miR-516a-5p1	-2.145	0.001
hsa-miR-411-5p	-0.432	0.027	hsa-miR-1468-5p1	-1.228	0.003
hsa-miR-577	0.903	0.027	hsa-miR-203b-3p	-1.3	0.006
hsa-miR-369-5p	-0.407	0.029	hsa-miR-1269a	1.638	0.014
hsa-miR-199b-5p	-0.322	0.032	hsa-miR-519c-5p1	-1.192	0.028
hsa-miR-1185-1-3p	-0.498	0.032	hsa-miR-33b-5p	-0.943	0.038
hsa-miR-376b-3p	-0.49	0.033	hsa-miR-44882	-1.86	0.038
hsa-miR-33a-5p	0.615	0.033	hsa-miR-133a-3p5	-2.081	0
hsa-miR-190b-5p	0.631	0.036	hsa-miR-122-5p3	3.679	0
hsa-miR-9-5p	0.985	0.039	hsa-miR-203a-3p2	-1.917	0

Supplementary Table S7. Differentially expressed miRNAs among SPL_SVM_DOM3 groups

miRNA	logFC	FDR p-value	miRNA	logFC	FDR p-value
hsa-miR-206	-5.466	0	hsa-miR-44881	2.944	0
hsa-miR-133a-3p	-3.824	0	hsa-miR-45161	1.842	0
hsa-miR-1-3p	-3.136	0	hsa-miR-370-3p	1.609	0
hsa-miR-122-5p	-2.539	0	hsa-miR-190b-5p1	-0.968	0
hsa-miR-378a-5p	-0.658	0.001	hsa-miR-675-3p1	-0.914	0.006
hsa-miR-378a-3p	-0.703	0.001	hsa-miR-378a-5p2	0.529	0.014
hsa-miR-1283	1.505	0.002	hsa-miR-320c	0.955	0.014
hsa-miR-425-3p	-0.528	0.003	hsa-miR-1260b	0.508	0.014
hsa-miR-378i	-0.728	0.003	hsa-miR-328-3p1	0.738	0.016
hsa-miR-143-5p	-0.432	0.005	hsa-miR-409-3p	0.703	0.02
hsa-miR-4443	-1.228	0.005	hsa-miR-3261	0.518	0.02
hsa-miR-190b-5p	0.761	0.009	hsa-miR-29c-5p	0.462	0.02
hsa-miR-675-3p	0.837	0.012	hsa-miR-320d	0.902	0.025
hsa-miR-328-3p	-0.749	0.014	hsa-miR-193a-5p1	0.752	0.027
hsa-miR-326	-0.535	0.014	hsa-miR-127-3p	0.616	0.027
hsa-miR-615-3p	-0.649	0.016	hsa-miR-378i2	0.57	0.038
hsa-miR-193a-5p	-0.807	0.018	hsa-miR-12831	-1.146	0.038
hsa-miR-516a-5p	1.269	0.019	hsa-miR-196a-3p	-0.769	0.04
hsa-miR-22-3p	-0.323	0.021	hsa-miR-9983-3p	-1.024	0.043
hsa-miR-320b	-0.737	0.023	hsa-miR-934	-1.053	0.044
hsa-miR-21-3p	-0.535	0.023	hsa-miR-1468-5p	1.254	0.001
hsa-miR-30c-1-3p	-0.411	0.038	hsa-miR-9-5p1	1.414	0.001
hsa-miR-769-5p	-0.565	0.038	hsa-miR-9-3p1	1.368	0.001
hsa-miR-320a-3p	-0.711	0.044	hsa-miR-12832	-1.44	0.002
hsa-miR-122-5p1	-3.335	0	hsa-miR-9983-3p1	-1.162	0.008
hsa-miR-3168	2.466	0	hsa-miR-516a-5p1	-1.34	0.008
hsa-miR-4516	2.15	0	hsa-miR-675-3p2	-0.79	0.019
hsa-miR-4508	2.177	0	hsa-miR-196b-5p	0.423	0.033
hsa-miR-4488	2.349	0.004	hsa-miR-190b-5p2	-0.625	0.045
hsa-miR-21-3p1	-0.671	0.004	hsa-miR-2063	-5.363	0
hsa-miR-137-3p	1.138	0.012	hsa-miR-31682	-2.925	0
hsa-miR-425-3p1	-0.481	0.015	hsa-miR-1-3p3	-2.789	0
hsa-miR-2061	-5.636	0	hsa-miR-133a-3p3	-2.938	0
hsa-miR-133a-3p1	-3.32	0	hsa-miR-45082	-2.6	0
hsa-miR-122-5p2	-2.237	0	hsa-miR-45162	-2.055	0
hsa-miR-1-3p1	-2.547	0	hsa-miR-44882	-2.746	0
hsa-miR-9-3p	1.748	0	hsa-miR-9-3p2	1.578	0
hsa-miR-9-5p	1.4	0	hsa-miR-9-5p2	1.496	0
hsa-miR-378a-3p1	-0.637	0.002	hsa-miR-47911	0.905	0.001
hsa-miR-378a-5p1	-0.54	0.005	hsa-miR-455-5p	0.767	0.001
hsa-miR-378i1	-0.654	0.005	hsa-miR-1468-5p1	1.07	0.002
hsa-miR-577	1.011	0.006	hsa-miR-196b-5p1	0.477	0.004
hsa-miR-378d	-0.593	0.007	hsa-miR-489-3p	0.76	0.004
hsa-miR-4791	0.708	0.014	hsa-miR-320d1	-0.968	0.005
hsa-miR-44431	-0.994	0.028	hsa-miR-320c1	-0.89	0.012

hsa-miR-671-5p	-0.744	0.043	hsa-miR-455-3p	0.505	0.014
hsa-miR-1-3p2	3.378	0	hsa-miR-146a-3p	0.859	0.019
hsa-miR-2062	5.194	0	hsa-miR-29c-5p1	-0.429	0.02
hsa-miR-133a-3p2	3.442	0	hsa-miR-1260a	-0.406	0.036
hsa-miR-31681	2.188	0	hsa-miR-34a-5p	0.216	0.039
hsa-miR-45081	2.44	0	hsa-miR-1260b1	-0.414	0.043

Supplementary Table S8. Differentially expressed miRNAs among SPL_EBM_THEME1 groups

miRNA	logFC	FDR p-value
hsa-miR-517a-3p	-1.881	0
hsa-miR-675-3p	1.582	0.002
hsa-miR-675-5p	1.788	0.009
hsa-miR-509-3p	-1.31	0.016
hsa-miR-519c-5p	-1.455	0.016
hsa-miR-671-5p	1.289	0.023
hsa-miR-1307-5p	1.124	0.031
hsa-miR-517a-3p1	-1.775	0
hsa-miR-122-5p	3.448	0
hsa-miR-509-3p1	-1.775	0
hsa-miR-3168	-1.736	0
hsa-miR-122-5p1	1.659	0
hsa-miR-675-5p1	-1.007	0
hsa-miR-589-5p	-0.407	0.009
hsa-miR-671-5p1	-0.673	0.024
hsa-miR-449a	-0.784	0.024
hsa-miR-675-3p1	-0.602	0.026
hsa-miR-196b-5p	-0.336	0.036
hsa-miR-335-3p	-0.402	0.036
hsa-miR-7975	-0.515	0.043
hsa-miR-489-3p	0.492	0.043
hsa-miR-1307-5p1	-0.521	0.046

Supplementary Table S9. Differentially expressed miRNAs among EPL_BPHIGH groups

miRNA	logFC	FDR p-value	miRNA	logFC	FDR p-value
hsa-miR-193b-5p	-1.255	0	hsa-miR-577	0.866	0.017
hsa-miR-9-3p	1.509	0	hsa-miR-2110	-0.765	0.019
hsa-miR-127-3p	-0.837	0	hsa-miR-455-5p	0.55	0.019
hsa-miR-409-3p	-0.9	0	hsa-miR-423-3p	-0.364	0.019
hsa-miR-193a-5p	-0.988	0	hsa-miR-193b-3p	-0.481	0.019
hsa-miR-1275	-1.087	0	hsa-miR-182-5p	0.554	0.019
hsa-miR-122-5p	-1.729	0	hsa-miR-22-3p	-0.293	0.02
hsa-miR-423-5p	-0.71	0	hsa-miR-139-3p	-0.862	0.02
hsa-miR-551b-3p	-0.615	0	hsa-miR-181c-3p	0.433	0.02

hsa-miR-9-5p	1.348	0.001	hsa-miR-33a-5p	0.597	0.021
hsa-miR-499a-5p	-0.829	0.001	hsa-miR-9985	0.469	0.023
hsa-miR-93-5p	0.397	0.001	hsa-miR-92b-3p	0.323	0.023
hsa-miR-370-3p	-1.326	0.002	hsa-miR-18a-5p	0.571	0.026
hsa-miR-378a-3p	-0.563	0.002	hsa-miR-196a-5p	0.517	0.027
hsa-miR-134-5p	-0.572	0.003	hsa-miR-590-5p	0.494	0.028
hsa-miR-382-5p	-0.685	0.003	hsa-miR-96-5p	0.622	0.034
hsa-miR-485-5p	-0.922	0.003	hsa-miR-342-3p	-0.284	0.035
hsa-miR-320a-3p	-0.769	0.007	hsa-miR-491-5p	-0.383	0.035
hsa-miR-106b-5p	0.514	0.007	hsa-miR-19a-3p	0.609	0.038
hsa-miR-378a-5p	-0.461	0.009	hsa-miR-1283	-0.931	0.038
hsa-miR-181d-5p	0.455	0.01	hsa-miR-183-5p	0.532	0.039
hsa-miR-25-3p	0.289	0.01	hsa-miR-4791	0.593	0.042
hsa-miR-4497	-0.916	0.01	hsa-miR-30b-5p	0.306	0.042
hsa-miR-190b-5p	0.66	0.01	hsa-miR-342-5p	-0.403	0.042
hsa-miR-125b-1-3p	-0.433	0.01	hsa-miR-181c-5p	0.401	0.042
hsa-miR-378i	-0.542	0.01	hsa-miR-874-3p	-0.661	0.042
hsa-miR-328-3p	-0.681	0.01	hsa-miR-141-3p	0.56	0.046
hsa-miR-148a-3p	0.473	0.016	hsa-miR-135b-5p	0.752	0.046

Supplementary Table S10. Differentially expressed miRNAs among RPL_SVM_DOM3 groups

**miRNA**	**logFC**	**FDR p-value**	**miRNA**	**logFC**	**FDR p-value**
hsa-miR-3168	2.943	0	hsa-miR-12831	-1.491	0.003
hsa-miR-206	-4.502	0	hsa-miR-499a-5p	0.913	0.004
hsa-miR-133a-3p	-3.07	0	hsa-miR-44921	1.905	0.004
hsa-miR-1-3p	-2.471	0	hsa-miR-203a-3p	1.205	0.004
hsa-miR-122-5p	-2.14	0	hsa-miR-378i2	0.706	0.006
hsa-miR-1283	1.821	0	hsa-miR-184	-1.07	0.016
hsa-miR-378a-3p	-0.84	0	hsa-miR-193a-5p1	0.865	0.017
hsa-miR-516a-5p	1.627	0.001	hsa-miR-9983-3p	-1.198	0.017
hsa-miR-378i	-0.816	0.001	hsa-miR-516a-5p1	-1.311	0.022
hsa-miR-519c-5p	1.197	0.001	hsa-miR-378a-3p2	0.55	0.032
hsa-miR-675-3p	1.021	0.001	hsa-miR-196a-3p	-0.819	0.037
hsa-miR-320d	-1.188	0.002	hsa-miR-127-3p	0.659	0.037
hsa-miR-320c	-1.17	0.002	hsa-miR-137-3p1	1.039	0.037
hsa-miR-190b-5p	0.796	0.003	hsa-miR-31682	-3.221	0
hsa-miR-27a-5p	-0.552	0.004	hsa-miR-1468-5p1	1.359	0.002
hsa-miR-378d	-0.697	0.005	hsa-miR-12832	-1.534	0.003
hsa-miR-378a-5p	-0.571	0.005	hsa-miR-675-3p2	-0.992	0.006
hsa-miR-320b	-0.933	0.007	hsa-miR-519c-5p1	-1.129	0.007
hsa-miR-574-5p	-0.501	0.022	hsa-miR-9-3p1	1.242	0.009
hsa-miR-4443	-1.138	0.023	hsa-miR-516a-5p2	-1.42	0.009
hsa-miR-491-5p	-0.507	0.023	hsa-miR-9-5p1	1.278	0.009
hsa-miR-193b-5p	-0.971	0.023	hsa-miR-9983-3p1	-1.146	0.024
hsa-miR-193a-5p	-0.807	0.024	hsa-miR-190b-5p2	-0.685	0.034
hsa-miR-125b-1-3p	-0.467	0.027	hsa-miR-2063	-5.244	0

hsa-miR-320a-3p	-0.83	0.027	hsa-miR-1-3p3	-2.524	0
hsa-miR-122-5p1	-3.004	0	hsa-miR-133a-3p3	-2.607	0
hsa-miR-4516	1.837	0	hsa-miR-45082	-2.556	0
hsa-miR-4508	1.966	0.003	hsa-miR-45162	-2.042	0
hsa-miR-137-3p	1.143	0.003	hsa-miR-9-3p2	1.541	0
hsa-miR-4488	2.206	0.005	hsa-miR-44882	-2.781	0
hsa-miR-425-3p	-0.501	0.024	hsa-miR-47911	1.007	0
hsa-miR-4492	1.538	0.045	hsa-miR-455-5p	0.835	0
hsa-miR-21-3p	-0.525	0.045	hsa-miR-1468-5p2	1.334	0
hsa-miR-2061	-5.022	0	hsa-miR-44922	-2.09	0
hsa-miR-9-3p	1.856	0	hsa-miR-9-5p2	1.393	0
hsa-miR-133a-3p1	-2.474	0	hsa-miR-31683	-1.437	0.002
hsa-miR-122-5p2	-1.751	0	hsa-miR-455-3p	0.568	0.003
hsa-miR-9-5p	1.528	0	hsa-miR-489-3p	0.756	0.003
hsa-miR-1-3p1	-1.873	0	hsa-miR-34a-5p	0.295	0.003
hsa-miR-378d1	-0.705	0.001	hsa-miR-196b-5p1	0.44	0.012
hsa-miR-378a-3p1	-0.605	0.003	hsa-miR-17-5p1	0.548	0.012
hsa-miR-4791	0.728	0.012	hsa-miR-320d3	-0.963	0.014
hsa-miR-378i1	-0.58	0.016	hsa-miR-493-3p	0.577	0.015
hsa-miR-1468-5p	0.94	0.023	hsa-miR-122-5p3	1.253	0.018
hsa-miR-577	0.855	0.023	hsa-miR-499a-5p1	-0.697	0.018
hsa-miR-18a-5p	0.625	0.024	hsa-miR-889-3p	0.51	0.02
hsa-miR-196b-5p	0.388	0.033	hsa-miR-137-3p2	-0.955	0.02
hsa-miR-320d1	-0.802	0.033	hsa-miR-190a-5p	0.426	0.021
hsa-miR-378a-5p1	-0.43	0.039	hsa-miR-146a-3p	0.817	0.021
hsa-miR-17-5p	0.463	0.041	hsa-miR-27b-3p	0.304	0.024
hsa-miR-671-5p	-0.784	0.044	hsa-miR-320c2	-0.875	0.024
hsa-miR-1-3p2	3.122	0	hsa-miR-25-3p	0.279	0.024
hsa-miR-133a-3p2	3.203	0	hsa-miR-106b-5p	0.464	0.024
hsa-miR-2062	4.724	0	hsa-miR-141-3p	0.677	0.024
hsa-miR-44881	3.183	0	hsa-miR-493-5p	0.499	0.025
hsa-miR-190b-5p1	-1.084	0	hsa-miR-93-5p	0.3	0.034
hsa-miR-45161	1.996	0	hsa-miR-204-5p	-0.558	0.036
hsa-miR-675-3p1	-1.191	0	hsa-miR-335-3p	-0.441	0.043
hsa-miR-45081	2.328	0	hsa-miR-19a-3p	0.658	0.043
hsa-miR-370-3p	1.753	0	hsa-miR-148b-3p	0.286	0.044
hsa-miR-320d2	1.349	0.001	hsa-miR-340-5p	0.302	0.047
hsa-miR-320c1	1.312	0.001	hsa-miR-20a-5p	0.389	0.047
hsa-miR-31681	-1.784	0.001	hsa-miR-19b-3p	0.495	0.047

Supplementary Table S11. Differentially expressed miRNAs among TNBC stages

miRNA	logFC	FDR p-value	miRNA	logFC	FDR p-value
hsa-miR-339-5p	0.606	0	hsa-miR-335-3p	-0.396	0.012
hsa-miR-1275	1.124	0	hsa-miR-1307-3p	0.568	0.013
hsa-miR-1468-5p	1.193	0	hsa-miR-24-3p	-0.182	0.013
hsa-miR-3168	1.654	0	hsa-miR-3615	0.625	0.014

hsa-miR-671-5p	1.017	0	hsa-miR-365a-3p	-0.316	0.014
hsa-miR-29c-5p	0.541	0	hsa-let-7d-5p	-0.239	0.015
hsa-miR-7-5p	0.833	0	hsa-miR-139-5p	-0.477	0.016
hsa-miR-148a-5p	0.619	0	hsa-miR-5701	0.509	0.017
hsa-miR-4791	0.755	0	hsa-miR-147b-3p	0.611	0.017
hsa-miR-335-5p	-0.515	0	hsa-miR-138-5p	0.625	0.017
hsa-miR-190b-5p	0.661	0	hsa-miR-29a-5p	0.365	0.017
hsa-miR-769-5p	0.615	0	hsa-miR-20b-5p	0.397	0.018
hsa-miR-589-5p	0.483	0	hsa-miR-451a	-0.521	0.018
hsa-miR-339-3p	0.337	0.001	hsa-miR-320d	0.598	0.026
hsa-miR-1307-5p	0.718	0.001	hsa-miR-1247-5p	0.558	0.026
hsa-miR-320a-3p	0.748	0.001	hsa-let-7i-3p	0.259	0.026
hsa-miR-4497	0.843	0.001	hsa-miR-125b-2-3p	-0.341	0.027
hsa-miR-133a-3p	-1.521	0.001	hsa-miR-10a-5p	-0.263	0.028
hsa-miR-362-5p	0.37	0.001	hsa-miR-5100	0.412	0.028
hsa-miR-874-3p	0.743	0.002	hsa-miR-210-3p	0.424	0.028
hsa-miR-629-5p	0.296	0.002	hsa-let-7a-5p	-0.251	0.028
hsa-miR-628-3p	-0.367	0.002	hsa-miR-887-3p	0.473	0.029
hsa-miR-551b-3p	-0.499	0.002	hsa-miR-29b-2-5p	-0.32	0.029
hsa-miR-7704	0.855	0.003	hsa-miR-361-5p	-0.22	0.029
hsa-miR-342-5p	0.437	0.003	hsa-miR-2110	0.541	0.031
hsa-miR-574-5p	0.369	0.004	hsa-miR-320c	0.565	0.033
hsa-miR-12136	0.303	0.004	hsa-let-7e-5p	-0.229	0.034
hsa-miR-1-3p	-1.227	0.004	hsa-miR-148a-3p	0.314	0.038
hsa-miR-23b-3p	-0.354	0.006	hsa-miR-627-5p	0.383	0.042
hsa-miR-500a-3p	0.316	0.006	hsa-miR-425-3p	0.26	0.042
hsa-miR-188-5p	0.445	0.009	hsa-miR-675-3p	-0.502	0.043
hsa-miR-361-3p	0.278	0.01	hsa-miR-576-5p	0.26	0.043
hsa-miR-4516	0.916	0.011	hsa-miR-30a-3p	-0.252	0.043
hsa-miR-378i	-0.474	0.011	hsa-miR-92a-3p	-0.218	0.045
hsa-miR-9-3p	-0.81	0.011			

Supplementary Table S12. Differentially expressed miRNAs among SPL_EBM_THEME4 groups

**miRNA**	**logFC**	**FDR p-value**	**miRNA**	**logFC**	**FDR p-value**
hsa-miR-133a-3p	3.076	0	hsa-miR-378d	0.453	0.05
hsa-miR-1-3p	2.425	0	hsa-miR-361-5p	-0.265	0.05
hsa-miR-206	3.26	0	hsa-miR-31681	3.167	0
hsa-miR-499a-5p	0.843	0	hsa-miR-320d1	2.102	0
hsa-miR-1307-3p	-0.631	0.018	hsa-miR-320c2	2.058	0
hsa-miR-320c	-0.678	0.018	hsa-miR-44881	3.635	0
hsa-miR-95-3p	0.361	0.018	hsa-miR-671-5p1	1.515	0
hsa-miR-4443	-0.858	0.024	hsa-miR-769-5p2	1.037	0
hsa-miR-378i	0.461	0.041	hsa-miR-45081	2.587	0
hsa-miR-769-5p	-0.452	0.042	hsa-miR-1307-5p1	1.25	0
hsa-miR-3168	2.565	0	hsa-miR-45161	2.174	0
hsa-miR-4488	3.422	0	hsa-miR-370-3p1	1.721	0

hsa-miR-196a-3p	1.385	0	hsa-miR-1307-3p1	1.162	0
hsa-miR-320d	1.505	0	hsa-miR-122-5p1	-2.038	0
hsa-miR-370-3p	1.827	0	hsa-miR-133a-3p1	-2.598	0
hsa-miR-4516	2.129	0	hsa-miR-196a-3p1	1.108	0
hsa-miR-320c1	1.38	0	hsa-miR-320b	1.048	0
hsa-miR-122-5p	-2.15	0	hsa-miR-77041	1.362	0
hsa-miR-4508	2.35	0	hsa-miR-2061	-3.28	0
hsa-miR-127-3p	0.758	0	hsa-miR-12751	1.192	0
hsa-miR-409-3p	0.809	0.001	hsa-miR-1-3p1	-2.202	0
hsa-miR-671-5p	0.939	0.001	hsa-miR-425-3p1	0.581	0
hsa-miR-33a-5p	0.746	0.001	hsa-miR-320a-3p1	1.018	0
hsa-miR-382-5p	0.726	0.001	hsa-miR-517a-3p	-1.104	0
hsa-miR-1246	0.789	0.009	hsa-miR-574-5p	0.582	0
hsa-miR-222-3p	-0.339	0.009	hsa-miR-874-3p1	0.983	0
hsa-miR-483-3p	-0.811	0.009	hsa-miR-33a-5p1	0.751	0
hsa-miR-769-5p1	0.585	0.009	hsa-miR-127-3p1	0.698	0.001
hsa-miR-187-3p	0.807	0.011	hsa-miR-339-5p	0.475	0.001
hsa-miR-219a-5p	0.642	0.011	hsa-miR-12901	0.82	0.002
hsa-miR-1307-5p	0.71	0.014	hsa-miR-382-5p1	0.668	0.003
hsa-miR-584-5p	0.528	0.015	hsa-miR-483-3p1	-0.783	0.007
hsa-miR-7704	0.892	0.015	hsa-miR-887-3p	0.705	0.007
hsa-miR-425-3p	0.397	0.018	hsa-miR-47911	0.695	0.007
hsa-miR-335-5p	-0.457	0.02	hsa-miR-191-3p	0.595	0.008
hsa-let-7b-5p	-0.397	0.02	hsa-miR-2110	0.779	0.012
hsa-miR-542-3p	0.495	0.023	hsa-miR-409-3p1	0.62	0.012
hsa-miR-4791	0.635	0.026	hsa-miR-44431	0.996	0.012
hsa-miR-145-5p	-0.509	0.026	hsa-miR-12461	0.721	0.012
hsa-miR-33b-5p	0.703	0.026	hsa-miR-219a-5p1	0.59	0.012
hsa-miR-30d-3p	0.425	0.027	hsa-miR-151a-3p1	0.274	0.013
hsa-miR-1290	0.658	0.029	hsa-miR-20b-5p	-0.55	0.013
hsa-miR-196a-5p	0.517	0.029	hsa-miR-33b-5p1	0.687	0.016
hsa-miR-1275	0.717	0.033	hsa-miR-132-3p	-0.347	0.018
hsa-let-7c-5p	-0.498	0.035	hsa-miR-3615	0.757	0.018
hsa-miR-874-3p	0.644	0.039	hsa-miR-335-5p1	-0.434	0.019
hsa-miR-190b-5p	0.546	0.04	hsa-miR-769-3p	0.663	0.019
hsa-miR-26a-5p	-0.288	0.04	hsa-miR-338-3p	-0.354	0.031
hsa-miR-151a-3p	0.244	0.043	hsa-miR-499a-5p1	-0.622	0.036
hsa-miR-10b-5p	-0.345	0.049	hsa-miR-615-3p	0.51	0.036
hsa-miR-320a-3p	0.598	0.049	hsa-miR-363-3p	-0.48	0.044

Supplementary Table S13. Differentially expressed miRNAs among EPL_MINRTY groups

**miRNA**	**logFC**	**FDR p-value**
hsa-miR-190b-5p	0.693	0.001
hsa-miR-675-3p	-0.724	0.004
hsa-miR-9-3p	0.979	0.004
hsa-miR-31-5p	-0.454	0.037

Supplementary Table S14. Differentially expressed miRNAs among SPL_SVM_DOM1 groups

miRNA	logFC	FDR p-value
hsa-miR-190b-5p	0.693	0.001
hsa-miR-675-3p	-0.724	0.004
hsa-miR-9-3p	0.979	0.004
hsa-miR-31-5p	-0.454	0.037

Supplementary Table S15. Differentially expressed miRNAs among RPL_SVM_DOM1 groups

miRNA	logFC	FDR p-value
hsa-miR-190b-5p	0.693	0.001
hsa-miR-675-3p	-0.724	0.004
hsa-miR-9-3p	0.979	0.004
hsa-miR-31-5p	-0.454	0.037

Supplementary Table S16. Differentially expressed miRNAs among EPL_DISABL groups

miRNA	logFC	FDR p-value
hsa-miR-671-5p	1.14	0
hsa-miR-675-3p	-0.915	0
hsa-miR-1307-5p	0.909	0
hsa-miR-769-5p	0.703	0
hsa-miR-320c	0.931	0
hsa-miR-320d	0.915	0
hsa-miR-133a-3p	1.768	0.001
hsa-miR-1307-3p	0.756	0.001
hsa-miR-1-3p	1.543	0.001
hsa-miR-1468-5p	0.828	0.001
hsa-miR-320a-3p	0.717	0.002
hsa-miR-378d	0.523	0.002
hsa-miR-574-5p	0.386	0.003
hsa-miR-4508	1.232	0.003
hsa-miR-4516	1.048	0.004
hsa-miR-33b-5p	0.643	0.009
hsa-miR-874-3p	0.621	0.01
hsa-miR-7704	0.736	0.015
hsa-miR-21-3p	-0.413	0.015
hsa-miR-151a-3p	0.225	0.015
hsa-miR-320b	0.51	0.033
hsa-miR-33a-5p	0.459	0.044
hsa-miR-425-3p	0.285	0.049
hsa-miR-138-5p	0.575	0.05

Supplementary Table S17. Differentially expressed miRNAs among EP_DISABL groups

miRNA	logFC	FDR p-value
hsa-miR-206	5.442	0
hsa-miR-671-5p	1.063	0
hsa-miR-1307-5p	0.862	0
hsa-miR-769-5p	0.669	0
hsa-miR-320c	0.911	0
hsa-miR-1468-5p	0.926	0
hsa-miR-320d	0.841	0.001
hsa-miR-1307-3p	0.685	0.007
hsa-miR-21-3p	-0.465	0.007
hsa-miR-1-3p	1.386	0.007
hsa-miR-133a-3p	1.553	0.007
hsa-miR-4508	1.171	0.007
hsa-miR-320a-3p	0.651	0.007
hsa-miR-378d	0.46	0.014
hsa-miR-7704	0.742	0.014
hsa-miR-33b-5p	0.632	0.014
hsa-miR-4516	0.923	0.018
hsa-miR-574-5p	0.338	0.019
hsa-miR-874-3p	0.578	0.02
hsa-miR-19a-3p	0.563	0.02
hsa-miR-151a-3p	0.228	0.02
hsa-miR-33a-5p	0.467	0.039
hsa-miR-320b	0.499	0.04
hsa-miR-3168	0.978	0.044
hsa-miR-1283	-0.844	0.048
hsa-miR-7-5p	0.513	0.049
hsa-miR-188-5p	0.396	0.049

Supplementary Table S18. Differentially expressed miRNAs among EPL_DIABETES groups

miRNA	logFC	FDR p-value
hsa-miR-21-3p	-0.656	0.001
hsa-miR-224-5p	-0.748	0.001
hsa-miR-335-3p	-0.602	0.001
hsa-miR-122-5p	-1.666	0.001
hsa-miR-499a-5p	-0.751	0.01
hsa-miR-9-3p	1.123	0.015
hsa-miR-551b-3p	-0.511	0.016
hsa-miR-151a-3p	0.315	0.022
hsa-miR-335-5p	-0.425	0.022
hsa-miR-190b-5p	0.659	0.022
hsa-miR-378i	-0.519	0.046
hsa-miR-378a-3p	-0.468	0.048

Supplementary Table S19. Differentially expressed miRNAs among FormTot groups

miRNA	logFC	FDR p-value	miRNA	logFC	FDR p-value
hsa-miR-320d	1.577	0	hsa-miR-589-5p	0.335	0.049
hsa-miR-320c	1.513	0	hsa-miR-425-3p	0.289	0.049
hsa-miR-206	-4.044	0	hsa-miR-122-5p	2.46	0
hsa-miR-4488	2.664	0	hsa-miR-1269a	1.682	0.025
hsa-miR-671-5p	1.179	0	hsa-miR-519a-3p	-1.547	0.033
hsa-miR-675-3p	1.054	0	hsa-miR-31681	1.312	0.033
hsa-miR-1307-5p	0.925	0	hsa-miR-1283	-1.58	0.033
hsa-miR-4516	1.618	0	hsa-miR-203b-3p	-1.155	0.033
hsa-miR-129-5p	1.166	0	hsa-miR-516a-5p	-1.627	0.033
hsa-miR-769-5p	0.692	0	hsa-miR-1468-5p	-1.029	0.033
hsa-miR-21-3p	0.655	0	hsa-miR-2061	4.65	0
hsa-miR-133a-3p	-2.085	0	hsa-miR-122-5p1	2.327	0
hsa-miR-582-3p	0.808	0	hsa-miR-44881	-3.148	0
hsa-miR-1-3p	-1.803	0	hsa-miR-519a-3p1	-2.151	0
hsa-miR-4492	1.791	0	hsa-miR-769-5p1	-0.99	0
hsa-miR-3960	1.706	0	hsa-miR-12831	-2.188	0.001
hsa-miR-4508	1.723	0	hsa-miR-516a-5p1	-2.252	0.001
hsa-miR-3200-3p	0.601	0	hsa-miR-1307-5p1	-1.197	0.001
hsa-miR-934	1.101	0	hsa-miR-203b-3p1	-1.342	0.007
hsa-miR-449a	0.982	0	hsa-miR-44921	-2.178	0.007
hsa-miR-339-5p	0.417	0	hsa-miR-33b-5p	-1.117	0.012
hsa-miR-370-3p	1.179	0	hsa-miR-45161	-1.634	0.016
hsa-miR-4791	0.695	0.001	hsa-miR-12751	-1.085	0.017
hsa-miR-320a-3p	0.727	0.001	hsa-miR-29c-5p	-0.56	0.02
hsa-miR-1307-3p	0.698	0.002	hsa-miR-45081	-1.889	0.023
hsa-miR-1275	0.783	0.002	hsa-miR-339-5p1	-0.504	0.023
hsa-miR-151a-3p	0.272	0.002	hsa-miR-147b-3p	-1.089	0.023
hsa-miR-301b-3p	0.662	0.003	hsa-miR-129-5p1	-1.193	0.029
hsa-miR-7704	0.781	0.016	hsa-miR-330-5p	-0.735	0.031
hsa-miR-3168	1.062	0.018	hsa-miR-675-3p1	-0.917	0.031
hsa-miR-874-3p	0.608	0.022	hsa-miR-39601	-1.823	0.037
hsa-miR-33a-5p	0.474	0.03	hsa-miR-320d1	-0.955	0.04
hsa-miR-127-3p	0.458	0.031	hsa-miR-3200-3p1	-0.65	0.04
hsa-miR-499a-5p	-0.562	0.035	hsa-miR-47911	-0.814	0.043
hsa-miR-574-5p	0.3	0.047			

Supplementary Table S20. Differentially expressed miRNAs linked to each of the target genes (including only target genes with 15 or more differentially expressed miRNAs)

Target gene	miRNA counts	miRNA names
SRRM2	31	hsa-miR-769-5p, hsa-miR-148a-3p, hsa-miR-20b-5p, hsa-miR-335-5p, hsa-miR-7-5p, hsa-miR-138-5p, hsa-miR-361-5p, hsa-miR-574-5p, hsa-miR-589-5p, hsa-miR-188-5p, hsa-miR-320d, hsa-miR-1275, hsa-miR-629-5p, hsa-miR-29c-5p, hsa-miR-361-3p, hsa-miR-342-5p, hsa-miR-335-3p, hsa-miR-425-3p, hsa-miR-500a-3p, hsa-miR-671-5p, hsa-miR-1307-3p, hsa-miR-1307-5p, hsa-miR-2110, hsa-miR-320c, hsa-miR-3615, hsa-miR-378i, hsa-miR-1468-5p, hsa-miR-1-3p, hsa-miR-887-3p, hsa-miR-320a-3p, hsa-miR-147b-3p
HUWE1	30	hsa-miR-361-5p, hsa-miR-148a-3p, hsa-miR-20b-5p, hsa-miR-335-5p, hsa-miR-7-5p, hsa-miR-138-5p, hsa-miR-339-5p, hsa-miR-574-5p, hsa-miR-589-5p, hsa-miR-769-5p, hsa-miR-320d, hsa-miR-1275, hsa-miR-629-5p, hsa-miR-361-3p, hsa-miR-342-5p, hsa-miR-335-3p, hsa-miR-425-3p, hsa-miR-500a-3p, hsa-miR-671-5p, hsa-miR-1307-3p, hsa-miR-2110, hsa-miR-320c, hsa-miR-3615, hsa-miR-378i, hsa-miR-874-3p, hsa-miR-1468-5p, hsa-miR-1-3p, hsa-miR-133a-3p, hsa-miR-320a-3p, hsa-miR-147b-3p
KMT2D	30	hsa-miR-1275, hsa-miR-148a-3p, hsa-miR-20b-5p, hsa-miR-335-5p, hsa-miR-7-5p, hsa-miR-9-3p, hsa-miR-138-5p, hsa-miR-361-5p, hsa-miR-339-5p, hsa-miR-574-5p, hsa-miR-589-5p, hsa-miR-769-5p, hsa-miR-188-5p, hsa-miR-320d, hsa-miR-629-5p, hsa-miR-361-3p, hsa-miR-335-3p, hsa-miR-425-3p, hsa-miR-500a-3p, hsa-miR-551b-3p, hsa-miR-671-5p, hsa-miR-1307-3p, hsa-miR-1307-5p, hsa-miR-2110, hsa-miR-320c, hsa-miR-378i, hsa-miR-1468-5p, hsa-miR-1-3p, hsa-miR-320a-3p, hsa-miR-147b-3p
ADNP	29	hsa-miR-148a-3p, hsa-miR-20b-5p, hsa-miR-335-5p, hsa-miR-7-5p, hsa-miR-9-3p, hsa-miR-138-5p, hsa-miR-361-5p, hsa-miR-339-5p, hsa-miR-574-5p, hsa-miR-769-5p, hsa-miR-188-5p, hsa-miR-320d, hsa-miR-629-5p, hsa-miR-29c-5p, hsa-miR-342-5p, hsa-miR-335-3p, hsa-miR-425-3p, hsa-miR-671-5p, hsa-miR-3168, hsa-miR-2110, hsa-miR-320c, hsa-miR-378i, hsa-miR-874-3p, hsa-miR-1-3p, hsa-miR-133a-3p, hsa-miR-887-3p, hsa-miR-320a-3p, hsa-miR-147b-3p, hsa-miR-190b-5p
AGO2	29	hsa-miR-148a-3p, hsa-miR-1275, hsa-miR-671-5p, hsa-miR-20b-5p, hsa-miR-7-5p, hsa-miR-9-3p, hsa-miR-138-5p, hsa-miR-361-5p, hsa-miR-339-5p, hsa-miR-574-5p, hsa-miR-589-5p, hsa-miR-769-5p, hsa-

		miR-320d, hsa-miR-629-5p, hsa-miR-29c-5p, hsa-miR-361-3p, hsa-miR-335-3p, hsa-miR-500a-3p, hsa-miR-1307-3p, hsa-miR-3168, hsa-miR-2110, hsa-miR-320c, hsa-miR-3615, hsa-miR-378i, hsa-miR-874-3p, hsa-miR-1-3p, hsa-miR-887-3p, hsa-miR-320a-3p, hsa-miR-190b-5p
KMT2A	29	hsa-miR-335-5p, hsa-miR-671-5p, hsa-miR-148a-3p, hsa-miR-20b-5p, hsa-miR-7-5p, hsa-miR-138-5p, hsa-miR-361-5p, hsa-miR-339-5p, hsa-miR-574-5p, hsa-miR-589-5p, hsa-miR-769-5p, hsa-miR-188-5p, hsa-miR-320d, hsa-miR-629-5p, hsa-miR-342-5p, hsa-miR-335-3p, hsa-miR-425-3p, hsa-miR-500a-3p, hsa-miR-1307-3p, hsa-miR-1307-5p, hsa-miR-2110, hsa-miR-320c, hsa-miR-3615, hsa-miR-378i, hsa-miR-1-3p, hsa-miR-133a-3p, hsa-miR-887-3p, hsa-miR-320a-3p, hsa-miR-147b-3p
DYNC1H1	28	hsa-miR-148a-3p, hsa-miR-20b-5p, hsa-miR-335-5p, hsa-miR-7-5p, hsa-miR-138-5p, hsa-miR-361-5p, hsa-miR-339-5p, hsa-miR-574-5p, hsa-miR-589-5p, hsa-miR-769-5p, hsa-miR-188-5p, hsa-miR-320d, hsa-miR-629-5p, hsa-miR-361-3p, hsa-miR-342-5p, hsa-miR-335-3p, hsa-miR-425-3p, hsa-miR-500a-3p, hsa-miR-671-5p, hsa-miR-1307-3p, hsa-miR-1307-5p, hsa-miR-2110, hsa-miR-320c, hsa-miR-3615, hsa-miR-378i, hsa-miR-1-3p, hsa-miR-320a-3p, hsa-miR-147b-3p
MAP1B	28	hsa-miR-7-5p, hsa-miR-148a-3p, hsa-miR-20b-5p, hsa-miR-335-5p, hsa-miR-9-3p, hsa-miR-138-5p, hsa-miR-361-5p, hsa-miR-589-5p, hsa-miR-769-5p, hsa-miR-188-5p, hsa-miR-320d, hsa-miR-629-5p, hsa-miR-342-5p, hsa-miR-335-3p, hsa-miR-425-3p, hsa-miR-500a-3p, hsa-miR-671-5p, hsa-miR-1307-3p, hsa-miR-1307-5p, hsa-miR-675-3p, hsa-miR-2110, hsa-miR-320c, hsa-miR-378i, hsa-miR-1468-5p, hsa-miR-1-3p, hsa-miR-133a-3p, hsa-miR-320a-3p, hsa-miR-147b-3p
MBNL1	28	hsa-miR-335-5p, hsa-miR-188-5p, hsa-miR-335-3p, hsa-miR-148a-3p, hsa-miR-20b-5p, hsa-miR-7-5p, hsa-miR-9-3p, hsa-miR-138-5p, hsa-miR-361-5p, hsa-miR-574-5p, hsa-miR-769-5p, hsa-miR-320d, hsa-miR-1275, hsa-miR-629-5p, hsa-miR-361-3p, hsa-miR-342-5p, hsa-miR-425-3p, hsa-miR-500a-3p, hsa-miR-671-5p, hsa-miR-2110, hsa-miR-320c, hsa-miR-3615, hsa-miR-378i, hsa-miR-874-3p, hsa-miR-1-3p, hsa-miR-320a-3p, hsa-miR-147b-3p, hsa-miR-190b-5p
PRPF8	28	hsa-miR-361-5p, hsa-miR-148a-3p, hsa-miR-20b-5p, hsa-miR-335-5p, hsa-miR-7-5p, hsa-miR-138-5p, hsa-miR-339-5p, hsa-miR-574-5p, hsa-miR-589-5p, hsa-miR-769-5p, hsa-miR-320d, hsa-miR-1275, hsa-miR-629-5p, hsa-miR-361-3p, hsa-miR-342-5p, hsa-miR-335-3p, hsa-miR-500a-3p, hsa-miR-671-5p, hsa-miR-1307-3p, hsa-miR-1307-5p, hsa-

		miR-2110, hsa-miR-320c, hsa-miR-3615, hsa-miR-378i, hsa-miR-1-3p, hsa-miR-320a-3p, hsa-miR-147b-3p, hsa-miR-190b-5p
RC3H2	28	hsa-miR-148a-3p, hsa-miR-20b-5p, hsa-miR-335-5p, hsa-miR-7-5p, hsa-miR-9-3p, hsa-miR-138-5p, hsa-miR-361-5p, hsa-miR-339-5p, hsa-miR-574-5p, hsa-miR-589-5p, hsa-miR-320d, hsa-miR-629-5p, hsa-miR-29c-5p, hsa-miR-361-3p, hsa-miR-335-3p, hsa-miR-425-3p, hsa-miR-500a-3p, hsa-miR-551b-3p, hsa-miR-671-5p, hsa-miR-1307-3p, hsa-miR-2110, hsa-miR-320c, hsa-miR-3615, hsa-miR-378i, hsa-miR-874-3p, hsa-miR-1-3p, hsa-miR-887-3p, hsa-miR-320a-3p
SETD5	28	hsa-miR-148a-3p, hsa-miR-20b-5p, hsa-miR-335-5p, hsa-miR-7-5p, hsa-miR-138-5p, hsa-miR-361-5p, hsa-miR-339-5p, hsa-miR-574-5p, hsa-miR-589-5p, hsa-miR-769-5p, hsa-miR-188-5p, hsa-miR-1275, hsa-miR-629-5p, hsa-miR-361-3p, hsa-miR-342-5p, hsa-miR-335-3p, hsa-miR-500a-3p, hsa-miR-671-5p, hsa-miR-1307-3p, hsa-miR-1307-5p, hsa-miR-3168, hsa-miR-2110, hsa-miR-378i, hsa-miR-1468-5p, hsa-miR-1-3p, hsa-miR-887-3p, hsa-miR-320a-3p, hsa-miR-147b-3p
SPEN	28	hsa-miR-769-5p, hsa-miR-148a-3p, hsa-miR-20b-5p, hsa-miR-335-5p, hsa-miR-7-5p, hsa-miR-138-5p, hsa-miR-361-5p, hsa-miR-339-5p, hsa-miR-574-5p, hsa-miR-589-5p, hsa-miR-320d, hsa-miR-1275, hsa-miR-29c-5p, hsa-miR-361-3p, hsa-miR-342-5p, hsa-miR-335-3p, hsa-miR-425-3p, hsa-miR-500a-3p, hsa-miR-671-5p, hsa-miR-1307-3p, hsa-miR-2110, hsa-miR-320c, hsa-miR-3615, hsa-miR-378i, hsa-miR-1468-5p, hsa-miR-133a-3p, hsa-miR-320a-3p, hsa-miR-147b-3p
ASH1L	27	hsa-miR-361-5p, hsa-miR-574-5p, hsa-miR-148a-3p, hsa-miR-20b-5p, hsa-miR-335-5p, hsa-miR-7-5p, hsa-miR-138-5p, hsa-miR-339-5p, hsa-miR-769-5p, hsa-miR-1275, hsa-miR-629-5p, hsa-miR-29c-5p, hsa-miR-342-5p, hsa-miR-335-3p, hsa-miR-425-3p, hsa-miR-500a-3p, hsa-miR-671-5p, hsa-miR-1307-5p, hsa-miR-675-3p, hsa-miR-2110, hsa-miR-320c, hsa-miR-378i, hsa-miR-874-3p, hsa-miR-1-3p, hsa-miR-133a-3p, hsa-miR-320a-3p, hsa-miR-147b-3p
CLTC	27	hsa-miR-1-3p, hsa-miR-148a-3p, hsa-miR-20b-5p, hsa-miR-335-5p, hsa-miR-7-5p, hsa-miR-9-3p, hsa-miR-138-5p, hsa-miR-361-5p, hsa-miR-574-5p, hsa-miR-589-5p, hsa-miR-320d, hsa-miR-629-5p, hsa-miR-361-3p, hsa-miR-342-5p, hsa-miR-335-3p, hsa-miR-425-3p, hsa-miR-500a-3p, hsa-miR-671-5p, hsa-miR-675-3p, hsa-miR-2110, hsa-miR-320c, hsa-miR-3615, hsa-miR-378i, hsa-miR-133a-3p, hsa-miR-887-3p, hsa-miR-320a-3p, hsa-miR-147b-3p

NUFIP2	27	hsa-miR-20b-5p, hsa-miR-342-5p, hsa-miR-335-3p, hsa-miR-4516, hsa-miR-874-3p, hsa-miR-148a-3p, hsa-miR-7-5p, hsa-miR-138-5p, hsa-miR-361-5p, hsa-miR-339-5p, hsa-miR-574-5p, hsa-miR-589-5p, hsa-miR-320d, hsa-miR-629-5p, hsa-miR-29c-5p, hsa-miR-361-3p, hsa-miR-500a-3p, hsa-miR-671-5p, hsa-miR-2110, hsa-miR-320c, hsa-miR-3615, hsa-miR-378i, hsa-miR-1468-5p, hsa-miR-1-3p, hsa-miR-133a-3p, hsa-miR-320a-3p, hsa-miR-190b-5p
TAOK1	27	hsa-miR-1275, hsa-miR-148a-3p, hsa-miR-20b-5p, hsa-miR-335-5p, hsa-miR-7-5p, hsa-miR-9-3p, hsa-miR-138-5p, hsa-miR-361-5p, hsa-miR-339-5p, hsa-miR-589-5p, hsa-miR-769-5p, hsa-miR-320d, hsa-miR-629-5p, hsa-miR-29c-5p, hsa-miR-335-3p, hsa-miR-500a-3p, hsa-miR-551b-3p, hsa-miR-671-5p, hsa-miR-1307-5p, hsa-miR-2110, hsa-miR-320c, hsa-miR-378i, hsa-miR-874-3p, hsa-miR-1-3p, hsa-miR-133a-3p, hsa-miR-320a-3p, hsa-miR-147b-3p
CELF1	26	hsa-miR-138-5p, hsa-miR-4791, hsa-miR-148a-3p, hsa-miR-20b-5p, hsa-miR-335-5p, hsa-miR-7-5p, hsa-miR-361-5p, hsa-miR-339-5p, hsa-miR-589-5p, hsa-miR-769-5p, hsa-miR-320d, hsa-miR-629-5p, hsa-miR-29c-5p, hsa-miR-361-3p, hsa-miR-335-3p, hsa-miR-500a-3p, hsa-miR-671-5p, hsa-miR-2110, hsa-miR-320c, hsa-miR-3615, hsa-miR-378i, hsa-miR-1-3p, hsa-miR-133a-3p, hsa-miR-320a-3p, hsa-miR-147b-3p, hsa-miR-190b-5p
CREBBP	26	hsa-miR-769-5p, hsa-miR-148a-3p, hsa-miR-20b-5p, hsa-miR-335-5p, hsa-miR-7-5p, hsa-miR-9-3p, hsa-miR-138-5p, hsa-miR-361-5p, hsa-miR-574-5p, hsa-miR-589-5p, hsa-miR-629-5p, hsa-miR-342-5p, hsa-miR-335-3p, hsa-miR-425-3p, hsa-miR-500a-3p, hsa-miR-671-5p, hsa-miR-1307-3p, hsa-miR-675-3p, hsa-miR-2110, hsa-miR-320c, hsa-miR-3615, hsa-miR-1-3p, hsa-miR-133a-3p, hsa-miR-887-3p, hsa-miR-320a-3p, hsa-miR-147b-3p
CSDE1	26	hsa-miR-20b-5p, hsa-miR-148a-3p, hsa-miR-335-5p, hsa-miR-7-5p, hsa-miR-9-3p, hsa-miR-138-5p, hsa-miR-361-5p, hsa-miR-589-5p, hsa-miR-769-5p, hsa-miR-320d, hsa-miR-1275, hsa-miR-29c-5p, hsa-miR-361-3p, hsa-miR-342-5p, hsa-miR-335-3p, hsa-miR-500a-3p, hsa-miR-671-5p, hsa-miR-2110, hsa-miR-320c, hsa-miR-3615, hsa-miR-378i, hsa-miR-133a-3p, hsa-miR-887-3p, hsa-miR-320a-3p, hsa-miR-147b-3p, hsa-miR-190b-5p
DICER1	26	hsa-miR-148a-3p, hsa-miR-20b-5p, hsa-miR-335-5p, hsa-miR-7-5p, hsa-miR-9-3p, hsa-miR-138-5p, hsa-miR-361-5p, hsa-miR-574-5p, hsa-miR-769-5p, hsa-miR-320d, hsa-miR-629-5p, hsa-miR-29c-5p, hsa-miR-342-

		5p, hsa-miR-335-3p, hsa-miR-500a-3p, hsa-miR-671-5p, hsa-miR-1307-3p, hsa-miR-3168, hsa-miR-2110, hsa-miR-320c, hsa-miR-3615, hsa-miR-1-3p, hsa-miR-887-3p, hsa-miR-320a-3p, hsa-miR-147b-3p, hsa-miR-190b-5p
DYRK1A	26	hsa-miR-148a-3p, hsa-miR-335-5p, hsa-miR-335-3p, hsa-miR-20b-5p, hsa-miR-7-5p, hsa-miR-361-5p, hsa-miR-339-5p, hsa-miR-574-5p, hsa-miR-589-5p, hsa-miR-769-5p, hsa-miR-320d, hsa-miR-1275, hsa-miR-629-5p, hsa-miR-29c-5p, hsa-miR-361-3p, hsa-miR-342-5p, hsa-miR-500a-3p, hsa-miR-551b-3p, hsa-miR-671-5p, hsa-miR-1307-5p, hsa-miR-320c, hsa-miR-378i, hsa-miR-874-3p, hsa-miR-133a-3p, hsa-miR-320a-3p, hsa-miR-147b-3p
EIF4G2	26	hsa-miR-20b-5p, hsa-miR-148a-3p, hsa-miR-335-5p, hsa-miR-7-5p, hsa-miR-138-5p, hsa-miR-361-5p, hsa-miR-339-5p, hsa-miR-574-5p, hsa-miR-589-5p, hsa-miR-769-5p, hsa-miR-188-5p, hsa-miR-320d, hsa-miR-1275, hsa-miR-629-5p, hsa-miR-361-3p, hsa-miR-342-5p, hsa-miR-335-3p, hsa-miR-425-3p, hsa-miR-500a-3p, hsa-miR-320c, hsa-miR-378i, hsa-miR-1-3p, hsa-miR-133a-3p, hsa-miR-887-3p, hsa-miR-320a-3p, hsa-miR-147b-3p
FASN	26	hsa-miR-2110, hsa-miR-1-3p, hsa-miR-148a-3p, hsa-miR-20b-5p, hsa-miR-335-5p, hsa-miR-7-5p, hsa-miR-138-5p, hsa-miR-361-5p, hsa-miR-339-5p, hsa-miR-574-5p, hsa-miR-769-5p, hsa-miR-320d, hsa-miR-629-5p, hsa-miR-361-3p, hsa-miR-342-5p, hsa-miR-335-3p, hsa-miR-425-3p, hsa-miR-500a-3p, hsa-miR-1307-3p, hsa-miR-1307-5p, hsa-miR-320c, hsa-miR-3615, hsa-miR-378i, hsa-miR-133a-3p, hsa-miR-320a-3p, hsa-miR-147b-3p
FAT1	26	hsa-miR-148a-3p, hsa-miR-20b-5p, hsa-miR-335-5p, hsa-miR-7-5p, hsa-miR-138-5p, hsa-miR-361-5p, hsa-miR-339-5p, hsa-miR-574-5p, hsa-miR-589-5p, hsa-miR-769-5p, hsa-miR-188-5p, hsa-miR-342-5p, hsa-miR-335-3p, hsa-miR-425-3p, hsa-miR-500a-3p, hsa-miR-671-5p, hsa-miR-1307-3p, hsa-miR-2110, hsa-miR-320c, hsa-miR-3615, hsa-miR-378i, hsa-miR-1-3p, hsa-miR-133a-3p, hsa-miR-887-3p, hsa-miR-320a-3p, hsa-miR-147b-3p
FLNA	26	hsa-miR-7-5p, hsa-miR-1-3p, hsa-miR-148a-3p, hsa-miR-20b-5p, hsa-miR-335-5p, hsa-miR-9-3p, hsa-miR-138-5p, hsa-miR-361-5p, hsa-miR-339-5p, hsa-miR-574-5p, hsa-miR-589-5p, hsa-miR-769-5p, hsa-miR-320d, hsa-miR-361-3p, hsa-miR-425-3p, hsa-miR-500a-3p, hsa-miR-671-5p, hsa-miR-1307-5p, hsa-miR-2110, hsa-miR-320c, hsa-miR-3615,

		hsa-miR-378i, hsa-miR-874-3p, hsa-miR-133a-3p, hsa-miR-320a-3p, hsa-miR-147b-3p
KLHL15	26	hsa-miR-20b-5p, hsa-miR-335-5p, hsa-miR-9-3p, hsa-miR-361-5p, hsa-miR-320d, hsa-miR-425-3p, hsa-miR-500a-3p, hsa-miR-675-3p, hsa-miR-320c, hsa-miR-148a-3p, hsa-miR-7-5p, hsa-miR-138-5p, hsa-miR-574-5p, hsa-miR-589-5p, hsa-miR-629-5p, hsa-miR-29c-5p, hsa-miR-342-5p, hsa-miR-335-3p, hsa-miR-671-5p, hsa-miR-2110, hsa-miR-378i, hsa-miR-1-3p, hsa-miR-133a-3p, hsa-miR-887-3p, hsa-miR-320a-3p, hsa-miR-147b-3p
KPNB1	26	hsa-miR-7-5p, hsa-miR-148a-3p, hsa-miR-20b-5p, hsa-miR-335-5p, hsa-miR-9-3p, hsa-miR-138-5p, hsa-miR-361-5p, hsa-miR-339-5p, hsa-miR-574-5p, hsa-miR-589-5p, hsa-miR-188-5p, hsa-miR-320d, hsa-miR-629-5p, hsa-miR-361-3p, hsa-miR-335-3p, hsa-miR-425-3p, hsa-miR-500a-3p, hsa-miR-1307-5p, hsa-miR-3168, hsa-miR-2110, hsa-miR-320c, hsa-miR-378i, hsa-miR-874-3p, hsa-miR-1-3p, hsa-miR-133a-3p, hsa-miR-320a-3p
PRRC2A	26	hsa-miR-148a-3p, hsa-miR-20b-5p, hsa-miR-335-5p, hsa-miR-7-5p, hsa-miR-138-5p, hsa-miR-361-5p, hsa-miR-339-5p, hsa-miR-574-5p, hsa-miR-589-5p, hsa-miR-769-5p, hsa-miR-320d, hsa-miR-1275, hsa-miR-361-3p, hsa-miR-342-5p, hsa-miR-335-3p, hsa-miR-425-3p, hsa-miR-500a-3p, hsa-miR-671-5p, hsa-miR-2110, hsa-miR-320c, hsa-miR-3615, hsa-miR-378i, hsa-miR-874-3p, hsa-miR-133a-3p, hsa-miR-320a-3p, hsa-miR-147b-3p
SMG1	26	hsa-miR-335-5p, hsa-miR-361-5p, hsa-miR-1307-3p, hsa-miR-148a-3p, hsa-miR-20b-5p, hsa-miR-7-5p, hsa-miR-9-3p, hsa-miR-138-5p, hsa-miR-574-5p, hsa-miR-769-5p, hsa-miR-320d, hsa-miR-629-5p, hsa-miR-361-3p, hsa-miR-342-5p, hsa-miR-335-3p, hsa-miR-425-3p, hsa-miR-500a-3p, hsa-miR-671-5p, hsa-miR-2110, hsa-miR-320c, hsa-miR-378i, hsa-miR-874-3p, hsa-miR-1-3p, hsa-miR-887-3p, hsa-miR-320a-3p, hsa-miR-147b-3p
TET3	26	hsa-miR-20b-5p, hsa-miR-2110, hsa-miR-148a-3p, hsa-miR-335-5p, hsa-miR-7-5p, hsa-miR-9-3p, hsa-miR-138-5p, hsa-miR-361-5p, hsa-miR-339-5p, hsa-miR-574-5p, hsa-miR-589-5p, hsa-miR-769-5p, hsa-miR-320d, hsa-miR-1275, hsa-miR-335-3p, hsa-miR-425-3p, hsa-miR-500a-3p, hsa-miR-671-5p, hsa-miR-1307-3p, hsa-miR-1307-5p, hsa-miR-320c, hsa-miR-3615, hsa-miR-378i, hsa-miR-1-3p, hsa-miR-320a-3p, hsa-miR-147b-3p

TNRC6A	26	hsa-miR-148a-3p, hsa-miR-20b-5p, hsa-miR-7-5p, hsa-miR-361-5p, hsa-miR-9-3p, hsa-miR-138-5p, hsa-miR-574-5p, hsa-miR-589-5p, hsa-miR-769-5p, hsa-miR-188-5p, hsa-miR-320d, hsa-miR-1275, hsa-miR-629-5p, hsa-miR-361-3p, hsa-miR-342-5p, hsa-miR-335-3p, hsa-miR-671-5p, hsa-miR-1307-3p, hsa-miR-1307-5p, hsa-miR-2110, hsa-miR-320c, hsa-miR-378i, hsa-miR-133a-3p, hsa-miR-320a-3p, hsa-miR-147b-3p, hsa-miR-190b-5p
ZFHX3	26	hsa-miR-148a-3p, hsa-miR-20b-5p, hsa-miR-335-5p, hsa-miR-7-5p, hsa-miR-9-3p, hsa-miR-138-5p, hsa-miR-339-5p, hsa-miR-574-5p, hsa-miR-589-5p, hsa-miR-320d, hsa-miR-1275, hsa-miR-629-5p, hsa-miR-29c-5p, hsa-miR-361-3p, hsa-miR-342-5p, hsa-miR-335-3p, hsa-miR-500a-3p, hsa-miR-671-5p, hsa-miR-1307-5p, hsa-miR-2110, hsa-miR-320c, hsa-miR-378i, hsa-miR-1-3p, hsa-miR-887-3p, hsa-miR-320a-3p, hsa-miR-147b-3p
AFF4	25	hsa-miR-335-5p, hsa-miR-148a-3p, hsa-miR-20b-5p, hsa-miR-7-5p, hsa-miR-9-3p, hsa-miR-138-5p, hsa-miR-361-5p, hsa-miR-574-5p, hsa-miR-769-5p, hsa-miR-188-5p, hsa-miR-320d, hsa-miR-1275, hsa-miR-629-5p, hsa-miR-29c-5p, hsa-miR-342-5p, hsa-miR-335-3p, hsa-miR-500a-3p, hsa-miR-671-5p, hsa-miR-320c, hsa-miR-1468-5p, hsa-miR-1-3p, hsa-miR-133a-3p, hsa-miR-887-3p, hsa-miR-320a-3p, hsa-miR-147b-3p
ANKRD17	25	hsa-miR-1-3p, hsa-miR-148a-3p, hsa-miR-20b-5p, hsa-miR-335-5p, hsa-miR-7-5p, hsa-miR-9-3p, hsa-miR-138-5p, hsa-miR-361-5p, hsa-miR-339-5p, hsa-miR-574-5p, hsa-miR-589-5p, hsa-miR-769-5p, hsa-miR-188-5p, hsa-miR-1275, hsa-miR-361-3p, hsa-miR-335-3p, hsa-miR-671-5p, hsa-miR-1307-3p, hsa-miR-2110, hsa-miR-320c, hsa-miR-3615, hsa-miR-133a-3p, hsa-miR-887-3p, hsa-miR-320a-3p, hsa-miR-147b-3p
CHD4	25	hsa-miR-361-3p, hsa-miR-148a-3p, hsa-miR-20b-5p, hsa-miR-335-5p, hsa-miR-7-5p, hsa-miR-138-5p, hsa-miR-361-5p, hsa-miR-339-5p, hsa-miR-574-5p, hsa-miR-589-5p, hsa-miR-769-5p, hsa-miR-320d, hsa-miR-629-5p, hsa-miR-342-5p, hsa-miR-335-3p, hsa-miR-500a-3p, hsa-miR-671-5p, hsa-miR-1307-3p, hsa-miR-2110, hsa-miR-320c, hsa-miR-1468-5p, hsa-miR-1-3p, hsa-miR-133a-3p, hsa-miR-887-3p, hsa-miR-320a-3p
CREBRF	25	hsa-miR-335-5p, hsa-miR-9-3p, hsa-miR-574-5p, hsa-miR-320d, hsa-miR-320c, hsa-miR-148a-3p, hsa-miR-20b-5p, hsa-miR-7-5p, hsa-miR-138-5p, hsa-miR-361-5p, hsa-miR-339-5p, hsa-miR-589-5p, hsa-miR-1275, hsa-miR-629-5p, hsa-miR-361-3p, hsa-miR-342-5p, hsa-miR-335-

		3p, hsa-miR-671-5p, hsa-miR-1307-5p, hsa-miR-3615, hsa-miR-874-3p, hsa-miR-1-3p, hsa-miR-133a-3p, hsa-miR-320a-3p, hsa-miR-147b-3p
EEF2	25	hsa-miR-148a-3p, hsa-miR-20b-5p, hsa-miR-335-5p, hsa-miR-7-5p, hsa-miR-138-5p, hsa-miR-361-5p, hsa-miR-574-5p, hsa-miR-589-5p, hsa-miR-769-5p, hsa-miR-188-5p, hsa-miR-320d, hsa-miR-361-3p, hsa-miR-342-5p, hsa-miR-335-3p, hsa-miR-500a-3p, hsa-miR-671-5p, hsa-miR-1307-3p, hsa-miR-2110, hsa-miR-320c, hsa-miR-3615, hsa-miR-378i, hsa-miR-1468-5p, hsa-miR-133a-3p, hsa-miR-320a-3p, hsa-miR-147b-3p
HECTD1	25	hsa-miR-335-5p, hsa-miR-148a-3p, hsa-miR-20b-5p, hsa-miR-7-5p, hsa-miR-9-3p, hsa-miR-138-5p, hsa-miR-361-5p, hsa-miR-339-5p, hsa-miR-574-5p, hsa-miR-589-5p, hsa-miR-320d, hsa-miR-1275, hsa-miR-629-5p, hsa-miR-29c-5p, hsa-miR-342-5p, hsa-miR-335-3p, hsa-miR-500a-3p, hsa-miR-1307-5p, hsa-miR-2110, hsa-miR-320c, hsa-miR-3615, hsa-miR-378i, hsa-miR-1-3p, hsa-miR-133a-3p, hsa-miR-320a-3p
HSP90AB1	25	hsa-miR-335-5p, hsa-miR-148a-3p, hsa-miR-20b-5p, hsa-miR-7-5p, hsa-miR-9-3p, hsa-miR-361-5p, hsa-miR-339-5p, hsa-miR-589-5p, hsa-miR-769-5p, hsa-miR-320d, hsa-miR-1275, hsa-miR-629-5p, hsa-miR-335-3p, hsa-miR-425-3p, hsa-miR-671-5p, hsa-miR-1307-5p, hsa-miR-3168, hsa-miR-2110, hsa-miR-320c, hsa-miR-3615, hsa-miR-378i, hsa-miR-874-3p, hsa-miR-1468-5p, hsa-miR-133a-3p, hsa-miR-320a-3p
LARP1	25	hsa-miR-148a-3p, hsa-miR-20b-5p, hsa-miR-335-5p, hsa-miR-7-5p, hsa-miR-9-3p, hsa-miR-138-5p, hsa-miR-361-5p, hsa-miR-339-5p, hsa-miR-574-5p, hsa-miR-589-5p, hsa-miR-769-5p, hsa-miR-320d, hsa-miR-1275, hsa-miR-342-5p, hsa-miR-335-3p, hsa-miR-425-3p, hsa-miR-500a-3p, hsa-miR-671-5p, hsa-miR-1307-3p, hsa-miR-2110, hsa-miR-320c, hsa-miR-378i, hsa-miR-1-3p, hsa-miR-320a-3p, hsa-miR-147b-3p
LCOR	25	hsa-miR-335-5p, hsa-miR-574-5p, hsa-miR-675-3p, hsa-miR-2110, hsa-miR-148a-3p, hsa-miR-20b-5p, hsa-miR-7-5p, hsa-miR-9-3p, hsa-miR-138-5p, hsa-miR-361-5p, hsa-miR-339-5p, hsa-miR-589-5p, hsa-miR-320d, hsa-miR-1275, hsa-miR-629-5p, hsa-miR-342-5p, hsa-miR-335-3p, hsa-miR-425-3p, hsa-miR-500a-3p, hsa-miR-671-5p, hsa-miR-320c, hsa-miR-378i, hsa-miR-874-3p, hsa-miR-1-3p, hsa-miR-320a-3p
MCL1	25	hsa-miR-320a-3p, hsa-miR-20b-5p, hsa-miR-320d, hsa-miR-320c, hsa-miR-133a-3p, hsa-miR-148a-3p, hsa-miR-335-5p, hsa-miR-7-5p, hsa-miR-9-3p, hsa-miR-138-5p, hsa-miR-361-5p, hsa-miR-574-5p, hsa-miR-769-5p, hsa-miR-1275, hsa-miR-629-5p, hsa-miR-29c-5p, hsa-miR-342-

		5p, hsa-miR-335-3p, hsa-miR-500a-3p, hsa-miR-671-5p, hsa-miR-1307-3p, hsa-miR-1307-5p, hsa-miR-2110, hsa-miR-1468-5p, hsa-miR-147b-3p
MED13	25	hsa-miR-148a-3p, hsa-miR-20b-5p, hsa-miR-335-5p, hsa-miR-7-5p, hsa-miR-9-3p, hsa-miR-138-5p, hsa-miR-361-5p, hsa-miR-339-5p, hsa-miR-574-5p, hsa-miR-589-5p, hsa-miR-769-5p, hsa-miR-1275, hsa-miR-361-3p, hsa-miR-342-5p, hsa-miR-335-3p, hsa-miR-671-5p, hsa-miR-2110, hsa-miR-320c, hsa-miR-378i, hsa-miR-874-3p, hsa-miR-1-3p, hsa-miR-133a-3p, hsa-miR-887-3p, hsa-miR-320a-3p, hsa-miR-190b-5p
MED13L	25	hsa-miR-148a-3p, hsa-miR-20b-5p, hsa-miR-335-5p, hsa-miR-7-5p, hsa-miR-138-5p, hsa-miR-361-5p, hsa-miR-339-5p, hsa-miR-574-5p, hsa-miR-589-5p, hsa-miR-769-5p, hsa-miR-1275, hsa-miR-629-5p, hsa-miR-29c-5p, hsa-miR-361-3p, hsa-miR-342-5p, hsa-miR-335-3p, hsa-miR-500a-3p, hsa-miR-671-5p, hsa-miR-2110, hsa-miR-320c, hsa-miR-3615, hsa-miR-378i, hsa-miR-887-3p, hsa-miR-320a-3p, hsa-miR-190b-5p
NOTCH2	25	hsa-miR-9-3p, hsa-miR-1-3p, hsa-miR-148a-3p, hsa-miR-20b-5p, hsa-miR-335-5p, hsa-miR-7-5p, hsa-miR-138-5p, hsa-miR-339-5p, hsa-miR-589-5p, hsa-miR-769-5p, hsa-miR-320d, hsa-miR-1275, hsa-miR-342-5p, hsa-miR-335-3p, hsa-miR-425-3p, hsa-miR-500a-3p, hsa-miR-551b-3p, hsa-miR-671-5p, hsa-miR-2110, hsa-miR-320c, hsa-miR-378i, hsa-miR-133a-3p, hsa-miR-887-3p, hsa-miR-320a-3p, hsa-miR-147b-3p
PDZD8	25	hsa-miR-335-5p, hsa-miR-148a-3p, hsa-miR-20b-5p, hsa-miR-7-5p, hsa-miR-138-5p, hsa-miR-361-5p, hsa-miR-339-5p, hsa-miR-574-5p, hsa-miR-589-5p, hsa-miR-769-5p, hsa-miR-320d, hsa-miR-629-5p, hsa-miR-29c-5p, hsa-miR-342-5p, hsa-miR-335-3p, hsa-miR-425-3p, hsa-miR-671-5p, hsa-miR-1307-5p, hsa-miR-320c, hsa-miR-1-3p, hsa-miR-133a-3p, hsa-miR-887-3p, hsa-miR-320a-3p, hsa-miR-147b-3p, hsa-miR-190b-5p
SNRNP200	25	hsa-miR-148a-3p, hsa-miR-20b-5p, hsa-miR-335-5p, hsa-miR-7-5p, hsa-miR-138-5p, hsa-miR-361-5p, hsa-miR-339-5p, hsa-miR-574-5p, hsa-miR-589-5p, hsa-miR-320d, hsa-miR-1275, hsa-miR-629-5p, hsa-miR-29c-5p, hsa-miR-342-5p, hsa-miR-335-3p, hsa-miR-425-3p, hsa-miR-500a-3p, hsa-miR-551b-3p, hsa-miR-671-5p, hsa-miR-3615, hsa-miR-378i, hsa-miR-1-3p, hsa-miR-133a-3p, hsa-miR-320a-3p, hsa-miR-147b-3p
SRCAP	25	hsa-miR-20b-5p, hsa-miR-9-3p, hsa-miR-320d, hsa-miR-2110, hsa-miR-320c, hsa-miR-148a-3p, hsa-miR-335-5p, hsa-miR-7-5p, hsa-miR-138-

		5p, hsa-miR-361-5p, hsa-miR-574-5p, hsa-miR-589-5p, hsa-miR-769-5p, hsa-miR-1275, hsa-miR-342-5p, hsa-miR-335-3p, hsa-miR-425-3p, hsa-miR-500a-3p, hsa-miR-671-5p, hsa-miR-1307-3p, hsa-miR-1307-5p, hsa-miR-378i, hsa-miR-133a-3p, hsa-miR-320a-3p, hsa-miR-147b-3p
TNRC6B	25	hsa-miR-148a-3p, hsa-miR-20b-5p, hsa-miR-7-5p, hsa-miR-335-5p, hsa-miR-138-5p, hsa-miR-361-5p, hsa-miR-339-5p, hsa-miR-574-5p, hsa-miR-769-5p, hsa-miR-320d, hsa-miR-1275, hsa-miR-629-5p, hsa-miR-361-3p, hsa-miR-342-5p, hsa-miR-335-3p, hsa-miR-500a-3p, hsa-miR-671-5p, hsa-miR-2110, hsa-miR-320c, hsa-miR-1468-5p, hsa-miR-1-3p, hsa-miR-887-3p, hsa-miR-320a-3p, hsa-miR-147b-3p, hsa-miR-190b-5p
ZNF207	25	hsa-miR-361-5p, hsa-miR-335-3p, hsa-miR-1307-3p, hsa-miR-1-3p, hsa-miR-148a-3p, hsa-miR-20b-5p, hsa-miR-335-5p, hsa-miR-7-5p, hsa-miR-9-3p, hsa-miR-138-5p, hsa-miR-339-5p, hsa-miR-589-5p, hsa-miR-320d, hsa-miR-1275, hsa-miR-629-5p, hsa-miR-342-5p, hsa-miR-500a-3p, hsa-miR-551b-3p, hsa-miR-671-5p, hsa-miR-2110, hsa-miR-320c, hsa-miR-378i, hsa-miR-133a-3p, hsa-miR-320a-3p, hsa-miR-147b-3p
ZNF281	25	hsa-miR-335-5p, hsa-miR-2110, hsa-miR-148a-3p, hsa-miR-20b-5p, hsa-miR-7-5p, hsa-miR-9-3p, hsa-miR-138-5p, hsa-miR-361-5p, hsa-miR-339-5p, hsa-miR-574-5p, hsa-miR-769-5p, hsa-miR-188-5p, hsa-miR-320d, hsa-miR-1275, hsa-miR-361-3p, hsa-miR-342-5p, hsa-miR-335-3p, hsa-miR-671-5p, hsa-miR-1307-3p, hsa-miR-320c, hsa-miR-874-3p, hsa-miR-1-3p, hsa-miR-320a-3p, hsa-miR-147b-3p, hsa-miR-190b-5p
ARID1A	24	hsa-miR-1-3p, hsa-miR-148a-3p, hsa-miR-20b-5p, hsa-miR-335-5p, hsa-miR-7-5p, hsa-miR-138-5p, hsa-miR-361-5p, hsa-miR-574-5p, hsa-miR-589-5p, hsa-miR-769-5p, hsa-miR-188-5p, hsa-miR-1275, hsa-miR-629-5p, hsa-miR-361-3p, hsa-miR-342-5p, hsa-miR-335-3p, hsa-miR-425-3p, hsa-miR-500a-3p, hsa-miR-671-5p, hsa-miR-2110, hsa-miR-3615, hsa-miR-378i, hsa-miR-133a-3p, hsa-miR-147b-3p
BAZ2A	24	hsa-miR-671-5p, hsa-miR-148a-3p, hsa-miR-20b-5p, hsa-miR-335-5p, hsa-miR-7-5p, hsa-miR-138-5p, hsa-miR-361-5p, hsa-miR-339-5p, hsa-miR-574-5p, hsa-miR-589-5p, hsa-miR-769-5p, hsa-miR-188-5p, hsa-miR-1275, hsa-miR-342-5p, hsa-miR-335-3p, hsa-miR-425-3p, hsa-miR-500a-3p, hsa-miR-1307-3p, hsa-miR-2110, hsa-miR-320c, hsa-miR-3615, hsa-miR-874-3p, hsa-miR-133a-3p, hsa-miR-320a-3p
CPSF6	24	hsa-miR-20b-5p, hsa-miR-9-3p, hsa-miR-138-5p, hsa-miR-361-5p, hsa-miR-339-5p, hsa-miR-574-5p, hsa-miR-589-5p, hsa-miR-188-5p, hsa-

		miR-320d, hsa-miR-1275, hsa-miR-629-5p, hsa-miR-361-3p, hsa-miR-342-5p, hsa-miR-335-3p, hsa-miR-425-3p, hsa-miR-500a-3p, hsa-miR-671-5p, hsa-miR-1307-5p, hsa-miR-320c, hsa-miR-3615, hsa-miR-378i, hsa-miR-1-3p, hsa-miR-887-3p, hsa-miR-320a-3p
EIF4G1	24	hsa-miR-1-3p, hsa-miR-148a-3p, hsa-miR-20b-5p, hsa-miR-335-5p, hsa-miR-7-5p, hsa-miR-138-5p, hsa-miR-361-5p, hsa-miR-339-5p, hsa-miR-574-5p, hsa-miR-589-5p, hsa-miR-769-5p, hsa-miR-188-5p, hsa-miR-320d, hsa-miR-361-3p, hsa-miR-342-5p, hsa-miR-335-3p, hsa-miR-500a-3p, hsa-miR-671-5p, hsa-miR-1307-3p, hsa-miR-2110, hsa-miR-3615, hsa-miR-378i, hsa-miR-320a-3p, hsa-miR-147b-3p
HIPK2	24	hsa-miR-148a-3p, hsa-miR-20b-5p, hsa-miR-335-5p, hsa-miR-7-5p, hsa-miR-138-5p, hsa-miR-361-5p, hsa-miR-339-5p, hsa-miR-574-5p, hsa-miR-589-5p, hsa-miR-769-5p, hsa-miR-320d, hsa-miR-629-5p, hsa-miR-335-3p, hsa-miR-425-3p, hsa-miR-500a-3p, hsa-miR-671-5p, hsa-miR-1307-3p, hsa-miR-2110, hsa-miR-320c, hsa-miR-378i, hsa-miR-874-3p, hsa-miR-133a-3p, hsa-miR-320a-3p, hsa-miR-147b-3p
KMT2C	24	hsa-miR-335-5p, hsa-miR-148a-3p, hsa-miR-20b-5p, hsa-miR-7-5p, hsa-miR-9-3p, hsa-miR-138-5p, hsa-miR-361-5p, hsa-miR-589-5p, hsa-miR-769-5p, hsa-miR-188-5p, hsa-miR-1275, hsa-miR-629-5p, hsa-miR-361-3p, hsa-miR-342-5p, hsa-miR-335-3p, hsa-miR-425-3p, hsa-miR-500a-3p, hsa-miR-671-5p, hsa-miR-3168, hsa-miR-2110, hsa-miR-320c, hsa-miR-1468-5p, hsa-miR-1-3p, hsa-miR-320a-3p
MACF1	24	hsa-miR-335-5p, hsa-miR-148a-3p, hsa-miR-20b-5p, hsa-miR-7-5p, hsa-miR-138-5p, hsa-miR-361-5p, hsa-miR-339-5p, hsa-miR-574-5p, hsa-miR-589-5p, hsa-miR-769-5p, hsa-miR-320d, hsa-miR-342-5p, hsa-miR-335-3p, hsa-miR-425-3p, hsa-miR-500a-3p, hsa-miR-671-5p, hsa-miR-675-3p, hsa-miR-2110, hsa-miR-320c, hsa-miR-1-3p, hsa-miR-133a-3p, hsa-miR-887-3p, hsa-miR-320a-3p, hsa-miR-147b-3p
MKNK2	24	hsa-miR-20b-5p, hsa-miR-335-5p, hsa-miR-361-3p, hsa-miR-148a-3p, hsa-miR-7-5p, hsa-miR-9-3p, hsa-miR-138-5p, hsa-miR-339-5p, hsa-miR-574-5p, hsa-miR-589-5p, hsa-miR-769-5p, hsa-miR-320d, hsa-miR-1275, hsa-miR-629-5p, hsa-miR-342-5p, hsa-miR-335-3p, hsa-miR-425-3p, hsa-miR-500a-3p, hsa-miR-671-5p, hsa-miR-1307-5p, hsa-miR-2110, hsa-miR-1-3p, hsa-miR-320a-3p, hsa-miR-147b-3p
MYH9	24	hsa-miR-20b-5p, hsa-miR-671-5p, hsa-miR-148a-3p, hsa-miR-7-5p, hsa-miR-9-3p, hsa-miR-138-5p, hsa-miR-361-5p, hsa-miR-339-5p, hsa-miR-574-5p, hsa-miR-589-5p, hsa-miR-769-5p, hsa-miR-320d, hsa-miR-361-

		3p, hsa-miR-335-3p, hsa-miR-500a-3p, hsa-miR-1307-5p, hsa-miR-320c, hsa-miR-3615, hsa-miR-378i, hsa-miR-874-3p, hsa-miR-1-3p, hsa-miR-133a-3p, hsa-miR-320a-3p, hsa-miR-147b-3p
NFAT5	24	hsa-miR-20b-5p, hsa-miR-335-5p, hsa-miR-1-3p, hsa-miR-148a-3p, hsa-miR-7-5p, hsa-miR-138-5p, hsa-miR-361-5p, hsa-miR-339-5p, hsa-miR-589-5p, hsa-miR-769-5p, hsa-miR-1275, hsa-miR-629-5p, hsa-miR-361-3p, hsa-miR-342-5p, hsa-miR-335-3p, hsa-miR-500a-3p, hsa-miR-671-5p, hsa-miR-2110, hsa-miR-320c, hsa-miR-3615, hsa-miR-378i, hsa-miR-887-3p, hsa-miR-320a-3p, hsa-miR-147b-3p
PRKDC	24	hsa-miR-148a-3p, hsa-miR-20b-5p, hsa-miR-335-5p, hsa-miR-7-5p, hsa-miR-138-5p, hsa-miR-361-5p, hsa-miR-339-5p, hsa-miR-574-5p, hsa-miR-589-5p, hsa-miR-1275, hsa-miR-29c-5p, hsa-miR-342-5p, hsa-miR-335-3p, hsa-miR-425-3p, hsa-miR-500a-3p, hsa-miR-1307-3p, hsa-miR-1307-5p, hsa-miR-320c, hsa-miR-378i, hsa-miR-1468-5p, hsa-miR-1-3p, hsa-miR-133a-3p, hsa-miR-320a-3p, hsa-miR-147b-3p
SBNO1	24	hsa-miR-188-5p, hsa-miR-4516, hsa-miR-148a-3p, hsa-miR-20b-5p, hsa-miR-7-5p, hsa-miR-9-3p, hsa-miR-361-5p, hsa-miR-339-5p, hsa-miR-589-5p, hsa-miR-769-5p, hsa-miR-320d, hsa-miR-1275, hsa-miR-29c-5p, hsa-miR-342-5p, hsa-miR-335-3p, hsa-miR-500a-3p, hsa-miR-671-5p, hsa-miR-1307-3p, hsa-miR-320c, hsa-miR-378i, hsa-miR-1-3p, hsa-miR-887-3p, hsa-miR-320a-3p, hsa-miR-190b-5p
SLC7A5	24	hsa-miR-7-5p, hsa-miR-574-5p, hsa-miR-769-5p, hsa-miR-671-5p, hsa-miR-4516, hsa-miR-148a-3p, hsa-miR-20b-5p, hsa-miR-335-5p, hsa-miR-138-5p, hsa-miR-361-5p, hsa-miR-320d, hsa-miR-1275, hsa-miR-342-5p, hsa-miR-335-3p, hsa-miR-425-3p, hsa-miR-500a-3p, hsa-miR-1307-3p, hsa-miR-1307-5p, hsa-miR-2110, hsa-miR-320c, hsa-miR-378i, hsa-miR-133a-3p, hsa-miR-320a-3p, hsa-miR-147b-3p
SPTBN1	24	hsa-miR-148a-3p, hsa-miR-20b-5p, hsa-miR-7-5p, hsa-miR-138-5p, hsa-miR-361-5p, hsa-miR-339-5p, hsa-miR-574-5p, hsa-miR-589-5p, hsa-miR-769-5p, hsa-miR-320d, hsa-miR-629-5p, hsa-miR-361-3p, hsa-miR-342-5p, hsa-miR-335-3p, hsa-miR-500a-3p, hsa-miR-671-5p, hsa-miR-2110, hsa-miR-320c, hsa-miR-3615, hsa-miR-378i, hsa-miR-1-3p, hsa-miR-887-3p, hsa-miR-320a-3p, hsa-miR-147b-3p
UBAP2L	24	hsa-miR-148a-3p, hsa-miR-20b-5p, hsa-miR-335-5p, hsa-miR-7-5p, hsa-miR-138-5p, hsa-miR-361-5p, hsa-miR-339-5p, hsa-miR-574-5p, hsa-miR-589-5p, hsa-miR-769-5p, hsa-miR-188-5p, hsa-miR-1275, hsa-miR-629-5p, hsa-miR-361-3p, hsa-miR-342-5p, hsa-miR-335-3p, hsa-miR-

		425-3p, hsa-miR-500a-3p, hsa-miR-551b-3p, hsa-miR-671-5p, hsa-miR-320c, hsa-miR-1-3p, hsa-miR-320a-3p, hsa-miR-147b-3p
WNK1	24	hsa-miR-148a-3p, hsa-miR-20b-5p, hsa-miR-335-5p, hsa-miR-7-5p, hsa-miR-138-5p, hsa-miR-361-5p, hsa-miR-339-5p, hsa-miR-589-5p, hsa-miR-769-5p, hsa-miR-320d, hsa-miR-1275, hsa-miR-342-5p, hsa-miR-335-3p, hsa-miR-500a-3p, hsa-miR-551b-3p, hsa-miR-671-5p, hsa-miR-2110, hsa-miR-320c, hsa-miR-378i, hsa-miR-1-3p, hsa-miR-887-3p, hsa-miR-320a-3p, hsa-miR-147b-3p, hsa-miR-190b-5p
YTHDF3	24	hsa-miR-148a-3p, hsa-miR-20b-5p, hsa-miR-335-5p, hsa-miR-7-5p, hsa-miR-138-5p, hsa-miR-339-5p, hsa-miR-574-5p, hsa-miR-589-5p, hsa-miR-769-5p, hsa-miR-320d, hsa-miR-629-5p, hsa-miR-361-3p, hsa-miR-342-5p, hsa-miR-335-3p, hsa-miR-500a-3p, hsa-miR-1307-3p, hsa-miR-3168, hsa-miR-675-3p, hsa-miR-320c, hsa-miR-378i, hsa-miR-874-3p, hsa-miR-1-3p, hsa-miR-320a-3p, hsa-miR-190b-5p
ADGRL1	23	hsa-miR-148a-3p, hsa-miR-20b-5p, hsa-miR-335-5p, hsa-miR-7-5p, hsa-miR-138-5p, hsa-miR-339-5p, hsa-miR-574-5p, hsa-miR-769-5p, hsa-miR-320d, hsa-miR-361-3p, hsa-miR-342-5p, hsa-miR-500a-3p, hsa-miR-551b-3p, hsa-miR-671-5p, hsa-miR-1307-3p, hsa-miR-1307-5p, hsa-miR-2110, hsa-miR-320c, hsa-miR-378i, hsa-miR-874-3p, hsa-miR-1-3p, hsa-miR-320a-3p, hsa-miR-147b-3p
ATP1A1	23	hsa-miR-148a-3p, hsa-miR-20b-5p, hsa-miR-335-5p, hsa-miR-7-5p, hsa-miR-361-5p, hsa-miR-574-5p, hsa-miR-589-5p, hsa-miR-769-5p, hsa-miR-629-5p, hsa-miR-29c-5p, hsa-miR-335-3p, hsa-miR-425-3p, hsa-miR-500a-3p, hsa-miR-671-5p, hsa-miR-1307-3p, hsa-miR-2110, hsa-miR-320c, hsa-miR-3615, hsa-miR-874-3p, hsa-miR-1-3p, hsa-miR-133a-3p, hsa-miR-320a-3p, hsa-miR-147b-3p
ATP2A2	23	hsa-miR-671-5p, hsa-miR-874-3p, hsa-miR-148a-3p, hsa-miR-20b-5p, hsa-miR-335-5p, hsa-miR-7-5p, hsa-miR-361-5p, hsa-miR-574-5p, hsa-miR-769-5p, hsa-miR-320d, hsa-miR-361-3p, hsa-miR-335-3p, hsa-miR-425-3p, hsa-miR-500a-3p, hsa-miR-1307-3p, hsa-miR-1307-5p, hsa-miR-3168, hsa-miR-2110, hsa-miR-320c, hsa-miR-378i, hsa-miR-1468-5p, hsa-miR-320a-3p, hsa-miR-147b-3p
BAZ1B	23	hsa-miR-148a-3p, hsa-miR-20b-5p, hsa-miR-335-5p, hsa-miR-7-5p, hsa-miR-138-5p, hsa-miR-361-5p, hsa-miR-339-5p, hsa-miR-574-5p, hsa-miR-769-5p, hsa-miR-629-5p, hsa-miR-342-5p, hsa-miR-335-3p, hsa-miR-500a-3p, hsa-miR-671-5p, hsa-miR-1307-3p, hsa-miR-2110, hsa-

		miR-320c, hsa-miR-3615, hsa-miR-378i, hsa-miR-1-3p, hsa-miR-133a-3p, hsa-miR-320a-3p, hsa-miR-147b-3p
BCL9L	23	hsa-miR-7-5p, hsa-miR-4516, hsa-miR-148a-3p, hsa-miR-20b-5p, hsa-miR-138-5p, hsa-miR-361-5p, hsa-miR-339-5p, hsa-miR-769-5p, hsa-miR-29c-5p, hsa-miR-361-3p, hsa-miR-335-3p, hsa-miR-425-3p, hsa-miR-500a-3p, hsa-miR-671-5p, hsa-miR-1307-3p, hsa-miR-1307-5p, hsa-miR-2110, hsa-miR-3615, hsa-miR-378i, hsa-miR-1-3p, hsa-miR-887-3p, hsa-miR-320a-3p, hsa-miR-147b-3p
BIRC6	23	hsa-miR-148a-3p, hsa-miR-20b-5p, hsa-miR-335-5p, hsa-miR-7-5p, hsa-miR-138-5p, hsa-miR-361-5p, hsa-miR-574-5p, hsa-miR-589-5p, hsa-miR-769-5p, hsa-miR-188-5p, hsa-miR-320d, hsa-miR-29c-5p, hsa-miR-342-5p, hsa-miR-335-3p, hsa-miR-425-3p, hsa-miR-500a-3p, hsa-miR-551b-3p, hsa-miR-671-5p, hsa-miR-2110, hsa-miR-320c, hsa-miR-378i, hsa-miR-1-3p, hsa-miR-320a-3p
BTBD7	23	hsa-miR-20b-5p, hsa-miR-887-3p, hsa-miR-148a-3p, hsa-miR-335-5p, hsa-miR-7-5p, hsa-miR-138-5p, hsa-miR-339-5p, hsa-miR-574-5p, hsa-miR-589-5p, hsa-miR-769-5p, hsa-miR-320d, hsa-miR-629-5p, hsa-miR-342-5p, hsa-miR-335-3p, hsa-miR-425-3p, hsa-miR-500a-3p, hsa-miR-671-5p, hsa-miR-2110, hsa-miR-320c, hsa-miR-378i, hsa-miR-1-3p, hsa-miR-320a-3p, hsa-miR-147b-3p
CBX5	23	hsa-miR-20b-5p, hsa-miR-339-5p, hsa-miR-2110, hsa-miR-1-3p, hsa-miR-148a-3p, hsa-miR-335-5p, hsa-miR-7-5p, hsa-miR-138-5p, hsa-miR-361-5p, hsa-miR-589-5p, hsa-miR-769-5p, hsa-miR-320d, hsa-miR-361-3p, hsa-miR-342-5p, hsa-miR-335-3p, hsa-miR-425-3p, hsa-miR-500a-3p, hsa-miR-671-5p, hsa-miR-320c, hsa-miR-1468-5p, hsa-miR-320a-3p, hsa-miR-147b-3p, hsa-miR-190b-5p
CPD	23	hsa-miR-148a-3p, hsa-miR-20b-5p, hsa-miR-335-5p, hsa-miR-7-5p, hsa-miR-9-3p, hsa-miR-138-5p, hsa-miR-361-5p, hsa-miR-574-5p, hsa-miR-589-5p, hsa-miR-769-5p, hsa-miR-320d, hsa-miR-629-5p, hsa-miR-361-3p, hsa-miR-335-3p, hsa-miR-500a-3p, hsa-miR-671-5p, hsa-miR-3168, hsa-miR-320c, hsa-miR-3615, hsa-miR-1-3p, hsa-miR-133a-3p, hsa-miR-320a-3p, hsa-miR-147b-3p
DHX15	23	hsa-miR-1-3p, hsa-miR-148a-3p, hsa-miR-20b-5p, hsa-miR-335-5p, hsa-miR-7-5p, hsa-miR-361-5p, hsa-miR-339-5p, hsa-miR-574-5p, hsa-miR-769-5p, hsa-miR-320d, hsa-miR-29c-5p, hsa-miR-342-5p, hsa-miR-335-3p, hsa-miR-425-3p, hsa-miR-500a-3p, hsa-miR-671-5p, hsa-miR-1307-

		5p, hsa-miR-320c, hsa-miR-3615, hsa-miR-874-3p, hsa-miR-887-3p, hsa-miR-320a-3p, hsa-miR-147b-3p
DST	23	hsa-miR-335-5p, hsa-miR-500a-3p, hsa-miR-148a-3p, hsa-miR-20b-5p, hsa-miR-7-5p, hsa-miR-138-5p, hsa-miR-361-5p, hsa-miR-339-5p, hsa-miR-574-5p, hsa-miR-589-5p, hsa-miR-769-5p, hsa-miR-629-5p, hsa-miR-29c-5p, hsa-miR-361-3p, hsa-miR-342-5p, hsa-miR-335-3p, hsa-miR-671-5p, hsa-miR-320c, hsa-miR-1468-5p, hsa-miR-1-3p, hsa-miR-887-3p, hsa-miR-320a-3p, hsa-miR-147b-3p
EP300	23	hsa-miR-574-5p, hsa-miR-148a-3p, hsa-miR-20b-5p, hsa-miR-335-5p, hsa-miR-7-5p, hsa-miR-138-5p, hsa-miR-361-5p, hsa-miR-339-5p, hsa-miR-589-5p, hsa-miR-1275, hsa-miR-629-5p, hsa-miR-335-3p, hsa-miR-425-3p, hsa-miR-500a-3p, hsa-miR-671-5p, hsa-miR-1307-5p, hsa-miR-2110, hsa-miR-320c, hsa-miR-1468-5p, hsa-miR-1-3p, hsa-miR-320a-3p, hsa-miR-147b-3p, hsa-miR-190b-5p
FN1	23	hsa-miR-1-3p, hsa-miR-148a-3p, hsa-miR-20b-5p, hsa-miR-335-5p, hsa-miR-7-5p, hsa-miR-9-3p, hsa-miR-138-5p, hsa-miR-361-5p, hsa-miR-574-5p, hsa-miR-769-5p, hsa-miR-320d, hsa-miR-335-3p, hsa-miR-425-3p, hsa-miR-500a-3p, hsa-miR-671-5p, hsa-miR-1307-3p, hsa-miR-2110, hsa-miR-320c, hsa-miR-3615, hsa-miR-378i, hsa-miR-133a-3p, hsa-miR-320a-3p, hsa-miR-147b-3p
HIPK3	23	hsa-miR-20b-5p, hsa-miR-335-5p, hsa-miR-378i, hsa-miR-1-3p, hsa-miR-148a-3p, hsa-miR-7-5p, hsa-miR-9-3p, hsa-miR-138-5p, hsa-miR-361-5p, hsa-miR-574-5p, hsa-miR-589-5p, hsa-miR-320d, hsa-miR-629-5p, hsa-miR-335-3p, hsa-miR-500a-3p, hsa-miR-671-5p, hsa-miR-2110, hsa-miR-320c, hsa-miR-874-3p, hsa-miR-133a-3p, hsa-miR-320a-3p, hsa-miR-147b-3p, hsa-miR-190b-5p
IGF1R	23	hsa-miR-335-5p, hsa-miR-7-5p, hsa-miR-138-5p, hsa-miR-1275, hsa-miR-675-3p, hsa-miR-133a-3p, hsa-miR-148a-3p, hsa-miR-20b-5p, hsa-miR-9-3p, hsa-miR-361-5p, hsa-miR-574-5p, hsa-miR-589-5p, hsa-miR-769-5p, hsa-miR-629-5p, hsa-miR-335-3p, hsa-miR-500a-3p, hsa-miR-671-5p, hsa-miR-2110, hsa-miR-320c, hsa-miR-1-3p, hsa-miR-887-3p, hsa-miR-320a-3p, hsa-miR-147b-3p
KHSRP	23	hsa-miR-335-3p, hsa-miR-3615, hsa-miR-20b-5p, hsa-miR-335-5p, hsa-miR-7-5p, hsa-miR-138-5p, hsa-miR-339-5p, hsa-miR-574-5p, hsa-miR-589-5p, hsa-miR-320d, hsa-miR-629-5p, hsa-miR-361-3p, hsa-miR-342-5p, hsa-miR-500a-3p, hsa-miR-671-5p, hsa-miR-1307-3p, hsa-miR-

		2110, hsa-miR-320c, hsa-miR-378i, hsa-miR-1468-5p, hsa-miR-1-3p, hsa-miR-133a-3p, hsa-miR-320a-3p
KPNA6	23	hsa-miR-20b-5p, hsa-miR-7-5p, hsa-miR-4516, hsa-miR-133a-3p, hsa-miR-148a-3p, hsa-miR-335-5p, hsa-miR-138-5p, hsa-miR-361-5p, hsa-miR-339-5p, hsa-miR-589-5p, hsa-miR-769-5p, hsa-miR-629-5p, hsa-miR-335-3p, hsa-miR-425-3p, hsa-miR-500a-3p, hsa-miR-1307-5p, hsa-miR-2110, hsa-miR-320c, hsa-miR-378i, hsa-miR-874-3p, hsa-miR-1-3p, hsa-miR-320a-3p, hsa-miR-147b-3p
NSD1	23	hsa-miR-7-5p, hsa-miR-148a-3p, hsa-miR-20b-5p, hsa-miR-335-5p, hsa-miR-138-5p, hsa-miR-361-5p, hsa-miR-339-5p, hsa-miR-574-5p, hsa-miR-188-5p, hsa-miR-320d, hsa-miR-1275, hsa-miR-629-5p, hsa-miR-335-3p, hsa-miR-425-3p, hsa-miR-500a-3p, hsa-miR-671-5p, hsa-miR-1307-3p, hsa-miR-2110, hsa-miR-320c, hsa-miR-378i, hsa-miR-1-3p, hsa-miR-887-3p, hsa-miR-320a-3p
PARP1	23	hsa-miR-335-5p, hsa-miR-7-5p, hsa-miR-339-5p, hsa-miR-874-3p, hsa-miR-148a-3p, hsa-miR-20b-5p, hsa-miR-9-3p, hsa-miR-138-5p, hsa-miR-361-5p, hsa-miR-574-5p, hsa-miR-589-5p, hsa-miR-320d, hsa-miR-1275, hsa-miR-29c-5p, hsa-miR-361-3p, hsa-miR-342-5p, hsa-miR-335-3p, hsa-miR-500a-3p, hsa-miR-671-5p, hsa-miR-2110, hsa-miR-320c, hsa-miR-378i, hsa-miR-320a-3p
POGZ	23	hsa-miR-2110, hsa-miR-148a-3p, hsa-miR-20b-5p, hsa-miR-7-5p, hsa-miR-138-5p, hsa-miR-361-5p, hsa-miR-589-5p, hsa-miR-769-5p, hsa-miR-1275, hsa-miR-629-5p, hsa-miR-29c-5p, hsa-miR-342-5p, hsa-miR-335-3p, hsa-miR-425-3p, hsa-miR-500a-3p, hsa-miR-671-5p, hsa-miR-1307-3p, hsa-miR-3168, hsa-miR-320c, hsa-miR-1-3p, hsa-miR-887-3p, hsa-miR-320a-3p, hsa-miR-147b-3p
PURB	23	hsa-miR-20b-5p, hsa-miR-7-5p, hsa-miR-148a-3p, hsa-miR-138-5p, hsa-miR-361-5p, hsa-miR-339-5p, hsa-miR-589-5p, hsa-miR-769-5p, hsa-miR-188-5p, hsa-miR-1275, hsa-miR-361-3p, hsa-miR-342-5p, hsa-miR-335-3p, hsa-miR-425-3p, hsa-miR-500a-3p, hsa-miR-671-5p, hsa-miR-2110, hsa-miR-320c, hsa-miR-378i, hsa-miR-1-3p, hsa-miR-133a-3p, hsa-miR-320a-3p, hsa-miR-147b-3p
SON	23	hsa-miR-20b-5p, hsa-miR-361-5p, hsa-miR-574-5p, hsa-miR-148a-3p, hsa-miR-335-5p, hsa-miR-7-5p, hsa-miR-138-5p, hsa-miR-339-5p, hsa-miR-769-5p, hsa-miR-629-5p, hsa-miR-335-3p, hsa-miR-425-3p, hsa-miR-500a-3p, hsa-miR-671-5p, hsa-miR-1307-5p, hsa-miR-2110, hsa-

		miR-320c, hsa-miR-3615, hsa-miR-378i, hsa-miR-874-3p, hsa-miR-1-3p, hsa-miR-320a-3p, hsa-miR-147b-3p
SP1	23	hsa-miR-335-5p, hsa-miR-7-5p, hsa-miR-2110, hsa-miR-1-3p, hsa-miR-133a-3p, hsa-miR-148a-3p, hsa-miR-20b-5p, hsa-miR-138-5p, hsa-miR-361-5p, hsa-miR-339-5p, hsa-miR-574-5p, hsa-miR-589-5p, hsa-miR-769-5p, hsa-miR-1275, hsa-miR-629-5p, hsa-miR-342-5p, hsa-miR-335-3p, hsa-miR-500a-3p, hsa-miR-671-5p, hsa-miR-320c, hsa-miR-378i, hsa-miR-320a-3p, hsa-miR-147b-3p
TRRAP	23	hsa-miR-335-5p, hsa-miR-148a-3p, hsa-miR-20b-5p, hsa-miR-7-5p, hsa-miR-138-5p, hsa-miR-361-5p, hsa-miR-339-5p, hsa-miR-574-5p, hsa-miR-589-5p, hsa-miR-769-5p, hsa-miR-320d, hsa-miR-361-3p, hsa-miR-342-5p, hsa-miR-335-3p, hsa-miR-500a-3p, hsa-miR-671-5p, hsa-miR-1307-5p, hsa-miR-2110, hsa-miR-320c, hsa-miR-378i, hsa-miR-1-3p, hsa-miR-320a-3p, hsa-miR-147b-3p
VPS13D	23	hsa-miR-7-5p, hsa-miR-9-3p, hsa-miR-320d, hsa-miR-342-5p, hsa-miR-320c, hsa-miR-148a-3p, hsa-miR-20b-5p, hsa-miR-335-5p, hsa-miR-138-5p, hsa-miR-361-5p, hsa-miR-589-5p, hsa-miR-769-5p, hsa-miR-629-5p, hsa-miR-29c-5p, hsa-miR-335-3p, hsa-miR-425-3p, hsa-miR-671-5p, hsa-miR-1307-3p, hsa-miR-2110, hsa-miR-1-3p, hsa-miR-133a-3p, hsa-miR-320a-3p, hsa-miR-147b-3p
WDR26	23	hsa-miR-148a-3p, hsa-miR-20b-5p, hsa-miR-7-5p, hsa-miR-9-3p, hsa-miR-138-5p, hsa-miR-339-5p, hsa-miR-574-5p, hsa-miR-769-5p, hsa-miR-188-5p, hsa-miR-361-3p, hsa-miR-342-5p, hsa-miR-335-3p, hsa-miR-425-3p, hsa-miR-500a-3p, hsa-miR-671-5p, hsa-miR-2110, hsa-miR-320c, hsa-miR-3615, hsa-miR-378i, hsa-miR-1468-5p, hsa-miR-1-3p, hsa-miR-320a-3p, hsa-miR-190b-5p
ZBTB20	23	hsa-miR-335-5p, hsa-miR-574-5p, hsa-miR-361-3p, hsa-miR-2110, hsa-miR-1-3p, hsa-miR-148a-3p, hsa-miR-20b-5p, hsa-miR-7-5p, hsa-miR-9-3p, hsa-miR-138-5p, hsa-miR-361-5p, hsa-miR-769-5p, hsa-miR-188-5p, hsa-miR-320d, hsa-miR-1275, hsa-miR-629-5p, hsa-miR-342-5p, hsa-miR-500a-3p, hsa-miR-671-5p, hsa-miR-1307-3p, hsa-miR-320c, hsa-miR-320a-3p, hsa-miR-190b-5p
ZMIZ1	23	hsa-miR-335-5p, hsa-miR-148a-3p, hsa-miR-20b-5p, hsa-miR-7-5p, hsa-miR-9-3p, hsa-miR-138-5p, hsa-miR-361-5p, hsa-miR-339-5p, hsa-miR-589-5p, hsa-miR-769-5p, hsa-miR-1275, hsa-miR-629-5p, hsa-miR-342-5p, hsa-miR-335-3p, hsa-miR-425-3p, hsa-miR-500a-3p, hsa-miR-671-

		5p, hsa-miR-1307-5p, hsa-miR-2110, hsa-miR-378i, hsa-miR-133a-3p, hsa-miR-320a-3p, hsa-miR-147b-3p
ZZZ3	23	hsa-miR-148a-3p, hsa-miR-20b-5p, hsa-miR-335-5p, hsa-miR-7-5p, hsa-miR-138-5p, hsa-miR-361-5p, hsa-miR-339-5p, hsa-miR-574-5p, hsa-miR-589-5p, hsa-miR-769-5p, hsa-miR-188-5p, hsa-miR-1275, hsa-miR-361-3p, hsa-miR-342-5p, hsa-miR-671-5p, hsa-miR-2110, hsa-miR-4516, hsa-miR-378i, hsa-miR-4497, hsa-miR-1-3p, hsa-miR-335-3p, hsa-miR-133a-3p, hsa-miR-147b-3p
ANKRD11	22	hsa-miR-335-5p, hsa-miR-148a-3p, hsa-miR-20b-5p, hsa-miR-7-5p, hsa-miR-138-5p, hsa-miR-361-5p, hsa-miR-339-5p, hsa-miR-589-5p, hsa-miR-320d, hsa-miR-629-5p, hsa-miR-361-3p, hsa-miR-342-5p, hsa-miR-335-3p, hsa-miR-500a-3p, hsa-miR-671-5p, hsa-miR-1307-3p, hsa-miR-1307-5p, hsa-miR-320c, hsa-miR-1-3p, hsa-miR-133a-3p, hsa-miR-320a-3p, hsa-miR-147b-3p
AP1G1	22	hsa-miR-20b-5p, hsa-miR-148a-3p, hsa-miR-335-5p, hsa-miR-7-5p, hsa-miR-9-3p, hsa-miR-138-5p, hsa-miR-361-5p, hsa-miR-574-5p, hsa-miR-188-5p, hsa-miR-320d, hsa-miR-1275, hsa-miR-29c-5p, hsa-miR-342-5p, hsa-miR-335-3p, hsa-miR-500a-3p, hsa-miR-671-5p, hsa-miR-1307-3p, hsa-miR-2110, hsa-miR-320c, hsa-miR-1-3p, hsa-miR-320a-3p, hsa-miR-147b-3p
CAND1	22	hsa-miR-1-3p, hsa-miR-148a-3p, hsa-miR-20b-5p, hsa-miR-335-5p, hsa-miR-7-5p, hsa-miR-361-5p, hsa-miR-574-5p, hsa-miR-769-5p, hsa-miR-188-5p, hsa-miR-1275, hsa-miR-629-5p, hsa-miR-342-5p, hsa-miR-335-3p, hsa-miR-425-3p, hsa-miR-671-5p, hsa-miR-2110, hsa-miR-320c, hsa-miR-378i, hsa-miR-133a-3p, hsa-miR-887-3p, hsa-miR-320a-3p, hsa-miR-147b-3p
CANX	22	hsa-miR-7-5p, hsa-miR-4516, hsa-miR-148a-3p, hsa-miR-20b-5p, hsa-miR-138-5p, hsa-miR-361-5p, hsa-miR-574-5p, hsa-miR-589-5p, hsa-miR-769-5p, hsa-miR-1275, hsa-miR-335-3p, hsa-miR-671-5p, hsa-miR-1307-3p, hsa-miR-1307-5p, hsa-miR-2110, hsa-miR-320c, hsa-miR-3615, hsa-miR-378i, hsa-miR-1-3p, hsa-miR-887-3p, hsa-miR-320a-3p, hsa-miR-147b-3p
CCND2	22	hsa-miR-335-5p, hsa-miR-4516, hsa-miR-148a-3p, hsa-miR-20b-5p, hsa-miR-7-5p, hsa-miR-361-5p, hsa-miR-339-5p, hsa-miR-589-5p, hsa-miR-769-5p, hsa-miR-188-5p, hsa-miR-320d, hsa-miR-1275, hsa-miR-29c-5p, hsa-miR-342-5p, hsa-miR-671-5p, hsa-miR-2110, hsa-miR-320c,

		hsa-miR-874-3p, hsa-miR-1-3p, hsa-miR-133a-3p, hsa-miR-887-3p, hsa-miR-320a-3p
CHD9	22	hsa-miR-20b-5p, hsa-miR-7-5p, hsa-miR-148a-3p, hsa-miR-335-5p, hsa-miR-9-3p, hsa-miR-138-5p, hsa-miR-361-5p, hsa-miR-574-5p, hsa-miR-320d, hsa-miR-1275, hsa-miR-629-5p, hsa-miR-342-5p, hsa-miR-335-3p, hsa-miR-425-3p, hsa-miR-500a-3p, hsa-miR-671-5p, hsa-miR-2110, hsa-miR-320c, hsa-miR-378i, hsa-miR-1-3p, hsa-miR-887-3p, hsa-miR-320a-3p
CNOT1	22	hsa-miR-148a-3p, hsa-miR-20b-5p, hsa-miR-335-5p, hsa-miR-7-5p, hsa-miR-138-5p, hsa-miR-361-5p, hsa-miR-574-5p, hsa-miR-589-5p, hsa-miR-769-5p, hsa-miR-1275, hsa-miR-629-5p, hsa-miR-342-5p, hsa-miR-335-3p, hsa-miR-671-5p, hsa-miR-2110, hsa-miR-320c, hsa-miR-1-3p, hsa-miR-133a-3p, hsa-miR-887-3p, hsa-miR-320a-3p, hsa-miR-147b-3p, hsa-miR-190b-5p
COPS2	22	hsa-miR-148a-3p, hsa-miR-20b-5p, hsa-miR-335-5p, hsa-miR-7-5p, hsa-miR-9-3p, hsa-miR-339-5p, hsa-miR-574-5p, hsa-miR-589-5p, hsa-miR-769-5p, hsa-miR-188-5p, hsa-miR-320d, hsa-miR-1275, hsa-miR-342-5p, hsa-miR-335-3p, hsa-miR-500a-3p, hsa-miR-320c, hsa-miR-3615, hsa-miR-1468-5p, hsa-miR-1-3p, hsa-miR-133a-3p, hsa-miR-320a-3p, hsa-miR-147b-3p
CSNK1A1	22	hsa-miR-20b-5p, hsa-miR-148a-3p, hsa-miR-335-5p, hsa-miR-7-5p, hsa-miR-138-5p, hsa-miR-361-5p, hsa-miR-339-5p, hsa-miR-574-5p, hsa-miR-769-5p, hsa-miR-188-5p, hsa-miR-629-5p, hsa-miR-361-3p, hsa-miR-335-3p, hsa-miR-425-3p, hsa-miR-671-5p, hsa-miR-2110, hsa-miR-320c, hsa-miR-874-3p, hsa-miR-1-3p, hsa-miR-133a-3p, hsa-miR-887-3p, hsa-miR-320a-3p
FOXJ3	22	hsa-miR-20b-5p, hsa-miR-335-3p, hsa-miR-148a-3p, hsa-miR-335-5p, hsa-miR-7-5p, hsa-miR-9-3p, hsa-miR-138-5p, hsa-miR-361-5p, hsa-miR-339-5p, hsa-miR-574-5p, hsa-miR-589-5p, hsa-miR-769-5p, hsa-miR-1275, hsa-miR-29c-5p, hsa-miR-342-5p, hsa-miR-500a-3p, hsa-miR-671-5p, hsa-miR-320c, hsa-miR-1-3p, hsa-miR-887-3p, hsa-miR-320a-3p, hsa-miR-147b-3p
GANAB	22	hsa-miR-148a-3p, hsa-miR-20b-5p, hsa-miR-335-5p, hsa-miR-7-5p, hsa-miR-9-3p, hsa-miR-138-5p, hsa-miR-361-5p, hsa-miR-339-5p, hsa-miR-574-5p, hsa-miR-589-5p, hsa-miR-769-5p, hsa-miR-188-5p, hsa-miR-320d, hsa-miR-629-5p, hsa-miR-29c-5p, hsa-miR-342-5p, hsa-miR-

		1307-3p, hsa-miR-378i, hsa-miR-1-3p, hsa-miR-133a-3p, hsa-miR-887-3p, hsa-miR-320a-3p
HIPK1	22	hsa-miR-148a-3p, hsa-miR-20b-5p, hsa-miR-335-5p, hsa-miR-7-5p, hsa-miR-138-5p, hsa-miR-361-5p, hsa-miR-589-5p, hsa-miR-769-5p, hsa-miR-320d, hsa-miR-1275, hsa-miR-629-5p, hsa-miR-342-5p, hsa-miR-335-3p, hsa-miR-500a-3p, hsa-miR-671-5p, hsa-miR-2110, hsa-miR-320c, hsa-miR-378i, hsa-miR-1-3p, hsa-miR-887-3p, hsa-miR-320a-3p, hsa-miR-147b-3p
HNRNPA2B1	22	hsa-miR-361-5p, hsa-miR-20b-5p, hsa-miR-335-5p, hsa-miR-7-5p, hsa-miR-9-3p, hsa-miR-138-5p, hsa-miR-589-5p, hsa-miR-769-5p, hsa-miR-188-5p, hsa-miR-320d, hsa-miR-629-5p, hsa-miR-342-5p, hsa-miR-335-3p, hsa-miR-500a-3p, hsa-miR-671-5p, hsa-miR-1307-5p, hsa-miR-2110, hsa-miR-320c, hsa-miR-378i, hsa-miR-1-3p, hsa-miR-133a-3p, hsa-miR-320a-3p
ILF3	22	hsa-miR-7-5p, hsa-miR-769-5p, hsa-miR-148a-3p, hsa-miR-20b-5p, hsa-miR-335-5p, hsa-miR-138-5p, hsa-miR-361-5p, hsa-miR-339-5p, hsa-miR-589-5p, hsa-miR-188-5p, hsa-miR-320d, hsa-miR-1275, hsa-miR-629-5p, hsa-miR-29c-5p, hsa-miR-361-3p, hsa-miR-342-5p, hsa-miR-335-3p, hsa-miR-671-5p, hsa-miR-1307-3p, hsa-miR-378i, hsa-miR-320a-3p, hsa-miR-147b-3p
LATS2	22	hsa-miR-574-5p, hsa-miR-148a-3p, hsa-miR-20b-5p, hsa-miR-335-5p, hsa-miR-7-5p, hsa-miR-138-5p, hsa-miR-589-5p, hsa-miR-188-5p, hsa-miR-320d, hsa-miR-1275, hsa-miR-629-5p, hsa-miR-29c-5p, hsa-miR-361-3p, hsa-miR-342-5p, hsa-miR-335-3p, hsa-miR-500a-3p, hsa-miR-671-5p, hsa-miR-2110, hsa-miR-320c, hsa-miR-1-3p, hsa-miR-887-3p, hsa-miR-320a-3p
LMNB2	22	hsa-miR-7-5p, hsa-miR-342-5p, hsa-miR-378i, hsa-miR-148a-3p, hsa-miR-20b-5p, hsa-miR-335-5p, hsa-miR-138-5p, hsa-miR-361-5p, hsa-miR-339-5p, hsa-miR-574-5p, hsa-miR-629-5p, hsa-miR-29c-5p, hsa-miR-361-3p, hsa-miR-335-3p, hsa-miR-500a-3p, hsa-miR-671-5p, hsa-miR-1307-3p, hsa-miR-2110, hsa-miR-320c, hsa-miR-3615, hsa-miR-320a-3p, hsa-miR-147b-3p
LRRC58	22	hsa-miR-20b-5p, hsa-miR-574-5p, hsa-miR-1275, hsa-miR-675-3p, hsa-miR-4516, hsa-miR-874-3p, hsa-miR-148a-3p, hsa-miR-7-5p, hsa-miR-9-3p, hsa-miR-138-5p, hsa-miR-361-5p, hsa-miR-769-5p, hsa-miR-320d, hsa-miR-629-5p, hsa-miR-335-3p, hsa-miR-425-3p, hsa-miR-671-5p,

		hsa-miR-320c, hsa-miR-1-3p, hsa-miR-133a-3p, hsa-miR-320a-3p, hsa-miR-147b-3p
MTPN	22	hsa-miR-500a-3p, hsa-miR-148a-3p, hsa-miR-20b-5p, hsa-miR-7-5p, hsa-miR-9-3p, hsa-miR-138-5p, hsa-miR-361-5p, hsa-miR-574-5p, hsa-miR-589-5p, hsa-miR-320d, hsa-miR-1275, hsa-miR-629-5p, hsa-miR-342-5p, hsa-miR-425-3p, hsa-miR-671-5p, hsa-miR-2110, hsa-miR-320c, hsa-miR-874-3p, hsa-miR-1468-5p, hsa-miR-1-3p, hsa-miR-320a-3p, hsa-miR-190b-5p
MYC	22	hsa-miR-148a-3p, hsa-miR-335-5p, hsa-miR-7-5p, hsa-miR-20b-5p, hsa-miR-138-5p, hsa-miR-339-5p, hsa-miR-574-5p, hsa-miR-589-5p, hsa-miR-769-5p, hsa-miR-320d, hsa-miR-629-5p, hsa-miR-29c-5p, hsa-miR-335-3p, hsa-miR-500a-3p, hsa-miR-671-5p, hsa-miR-1307-3p, hsa-miR-1307-5p, hsa-miR-3168, hsa-miR-320c, hsa-miR-3615, hsa-miR-133a-3p, hsa-miR-320a-3p
NCOR2	22	hsa-miR-2110, hsa-miR-148a-3p, hsa-miR-20b-5p, hsa-miR-335-5p, hsa-miR-7-5p, hsa-miR-138-5p, hsa-miR-574-5p, hsa-miR-769-5p, hsa-miR-629-5p, hsa-miR-29c-5p, hsa-miR-361-3p, hsa-miR-342-5p, hsa-miR-335-3p, hsa-miR-500a-3p, hsa-miR-1307-5p, hsa-miR-320c, hsa-miR-3615, hsa-miR-378i, hsa-miR-874-3p, hsa-miR-887-3p, hsa-miR-320a-3p, hsa-miR-147b-3p
NFE2L1	22	hsa-miR-342-5p, hsa-miR-148a-3p, hsa-miR-20b-5p, hsa-miR-335-5p, hsa-miR-7-5p, hsa-miR-138-5p, hsa-miR-339-5p, hsa-miR-574-5p, hsa-miR-769-5p, hsa-miR-629-5p, hsa-miR-361-3p, hsa-miR-335-3p, hsa-miR-425-3p, hsa-miR-500a-3p, hsa-miR-1307-5p, hsa-miR-2110, hsa-miR-320c, hsa-miR-3615, hsa-miR-874-3p, hsa-miR-1-3p, hsa-miR-320a-3p, hsa-miR-147b-3p
NFIC	22	hsa-miR-339-5p, hsa-miR-769-5p, hsa-miR-2110, hsa-miR-4516, hsa-miR-148a-3p, hsa-miR-20b-5p, hsa-miR-335-5p, hsa-miR-7-5p, hsa-miR-138-5p, hsa-miR-361-5p, hsa-miR-574-5p, hsa-miR-320d, hsa-miR-361-3p, hsa-miR-335-3p, hsa-miR-1307-3p, hsa-miR-1307-5p, hsa-miR-320c, hsa-miR-378i, hsa-miR-887-3p, hsa-miR-320a-3p, hsa-miR-147b-3p, hsa-miR-190b-5p
P4HB	22	hsa-miR-188-5p, hsa-miR-378i, hsa-miR-887-3p, hsa-miR-148a-3p, hsa-miR-20b-5p, hsa-miR-335-5p, hsa-miR-339-5p, hsa-miR-574-5p, hsa-miR-589-5p, hsa-miR-769-5p, hsa-miR-320d, hsa-miR-335-3p, hsa-miR-425-3p, hsa-miR-500a-3p, hsa-miR-671-5p, hsa-miR-1307-3p, hsa-miR-

		320c, hsa-miR-3615, hsa-miR-874-3p, hsa-miR-1468-5p, hsa-miR-320a-3p, hsa-miR-147b-3p
PAFAH1B1	22	hsa-miR-20b-5p, hsa-miR-339-5p, hsa-miR-148a-3p, hsa-miR-335-5p, hsa-miR-7-5p, hsa-miR-9-3p, hsa-miR-138-5p, hsa-miR-361-5p, hsa-miR-1275, hsa-miR-29c-5p, hsa-miR-342-5p, hsa-miR-335-3p, hsa-miR-425-3p, hsa-miR-500a-3p, hsa-miR-671-5p, hsa-miR-2110, hsa-miR-320c, hsa-miR-378i, hsa-miR-1-3p, hsa-miR-133a-3p, hsa-miR-887-3p, hsa-miR-320a-3p
PANK3	22	hsa-miR-20b-5p, hsa-miR-148a-3p, hsa-miR-335-5p, hsa-miR-7-5p, hsa-miR-138-5p, hsa-miR-589-5p, hsa-miR-769-5p, hsa-miR-188-5p, hsa-miR-320d, hsa-miR-629-5p, hsa-miR-335-3p, hsa-miR-425-3p, hsa-miR-500a-3p, hsa-miR-671-5p, hsa-miR-3168, hsa-miR-2110, hsa-miR-320c, hsa-miR-1468-5p, hsa-miR-1-3p, hsa-miR-133a-3p, hsa-miR-320a-3p, hsa-miR-190b-5p
PLEC	22	hsa-miR-7-5p, hsa-miR-574-5p, hsa-miR-148a-3p, hsa-miR-335-5p, hsa-miR-138-5p, hsa-miR-361-5p, hsa-miR-339-5p, hsa-miR-589-5p, hsa-miR-769-5p, hsa-miR-361-3p, hsa-miR-335-3p, hsa-miR-425-3p, hsa-miR-500a-3p, hsa-miR-671-5p, hsa-miR-1307-3p, hsa-miR-1307-5p, hsa-miR-2110, hsa-miR-3615, hsa-miR-378i, hsa-miR-1-3p, hsa-miR-320a-3p, hsa-miR-147b-3p
PRRC2C	22	hsa-miR-342-5p, hsa-miR-148a-3p, hsa-miR-20b-5p, hsa-miR-7-5p, hsa-miR-138-5p, hsa-miR-361-5p, hsa-miR-589-5p, hsa-miR-769-5p, hsa-miR-320d, hsa-miR-29c-5p, hsa-miR-335-3p, hsa-miR-500a-3p, hsa-miR-671-5p, hsa-miR-2110, hsa-miR-320c, hsa-miR-3615, hsa-miR-378i, hsa-miR-1-3p, hsa-miR-133a-3p, hsa-miR-887-3p, hsa-miR-320a-3p, hsa-miR-147b-3p
PUM1	22	hsa-miR-335-3p, hsa-miR-148a-3p, hsa-miR-20b-5p, hsa-miR-335-5p, hsa-miR-7-5p, hsa-miR-138-5p, hsa-miR-361-5p, hsa-miR-339-5p, hsa-miR-574-5p, hsa-miR-589-5p, hsa-miR-769-5p, hsa-miR-320d, hsa-miR-342-5p, hsa-miR-425-3p, hsa-miR-500a-3p, hsa-miR-671-5p, hsa-miR-675-3p, hsa-miR-2110, hsa-miR-320c, hsa-miR-1-3p, hsa-miR-133a-3p, hsa-miR-320a-3p
PURA	22	hsa-miR-148a-3p, hsa-miR-20b-5p, hsa-miR-7-5p, hsa-miR-138-5p, hsa-miR-339-5p, hsa-miR-574-5p, hsa-miR-589-5p, hsa-miR-769-5p, hsa-miR-629-5p, hsa-miR-361-3p, hsa-miR-342-5p, hsa-miR-335-3p, hsa-miR-500a-3p, hsa-miR-671-5p, hsa-miR-675-3p, hsa-miR-2110, hsa-

		miR-320c, hsa-miR-378i, hsa-miR-1-3p, hsa-miR-133a-3p, hsa-miR-887-3p, hsa-miR-320a-3p
RREB1	22	hsa-miR-1275, hsa-miR-671-5p, hsa-miR-148a-3p, hsa-miR-20b-5p, hsa-miR-7-5p, hsa-miR-9-3p, hsa-miR-138-5p, hsa-miR-574-5p, hsa-miR-589-5p, hsa-miR-769-5p, hsa-miR-320d, hsa-miR-629-5p, hsa-miR-29c-5p, hsa-miR-342-5p, hsa-miR-335-3p, hsa-miR-500a-3p, hsa-miR-1307-5p, hsa-miR-3168, hsa-miR-320c, hsa-miR-133a-3p, hsa-miR-320a-3p, hsa-miR-147b-3p
SF3B1	22	hsa-miR-1307-3p, hsa-miR-148a-3p, hsa-miR-20b-5p, hsa-miR-335-5p, hsa-miR-7-5p, hsa-miR-138-5p, hsa-miR-361-5p, hsa-miR-339-5p, hsa-miR-769-5p, hsa-miR-188-5p, hsa-miR-342-5p, hsa-miR-335-3p, hsa-miR-425-3p, hsa-miR-500a-3p, hsa-miR-671-5p, hsa-miR-2110, hsa-miR-320c, hsa-miR-378i, hsa-miR-1468-5p, hsa-miR-1-3p, hsa-miR-133a-3p, hsa-miR-320a-3p
SF3B3	22	hsa-miR-20b-5p, hsa-miR-1275, hsa-miR-148a-3p, hsa-miR-335-5p, hsa-miR-7-5p, hsa-miR-138-5p, hsa-miR-361-5p, hsa-miR-339-5p, hsa-miR-629-5p, hsa-miR-361-3p, hsa-miR-335-3p, hsa-miR-500a-3p, hsa-miR-551b-3p, hsa-miR-671-5p, hsa-miR-1307-5p, hsa-miR-2110, hsa-miR-320c, hsa-miR-1468-5p, hsa-miR-1-3p, hsa-miR-133a-3p, hsa-miR-320a-3p, hsa-miR-147b-3p
SIPA1L1	22	hsa-miR-4516, hsa-miR-148a-3p, hsa-miR-20b-5p, hsa-miR-7-5p, hsa-miR-138-5p, hsa-miR-361-5p, hsa-miR-589-5p, hsa-miR-769-5p, hsa-miR-342-5p, hsa-miR-335-3p, hsa-miR-500a-3p, hsa-miR-551b-3p, hsa-miR-671-5p, hsa-miR-2110, hsa-miR-320c, hsa-miR-3615, hsa-miR-378i, hsa-miR-874-3p, hsa-miR-133a-3p, hsa-miR-887-3p, hsa-miR-320a-3p, hsa-miR-147b-3p
TANC2	22	hsa-miR-335-5p, hsa-miR-148a-3p, hsa-miR-20b-5p, hsa-miR-7-5p, hsa-miR-9-3p, hsa-miR-138-5p, hsa-miR-361-5p, hsa-miR-574-5p, hsa-miR-589-5p, hsa-miR-769-5p, hsa-miR-188-5p, hsa-miR-320d, hsa-miR-629-5p, hsa-miR-361-3p, hsa-miR-342-5p, hsa-miR-335-3p, hsa-miR-671-5p, hsa-miR-2110, hsa-miR-320c, hsa-miR-1-3p, hsa-miR-320a-3p, hsa-miR-147b-3p
TBL1XR1	22	hsa-miR-20b-5p, hsa-miR-4516, hsa-miR-148a-3p, hsa-miR-335-5p, hsa-miR-7-5p, hsa-miR-9-3p, hsa-miR-138-5p, hsa-miR-361-5p, hsa-miR-339-5p, hsa-miR-574-5p, hsa-miR-188-5p, hsa-miR-361-3p, hsa-miR-342-5p, hsa-miR-335-3p, hsa-miR-500a-3p, hsa-miR-3168, hsa-

		miR-675-3p, hsa-miR-2110, hsa-miR-320c, hsa-miR-1-3p, hsa-miR-887-3p, hsa-miR-320a-3p
THBS1	22	hsa-miR-675-3p, hsa-miR-320c, hsa-miR-1-3p, hsa-miR-148a-3p, hsa-miR-20b-5p, hsa-miR-335-5p, hsa-miR-7-5p, hsa-miR-9-3p, hsa-miR-138-5p, hsa-miR-361-5p, hsa-miR-339-5p, hsa-miR-769-5p, hsa-miR-320d, hsa-miR-335-3p, hsa-miR-425-3p, hsa-miR-500a-3p, hsa-miR-671-5p, hsa-miR-2110, hsa-miR-378i, hsa-miR-133a-3p, hsa-miR-320a-3p, hsa-miR-147b-3p
TNPO1	22	hsa-miR-148a-3p, hsa-miR-20b-5p, hsa-miR-335-5p, hsa-miR-7-5p, hsa-miR-9-3p, hsa-miR-138-5p, hsa-miR-361-5p, hsa-miR-574-5p, hsa-miR-589-5p, hsa-miR-1275, hsa-miR-629-5p, hsa-miR-342-5p, hsa-miR-335-3p, hsa-miR-500a-3p, hsa-miR-1307-5p, hsa-miR-675-3p, hsa-miR-2110, hsa-miR-320c, hsa-miR-1-3p, hsa-miR-887-3p, hsa-miR-320a-3p, hsa-miR-190b-5p
TNPO2	22	hsa-miR-4516, hsa-miR-148a-3p, hsa-miR-20b-5p, hsa-miR-335-5p, hsa-miR-7-5p, hsa-miR-138-5p, hsa-miR-361-5p, hsa-miR-574-5p, hsa-miR-589-5p, hsa-miR-769-5p, hsa-miR-629-5p, hsa-miR-29c-5p, hsa-miR-342-5p, hsa-miR-335-3p, hsa-miR-500a-3p, hsa-miR-671-5p, hsa-miR-1307-3p, hsa-miR-2110, hsa-miR-320c, hsa-miR-3615, hsa-miR-1-3p, hsa-miR-320a-3p
TRIP12	22	hsa-miR-148a-3p, hsa-miR-20b-5p, hsa-miR-7-5p, hsa-miR-9-3p, hsa-miR-138-5p, hsa-miR-361-5p, hsa-miR-339-5p, hsa-miR-589-5p, hsa-miR-769-5p, hsa-miR-1275, hsa-miR-629-5p, hsa-miR-335-3p, hsa-miR-425-3p, hsa-miR-500a-3p, hsa-miR-671-5p, hsa-miR-2110, hsa-miR-320c, hsa-miR-378i, hsa-miR-1-3p, hsa-miR-133a-3p, hsa-miR-320a-3p, hsa-miR-147b-3p
UBR5	22	hsa-miR-20b-5p, hsa-miR-1-3p, hsa-miR-148a-3p, hsa-miR-335-5p, hsa-miR-7-5p, hsa-miR-138-5p, hsa-miR-361-5p, hsa-miR-339-5p, hsa-miR-574-5p, hsa-miR-589-5p, hsa-miR-188-5p, hsa-miR-1275, hsa-miR-335-3p, hsa-miR-425-3p, hsa-miR-671-5p, hsa-miR-378i, hsa-miR-874-3p, hsa-miR-133a-3p, hsa-miR-887-3p, hsa-miR-320a-3p, hsa-miR-147b-3p, hsa-miR-190b-5p
UHMK1	22	hsa-miR-1-3p, hsa-miR-335-5p, hsa-miR-874-3p, hsa-miR-148a-3p, hsa-miR-20b-5p, hsa-miR-7-5p, hsa-miR-9-3p, hsa-miR-138-5p, hsa-miR-361-5p, hsa-miR-574-5p, hsa-miR-589-5p, hsa-miR-188-5p, hsa-miR-629-5p, hsa-miR-335-3p, hsa-miR-425-3p, hsa-miR-671-5p, hsa-miR-

		1307-3p, hsa-miR-3168, hsa-miR-2110, hsa-miR-320c, hsa-miR-887-3p, hsa-miR-320a-3p
USP34	22	hsa-miR-148a-3p, hsa-miR-20b-5p, hsa-miR-335-5p, hsa-miR-7-5p, hsa-miR-138-5p, hsa-miR-361-5p, hsa-miR-574-5p, hsa-miR-589-5p, hsa-miR-769-5p, hsa-miR-629-5p, hsa-miR-29c-5p, hsa-miR-361-3p, hsa-miR-335-3p, hsa-miR-425-3p, hsa-miR-500a-3p, hsa-miR-671-5p, hsa-miR-2110, hsa-miR-320c, hsa-miR-378i, hsa-miR-133a-3p, hsa-miR-887-3p, hsa-miR-320a-3p
WAC	22	hsa-miR-20b-5p, hsa-miR-148a-3p, hsa-miR-7-5p, hsa-miR-9-3p, hsa-miR-138-5p, hsa-miR-361-5p, hsa-miR-339-5p, hsa-miR-574-5p, hsa-miR-589-5p, hsa-miR-188-5p, hsa-miR-629-5p, hsa-miR-342-5p, hsa-miR-335-3p, hsa-miR-425-3p, hsa-miR-500a-3p, hsa-miR-1307-5p, hsa-miR-320c, hsa-miR-378i, hsa-miR-1-3p, hsa-miR-133a-3p, hsa-miR-320a-3p, hsa-miR-147b-3p
ADIPOR2	21	hsa-miR-148a-3p, hsa-miR-20b-5p, hsa-miR-335-5p, hsa-miR-7-5p, hsa-miR-9-3p, hsa-miR-138-5p, hsa-miR-361-5p, hsa-miR-574-5p, hsa-miR-589-5p, hsa-miR-320d, hsa-miR-1275, hsa-miR-629-5p, hsa-miR-335-3p, hsa-miR-500a-3p, hsa-miR-671-5p, hsa-miR-1307-5p, hsa-miR-2110, hsa-miR-887-3p, hsa-miR-12136, hsa-miR-320a-3p, hsa-miR-147b-3p
ANKRD40	21	hsa-miR-148a-3p, hsa-miR-20b-5p, hsa-miR-335-5p, hsa-miR-7-5p, hsa-miR-138-5p, hsa-miR-361-5p, hsa-miR-339-5p, hsa-miR-769-5p, hsa-miR-320d, hsa-miR-1275, hsa-miR-629-5p, hsa-miR-335-3p, hsa-miR-425-3p, hsa-miR-500a-3p, hsa-miR-671-5p, hsa-miR-2110, hsa-miR-320c, hsa-miR-378i, hsa-miR-1-3p, hsa-miR-320a-3p, hsa-miR-147b-3p
AP2B1	21	hsa-miR-188-5p, hsa-miR-148a-3p, hsa-miR-20b-5p, hsa-miR-335-5p, hsa-miR-7-5p, hsa-miR-361-5p, hsa-miR-589-5p, hsa-miR-320d, hsa-miR-629-5p, hsa-miR-29c-5p, hsa-miR-342-5p, hsa-miR-335-3p, hsa-miR-500a-3p, hsa-miR-671-5p, hsa-miR-2110, hsa-miR-320c, hsa-miR-378i, hsa-miR-874-3p, hsa-miR-1-3p, hsa-miR-320a-3p, hsa-miR-147b-3p
ARPP19	21	hsa-miR-574-5p, hsa-miR-1275, hsa-miR-4791, hsa-miR-1-3p, hsa-miR-148a-3p, hsa-miR-20b-5p, hsa-miR-335-5p, hsa-miR-7-5p, hsa-miR-9-3p, hsa-miR-138-5p, hsa-miR-589-5p, hsa-miR-769-5p, hsa-miR-320d, hsa-miR-335-3p, hsa-miR-671-5p, hsa-miR-3168, hsa-miR-2110, hsa-miR-320c, hsa-miR-378i, hsa-miR-887-3p, hsa-miR-320a-3p

CCND1	21	hsa-miR-20b-5p, hsa-miR-138-5p, hsa-miR-574-5p, hsa-miR-342-5p, hsa-miR-1-3p, hsa-miR-148a-3p, hsa-miR-335-5p, hsa-miR-7-5p, hsa-miR-361-5p, hsa-miR-769-5p, hsa-miR-188-5p, hsa-miR-320d, hsa-miR-335-3p, hsa-miR-500a-3p, hsa-miR-671-5p, hsa-miR-320c, hsa-miR-3615, hsa-miR-133a-3p, hsa-miR-887-3p, hsa-miR-320a-3p, hsa-miR-147b-3p
CDK6	21	hsa-miR-20b-5p, hsa-miR-138-5p, hsa-miR-335-5p, hsa-miR-7-5p, hsa-miR-9-3p, hsa-miR-574-5p, hsa-miR-769-5p, hsa-miR-320d, hsa-miR-335-3p, hsa-miR-425-3p, hsa-miR-500a-3p, hsa-miR-671-5p, hsa-miR-1307-3p, hsa-miR-2110, hsa-miR-320c, hsa-miR-3615, hsa-miR-378i, hsa-miR-1468-5p, hsa-miR-1-3p, hsa-miR-320a-3p, hsa-miR-147b-3p
CLUH	21	hsa-miR-148a-3p, hsa-miR-20b-5p, hsa-miR-335-5p, hsa-miR-7-5p, hsa-miR-138-5p, hsa-miR-339-5p, hsa-miR-574-5p, hsa-miR-589-5p, hsa-miR-769-5p, hsa-miR-320d, hsa-miR-629-5p, hsa-miR-29c-5p, hsa-miR-335-3p, hsa-miR-500a-3p, hsa-miR-671-5p, hsa-miR-1307-3p, hsa-miR-1307-5p, hsa-miR-320c, hsa-miR-3615, hsa-miR-320a-3p, hsa-miR-147b-3p
CPEB2	21	hsa-miR-335-5p, hsa-miR-148a-3p, hsa-miR-20b-5p, hsa-miR-7-5p, hsa-miR-9-3p, hsa-miR-138-5p, hsa-miR-361-5p, hsa-miR-320d, hsa-miR-629-5p, hsa-miR-29c-5p, hsa-miR-342-5p, hsa-miR-335-3p, hsa-miR-500a-3p, hsa-miR-671-5p, hsa-miR-2110, hsa-miR-320c, hsa-miR-378i, hsa-miR-1-3p, hsa-miR-887-3p, hsa-miR-320a-3p, hsa-miR-147b-3p
CTNNB1	21	hsa-miR-148a-3p, hsa-miR-335-5p, hsa-miR-7-5p, hsa-miR-138-5p, hsa-miR-361-5p, hsa-miR-339-5p, hsa-miR-589-5p, hsa-miR-769-5p, hsa-miR-320d, hsa-miR-629-5p, hsa-miR-361-3p, hsa-miR-335-3p, hsa-miR-500a-3p, hsa-miR-671-5p, hsa-miR-2110, hsa-miR-320c, hsa-miR-378i, hsa-miR-1-3p, hsa-miR-133a-3p, hsa-miR-887-3p, hsa-miR-320a-3p
DIP2B	21	hsa-miR-148a-3p, hsa-miR-20b-5p, hsa-miR-335-5p, hsa-miR-7-5p, hsa-miR-339-5p, hsa-miR-589-5p, hsa-miR-769-5p, hsa-miR-188-5p, hsa-miR-342-5p, hsa-miR-500a-3p, hsa-miR-671-5p, hsa-miR-1307-3p, hsa-miR-1307-5p, hsa-miR-2110, hsa-miR-320c, hsa-miR-378i, hsa-miR-1-3p, hsa-miR-133a-3p, hsa-miR-887-3p, hsa-miR-320a-3p, hsa-miR-147b-3p
ENAH	21	hsa-miR-188-5p, hsa-miR-148a-3p, hsa-miR-20b-5p, hsa-miR-335-5p, hsa-miR-7-5p, hsa-miR-138-5p, hsa-miR-574-5p, hsa-miR-589-5p, hsa-miR-769-5p, hsa-miR-1275, hsa-miR-342-5p, hsa-miR-335-3p, hsa-miR-

		500a-3p, hsa-miR-671-5p, hsa-miR-2110, hsa-miR-320c, hsa-miR-1-3p, hsa-miR-133a-3p, hsa-miR-887-3p, hsa-miR-320a-3p, hsa-miR-147b-3p
FEM1B	21	hsa-miR-20b-5p, hsa-miR-138-5p, hsa-miR-148a-3p, hsa-miR-335-5p, hsa-miR-7-5p, hsa-miR-574-5p, hsa-miR-589-5p, hsa-miR-188-5p, hsa-miR-320d, hsa-miR-1275, hsa-miR-629-5p, hsa-miR-29c-5p, hsa-miR-361-3p, hsa-miR-342-5p, hsa-miR-335-3p, hsa-miR-425-3p, hsa-miR-500a-3p, hsa-miR-320c, hsa-miR-1-3p, hsa-miR-887-3p, hsa-miR-320a-3p
G3BP1	21	hsa-miR-1-3p, hsa-miR-7704, hsa-miR-148a-3p, hsa-miR-20b-5p, hsa-miR-335-5p, hsa-miR-7-5p, hsa-miR-361-5p, hsa-miR-589-5p, hsa-miR-320d, hsa-miR-629-5p, hsa-miR-335-3p, hsa-miR-425-3p, hsa-miR-671-5p, hsa-miR-1307-5p, hsa-miR-3168, hsa-miR-2110, hsa-miR-320c, hsa-miR-378i, hsa-miR-133a-3p, hsa-miR-320a-3p, hsa-miR-190b-5p
HERC1	21	hsa-miR-148a-3p, hsa-miR-20b-5p, hsa-miR-335-5p, hsa-miR-7-5p, hsa-miR-138-5p, hsa-miR-361-5p, hsa-miR-339-5p, hsa-miR-589-5p, hsa-miR-769-5p, hsa-miR-320d, hsa-miR-1275, hsa-miR-629-5p, hsa-miR-29c-5p, hsa-miR-335-3p, hsa-miR-500a-3p, hsa-miR-2110, hsa-miR-320c, hsa-miR-1-3p, hsa-miR-133a-3p, hsa-miR-320a-3p, hsa-miR-147b-3p
ITGB4	21	hsa-miR-335-5p, hsa-miR-1-3p, hsa-miR-148a-3p, hsa-miR-20b-5p, hsa-miR-7-5p, hsa-miR-9-3p, hsa-miR-138-5p, hsa-miR-339-5p, hsa-miR-574-5p, hsa-miR-589-5p, hsa-miR-769-5p, hsa-miR-188-5p, hsa-miR-320d, hsa-miR-361-3p, hsa-miR-500a-3p, hsa-miR-1307-3p, hsa-miR-1307-5p, hsa-miR-3615, hsa-miR-874-3p, hsa-miR-320a-3p, hsa-miR-147b-3p
LATS1	21	hsa-miR-335-5p, hsa-miR-148a-3p, hsa-miR-20b-5p, hsa-miR-7-5p, hsa-miR-138-5p, hsa-miR-361-5p, hsa-miR-574-5p, hsa-miR-769-5p, hsa-miR-320d, hsa-miR-1275, hsa-miR-629-5p, hsa-miR-342-5p, hsa-miR-335-3p, hsa-miR-500a-3p, hsa-miR-671-5p, hsa-miR-2110, hsa-miR-320c, hsa-miR-1-3p, hsa-miR-320a-3p, hsa-miR-147b-3p, hsa-miR-190b-5p
MAPK1	21	hsa-miR-20b-5p, hsa-miR-335-5p, hsa-miR-9-3p, hsa-miR-769-5p, hsa-miR-1307-3p, hsa-miR-2110, hsa-miR-148a-3p, hsa-miR-7-5p, hsa-miR-138-5p, hsa-miR-361-5p, hsa-miR-339-5p, hsa-miR-589-5p, hsa-miR-320d, hsa-miR-342-5p, hsa-miR-335-3p, hsa-miR-671-5p, hsa-miR-320c, hsa-miR-3615, hsa-miR-378i, hsa-miR-1-3p, hsa-miR-320a-3p

MGA	21	hsa-miR-335-5p, hsa-miR-148a-3p, hsa-miR-20b-5p, hsa-miR-7-5p, hsa-miR-138-5p, hsa-miR-361-5p, hsa-miR-589-5p, hsa-miR-769-5p, hsa-miR-1275, hsa-miR-629-5p, hsa-miR-342-5p, hsa-miR-335-3p, hsa-miR-671-5p, hsa-miR-2110, hsa-miR-320c, hsa-miR-3615, hsa-miR-378i, hsa-miR-874-3p, hsa-miR-1-3p, hsa-miR-320a-3p, hsa-miR-147b-3p
MYCBP2	21	hsa-miR-148a-3p, hsa-miR-20b-5p, hsa-miR-335-5p, hsa-miR-7-5p, hsa-miR-138-5p, hsa-miR-361-5p, hsa-miR-339-5p, hsa-miR-574-5p, hsa-miR-589-5p, hsa-miR-188-5p, hsa-miR-342-5p, hsa-miR-335-3p, hsa-miR-425-3p, hsa-miR-500a-3p, hsa-miR-671-5p, hsa-miR-320c, hsa-miR-1-3p, hsa-miR-133a-3p, hsa-miR-887-3p, hsa-miR-320a-3p, hsa-miR-147b-3p
NACC1	21	hsa-miR-7-5p, hsa-miR-138-5p, hsa-miR-574-5p, hsa-miR-589-5p, hsa-miR-1275, hsa-miR-361-3p, hsa-miR-2110, hsa-miR-3615, hsa-miR-148a-3p, hsa-miR-20b-5p, hsa-miR-335-5p, hsa-miR-339-5p, hsa-miR-769-5p, hsa-miR-342-5p, hsa-miR-335-3p, hsa-miR-500a-3p, hsa-miR-671-5p, hsa-miR-378i, hsa-miR-1-3p, hsa-miR-320a-3p, hsa-miR-147b-3p
NSD2	21	hsa-miR-20b-5p, hsa-miR-339-5p, hsa-miR-148a-3p, hsa-miR-335-5p, hsa-miR-7-5p, hsa-miR-138-5p, hsa-miR-361-5p, hsa-miR-574-5p, hsa-miR-589-5p, hsa-miR-769-5p, hsa-miR-188-5p, hsa-miR-629-5p, hsa-miR-361-3p, hsa-miR-342-5p, hsa-miR-335-3p, hsa-miR-500a-3p, hsa-miR-671-5p, hsa-miR-1307-5p, hsa-miR-1-3p, hsa-miR-320a-3p, hsa-miR-147b-3p
NUCKS1	21	hsa-miR-675-3p, hsa-miR-148a-3p, hsa-miR-20b-5p, hsa-miR-335-5p, hsa-miR-7-5p, hsa-miR-361-5p, hsa-miR-574-5p, hsa-miR-320d, hsa-miR-361-3p, hsa-miR-335-3p, hsa-miR-500a-3p, hsa-miR-671-5p, hsa-miR-2110, hsa-miR-320c, hsa-miR-378i, hsa-miR-874-3p, hsa-miR-1468-5p, hsa-miR-1-3p, hsa-miR-320a-3p, hsa-miR-147b-3p, hsa-miR-190b-5p
PNN	21	hsa-miR-320d, hsa-miR-320c, hsa-miR-1-3p, hsa-miR-148a-3p, hsa-miR-20b-5p, hsa-miR-335-5p, hsa-miR-7-5p, hsa-miR-361-5p, hsa-miR-574-5p, hsa-miR-769-5p, hsa-miR-335-3p, hsa-miR-425-3p, hsa-miR-500a-3p, hsa-miR-671-5p, hsa-miR-1307-5p, hsa-miR-3615, hsa-miR-378i, hsa-miR-874-3p, hsa-miR-1468-5p, hsa-miR-320a-3p, hsa-miR-147b-3p
PPP1R12A	21	hsa-miR-148a-3p, hsa-miR-20b-5p, hsa-miR-7-5p, hsa-miR-9-3p, hsa-miR-138-5p, hsa-miR-361-5p, hsa-miR-188-5p, hsa-miR-320d, hsa-miR-

		629-5p, hsa-miR-29c-5p, hsa-miR-342-5p, hsa-miR-335-3p, hsa-miR-671-5p, hsa-miR-320c, hsa-miR-3615, hsa-miR-378i, hsa-miR-1-3p, hsa-miR-133a-3p, hsa-miR-887-3p, hsa-miR-320a-3p, hsa-miR-147b-3p
PRDM2	21	hsa-miR-188-5p, hsa-miR-148a-3p, hsa-miR-20b-5p, hsa-miR-7-5p, hsa-miR-9-3p, hsa-miR-138-5p, hsa-miR-574-5p, hsa-miR-589-5p, hsa-miR-320d, hsa-miR-629-5p, hsa-miR-335-3p, hsa-miR-500a-3p, hsa-miR-671-5p, hsa-miR-2110, hsa-miR-320c, hsa-miR-3615, hsa-miR-378i, hsa-miR-1-3p, hsa-miR-320a-3p, hsa-miR-147b-3p, hsa-miR-190b-5p
PRRC2B	21	hsa-miR-1275, hsa-miR-342-5p, hsa-miR-335-3p, hsa-miR-500a-3p, hsa-miR-148a-3p, hsa-miR-20b-5p, hsa-miR-335-5p, hsa-miR-7-5p, hsa-miR-138-5p, hsa-miR-361-5p, hsa-miR-339-5p, hsa-miR-589-5p, hsa-miR-361-3p, hsa-miR-671-5p, hsa-miR-1307-3p, hsa-miR-2110, hsa-miR-320c, hsa-miR-378i, hsa-miR-1-3p, hsa-miR-133a-3p, hsa-miR-320a-3p
PTBP1	21	hsa-miR-671-5p, hsa-miR-1-3p, hsa-miR-133a-3p, hsa-miR-148a-3p, hsa-miR-20b-5p, hsa-miR-7-5p, hsa-miR-9-3p, hsa-miR-138-5p, hsa-miR-339-5p, hsa-miR-574-5p, hsa-miR-589-5p, hsa-miR-320d, hsa-miR-342-5p, hsa-miR-335-3p, hsa-miR-425-3p, hsa-miR-500a-3p, hsa-miR-1307-5p, hsa-miR-2110, hsa-miR-320c, hsa-miR-320a-3p, hsa-miR-147b-3p
QSER1	21	hsa-miR-1-3p, hsa-miR-148a-3p, hsa-miR-20b-5p, hsa-miR-335-5p, hsa-miR-7-5p, hsa-miR-138-5p, hsa-miR-361-5p, hsa-miR-339-5p, hsa-miR-769-5p, hsa-miR-320d, hsa-miR-629-5p, hsa-miR-342-5p, hsa-miR-335-3p, hsa-miR-500a-3p, hsa-miR-671-5p, hsa-miR-2110, hsa-miR-320c, hsa-miR-1468-5p, hsa-miR-133a-3p, hsa-miR-320a-3p, hsa-miR-147b-3p
RAD21	21	hsa-miR-574-5p, hsa-miR-148a-3p, hsa-miR-20b-5p, hsa-miR-335-5p, hsa-miR-7-5p, hsa-miR-9-3p, hsa-miR-138-5p, hsa-miR-361-5p, hsa-miR-339-5p, hsa-miR-320d, hsa-miR-629-5p, hsa-miR-335-3p, hsa-miR-500a-3p, hsa-miR-671-5p, hsa-miR-2110, hsa-miR-320c, hsa-miR-378i, hsa-miR-1468-5p, hsa-miR-133a-3p, hsa-miR-320a-3p, hsa-miR-147b-3p
RORA	21	hsa-miR-20b-5p, hsa-miR-335-5p, hsa-miR-148a-3p, hsa-miR-7-5p, hsa-miR-9-3p, hsa-miR-361-5p, hsa-miR-339-5p, hsa-miR-574-5p, hsa-miR-188-5p, hsa-miR-1275, hsa-miR-629-5p, hsa-miR-335-3p, hsa-miR-671-5p, hsa-miR-2110, hsa-miR-320c, hsa-miR-378i, hsa-miR-874-3p, hsa-miR-1-3p, hsa-miR-887-3p, hsa-miR-320a-3p, hsa-miR-190b-5p

SERTAD2	21	hsa-miR-7-5p, hsa-miR-4516, hsa-miR-148a-3p, hsa-miR-20b-5p, hsa-miR-335-5p, hsa-miR-138-5p, hsa-miR-574-5p, hsa-miR-769-5p, hsa-miR-320d, hsa-miR-1275, hsa-miR-629-5p, hsa-miR-342-5p, hsa-miR-335-3p, hsa-miR-500a-3p, hsa-miR-671-5p, hsa-miR-1307-3p, hsa-miR-2110, hsa-miR-320c, hsa-miR-1-3p, hsa-miR-320a-3p, hsa-miR-147b-3p
SKI	21	hsa-miR-20b-5p, hsa-miR-342-5p, hsa-miR-2110, hsa-miR-148a-3p, hsa-miR-335-5p, hsa-miR-7-5p, hsa-miR-138-5p, hsa-miR-361-5p, hsa-miR-339-5p, hsa-miR-574-5p, hsa-miR-589-5p, hsa-miR-769-5p, hsa-miR-1275, hsa-miR-629-5p, hsa-miR-335-3p, hsa-miR-500a-3p, hsa-miR-3615, hsa-miR-1-3p, hsa-miR-887-3p, hsa-miR-320a-3p, hsa-miR-147b-3p
SOX4	21	hsa-miR-335-5p, hsa-miR-20b-5p, hsa-miR-138-5p, hsa-miR-4516, hsa-miR-133a-3p, hsa-miR-148a-3p, hsa-miR-7-5p, hsa-miR-9-3p, hsa-miR-361-5p, hsa-miR-339-5p, hsa-miR-320d, hsa-miR-335-3p, hsa-miR-425-3p, hsa-miR-500a-3p, hsa-miR-671-5p, hsa-miR-1307-3p, hsa-miR-1307-5p, hsa-miR-2110, hsa-miR-320c, hsa-miR-3615, hsa-miR-320a-3p
SSH2	21	hsa-miR-20b-5p, hsa-miR-574-5p, hsa-miR-148a-3p, hsa-miR-335-5p, hsa-miR-7-5p, hsa-miR-138-5p, hsa-miR-361-5p, hsa-miR-589-5p, hsa-miR-769-5p, hsa-miR-188-5p, hsa-miR-1275, hsa-miR-629-5p, hsa-miR-361-3p, hsa-miR-335-3p, hsa-miR-500a-3p, hsa-miR-671-5p, hsa-miR-2110, hsa-miR-320c, hsa-miR-378i, hsa-miR-320a-3p, hsa-miR-147b-3p
TNKS	21	hsa-miR-148a-3p, hsa-miR-20b-5p, hsa-miR-7-5p, hsa-miR-9-3p, hsa-miR-138-5p, hsa-miR-361-5p, hsa-miR-339-5p, hsa-miR-574-5p, hsa-miR-589-5p, hsa-miR-769-5p, hsa-miR-320d, hsa-miR-1275, hsa-miR-342-5p, hsa-miR-335-3p, hsa-miR-671-5p, hsa-miR-2110, hsa-miR-3615, hsa-miR-1-3p, hsa-miR-887-3p, hsa-miR-320a-3p, hsa-miR-147b-3p
TRIB1	21	hsa-miR-335-5p, hsa-miR-148a-3p, hsa-miR-20b-5p, hsa-miR-7-5p, hsa-miR-138-5p, hsa-miR-339-5p, hsa-miR-574-5p, hsa-miR-589-5p, hsa-miR-1275, hsa-miR-361-3p, hsa-miR-342-5p, hsa-miR-335-3p, hsa-miR-425-3p, hsa-miR-671-5p, hsa-miR-2110, hsa-miR-320c, hsa-miR-3615, hsa-miR-378i, hsa-miR-1-3p, hsa-miR-320a-3p, hsa-miR-147b-3p
UBN2	21	hsa-miR-574-5p, hsa-miR-3168, hsa-miR-148a-3p, hsa-miR-20b-5p, hsa-miR-335-5p, hsa-miR-7-5p, hsa-miR-138-5p, hsa-miR-361-5p, hsa-miR-589-5p, hsa-miR-1275, hsa-miR-629-5p, hsa-miR-361-3p, hsa-miR-342-5p, hsa-miR-335-3p, hsa-miR-500a-3p, hsa-miR-671-5p, hsa-miR-1307-3p, hsa-miR-2110, hsa-miR-320c, hsa-miR-3615, hsa-miR-320a-3p

UBR4	21	hsa-miR-148a-3p, hsa-miR-20b-5p, hsa-miR-335-5p, hsa-miR-7-5p, hsa-miR-138-5p, hsa-miR-361-5p, hsa-miR-339-5p, hsa-miR-574-5p, hsa-miR-769-5p, hsa-miR-320d, hsa-miR-629-5p, hsa-miR-29c-5p, hsa-miR-335-3p, hsa-miR-425-3p, hsa-miR-500a-3p, hsa-miR-671-5p, hsa-miR-2110, hsa-miR-320c, hsa-miR-3615, hsa-miR-320a-3p, hsa-miR-147b-3p
USP22	21	hsa-miR-361-5p, hsa-miR-29c-5p, hsa-miR-148a-3p, hsa-miR-20b-5p, hsa-miR-335-5p, hsa-miR-7-5p, hsa-miR-138-5p, hsa-miR-574-5p, hsa-miR-589-5p, hsa-miR-769-5p, hsa-miR-1275, hsa-miR-335-3p, hsa-miR-671-5p, hsa-miR-1307-5p, hsa-miR-2110, hsa-miR-320c, hsa-miR-874-3p, hsa-miR-1-3p, hsa-miR-133a-3p, hsa-miR-320a-3p, hsa-miR-147b-3p
YWHAB	21	hsa-miR-148a-3p, hsa-miR-769-5p, hsa-miR-20b-5p, hsa-miR-7-5p, hsa-miR-9-3p, hsa-miR-138-5p, hsa-miR-361-5p, hsa-miR-320d, hsa-miR-629-5p, hsa-miR-29c-5p, hsa-miR-361-3p, hsa-miR-342-5p, hsa-miR-335-3p, hsa-miR-425-3p, hsa-miR-500a-3p, hsa-miR-2110, hsa-miR-320c, hsa-miR-378i, hsa-miR-1-3p, hsa-miR-320a-3p, hsa-miR-147b-3p
ZBED6	21	hsa-miR-148a-3p, hsa-miR-20b-5p, hsa-miR-335-5p, hsa-miR-7-5p, hsa-miR-138-5p, hsa-miR-574-5p, hsa-miR-769-5p, hsa-miR-320d, hsa-miR-629-5p, hsa-miR-29c-5p, hsa-miR-335-3p, hsa-miR-500a-3p, hsa-miR-671-5p, hsa-miR-2110, hsa-miR-320c, hsa-miR-3615, hsa-miR-378i, hsa-miR-1468-5p, hsa-miR-1-3p, hsa-miR-887-3p, hsa-miR-320a-3p
ZNF460	21	hsa-miR-335-5p, hsa-miR-7-5p, hsa-miR-9-3p, hsa-miR-361-5p, hsa-miR-339-5p, hsa-miR-361-3p, hsa-miR-335-3p, hsa-miR-425-3p, hsa-miR-500a-3p, hsa-miR-148a-3p, hsa-miR-20b-5p, hsa-miR-138-5p, hsa-miR-574-5p, hsa-miR-1275, hsa-miR-342-5p, hsa-miR-1307-3p, hsa-miR-320c, hsa-miR-1468-5p, hsa-miR-1-3p, hsa-miR-320a-3p, hsa-miR-147b-3p
ACTB	20	hsa-miR-1275, hsa-miR-1307-3p, hsa-miR-1-3p, hsa-miR-148a-3p, hsa-miR-20b-5p, hsa-miR-7-5p, hsa-miR-9-3p, hsa-miR-138-5p, hsa-miR-339-5p, hsa-miR-589-5p, hsa-miR-769-5p, hsa-miR-342-5p, hsa-miR-335-3p, hsa-miR-500a-3p, hsa-miR-671-5p, hsa-miR-1307-5p, hsa-miR-2110, hsa-miR-3615, hsa-miR-887-3p, hsa-miR-147b-3p
ADAR	20	hsa-miR-1-3p, hsa-miR-20b-5p, hsa-miR-148a-3p, hsa-miR-7-5p, hsa-miR-138-5p, hsa-miR-361-5p, hsa-miR-589-5p, hsa-miR-769-5p, hsa-miR-320d, hsa-miR-1275, hsa-miR-361-3p, hsa-miR-342-5p, hsa-miR-335-3p, hsa-miR-500a-3p, hsa-miR-671-5p, hsa-miR-2110, hsa-miR-320c, hsa-miR-378i, hsa-miR-320a-3p, hsa-miR-147b-3p

ANKHD1-EIF4EBP3	20	hsa-miR-148a-3p, hsa-miR-20b-5p, hsa-miR-7-5p, hsa-miR-138-5p, hsa-miR-361-5p, hsa-miR-339-5p, hsa-miR-574-5p, hsa-miR-589-5p, hsa-miR-320d, hsa-miR-629-5p, hsa-miR-342-5p, hsa-miR-335-3p, hsa-miR-500a-3p, hsa-miR-671-5p, hsa-miR-2110, hsa-miR-320c, hsa-miR-378i, hsa-miR-133a-3p, hsa-miR-320a-3p, hsa-miR-147b-3p
ARL5B	20	hsa-miR-335-5p, hsa-miR-138-5p, hsa-miR-20b-5p, hsa-miR-7-5p, hsa-miR-9-3p, hsa-miR-361-5p, hsa-miR-339-5p, hsa-miR-574-5p, hsa-miR-589-5p, hsa-miR-342-5p, hsa-miR-335-3p, hsa-miR-425-3p, hsa-miR-500a-3p, hsa-miR-671-5p, hsa-miR-320c, hsa-miR-3615, hsa-miR-378i, hsa-miR-1-3p, hsa-miR-320a-3p, hsa-miR-147b-3p
ATXN1	20	hsa-miR-20b-5p, hsa-miR-335-5p, hsa-miR-7-5p, hsa-miR-188-5p, hsa-miR-361-3p, hsa-miR-874-3p, hsa-miR-148a-3p, hsa-miR-138-5p, hsa-miR-574-5p, hsa-miR-589-5p, hsa-miR-769-5p, hsa-miR-320d, hsa-miR-1275, hsa-miR-629-5p, hsa-miR-29c-5p, hsa-miR-3168, hsa-miR-2110, hsa-miR-320c, hsa-miR-320a-3p, hsa-miR-147b-3p
ATXN1L	20	hsa-miR-2110, hsa-miR-148a-3p, hsa-miR-20b-5p, hsa-miR-335-5p, hsa-miR-7-5p, hsa-miR-361-5p, hsa-miR-339-5p, hsa-miR-574-5p, hsa-miR-769-5p, hsa-miR-629-5p, hsa-miR-361-3p, hsa-miR-342-5p, hsa-miR-500a-3p, hsa-miR-671-5p, hsa-miR-1307-5p, hsa-miR-320c, hsa-miR-378i, hsa-miR-874-3p, hsa-miR-1-3p, hsa-miR-320a-3p
BBX	20	hsa-miR-20b-5p, hsa-miR-335-5p, hsa-miR-9-3p, hsa-miR-148a-3p, hsa-miR-7-5p, hsa-miR-138-5p, hsa-miR-361-5p, hsa-miR-339-5p, hsa-miR-589-5p, hsa-miR-769-5p, hsa-miR-629-5p, hsa-miR-342-5p, hsa-miR-335-3p, hsa-miR-500a-3p, hsa-miR-671-5p, hsa-miR-2110, hsa-miR-378i, hsa-miR-1-3p, hsa-miR-320a-3p, hsa-miR-147b-3p
BMPR2	20	hsa-miR-20b-5p, hsa-miR-7-5p, hsa-miR-3615, hsa-miR-9-3p, hsa-miR-138-5p, hsa-miR-361-5p, hsa-miR-589-5p, hsa-miR-320d, hsa-miR-629-5p, hsa-miR-29c-5p, hsa-miR-342-5p, hsa-miR-335-3p, hsa-miR-425-3p, hsa-miR-671-5p, hsa-miR-2110, hsa-miR-320c, hsa-miR-1-3p, hsa-miR-887-3p, hsa-miR-320a-3p, hsa-miR-147b-3p
CAPRIN1	20	hsa-miR-1-3p, hsa-miR-148a-3p, hsa-miR-20b-5p, hsa-miR-335-5p, hsa-miR-7-5p, hsa-miR-138-5p, hsa-miR-361-5p, hsa-miR-574-5p, hsa-miR-769-5p, hsa-miR-188-5p, hsa-miR-629-5p, hsa-miR-29c-5p, hsa-miR-500a-3p, hsa-miR-671-5p, hsa-miR-2110, hsa-miR-320c, hsa-miR-1468-5p, hsa-miR-133a-3p, hsa-miR-320a-3p, hsa-miR-147b-3p

CBX6	20	hsa-miR-361-3p, hsa-miR-342-5p, hsa-miR-874-3p, hsa-miR-148a-3p, hsa-miR-20b-5p, hsa-miR-335-5p, hsa-miR-7-5p, hsa-miR-138-5p, hsa-miR-339-5p, hsa-miR-589-5p, hsa-miR-769-5p, hsa-miR-1275, hsa-miR-629-5p, hsa-miR-500a-3p, hsa-miR-671-5p, hsa-miR-320c, hsa-miR-1-3p, hsa-miR-133a-3p, hsa-miR-320a-3p, hsa-miR-147b-3p
CCDC6	20	hsa-miR-20b-5p, hsa-miR-1275, hsa-miR-335-3p, hsa-miR-671-5p, hsa-miR-148a-3p, hsa-miR-335-5p, hsa-miR-7-5p, hsa-miR-138-5p, hsa-miR-574-5p, hsa-miR-361-3p, hsa-miR-342-5p, hsa-miR-425-3p, hsa-miR-500a-3p, hsa-miR-1307-3p, hsa-miR-320c, hsa-miR-378i, hsa-miR-1-3p, hsa-miR-320a-3p, hsa-miR-147b-3p, hsa-miR-190b-5p
CCNT1	20	hsa-miR-335-5p, hsa-miR-4516, hsa-miR-148a-3p, hsa-miR-20b-5p, hsa-miR-7-5p, hsa-miR-9-3p, hsa-miR-138-5p, hsa-miR-361-5p, hsa-miR-574-5p, hsa-miR-769-5p, hsa-miR-1275, hsa-miR-629-5p, hsa-miR-29c-5p, hsa-miR-342-5p, hsa-miR-335-3p, hsa-miR-671-5p, hsa-miR-320c, hsa-miR-320a-3p, hsa-miR-147b-3p, hsa-miR-190b-5p
CCNT2	20	hsa-miR-7-5p, hsa-miR-500a-3p, hsa-miR-148a-3p, hsa-miR-20b-5p, hsa-miR-335-5p, hsa-miR-9-3p, hsa-miR-138-5p, hsa-miR-361-5p, hsa-miR-589-5p, hsa-miR-769-5p, hsa-miR-188-5p, hsa-miR-320d, hsa-miR-335-3p, hsa-miR-425-3p, hsa-miR-2110, hsa-miR-320c, hsa-miR-874-3p, hsa-miR-1468-5p, hsa-miR-1-3p, hsa-miR-320a-3p
CDC42BPA	20	hsa-miR-335-5p, hsa-miR-148a-3p, hsa-miR-20b-5p, hsa-miR-7-5p, hsa-miR-138-5p, hsa-miR-361-5p, hsa-miR-339-5p, hsa-miR-589-5p, hsa-miR-769-5p, hsa-miR-188-5p, hsa-miR-29c-5p, hsa-miR-335-3p, hsa-miR-500a-3p, hsa-miR-671-5p, hsa-miR-320c, hsa-miR-3615, hsa-miR-378i, hsa-miR-1-3p, hsa-miR-320a-3p, hsa-miR-147b-3p
CDK12	20	hsa-miR-148a-3p, hsa-miR-20b-5p, hsa-miR-335-5p, hsa-miR-7-5p, hsa-miR-138-5p, hsa-miR-361-5p, hsa-miR-589-5p, hsa-miR-769-5p, hsa-miR-361-3p, hsa-miR-335-3p, hsa-miR-425-3p, hsa-miR-500a-3p, hsa-miR-671-5p, hsa-miR-1307-5p, hsa-miR-3615, hsa-miR-378i, hsa-miR-1-3p, hsa-miR-887-3p, hsa-miR-320a-3p, hsa-miR-147b-3p
CHD2	20	hsa-miR-148a-3p, hsa-miR-20b-5p, hsa-miR-335-5p, hsa-miR-7-5p, hsa-miR-9-3p, hsa-miR-138-5p, hsa-miR-361-5p, hsa-miR-574-5p, hsa-miR-589-5p, hsa-miR-769-5p, hsa-miR-188-5p, hsa-miR-361-3p, hsa-miR-335-3p, hsa-miR-500a-3p, hsa-miR-671-5p, hsa-miR-1307-3p, hsa-miR-320c, hsa-miR-133a-3p, hsa-miR-320a-3p, hsa-miR-147b-3p

CKAP5	20	hsa-miR-148a-3p, hsa-miR-20b-5p, hsa-miR-7-5p, hsa-miR-138-5p, hsa-miR-361-5p, hsa-miR-574-5p, hsa-miR-769-5p, hsa-miR-320d, hsa-miR-629-5p, hsa-miR-361-3p, hsa-miR-342-5p, hsa-miR-335-3p, hsa-miR-425-3p, hsa-miR-671-5p, hsa-miR-2110, hsa-miR-320c, hsa-miR-378i, hsa-miR-1-3p, hsa-miR-887-3p, hsa-miR-320a-3p
CRK	20	hsa-miR-20b-5p, hsa-miR-9-3p, hsa-miR-320d, hsa-miR-2110, hsa-miR-320c, hsa-miR-1-3p, hsa-miR-335-5p, hsa-miR-7-5p, hsa-miR-138-5p, hsa-miR-361-5p, hsa-miR-339-5p, hsa-miR-574-5p, hsa-miR-589-5p, hsa-miR-769-5p, hsa-miR-500a-3p, hsa-miR-671-5p, hsa-miR-1307-3p, hsa-miR-3615, hsa-miR-320a-3p, hsa-miR-147b-3p
DDB1	20	hsa-miR-148a-3p, hsa-miR-20b-5p, hsa-miR-335-5p, hsa-miR-7-5p, hsa-miR-138-5p, hsa-miR-361-5p, hsa-miR-574-5p, hsa-miR-769-5p, hsa-miR-629-5p, hsa-miR-29c-5p, hsa-miR-342-5p, hsa-miR-500a-3p, hsa-miR-671-5p, hsa-miR-1307-5p, hsa-miR-320c, hsa-miR-3615, hsa-miR-378i, hsa-miR-1-3p, hsa-miR-320a-3p, hsa-miR-147b-3p
DYRK2	20	hsa-miR-20b-5p, hsa-miR-1275, hsa-miR-629-5p, hsa-miR-148a-3p, hsa-miR-7-5p, hsa-miR-361-5p, hsa-miR-339-5p, hsa-miR-574-5p, hsa-miR-589-5p, hsa-miR-769-5p, hsa-miR-29c-5p, hsa-miR-335-3p, hsa-miR-500a-3p, hsa-miR-671-5p, hsa-miR-3168, hsa-miR-320c, hsa-miR-874-3p, hsa-miR-1-3p, hsa-miR-320a-3p, hsa-miR-147b-3p
EPHA4	20	hsa-miR-20b-5p, hsa-miR-335-5p, hsa-miR-361-5p, hsa-miR-148a-3p, hsa-miR-7-5p, hsa-miR-138-5p, hsa-miR-339-5p, hsa-miR-589-5p, hsa-miR-188-5p, hsa-miR-320d, hsa-miR-629-5p, hsa-miR-29c-5p, hsa-miR-425-3p, hsa-miR-2110, hsa-miR-320c, hsa-miR-1-3p, hsa-miR-133a-3p, hsa-miR-887-3p, hsa-miR-320a-3p, hsa-miR-147b-3p
FAM120A	20	hsa-miR-148a-3p, hsa-miR-20b-5p, hsa-miR-335-5p, hsa-miR-7-5p, hsa-miR-138-5p, hsa-miR-361-5p, hsa-miR-339-5p, hsa-miR-574-5p, hsa-miR-589-5p, hsa-miR-769-5p, hsa-miR-629-5p, hsa-miR-342-5p, hsa-miR-335-3p, hsa-miR-425-3p, hsa-miR-500a-3p, hsa-miR-671-5p, hsa-miR-2110, hsa-miR-874-3p, hsa-miR-1-3p, hsa-miR-320a-3p
FAM168B	20	hsa-miR-148a-3p, hsa-miR-20b-5p, hsa-miR-335-5p, hsa-miR-7-5p, hsa-miR-138-5p, hsa-miR-361-5p, hsa-miR-339-5p, hsa-miR-589-5p, hsa-miR-769-5p, hsa-miR-320d, hsa-miR-29c-5p, hsa-miR-335-3p, hsa-miR-425-3p, hsa-miR-500a-3p, hsa-miR-671-5p, hsa-miR-2110, hsa-miR-320c, hsa-miR-874-3p, hsa-miR-1-3p, hsa-miR-320a-3p

FBXO28	20	hsa-miR-9-3p, hsa-miR-320d, hsa-miR-320c, hsa-miR-148a-3p, hsa-miR-20b-5p, hsa-miR-335-5p, hsa-miR-7-5p, hsa-miR-138-5p, hsa-miR-339-5p, hsa-miR-589-5p, hsa-miR-188-5p, hsa-miR-629-5p, hsa-miR-335-3p, hsa-miR-671-5p, hsa-miR-3168, hsa-miR-2110, hsa-miR-3615, hsa-miR-1468-5p, hsa-miR-1-3p, hsa-miR-320a-3p
GATAD2B	20	hsa-miR-7-5p, hsa-miR-335-3p, hsa-miR-148a-3p, hsa-miR-20b-5p, hsa-miR-138-5p, hsa-miR-589-5p, hsa-miR-320d, hsa-miR-1275, hsa-miR-361-3p, hsa-miR-342-5p, hsa-miR-425-3p, hsa-miR-500a-3p, hsa-miR-671-5p, hsa-miR-3168, hsa-miR-320c, hsa-miR-378i, hsa-miR-1-3p, hsa-miR-320a-3p, hsa-miR-147b-3p, hsa-miR-190b-5p
GNA13	20	hsa-miR-1-3p, hsa-miR-20b-5p, hsa-miR-335-5p, hsa-miR-7-5p, hsa-miR-9-3p, hsa-miR-138-5p, hsa-miR-361-5p, hsa-miR-574-5p, hsa-miR-589-5p, hsa-miR-188-5p, hsa-miR-361-3p, hsa-miR-335-3p, hsa-miR-425-3p, hsa-miR-500a-3p, hsa-miR-671-5p, hsa-miR-1307-3p, hsa-miR-320c, hsa-miR-378i, hsa-miR-320a-3p, hsa-miR-190b-5p
GPI	20	hsa-miR-671-5p, hsa-miR-20b-5p, hsa-miR-7-5p, hsa-miR-9-3p, hsa-miR-138-5p, hsa-miR-361-5p, hsa-miR-574-5p, hsa-miR-589-5p, hsa-miR-320d, hsa-miR-342-5p, hsa-miR-335-3p, hsa-miR-500a-3p, hsa-miR-2110, hsa-miR-320c, hsa-miR-378i, hsa-miR-874-3p, hsa-miR-133a-3p, hsa-miR-887-3p, hsa-miR-320a-3p, hsa-miR-147b-3p
HDLBP	20	hsa-miR-7-5p, hsa-miR-425-3p, hsa-miR-148a-3p, hsa-miR-20b-5p, hsa-miR-138-5p, hsa-miR-339-5p, hsa-miR-574-5p, hsa-miR-769-5p, hsa-miR-320d, hsa-miR-629-5p, hsa-miR-361-3p, hsa-miR-500a-3p, hsa-miR-671-5p, hsa-miR-1307-5p, hsa-miR-3168, hsa-miR-320c, hsa-miR-874-3p, hsa-miR-1-3p, hsa-miR-133a-3p, hsa-miR-147b-3p
HNRNPUL1	20	hsa-miR-138-5p, hsa-miR-671-5p, hsa-miR-2110, hsa-miR-148a-3p, hsa-miR-20b-5p, hsa-miR-7-5p, hsa-miR-361-5p, hsa-miR-339-5p, hsa-miR-769-5p, hsa-miR-320d, hsa-miR-342-5p, hsa-miR-335-3p, hsa-miR-425-3p, hsa-miR-500a-3p, hsa-miR-320c, hsa-miR-3615, hsa-miR-378i, hsa-miR-887-3p, hsa-miR-320a-3p, hsa-miR-147b-3p
INO80D	20	hsa-miR-148a-3p, hsa-miR-20b-5p, hsa-miR-335-5p, hsa-miR-7-5p, hsa-miR-9-3p, hsa-miR-138-5p, hsa-miR-361-5p, hsa-miR-339-5p, hsa-miR-574-5p, hsa-miR-589-5p, hsa-miR-769-5p, hsa-miR-320d, hsa-miR-1275, hsa-miR-629-5p, hsa-miR-361-3p, hsa-miR-342-5p, hsa-miR-1307-3p, hsa-miR-2110, hsa-miR-320c, hsa-miR-320a-3p

IPO7	20	hsa-miR-7-5p, hsa-miR-148a-3p, hsa-miR-20b-5p, hsa-miR-335-5p, hsa-miR-138-5p, hsa-miR-361-5p, hsa-miR-574-5p, hsa-miR-589-5p, hsa-miR-320d, hsa-miR-361-3p, hsa-miR-342-5p, hsa-miR-335-3p, hsa-miR-500a-3p, hsa-miR-671-5p, hsa-miR-2110, hsa-miR-320c, hsa-miR-874-3p, hsa-miR-133a-3p, hsa-miR-320a-3p, hsa-miR-147b-3p
KAT6A	20	hsa-miR-574-5p, hsa-miR-188-5p, hsa-miR-148a-3p, hsa-miR-20b-5p, hsa-miR-335-5p, hsa-miR-7-5p, hsa-miR-138-5p, hsa-miR-361-5p, hsa-miR-339-5p, hsa-miR-589-5p, hsa-miR-629-5p, hsa-miR-335-3p, hsa-miR-671-5p, hsa-miR-1307-3p, hsa-miR-3168, hsa-miR-2110, hsa-miR-3615, hsa-miR-1-3p, hsa-miR-320a-3p, hsa-miR-147b-3p
KMT2E	20	hsa-miR-335-5p, hsa-miR-148a-3p, hsa-miR-20b-5p, hsa-miR-7-5p, hsa-miR-9-3p, hsa-miR-138-5p, hsa-miR-361-5p, hsa-miR-574-5p, hsa-miR-769-5p, hsa-miR-320d, hsa-miR-1275, hsa-miR-629-5p, hsa-miR-342-5p, hsa-miR-335-3p, hsa-miR-500a-3p, hsa-miR-671-5p, hsa-miR-320c, hsa-miR-1-3p, hsa-miR-887-3p, hsa-miR-190b-5p
LRP1	20	hsa-miR-335-5p, hsa-miR-1-3p, hsa-miR-148a-3p, hsa-miR-20b-5p, hsa-miR-7-5p, hsa-miR-138-5p, hsa-miR-361-5p, hsa-miR-339-5p, hsa-miR-574-5p, hsa-miR-589-5p, hsa-miR-769-5p, hsa-miR-629-5p, hsa-miR-335-3p, hsa-miR-500a-3p, hsa-miR-1307-3p, hsa-miR-2110, hsa-miR-320c, hsa-miR-133a-3p, hsa-miR-320a-3p, hsa-miR-147b-3p
MAP4K4	20	hsa-miR-148a-3p, hsa-miR-20b-5p, hsa-miR-335-5p, hsa-miR-7-5p, hsa-miR-361-5p, hsa-miR-574-5p, hsa-miR-589-5p, hsa-miR-769-5p, hsa-miR-188-5p, hsa-miR-320d, hsa-miR-361-3p, hsa-miR-335-3p, hsa-miR-425-3p, hsa-miR-500a-3p, hsa-miR-671-5p, hsa-miR-320c, hsa-miR-3615, hsa-miR-1468-5p, hsa-miR-1-3p, hsa-miR-320a-3p
MDM2	20	hsa-miR-20b-5p, hsa-miR-335-5p, hsa-miR-339-5p, hsa-miR-335-3p, hsa-miR-500a-3p, hsa-miR-148a-3p, hsa-miR-7-5p, hsa-miR-9-3p, hsa-miR-138-5p, hsa-miR-361-5p, hsa-miR-769-5p, hsa-miR-320d, hsa-miR-1275, hsa-miR-629-5p, hsa-miR-425-3p, hsa-miR-671-5p, hsa-miR-2110, hsa-miR-320c, hsa-miR-133a-3p, hsa-miR-320a-3p
MKI67	20	hsa-miR-1-3p, hsa-miR-148a-3p, hsa-miR-20b-5p, hsa-miR-335-5p, hsa-miR-7-5p, hsa-miR-138-5p, hsa-miR-361-5p, hsa-miR-339-5p, hsa-miR-574-5p, hsa-miR-589-5p, hsa-miR-769-5p, hsa-miR-188-5p, hsa-miR-320d, hsa-miR-629-5p, hsa-miR-335-3p, hsa-miR-500a-3p, hsa-miR-671-5p, hsa-miR-320c, hsa-miR-320a-3p, hsa-miR-147b-3p

NCOA3	20	hsa-miR-20b-5p, hsa-miR-335-5p, hsa-miR-342-5p, hsa-miR-148a-3p, hsa-miR-7-5p, hsa-miR-138-5p, hsa-miR-361-5p, hsa-miR-339-5p, hsa-miR-769-5p, hsa-miR-188-5p, hsa-miR-629-5p, hsa-miR-500a-3p, hsa-miR-671-5p, hsa-miR-320c, hsa-miR-874-3p, hsa-miR-1468-5p, hsa-miR-1-3p, hsa-miR-133a-3p, hsa-miR-320a-3p, hsa-miR-147b-3p
PABPC1	20	hsa-miR-20b-5p, hsa-miR-335-5p, hsa-miR-7-5p, hsa-miR-9-3p, hsa-miR-138-5p, hsa-miR-361-5p, hsa-miR-339-5p, hsa-miR-574-5p, hsa-miR-589-5p, hsa-miR-769-5p, hsa-miR-342-5p, hsa-miR-335-3p, hsa-miR-500a-3p, hsa-miR-671-5p, hsa-miR-1307-5p, hsa-miR-320c, hsa-miR-874-3p, hsa-miR-1468-5p, hsa-miR-320a-3p, hsa-miR-147b-3p
PKM	20	hsa-miR-769-5p, hsa-miR-1275, hsa-miR-133a-3p, hsa-miR-148a-3p, hsa-miR-20b-5p, hsa-miR-7-5p, hsa-miR-9-3p, hsa-miR-361-5p, hsa-miR-339-5p, hsa-miR-574-5p, hsa-miR-335-3p, hsa-miR-425-3p, hsa-miR-671-5p, hsa-miR-1307-5p, hsa-miR-3168, hsa-miR-320c, hsa-miR-3615, hsa-miR-378i, hsa-miR-1-3p, hsa-miR-320a-3p
POU2F1	20	hsa-miR-148a-3p, hsa-miR-20b-5p, hsa-miR-335-5p, hsa-miR-7-5p, hsa-miR-361-5p, hsa-miR-574-5p, hsa-miR-589-5p, hsa-miR-320d, hsa-miR-1275, hsa-miR-629-5p, hsa-miR-342-5p, hsa-miR-335-3p, hsa-miR-425-3p, hsa-miR-500a-3p, hsa-miR-671-5p, hsa-miR-320c, hsa-miR-874-3p, hsa-miR-1-3p, hsa-miR-320a-3p, hsa-miR-147b-3p
PPP1CC	20	hsa-miR-188-5p, hsa-miR-148a-3p, hsa-miR-20b-5p, hsa-miR-335-5p, hsa-miR-7-5p, hsa-miR-138-5p, hsa-miR-339-5p, hsa-miR-320d, hsa-miR-1275, hsa-miR-629-5p, hsa-miR-29c-5p, hsa-miR-335-3p, hsa-miR-425-3p, hsa-miR-500a-3p, hsa-miR-3168, hsa-miR-2110, hsa-miR-3615, hsa-miR-378i, hsa-miR-1-3p, hsa-miR-320a-3p
PPP1R10	20	hsa-miR-335-5p, hsa-miR-148a-3p, hsa-miR-20b-5p, hsa-miR-7-5p, hsa-miR-138-5p, hsa-miR-574-5p, hsa-miR-589-5p, hsa-miR-320d, hsa-miR-1275, hsa-miR-29c-5p, hsa-miR-342-5p, hsa-miR-335-3p, hsa-miR-425-3p, hsa-miR-671-5p, hsa-miR-2110, hsa-miR-3615, hsa-miR-1-3p, hsa-miR-887-3p, hsa-miR-320a-3p, hsa-miR-147b-3p
PUM2	20	hsa-miR-148a-3p, hsa-miR-20b-5p, hsa-miR-335-5p, hsa-miR-7-5p, hsa-miR-138-5p, hsa-miR-361-5p, hsa-miR-339-5p, hsa-miR-589-5p, hsa-miR-769-5p, hsa-miR-29c-5p, hsa-miR-335-3p, hsa-miR-500a-3p, hsa-miR-671-5p, hsa-miR-1307-3p, hsa-miR-1307-5p, hsa-miR-320c, hsa-miR-1468-5p, hsa-miR-1-3p, hsa-miR-133a-3p, hsa-miR-320a-3p

QKI	20	hsa-miR-148a-3p, hsa-miR-20b-5p, hsa-miR-335-5p, hsa-miR-7-5p, hsa-miR-9-3p, hsa-miR-138-5p, hsa-miR-361-5p, hsa-miR-589-5p, hsa-miR-769-5p, hsa-miR-320d, hsa-miR-1275, hsa-miR-29c-5p, hsa-miR-335-3p, hsa-miR-500a-3p, hsa-miR-320c, hsa-miR-3615, hsa-miR-378i, hsa-miR-1-3p, hsa-miR-133a-3p, hsa-miR-320a-3p
QRICH1	20	hsa-miR-148a-3p, hsa-miR-20b-5p, hsa-miR-7-5p, hsa-miR-138-5p, hsa-miR-361-5p, hsa-miR-339-5p, hsa-miR-574-5p, hsa-miR-769-5p, hsa-miR-629-5p, hsa-miR-342-5p, hsa-miR-500a-3p, hsa-miR-671-5p, hsa-miR-2110, hsa-miR-320c, hsa-miR-874-3p, hsa-miR-1468-5p, hsa-miR-1-3p, hsa-miR-887-3p, hsa-miR-320a-3p, hsa-miR-147b-3p
RAPGEF2	20	hsa-miR-335-5p, hsa-miR-339-5p, hsa-miR-335-3p, hsa-miR-1-3p, hsa-miR-148a-3p, hsa-miR-20b-5p, hsa-miR-7-5p, hsa-miR-9-3p, hsa-miR-138-5p, hsa-miR-361-5p, hsa-miR-589-5p, hsa-miR-188-5p, hsa-miR-320d, hsa-miR-629-5p, hsa-miR-342-5p, hsa-miR-500a-3p, hsa-miR-671-5p, hsa-miR-2110, hsa-miR-320c, hsa-miR-320a-3p
RBFOX2	20	hsa-miR-320d, hsa-miR-148a-3p, hsa-miR-20b-5p, hsa-miR-335-5p, hsa-miR-7-5p, hsa-miR-9-3p, hsa-miR-138-5p, hsa-miR-361-5p, hsa-miR-339-5p, hsa-miR-574-5p, hsa-miR-589-5p, hsa-miR-188-5p, hsa-miR-361-3p, hsa-miR-671-5p, hsa-miR-320c, hsa-miR-378i, hsa-miR-1-3p, hsa-miR-887-3p, hsa-miR-320a-3p, hsa-miR-147b-3p
RBM39	20	hsa-miR-1-3p, hsa-miR-148a-3p, hsa-miR-20b-5p, hsa-miR-335-5p, hsa-miR-7-5p, hsa-miR-9-3p, hsa-miR-138-5p, hsa-miR-361-5p, hsa-miR-574-5p, hsa-miR-188-5p, hsa-miR-320d, hsa-miR-29c-5p, hsa-miR-361-3p, hsa-miR-335-3p, hsa-miR-425-3p, hsa-miR-671-5p, hsa-miR-1307-5p, hsa-miR-320c, hsa-miR-133a-3p, hsa-miR-320a-3p
RICTOR	20	hsa-miR-335-5p, hsa-miR-1275, hsa-miR-148a-3p, hsa-miR-20b-5p, hsa-miR-7-5p, hsa-miR-361-5p, hsa-miR-574-5p, hsa-miR-769-5p, hsa-miR-188-5p, hsa-miR-320d, hsa-miR-629-5p, hsa-miR-342-5p, hsa-miR-335-3p, hsa-miR-500a-3p, hsa-miR-2110, hsa-miR-320c, hsa-miR-378i, hsa-miR-1-3p, hsa-miR-887-3p, hsa-miR-320a-3p
RPRD2	20	hsa-miR-20b-5p, hsa-miR-335-5p, hsa-miR-7-5p, hsa-miR-769-5p, hsa-miR-2110, hsa-miR-1-3p, hsa-miR-148a-3p, hsa-miR-138-5p, hsa-miR-361-5p, hsa-miR-339-5p, hsa-miR-629-5p, hsa-miR-342-5p, hsa-miR-335-3p, hsa-miR-500a-3p, hsa-miR-671-5p, hsa-miR-320c, hsa-miR-3615, hsa-miR-887-3p, hsa-miR-320a-3p, hsa-miR-147b-3p

SCD	20	hsa-miR-20b-5p, hsa-miR-335-5p, hsa-miR-574-5p, hsa-miR-7-5p, hsa-miR-138-5p, hsa-miR-361-5p, hsa-miR-339-5p, hsa-miR-589-5p, hsa-miR-769-5p, hsa-miR-335-3p, hsa-miR-425-3p, hsa-miR-500a-3p, hsa-miR-671-5p, hsa-miR-1307-3p, hsa-miR-3168, hsa-miR-2110, hsa-miR-320c, hsa-miR-1-3p, hsa-miR-320a-3p, hsa-miR-147b-3p
SEC16A	20	hsa-miR-20b-5p, hsa-miR-1-3p, hsa-miR-148a-3p, hsa-miR-335-5p, hsa-miR-7-5p, hsa-miR-9-3p, hsa-miR-138-5p, hsa-miR-339-5p, hsa-miR-574-5p, hsa-miR-1275, hsa-miR-335-3p, hsa-miR-425-3p, hsa-miR-500a-3p, hsa-miR-671-5p, hsa-miR-1307-3p, hsa-miR-1307-5p, hsa-miR-2110, hsa-miR-320c, hsa-miR-320a-3p, hsa-miR-147b-3p
SEC24C	20	hsa-miR-335-5p, hsa-miR-148a-3p, hsa-miR-20b-5p, hsa-miR-7-5p, hsa-miR-138-5p, hsa-miR-361-5p, hsa-miR-339-5p, hsa-miR-769-5p, hsa-miR-320d, hsa-miR-1275, hsa-miR-629-5p, hsa-miR-361-3p, hsa-miR-342-5p, hsa-miR-335-3p, hsa-miR-500a-3p, hsa-miR-671-5p, hsa-miR-2110, hsa-miR-3615, hsa-miR-874-3p, hsa-miR-320a-3p
SF1	20	hsa-miR-148a-3p, hsa-miR-20b-5p, hsa-miR-335-5p, hsa-miR-7-5p, hsa-miR-361-5p, hsa-miR-339-5p, hsa-miR-574-5p, hsa-miR-589-5p, hsa-miR-769-5p, hsa-miR-188-5p, hsa-miR-320d, hsa-miR-361-3p, hsa-miR-500a-3p, hsa-miR-671-5p, hsa-miR-1307-3p, hsa-miR-2110, hsa-miR-3615, hsa-miR-1-3p, hsa-miR-320a-3p, hsa-miR-147b-3p
SLC38A2	20	hsa-miR-148a-3p, hsa-miR-335-5p, hsa-miR-9-3p, hsa-miR-320d, hsa-miR-320c, hsa-miR-20b-5p, hsa-miR-7-5p, hsa-miR-138-5p, hsa-miR-361-5p, hsa-miR-589-5p, hsa-miR-769-5p, hsa-miR-629-5p, hsa-miR-361-3p, hsa-miR-342-5p, hsa-miR-335-3p, hsa-miR-500a-3p, hsa-miR-671-5p, hsa-miR-378i, hsa-miR-1-3p, hsa-miR-320a-3p
SLC7A1	20	hsa-miR-671-5p, hsa-miR-148a-3p, hsa-miR-20b-5p, hsa-miR-335-5p, hsa-miR-7-5p, hsa-miR-138-5p, hsa-miR-361-5p, hsa-miR-339-5p, hsa-miR-574-5p, hsa-miR-589-5p, hsa-miR-1275, hsa-miR-29c-5p, hsa-miR-335-3p, hsa-miR-425-3p, hsa-miR-500a-3p, hsa-miR-378i, hsa-miR-1-3p, hsa-miR-133a-3p, hsa-miR-320a-3p, hsa-miR-147b-3p
SMAD3	20	hsa-miR-335-5p, hsa-miR-148a-3p, hsa-miR-20b-5p, hsa-miR-7-5p, hsa-miR-138-5p, hsa-miR-361-5p, hsa-miR-589-5p, hsa-miR-1275, hsa-miR-629-5p, hsa-miR-29c-5p, hsa-miR-342-5p, hsa-miR-425-3p, hsa-miR-500a-3p, hsa-miR-671-5p, hsa-miR-1307-5p, hsa-miR-2110, hsa-miR-378i, hsa-miR-1-3p, hsa-miR-320a-3p, hsa-miR-147b-3p

SPTAN1	20	hsa-miR-148a-3p, hsa-miR-20b-5p, hsa-miR-335-5p, hsa-miR-7-5p, hsa-miR-138-5p, hsa-miR-361-5p, hsa-miR-339-5p, hsa-miR-188-5p, hsa-miR-320d, hsa-miR-629-5p, hsa-miR-335-3p, hsa-miR-500a-3p, hsa-miR-671-5p, hsa-miR-320c, hsa-miR-3615, hsa-miR-1468-5p, hsa-miR-1-3p, hsa-miR-133a-3p, hsa-miR-320a-3p, hsa-miR-147b-3p
THRAP3	20	hsa-miR-148a-3p, hsa-miR-20b-5p, hsa-miR-335-5p, hsa-miR-7-5p, hsa-miR-138-5p, hsa-miR-361-5p, hsa-miR-339-5p, hsa-miR-574-5p, hsa-miR-589-5p, hsa-miR-320d, hsa-miR-342-5p, hsa-miR-335-3p, hsa-miR-500a-3p, hsa-miR-1307-5p, hsa-miR-320c, hsa-miR-3615, hsa-miR-378i, hsa-miR-133a-3p, hsa-miR-887-3p, hsa-miR-320a-3p
TNRC6C	20	hsa-miR-320d, hsa-miR-320c, hsa-miR-148a-3p, hsa-miR-20b-5p, hsa-miR-335-5p, hsa-miR-7-5p, hsa-miR-339-5p, hsa-miR-769-5p, hsa-miR-629-5p, hsa-miR-29c-5p, hsa-miR-361-3p, hsa-miR-342-5p, hsa-miR-335-3p, hsa-miR-2110, hsa-miR-3615, hsa-miR-378i, hsa-miR-1-3p, hsa-miR-133a-3p, hsa-miR-320a-3p, hsa-miR-147b-3p
TRPS1	20	hsa-miR-1-3p, hsa-miR-148a-3p, hsa-miR-20b-5p, hsa-miR-7-5p, hsa-miR-361-5p, hsa-miR-188-5p, hsa-miR-320d, hsa-miR-629-5p, hsa-miR-342-5p, hsa-miR-335-3p, hsa-miR-425-3p, hsa-miR-500a-3p, hsa-miR-671-5p, hsa-miR-1307-3p, hsa-miR-320c, hsa-miR-378i, hsa-miR-133a-3p, hsa-miR-887-3p, hsa-miR-320a-3p, hsa-miR-147b-3p
TUBB	20	hsa-miR-320c, hsa-miR-148a-3p, hsa-miR-20b-5p, hsa-miR-335-5p, hsa-miR-7-5p, hsa-miR-138-5p, hsa-miR-361-5p, hsa-miR-339-5p, hsa-miR-574-5p, hsa-miR-769-5p, hsa-miR-335-3p, hsa-miR-500a-3p, hsa-miR-671-5p, hsa-miR-1307-3p, hsa-miR-1307-5p, hsa-miR-2110, hsa-miR-874-3p, hsa-miR-133a-3p, hsa-miR-320a-3p, hsa-miR-147b-3p
U2AF2	20	hsa-miR-671-5p, hsa-miR-148a-3p, hsa-miR-20b-5p, hsa-miR-335-5p, hsa-miR-7-5p, hsa-miR-9-3p, hsa-miR-361-5p, hsa-miR-339-5p, hsa-miR-574-5p, hsa-miR-320d, hsa-miR-1275, hsa-miR-361-3p, hsa-miR-335-3p, hsa-miR-500a-3p, hsa-miR-1307-5p, hsa-miR-378i, hsa-miR-1-3p, hsa-miR-887-3p, hsa-miR-320a-3p, hsa-miR-147b-3p
UBE2Z	20	hsa-miR-7-5p, hsa-miR-1275, hsa-miR-500a-3p, hsa-miR-671-5p, hsa-miR-148a-3p, hsa-miR-20b-5p, hsa-miR-335-5p, hsa-miR-138-5p, hsa-miR-361-5p, hsa-miR-339-5p, hsa-miR-769-5p, hsa-miR-320d, hsa-miR-629-5p, hsa-miR-29c-5p, hsa-miR-361-3p, hsa-miR-342-5p, hsa-miR-335-3p, hsa-miR-320c, hsa-miR-378i, hsa-miR-320a-3p

UBR3	20	hsa-miR-3168, hsa-miR-148a-3p, hsa-miR-20b-5p, hsa-miR-335-5p, hsa-miR-7-5p, hsa-miR-9-3p, hsa-miR-138-5p, hsa-miR-361-5p, hsa-miR-769-5p, hsa-miR-320d, hsa-miR-629-5p, hsa-miR-361-3p, hsa-miR-335-3p, hsa-miR-500a-3p, hsa-miR-320c, hsa-miR-1-3p, hsa-miR-887-3p, hsa-miR-320a-3p, hsa-miR-147b-3p, hsa-miR-190b-5p
VIRMA	20	hsa-miR-148a-3p, hsa-miR-20b-5p, hsa-miR-335-5p, hsa-miR-7-5p, hsa-miR-361-5p, hsa-miR-339-5p, hsa-miR-574-5p, hsa-miR-769-5p, hsa-miR-629-5p, hsa-miR-342-5p, hsa-miR-335-3p, hsa-miR-500a-3p, hsa-miR-671-5p, hsa-miR-1307-5p, hsa-miR-2110, hsa-miR-320c, hsa-miR-3615, hsa-miR-378i, hsa-miR-133a-3p, hsa-miR-320a-3p
VPS35	20	hsa-miR-574-5p, hsa-miR-148a-3p, hsa-miR-20b-5p, hsa-miR-335-5p, hsa-miR-7-5p, hsa-miR-138-5p, hsa-miR-361-5p, hsa-miR-589-5p, hsa-miR-769-5p, hsa-miR-320d, hsa-miR-29c-5p, hsa-miR-335-3p, hsa-miR-500a-3p, hsa-miR-1307-3p, hsa-miR-1307-5p, hsa-miR-2110, hsa-miR-320c, hsa-miR-378i, hsa-miR-320a-3p, hsa-miR-147b-3p
WASF2	20	hsa-miR-7-5p, hsa-miR-1307-3p, hsa-miR-3615, hsa-miR-1-3p, hsa-miR-20b-5p, hsa-miR-138-5p, hsa-miR-361-5p, hsa-miR-574-5p, hsa-miR-589-5p, hsa-miR-769-5p, hsa-miR-320d, hsa-miR-1275, hsa-miR-629-5p, hsa-miR-342-5p, hsa-miR-335-3p, hsa-miR-671-5p, hsa-miR-320c, hsa-miR-378i, hsa-miR-320a-3p, hsa-miR-147b-3p
XRCC5	20	hsa-miR-7-5p, hsa-miR-361-5p, hsa-miR-148a-3p, hsa-miR-20b-5p, hsa-miR-335-5p, hsa-miR-9-3p, hsa-miR-138-5p, hsa-miR-589-5p, hsa-miR-769-5p, hsa-miR-188-5p, hsa-miR-320d, hsa-miR-1275, hsa-miR-629-5p, hsa-miR-335-3p, hsa-miR-671-5p, hsa-miR-2110, hsa-miR-320c, hsa-miR-378i, hsa-miR-1-3p, hsa-miR-320a-3p
ZBTB34	20	hsa-miR-148a-3p, hsa-miR-20b-5p, hsa-miR-335-5p, hsa-miR-7-5p, hsa-miR-361-5p, hsa-miR-339-5p, hsa-miR-574-5p, hsa-miR-769-5p, hsa-miR-188-5p, hsa-miR-1275, hsa-miR-629-5p, hsa-miR-29c-5p, hsa-miR-425-3p, hsa-miR-500a-3p, hsa-miR-671-5p, hsa-miR-320c, hsa-miR-3615, hsa-miR-320a-3p, hsa-miR-147b-3p, hsa-miR-190b-5p
ZC3H12C	20	hsa-miR-20b-5p, hsa-miR-361-5p, hsa-miR-148a-3p, hsa-miR-335-5p, hsa-miR-7-5p, hsa-miR-9-3p, hsa-miR-138-5p, hsa-miR-574-5p, hsa-miR-320d, hsa-miR-629-5p, hsa-miR-361-3p, hsa-miR-342-5p, hsa-miR-335-3p, hsa-miR-500a-3p, hsa-miR-675-3p, hsa-miR-2110, hsa-miR-320c, hsa-miR-378i, hsa-miR-320a-3p, hsa-miR-147b-3p

ZFP36L1	20	hsa-miR-335-3p, hsa-miR-20b-5p, hsa-miR-335-5p, hsa-miR-7-5p, hsa-miR-138-5p, hsa-miR-574-5p, hsa-miR-320d, hsa-miR-1275, hsa-miR-629-5p, hsa-miR-342-5p, hsa-miR-425-3p, hsa-miR-1307-3p, hsa-miR-1307-5p, hsa-miR-2110, hsa-miR-320c, hsa-miR-378i, hsa-miR-874-3p, hsa-miR-1-3p, hsa-miR-320a-3p, hsa-miR-147b-3p
ABCA2	19	hsa-miR-320d, hsa-miR-320c, hsa-miR-148a-3p, hsa-miR-20b-5p, hsa-miR-335-5p, hsa-miR-7-5p, hsa-miR-138-5p, hsa-miR-361-5p, hsa-miR-339-5p, hsa-miR-574-5p, hsa-miR-500a-3p, hsa-miR-671-5p, hsa-miR-1307-3p, hsa-miR-1307-5p, hsa-miR-2110, hsa-miR-378i, hsa-miR-1-3p, hsa-miR-133a-3p, hsa-miR-320a-3p
AKAP12	19	hsa-miR-335-5p, hsa-miR-1-3p, hsa-miR-148a-3p, hsa-miR-20b-5p, hsa-miR-7-5p, hsa-miR-138-5p, hsa-miR-361-5p, hsa-miR-339-5p, hsa-miR-574-5p, hsa-miR-589-5p, hsa-miR-769-5p, hsa-miR-335-3p, hsa-miR-500a-3p, hsa-miR-671-5p, hsa-miR-320c, hsa-miR-3615, hsa-miR-874-3p, hsa-miR-133a-3p, hsa-miR-320a-3p
ARRDC3	19	hsa-miR-148a-3p, hsa-miR-335-5p, hsa-miR-20b-5p, hsa-miR-7-5p, hsa-miR-138-5p, hsa-miR-361-5p, hsa-miR-574-5p, hsa-miR-769-5p, hsa-miR-1275, hsa-miR-629-5p, hsa-miR-29c-5p, hsa-miR-335-3p, hsa-miR-425-3p, hsa-miR-671-5p, hsa-miR-675-3p, hsa-miR-320c, hsa-miR-1468-5p, hsa-miR-320a-3p, hsa-miR-147b-3p
ASXL1	19	hsa-miR-7-5p, hsa-miR-148a-3p, hsa-miR-20b-5p, hsa-miR-335-5p, hsa-miR-138-5p, hsa-miR-361-5p, hsa-miR-339-5p, hsa-miR-769-5p, hsa-miR-320d, hsa-miR-1275, hsa-miR-342-5p, hsa-miR-335-3p, hsa-miR-671-5p, hsa-miR-1307-3p, hsa-miR-1307-5p, hsa-miR-1-3p, hsa-miR-133a-3p, hsa-miR-320a-3p, hsa-miR-147b-3p
ATF7IP	19	hsa-miR-148a-3p, hsa-miR-20b-5p, hsa-miR-335-5p, hsa-miR-7-5p, hsa-miR-9-3p, hsa-miR-138-5p, hsa-miR-361-5p, hsa-miR-769-5p, hsa-miR-320d, hsa-miR-1275, hsa-miR-629-5p, hsa-miR-29c-5p, hsa-miR-335-3p, hsa-miR-500a-3p, hsa-miR-2110, hsa-miR-320c, hsa-miR-1-3p, hsa-miR-320a-3p, hsa-miR-147b-3p
BRD2	19	hsa-miR-148a-3p, hsa-miR-20b-5p, hsa-miR-335-5p, hsa-miR-7-5p, hsa-miR-138-5p, hsa-miR-361-5p, hsa-miR-574-5p, hsa-miR-589-5p, hsa-miR-188-5p, hsa-miR-361-3p, hsa-miR-342-5p, hsa-miR-335-3p, hsa-miR-500a-3p, hsa-miR-671-5p, hsa-miR-2110, hsa-miR-320c, hsa-miR-378i, hsa-miR-320a-3p, hsa-miR-147b-3p

CAMSAP2	19	hsa-miR-148a-3p, hsa-miR-20b-5p, hsa-miR-7-5p, hsa-miR-9-3p, hsa-miR-138-5p, hsa-miR-361-5p, hsa-miR-574-5p, hsa-miR-589-5p, hsa-miR-1275, hsa-miR-629-5p, hsa-miR-335-3p, hsa-miR-500a-3p, hsa-miR-671-5p, hsa-miR-2110, hsa-miR-320c, hsa-miR-3615, hsa-miR-1468-5p, hsa-miR-1-3p, hsa-miR-320a-3p
CAV1	19	hsa-miR-20b-5p, hsa-miR-7-5p, hsa-miR-4791, hsa-miR-335-5p, hsa-miR-9-3p, hsa-miR-138-5p, hsa-miR-361-5p, hsa-miR-574-5p, hsa-miR-320d, hsa-miR-29c-5p, hsa-miR-361-3p, hsa-miR-342-5p, hsa-miR-425-3p, hsa-miR-500a-3p, hsa-miR-1307-5p, hsa-miR-320c, hsa-miR-1-3p, hsa-miR-320a-3p, hsa-miR-147b-3p
CD44	19	hsa-miR-138-5p, hsa-miR-188-5p, hsa-miR-1-3p, hsa-miR-148a-3p, hsa-miR-20b-5p, hsa-miR-335-5p, hsa-miR-7-5p, hsa-miR-361-5p, hsa-miR-339-5p, hsa-miR-574-5p, hsa-miR-589-5p, hsa-miR-320d, hsa-miR-335-3p, hsa-miR-500a-3p, hsa-miR-671-5p, hsa-miR-320c, hsa-miR-378i, hsa-miR-320a-3p, hsa-miR-147b-3p
CDK13	19	hsa-miR-148a-3p, hsa-miR-20b-5p, hsa-miR-7-5p, hsa-miR-9-3p, hsa-miR-138-5p, hsa-miR-361-5p, hsa-miR-574-5p, hsa-miR-589-5p, hsa-miR-769-5p, hsa-miR-320d, hsa-miR-335-3p, hsa-miR-425-3p, hsa-miR-1307-3p, hsa-miR-320c, hsa-miR-378i, hsa-miR-1-3p, hsa-miR-887-3p, hsa-miR-320a-3p, hsa-miR-147b-3p
CERS2	19	hsa-miR-1-3p, hsa-miR-133a-3p, hsa-miR-148a-3p, hsa-miR-20b-5p, hsa-miR-335-5p, hsa-miR-7-5p, hsa-miR-9-3p, hsa-miR-138-5p, hsa-miR-361-5p, hsa-miR-339-5p, hsa-miR-574-5p, hsa-miR-320d, hsa-miR-342-5p, hsa-miR-335-3p, hsa-miR-500a-3p, hsa-miR-2110, hsa-miR-320c, hsa-miR-378i, hsa-miR-320a-3p
CNOT6	19	hsa-miR-1-3p, hsa-miR-769-5p, hsa-miR-148a-3p, hsa-miR-20b-5p, hsa-miR-335-5p, hsa-miR-7-5p, hsa-miR-9-3p, hsa-miR-361-5p, hsa-miR-339-5p, hsa-miR-589-5p, hsa-miR-320d, hsa-miR-1275, hsa-miR-335-3p, hsa-miR-425-3p, hsa-miR-500a-3p, hsa-miR-320c, hsa-miR-133a-3p, hsa-miR-320a-3p, hsa-miR-147b-3p
CSE1L	19	hsa-miR-148a-3p, hsa-miR-20b-5p, hsa-miR-335-5p, hsa-miR-7-5p, hsa-miR-138-5p, hsa-miR-361-5p, hsa-miR-574-5p, hsa-miR-769-5p, hsa-miR-320d, hsa-miR-1275, hsa-miR-425-3p, hsa-miR-500a-3p, hsa-miR-2110, hsa-miR-320c, hsa-miR-378i, hsa-miR-1-3p, hsa-miR-133a-3p, hsa-miR-320a-3p, hsa-miR-147b-3p

DAG1	19	hsa-miR-148a-3p, hsa-miR-20b-5p, hsa-miR-7-5p, hsa-miR-138-5p, hsa-miR-361-5p, hsa-miR-339-5p, hsa-miR-589-5p, hsa-miR-769-5p, hsa-miR-361-3p, hsa-miR-335-3p, hsa-miR-500a-3p, hsa-miR-671-5p, hsa-miR-1307-5p, hsa-miR-2110, hsa-miR-320c, hsa-miR-874-3p, hsa-miR-1-3p, hsa-miR-320a-3p, hsa-miR-147b-3p
DDX21	19	hsa-miR-148a-3p, hsa-miR-20b-5p, hsa-miR-7-5p, hsa-miR-138-5p, hsa-miR-361-5p, hsa-miR-339-5p, hsa-miR-574-5p, hsa-miR-769-5p, hsa-miR-320d, hsa-miR-629-5p, hsa-miR-361-3p, hsa-miR-335-3p, hsa-miR-500a-3p, hsa-miR-2110, hsa-miR-320c, hsa-miR-3615, hsa-miR-1-3p, hsa-miR-133a-3p, hsa-miR-320a-3p
DDX3X	19	hsa-miR-148a-3p, hsa-miR-20b-5p, hsa-miR-7-5p, hsa-miR-9-3p, hsa-miR-138-5p, hsa-miR-574-5p, hsa-miR-589-5p, hsa-miR-1275, hsa-miR-629-5p, hsa-miR-29c-5p, hsa-miR-342-5p, hsa-miR-500a-3p, hsa-miR-671-5p, hsa-miR-320c, hsa-miR-874-3p, hsa-miR-1-3p, hsa-miR-133a-3p, hsa-miR-320a-3p, hsa-miR-147b-3p
DLG5	19	hsa-miR-148a-3p, hsa-miR-20b-5p, hsa-miR-335-5p, hsa-miR-7-5p, hsa-miR-361-5p, hsa-miR-339-5p, hsa-miR-769-5p, hsa-miR-188-5p, hsa-miR-320d, hsa-miR-335-3p, hsa-miR-425-3p, hsa-miR-500a-3p, hsa-miR-2110, hsa-miR-320c, hsa-miR-874-3p, hsa-miR-1-3p, hsa-miR-133a-3p, hsa-miR-320a-3p, hsa-miR-147b-3p
DMXL1	19	hsa-miR-335-5p, hsa-miR-629-5p, hsa-miR-335-3p, hsa-miR-148a-3p, hsa-miR-20b-5p, hsa-miR-7-5p, hsa-miR-361-5p, hsa-miR-589-5p, hsa-miR-769-5p, hsa-miR-1275, hsa-miR-342-5p, hsa-miR-500a-3p, hsa-miR-671-5p, hsa-miR-2110, hsa-miR-320c, hsa-miR-378i, hsa-miR-1-3p, hsa-miR-887-3p, hsa-miR-320a-3p
DNAJA1	19	hsa-miR-335-5p, hsa-miR-148a-3p, hsa-miR-20b-5p, hsa-miR-7-5p, hsa-miR-9-3p, hsa-miR-138-5p, hsa-miR-361-5p, hsa-miR-339-5p, hsa-miR-589-5p, hsa-miR-629-5p, hsa-miR-335-3p, hsa-miR-500a-3p, hsa-miR-551b-3p, hsa-miR-671-5p, hsa-miR-320c, hsa-miR-3615, hsa-miR-1-3p, hsa-miR-133a-3p, hsa-miR-320a-3p
EIF3A	19	hsa-miR-148a-3p, hsa-miR-20b-5p, hsa-miR-335-5p, hsa-miR-7-5p, hsa-miR-138-5p, hsa-miR-361-5p, hsa-miR-339-5p, hsa-miR-574-5p, hsa-miR-769-5p, hsa-miR-1275, hsa-miR-29c-5p, hsa-miR-361-3p, hsa-miR-335-3p, hsa-miR-425-3p, hsa-miR-1307-3p, hsa-miR-320c, hsa-miR-1468-5p, hsa-miR-133a-3p, hsa-miR-320a-3p

EIF4A1	19	hsa-miR-361-5p, hsa-miR-148a-3p, hsa-miR-20b-5p, hsa-miR-335-5p, hsa-miR-7-5p, hsa-miR-138-5p, hsa-miR-574-5p, hsa-miR-589-5p, hsa-miR-188-5p, hsa-miR-1275, hsa-miR-342-5p, hsa-miR-335-3p, hsa-miR-500a-3p, hsa-miR-2110, hsa-miR-320c, hsa-miR-1468-5p, hsa-miR-1-3p, hsa-miR-133a-3p, hsa-miR-320a-3p
ENO1	19	hsa-miR-148a-3p, hsa-miR-20b-5p, hsa-miR-335-5p, hsa-miR-7-5p, hsa-miR-361-5p, hsa-miR-589-5p, hsa-miR-769-5p, hsa-miR-188-5p, hsa-miR-320d, hsa-miR-29c-5p, hsa-miR-361-3p, hsa-miR-342-5p, hsa-miR-425-3p, hsa-miR-500a-3p, hsa-miR-671-5p, hsa-miR-320c, hsa-miR-3615, hsa-miR-320a-3p, hsa-miR-147b-3p
FKBP4	19	hsa-miR-148a-3p, hsa-miR-20b-5p, hsa-miR-335-5p, hsa-miR-7-5p, hsa-miR-9-3p, hsa-miR-138-5p, hsa-miR-361-5p, hsa-miR-339-5p, hsa-miR-769-5p, hsa-miR-1275, hsa-miR-629-5p, hsa-miR-361-3p, hsa-miR-335-3p, hsa-miR-500a-3p, hsa-miR-320c, hsa-miR-378i, hsa-miR-1-3p, hsa-miR-133a-3p, hsa-miR-320a-3p
G3BP2	19	hsa-miR-361-5p, hsa-miR-339-5p, hsa-miR-188-5p, hsa-miR-1-3p, hsa-miR-148a-3p, hsa-miR-20b-5p, hsa-miR-7-5p, hsa-miR-138-5p, hsa-miR-574-5p, hsa-miR-589-5p, hsa-miR-320d, hsa-miR-1275, hsa-miR-342-5p, hsa-miR-335-3p, hsa-miR-2110, hsa-miR-133a-3p, hsa-miR-887-3p, hsa-miR-320a-3p, hsa-miR-147b-3p
GCN1	19	hsa-miR-425-3p, hsa-miR-20b-5p, hsa-miR-335-5p, hsa-miR-7-5p, hsa-miR-361-5p, hsa-miR-339-5p, hsa-miR-589-5p, hsa-miR-769-5p, hsa-miR-320d, hsa-miR-361-3p, hsa-miR-342-5p, hsa-miR-335-3p, hsa-miR-500a-3p, hsa-miR-671-5p, hsa-miR-2110, hsa-miR-320c, hsa-miR-378i, hsa-miR-320a-3p, hsa-miR-147b-3p
GIGYF1	19	hsa-miR-20b-5p, hsa-miR-4516, hsa-miR-148a-3p, hsa-miR-335-5p, hsa-miR-7-5p, hsa-miR-339-5p, hsa-miR-574-5p, hsa-miR-589-5p, hsa-miR-769-5p, hsa-miR-1275, hsa-miR-629-5p, hsa-miR-361-3p, hsa-miR-342-5p, hsa-miR-500a-3p, hsa-miR-3168, hsa-miR-2110, hsa-miR-3615, hsa-miR-320a-3p, hsa-miR-147b-3p
HERC2	19	hsa-miR-1-3p, hsa-miR-148a-3p, hsa-miR-20b-5p, hsa-miR-7-5p, hsa-miR-138-5p, hsa-miR-361-5p, hsa-miR-339-5p, hsa-miR-589-5p, hsa-miR-769-5p, hsa-miR-188-5p, hsa-miR-361-3p, hsa-miR-335-3p, hsa-miR-425-3p, hsa-miR-500a-3p, hsa-miR-671-5p, hsa-miR-2110, hsa-miR-320c, hsa-miR-378i, hsa-miR-320a-3p

HIF1A	19	hsa-miR-20b-5p, hsa-miR-138-5p, hsa-miR-148a-3p, hsa-miR-335-5p, hsa-miR-7-5p, hsa-miR-361-5p, hsa-miR-574-5p, hsa-miR-589-5p, hsa-miR-188-5p, hsa-miR-629-5p, hsa-miR-335-3p, hsa-miR-500a-3p, hsa-miR-1307-3p, hsa-miR-2110, hsa-miR-320c, hsa-miR-1-3p, hsa-miR-133a-3p, hsa-miR-320a-3p, hsa-miR-147b-3p
INTS1	19	hsa-miR-769-5p, hsa-miR-148a-3p, hsa-miR-20b-5p, hsa-miR-335-5p, hsa-miR-7-5p, hsa-miR-138-5p, hsa-miR-589-5p, hsa-miR-320d, hsa-miR-361-3p, hsa-miR-342-5p, hsa-miR-500a-3p, hsa-miR-1307-3p, hsa-miR-320c, hsa-miR-3615, hsa-miR-378i, hsa-miR-874-3p, hsa-miR-1-3p, hsa-miR-887-3p, hsa-miR-320a-3p
KAT6B	19	hsa-miR-335-5p, hsa-miR-148a-3p, hsa-miR-20b-5p, hsa-miR-7-5p, hsa-miR-9-3p, hsa-miR-138-5p, hsa-miR-574-5p, hsa-miR-589-5p, hsa-miR-769-5p, hsa-miR-320d, hsa-miR-629-5p, hsa-miR-335-3p, hsa-miR-500a-3p, hsa-miR-671-5p, hsa-miR-1307-5p, hsa-miR-320c, hsa-miR-133a-3p, hsa-miR-887-3p, hsa-miR-320a-3p
KDM2A	19	hsa-miR-574-5p, hsa-miR-769-5p, hsa-miR-148a-3p, hsa-miR-20b-5p, hsa-miR-335-5p, hsa-miR-7-5p, hsa-miR-138-5p, hsa-miR-361-5p, hsa-miR-339-5p, hsa-miR-320d, hsa-miR-1275, hsa-miR-29c-5p, hsa-miR-335-3p, hsa-miR-500a-3p, hsa-miR-671-5p, hsa-miR-2110, hsa-miR-1-3p, hsa-miR-133a-3p, hsa-miR-320a-3p
KDM3B	19	hsa-miR-148a-3p, hsa-miR-20b-5p, hsa-miR-335-5p, hsa-miR-7-5p, hsa-miR-339-5p, hsa-miR-574-5p, hsa-miR-589-5p, hsa-miR-769-5p, hsa-miR-320d, hsa-miR-629-5p, hsa-miR-342-5p, hsa-miR-335-3p, hsa-miR-425-3p, hsa-miR-500a-3p, hsa-miR-671-5p, hsa-miR-320c, hsa-miR-1-3p, hsa-miR-320a-3p, hsa-miR-147b-3p
KDM5A	19	hsa-miR-335-5p, hsa-miR-1307-3p, hsa-miR-148a-3p, hsa-miR-20b-5p, hsa-miR-7-5p, hsa-miR-138-5p, hsa-miR-361-5p, hsa-miR-589-5p, hsa-miR-769-5p, hsa-miR-320d, hsa-miR-1275, hsa-miR-629-5p, hsa-miR-361-3p, hsa-miR-335-3p, hsa-miR-500a-3p, hsa-miR-320c, hsa-miR-3615, hsa-miR-1-3p, hsa-miR-320a-3p
KDM6B	19	hsa-miR-148a-3p, hsa-miR-20b-5p, hsa-miR-335-5p, hsa-miR-589-5p, hsa-miR-7704, hsa-miR-7-5p, hsa-miR-138-5p, hsa-miR-769-5p, hsa-miR-1275, hsa-miR-342-5p, hsa-miR-335-3p, hsa-miR-500a-3p, hsa-miR-671-5p, hsa-miR-1307-3p, hsa-miR-320c, hsa-miR-378i, hsa-miR-1468-5p, hsa-miR-320a-3p, hsa-miR-147b-3p

KIF5B	19	hsa-miR-7-5p, hsa-miR-1-3p, hsa-miR-148a-3p, hsa-miR-20b-5p, hsa-miR-335-5p, hsa-miR-9-3p, hsa-miR-138-5p, hsa-miR-361-5p, hsa-miR-188-5p, hsa-miR-320d, hsa-miR-342-5p, hsa-miR-335-3p, hsa-miR-500a-3p, hsa-miR-671-5p, hsa-miR-320c, hsa-miR-378i, hsa-miR-1468-5p, hsa-miR-887-3p, hsa-miR-320a-3p
KMT5B	19	hsa-miR-20b-5p, hsa-miR-148a-3p, hsa-miR-335-5p, hsa-miR-7-5p, hsa-miR-138-5p, hsa-miR-361-5p, hsa-miR-574-5p, hsa-miR-769-5p, hsa-miR-629-5p, hsa-miR-361-3p, hsa-miR-335-3p, hsa-miR-500a-3p, hsa-miR-671-5p, hsa-miR-2110, hsa-miR-320c, hsa-miR-378i, hsa-miR-874-3p, hsa-miR-1-3p, hsa-miR-320a-3p
LNPEP	19	hsa-miR-148a-3p, hsa-miR-138-5p, hsa-miR-361-5p, hsa-miR-1-3p, hsa-miR-20b-5p, hsa-miR-7-5p, hsa-miR-339-5p, hsa-miR-574-5p, hsa-miR-769-5p, hsa-miR-320d, hsa-miR-1275, hsa-miR-342-5p, hsa-miR-335-3p, hsa-miR-500a-3p, hsa-miR-671-5p, hsa-miR-320c, hsa-miR-320a-3p, hsa-miR-147b-3p, hsa-miR-190b-5p
LPGAT1	19	hsa-miR-20b-5p, hsa-miR-148a-3p, hsa-miR-7-5p, hsa-miR-9-3p, hsa-miR-138-5p, hsa-miR-361-5p, hsa-miR-574-5p, hsa-miR-589-5p, hsa-miR-320d, hsa-miR-361-3p, hsa-miR-342-5p, hsa-miR-335-3p, hsa-miR-671-5p, hsa-miR-378i, hsa-miR-1468-5p, hsa-miR-1-3p, hsa-miR-133a-3p, hsa-miR-320a-3p, hsa-miR-147b-3p
LRRC8B	19	hsa-miR-1307-3p, hsa-miR-1-3p, hsa-miR-148a-3p, hsa-miR-20b-5p, hsa-miR-7-5p, hsa-miR-138-5p, hsa-miR-361-5p, hsa-miR-574-5p, hsa-miR-589-5p, hsa-miR-320d, hsa-miR-1275, hsa-miR-629-5p, hsa-miR-335-3p, hsa-miR-425-3p, hsa-miR-671-5p, hsa-miR-2110, hsa-miR-320c, hsa-miR-320a-3p, hsa-miR-147b-3p
MAPK6	19	hsa-miR-148a-3p, hsa-miR-20b-5p, hsa-miR-7-5p, hsa-miR-138-5p, hsa-miR-361-5p, hsa-miR-574-5p, hsa-miR-589-5p, hsa-miR-320d, hsa-miR-1275, hsa-miR-629-5p, hsa-miR-361-3p, hsa-miR-335-3p, hsa-miR-551b-3p, hsa-miR-671-5p, hsa-miR-2110, hsa-miR-320c, hsa-miR-378i, hsa-miR-1-3p, hsa-miR-320a-3p
MAT2A	19	hsa-miR-148a-3p, hsa-miR-20b-5p, hsa-miR-7-5p, hsa-miR-138-5p, hsa-miR-574-5p, hsa-miR-589-5p, hsa-miR-320d, hsa-miR-29c-5p, hsa-miR-342-5p, hsa-miR-335-3p, hsa-miR-500a-3p, hsa-miR-671-5p, hsa-miR-2110, hsa-miR-320c, hsa-miR-874-3p, hsa-miR-1-3p, hsa-miR-887-3p, hsa-miR-320a-3p, hsa-miR-147b-3p

MATR3	19	hsa-miR-7-5p, hsa-miR-1-3p, hsa-miR-148a-3p, hsa-miR-20b-5p, hsa-miR-138-5p, hsa-miR-361-5p, hsa-miR-339-5p, hsa-miR-589-5p, hsa-miR-769-5p, hsa-miR-629-5p, hsa-miR-335-3p, hsa-miR-425-3p, hsa-miR-500a-3p, hsa-miR-671-5p, hsa-miR-1307-5p, hsa-miR-320c, hsa-miR-3615, hsa-miR-887-3p, hsa-miR-320a-3p
MED14	19	hsa-miR-148a-3p, hsa-miR-20b-5p, hsa-miR-335-5p, hsa-miR-7-5p, hsa-miR-9-3p, hsa-miR-138-5p, hsa-miR-574-5p, hsa-miR-589-5p, hsa-miR-188-5p, hsa-miR-320d, hsa-miR-629-5p, hsa-miR-29c-5p, hsa-miR-671-5p, hsa-miR-2110, hsa-miR-320c, hsa-miR-1-3p, hsa-miR-887-3p, hsa-miR-320a-3p, hsa-miR-147b-3p
MIDN	19	hsa-miR-20b-5p, hsa-miR-335-3p, hsa-miR-2110, hsa-miR-148a-3p, hsa-miR-7-5p, hsa-miR-339-5p, hsa-miR-769-5p, hsa-miR-188-5p, hsa-miR-1275, hsa-miR-629-5p, hsa-miR-361-3p, hsa-miR-425-3p, hsa-miR-500a-3p, hsa-miR-671-5p, hsa-miR-1307-3p, hsa-miR-1307-5p, hsa-miR-1-3p, hsa-miR-320a-3p, hsa-miR-147b-3p
MLLT10	19	hsa-miR-148a-3p, hsa-miR-20b-5p, hsa-miR-335-5p, hsa-miR-7-5p, hsa-miR-9-3p, hsa-miR-138-5p, hsa-miR-361-5p, hsa-miR-574-5p, hsa-miR-320d, hsa-miR-1275, hsa-miR-629-5p, hsa-miR-361-3p, hsa-miR-335-3p, hsa-miR-2110, hsa-miR-320c, hsa-miR-1-3p, hsa-miR-133a-3p, hsa-miR-320a-3p, hsa-miR-147b-3p
MT-ND5	19	hsa-miR-148a-3p, hsa-miR-7-5p, hsa-miR-138-5p, hsa-miR-574-5p, hsa-miR-769-5p, hsa-miR-320d, hsa-miR-1275, hsa-miR-629-5p, hsa-miR-342-5p, hsa-miR-671-5p, hsa-miR-3168, hsa-miR-2110, hsa-miR-320c, hsa-miR-378i, hsa-miR-1-3p, hsa-miR-887-3p, hsa-miR-320a-3p, hsa-miR-147b-3p, hsa-miR-190b-5p
MYO18A	19	hsa-miR-335-5p, hsa-miR-1-3p, hsa-miR-148a-3p, hsa-miR-20b-5p, hsa-miR-7-5p, hsa-miR-138-5p, hsa-miR-361-5p, hsa-miR-769-5p, hsa-miR-188-5p, hsa-miR-1275, hsa-miR-629-5p, hsa-miR-29c-5p, hsa-miR-335-3p, hsa-miR-500a-3p, hsa-miR-671-5p, hsa-miR-1307-5p, hsa-miR-320c, hsa-miR-3615, hsa-miR-320a-3p
MYO9A	19	hsa-miR-148a-3p, hsa-miR-20b-5p, hsa-miR-7-5p, hsa-miR-9-3p, hsa-miR-138-5p, hsa-miR-361-5p, hsa-miR-589-5p, hsa-miR-188-5p, hsa-miR-320d, hsa-miR-629-5p, hsa-miR-342-5p, hsa-miR-335-3p, hsa-miR-671-5p, hsa-miR-2110, hsa-miR-320c, hsa-miR-3615, hsa-miR-1-3p, hsa-miR-133a-3p, hsa-miR-320a-3p

NBEA	19	hsa-miR-335-5p, hsa-miR-148a-3p, hsa-miR-20b-5p, hsa-miR-7-5p, hsa-miR-9-3p, hsa-miR-138-5p, hsa-miR-361-5p, hsa-miR-589-5p, hsa-miR-320d, hsa-miR-629-5p, hsa-miR-342-5p, hsa-miR-500a-3p, hsa-miR-671-5p, hsa-miR-2110, hsa-miR-320c, hsa-miR-378i, hsa-miR-1468-5p, hsa-miR-1-3p, hsa-miR-320a-3p
NCOA2	19	hsa-miR-148a-3p, hsa-miR-20b-5p, hsa-miR-335-5p, hsa-miR-7-5p, hsa-miR-9-3p, hsa-miR-138-5p, hsa-miR-361-5p, hsa-miR-574-5p, hsa-miR-589-5p, hsa-miR-1275, hsa-miR-629-5p, hsa-miR-335-3p, hsa-miR-500a-3p, hsa-miR-1307-3p, hsa-miR-2110, hsa-miR-320c, hsa-miR-378i, hsa-miR-320a-3p, hsa-miR-147b-3p
NCOA6	19	hsa-miR-361-5p, hsa-miR-148a-3p, hsa-miR-20b-5p, hsa-miR-335-5p, hsa-miR-7-5p, hsa-miR-138-5p, hsa-miR-769-5p, hsa-miR-29c-5p, hsa-miR-342-5p, hsa-miR-335-3p, hsa-miR-425-3p, hsa-miR-500a-3p, hsa-miR-671-5p, hsa-miR-1307-5p, hsa-miR-2110, hsa-miR-378i, hsa-miR-133a-3p, hsa-miR-320a-3p, hsa-miR-147b-3p
NFATC3	19	hsa-miR-361-3p, hsa-miR-20b-5p, hsa-miR-335-5p, hsa-miR-7-5p, hsa-miR-9-3p, hsa-miR-138-5p, hsa-miR-361-5p, hsa-miR-574-5p, hsa-miR-589-5p, hsa-miR-769-5p, hsa-miR-320d, hsa-miR-629-5p, hsa-miR-335-3p, hsa-miR-500a-3p, hsa-miR-2110, hsa-miR-320c, hsa-miR-1-3p, hsa-miR-133a-3p, hsa-miR-320a-3p
NFIB	19	hsa-miR-20b-5p, hsa-miR-339-5p, hsa-miR-335-3p, hsa-miR-148a-3p, hsa-miR-7-5p, hsa-miR-138-5p, hsa-miR-769-5p, hsa-miR-320d, hsa-miR-1275, hsa-miR-629-5p, hsa-miR-425-3p, hsa-miR-500a-3p, hsa-miR-671-5p, hsa-miR-2110, hsa-miR-320c, hsa-miR-874-3p, hsa-miR-1-3p, hsa-miR-887-3p, hsa-miR-320a-3p
NUMA1	19	hsa-miR-335-5p, hsa-miR-148a-3p, hsa-miR-20b-5p, hsa-miR-7-5p, hsa-miR-138-5p, hsa-miR-574-5p, hsa-miR-589-5p, hsa-miR-320d, hsa-miR-335-3p, hsa-miR-500a-3p, hsa-miR-671-5p, hsa-miR-2110, hsa-miR-320c, hsa-miR-3615, hsa-miR-1468-5p, hsa-miR-1-3p, hsa-miR-133a-3p, hsa-miR-320a-3p, hsa-miR-147b-3p
NUP153	19	hsa-miR-148a-3p, hsa-miR-20b-5p, hsa-miR-7-5p, hsa-miR-138-5p, hsa-miR-361-5p, hsa-miR-339-5p, hsa-miR-574-5p, hsa-miR-589-5p, hsa-miR-320d, hsa-miR-1275, hsa-miR-629-5p, hsa-miR-342-5p, hsa-miR-335-3p, hsa-miR-500a-3p, hsa-miR-671-5p, hsa-miR-675-3p, hsa-miR-378i, hsa-miR-1-3p, hsa-miR-320a-3p

PAFAH1B2	19	hsa-miR-148a-3p, hsa-miR-20b-5p, hsa-miR-7-5p, hsa-miR-9-3p, hsa-miR-138-5p, hsa-miR-361-5p, hsa-miR-574-5p, hsa-miR-769-5p, hsa-miR-320d, hsa-miR-629-5p, hsa-miR-29c-5p, hsa-miR-361-3p, hsa-miR-335-3p, hsa-miR-2110, hsa-miR-320c, hsa-miR-1-3p, hsa-miR-887-3p, hsa-miR-320a-3p, hsa-miR-147b-3p
PHF12	19	hsa-miR-148a-3p, hsa-miR-20b-5p, hsa-miR-335-5p, hsa-miR-7-5p, hsa-miR-9-3p, hsa-miR-339-5p, hsa-miR-589-5p, hsa-miR-769-5p, hsa-miR-629-5p, hsa-miR-29c-5p, hsa-miR-361-3p, hsa-miR-342-5p, hsa-miR-2110, hsa-miR-320c, hsa-miR-378i, hsa-miR-874-3p, hsa-miR-1-3p, hsa-miR-320a-3p, hsa-miR-147b-3p
PHF3	19	hsa-miR-148a-3p, hsa-miR-20b-5p, hsa-miR-7-5p, hsa-miR-138-5p, hsa-miR-361-5p, hsa-miR-339-5p, hsa-miR-574-5p, hsa-miR-188-5p, hsa-miR-629-5p, hsa-miR-29c-5p, hsa-miR-335-3p, hsa-miR-500a-3p, hsa-miR-671-5p, hsa-miR-2110, hsa-miR-320c, hsa-miR-378i, hsa-miR-1-3p, hsa-miR-320a-3p, hsa-miR-147b-3p
PPP2CA	19	hsa-miR-188-5p, hsa-miR-671-5p, hsa-miR-133a-3p, hsa-miR-148a-3p, hsa-miR-20b-5p, hsa-miR-335-5p, hsa-miR-7-5p, hsa-miR-138-5p, hsa-miR-361-5p, hsa-miR-574-5p, hsa-miR-589-5p, hsa-miR-320d, hsa-miR-29c-5p, hsa-miR-342-5p, hsa-miR-335-3p, hsa-miR-2110, hsa-miR-320c, hsa-miR-3615, hsa-miR-320a-3p
PRKAA1	19	hsa-miR-675-3p, hsa-miR-148a-3p, hsa-miR-20b-5p, hsa-miR-7-5p, hsa-miR-9-3p, hsa-miR-339-5p, hsa-miR-589-5p, hsa-miR-769-5p, hsa-miR-320d, hsa-miR-629-5p, hsa-miR-361-3p, hsa-miR-335-3p, hsa-miR-425-3p, hsa-miR-671-5p, hsa-miR-3168, hsa-miR-320c, hsa-miR-1-3p, hsa-miR-320a-3p, hsa-miR-147b-3p
PSMD11	19	hsa-miR-4516, hsa-miR-148a-3p, hsa-miR-20b-5p, hsa-miR-335-5p, hsa-miR-9-3p, hsa-miR-138-5p, hsa-miR-361-5p, hsa-miR-339-5p, hsa-miR-574-5p, hsa-miR-589-5p, hsa-miR-320d, hsa-miR-29c-5p, hsa-miR-361-3p, hsa-miR-335-3p, hsa-miR-500a-3p, hsa-miR-320c, hsa-miR-1-3p, hsa-miR-320a-3p, hsa-miR-147b-3p
PTEN	19	hsa-miR-148a-3p, hsa-miR-20b-5p, hsa-miR-29c-5p, hsa-miR-500a-3p, hsa-miR-4791, hsa-miR-335-5p, hsa-miR-7-5p, hsa-miR-9-3p, hsa-miR-138-5p, hsa-miR-574-5p, hsa-miR-188-5p, hsa-miR-320d, hsa-miR-425-3p, hsa-miR-671-5p, hsa-miR-320c, hsa-miR-1-3p, hsa-miR-133a-3p, hsa-miR-320a-3p, hsa-miR-147b-3p

PTPRK	19	hsa-miR-148a-3p, hsa-miR-20b-5p, hsa-miR-335-5p, hsa-miR-7-5p, hsa-miR-138-5p, hsa-miR-361-5p, hsa-miR-574-5p, hsa-miR-589-5p, hsa-miR-769-5p, hsa-miR-320d, hsa-miR-361-3p, hsa-miR-335-3p, hsa-miR-500a-3p, hsa-miR-671-5p, hsa-miR-320c, hsa-miR-378i, hsa-miR-1-3p, hsa-miR-133a-3p, hsa-miR-320a-3p
RBM12	19	hsa-miR-20b-5p, hsa-miR-335-5p, hsa-miR-7-5p, hsa-miR-9-3p, hsa-miR-138-5p, hsa-miR-589-5p, hsa-miR-769-5p, hsa-miR-1275, hsa-miR-629-5p, hsa-miR-342-5p, hsa-miR-335-3p, hsa-miR-425-3p, hsa-miR-500a-3p, hsa-miR-671-5p, hsa-miR-320c, hsa-miR-378i, hsa-miR-1-3p, hsa-miR-133a-3p, hsa-miR-320a-3p
SAMD4B	19	hsa-miR-148a-3p, hsa-miR-20b-5p, hsa-miR-7-5p, hsa-miR-138-5p, hsa-miR-361-5p, hsa-miR-574-5p, hsa-miR-589-5p, hsa-miR-769-5p, hsa-miR-320d, hsa-miR-629-5p, hsa-miR-361-3p, hsa-miR-425-3p, hsa-miR-671-5p, hsa-miR-1307-5p, hsa-miR-2110, hsa-miR-320c, hsa-miR-874-3p, hsa-miR-1-3p, hsa-miR-320a-3p
SEC61A1	19	hsa-miR-335-5p, hsa-miR-589-5p, hsa-miR-1-3p, hsa-miR-148a-3p, hsa-miR-20b-5p, hsa-miR-7-5p, hsa-miR-138-5p, hsa-miR-361-5p, hsa-miR-574-5p, hsa-miR-769-5p, hsa-miR-320d, hsa-miR-629-5p, hsa-miR-335-3p, hsa-miR-500a-3p, hsa-miR-671-5p, hsa-miR-2110, hsa-miR-378i, hsa-miR-320a-3p, hsa-miR-147b-3p
SEMA7A	19	hsa-miR-20b-5p, hsa-miR-320d, hsa-miR-320c, hsa-miR-148a-3p, hsa-miR-335-5p, hsa-miR-7-5p, hsa-miR-9-3p, hsa-miR-138-5p, hsa-miR-574-5p, hsa-miR-589-5p, hsa-miR-769-5p, hsa-miR-1275, hsa-miR-629-5p, hsa-miR-342-5p, hsa-miR-335-3p, hsa-miR-500a-3p, hsa-miR-3615, hsa-miR-133a-3p, hsa-miR-320a-3p
SESN3	19	hsa-miR-148a-3p, hsa-miR-20b-5p, hsa-miR-335-5p, hsa-miR-133a-3p, hsa-miR-7-5p, hsa-miR-9-3p, hsa-miR-361-5p, hsa-miR-589-5p, hsa-miR-769-5p, hsa-miR-320d, hsa-miR-1275, hsa-miR-335-3p, hsa-miR-425-3p, hsa-miR-500a-3p, hsa-miR-671-5p, hsa-miR-1307-3p, hsa-miR-320c, hsa-miR-1-3p, hsa-miR-320a-3p
SETD1B	19	hsa-miR-7-5p, hsa-miR-1275, hsa-miR-342-5p, hsa-miR-148a-3p, hsa-miR-20b-5p, hsa-miR-335-5p, hsa-miR-361-5p, hsa-miR-339-5p, hsa-miR-589-5p, hsa-miR-769-5p, hsa-miR-320d, hsa-miR-361-3p, hsa-miR-335-3p, hsa-miR-500a-3p, hsa-miR-671-5p, hsa-miR-1307-5p, hsa-miR-320c, hsa-miR-320a-3p, hsa-miR-147b-3p

SMC1A	19	hsa-miR-339-5p, hsa-miR-342-5p, hsa-miR-148a-3p, hsa-miR-20b-5p, hsa-miR-7-5p, hsa-miR-361-5p, hsa-miR-574-5p, hsa-miR-589-5p, hsa-miR-320d, hsa-miR-335-3p, hsa-miR-500a-3p, hsa-miR-671-5p, hsa-miR-1307-5p, hsa-miR-2110, hsa-miR-320c, hsa-miR-3615, hsa-miR-1-3p, hsa-miR-133a-3p, hsa-miR-320a-3p
STAU1	19	hsa-miR-148a-3p, hsa-miR-20b-5p, hsa-miR-335-5p, hsa-miR-7-5p, hsa-miR-361-5p, hsa-miR-574-5p, hsa-miR-320d, hsa-miR-629-5p, hsa-miR-361-3p, hsa-miR-342-5p, hsa-miR-335-3p, hsa-miR-671-5p, hsa-miR-2110, hsa-miR-320c, hsa-miR-3615, hsa-miR-378i, hsa-miR-1-3p, hsa-miR-133a-3p, hsa-miR-320a-3p
SUPT16H	19	hsa-miR-361-5p, hsa-miR-339-5p, hsa-miR-133a-3p, hsa-miR-148a-3p, hsa-miR-20b-5p, hsa-miR-335-5p, hsa-miR-7-5p, hsa-miR-138-5p, hsa-miR-589-5p, hsa-miR-1275, hsa-miR-629-5p, hsa-miR-342-5p, hsa-miR-335-3p, hsa-miR-551b-3p, hsa-miR-320c, hsa-miR-378i, hsa-miR-1-3p, hsa-miR-320a-3p, hsa-miR-147b-3p
SYNCRIP	19	hsa-miR-148a-3p, hsa-miR-20b-5p, hsa-miR-335-5p, hsa-miR-7-5p, hsa-miR-138-5p, hsa-miR-361-5p, hsa-miR-589-5p, hsa-miR-188-5p, hsa-miR-1275, hsa-miR-342-5p, hsa-miR-335-3p, hsa-miR-500a-3p, hsa-miR-671-5p, hsa-miR-2110, hsa-miR-320c, hsa-miR-3615, hsa-miR-1-3p, hsa-miR-887-3p, hsa-miR-320a-3p
TAB2	19	hsa-miR-20b-5p, hsa-miR-7-5p, hsa-miR-9-3p, hsa-miR-361-5p, hsa-miR-769-5p, hsa-miR-320d, hsa-miR-1275, hsa-miR-629-5p, hsa-miR-335-3p, hsa-miR-425-3p, hsa-miR-500a-3p, hsa-miR-671-5p, hsa-miR-1307-3p, hsa-miR-2110, hsa-miR-320c, hsa-miR-378i, hsa-miR-1-3p, hsa-miR-320a-3p, hsa-miR-190b-5p
TCF20	19	hsa-miR-188-5p, hsa-miR-148a-3p, hsa-miR-20b-5p, hsa-miR-335-5p, hsa-miR-7-5p, hsa-miR-574-5p, hsa-miR-589-5p, hsa-miR-769-5p, hsa-miR-29c-5p, hsa-miR-361-3p, hsa-miR-342-5p, hsa-miR-335-3p, hsa-miR-425-3p, hsa-miR-500a-3p, hsa-miR-320c, hsa-miR-3615, hsa-miR-378i, hsa-miR-320a-3p, hsa-miR-147b-3p
TRIM2	19	hsa-miR-1-3p, hsa-miR-335-5p, hsa-miR-574-5p, hsa-miR-671-5p, hsa-miR-148a-3p, hsa-miR-20b-5p, hsa-miR-7-5p, hsa-miR-138-5p, hsa-miR-361-5p, hsa-miR-589-5p, hsa-miR-188-5p, hsa-miR-29c-5p, hsa-miR-335-3p, hsa-miR-500a-3p, hsa-miR-675-3p, hsa-miR-320c, hsa-miR-874-3p, hsa-miR-887-3p, hsa-miR-320a-3p

UBP1	19	hsa-miR-20b-5p, hsa-miR-7-5p, hsa-miR-339-5p, hsa-miR-589-5p, hsa-miR-769-5p, hsa-miR-188-5p, hsa-miR-1275, hsa-miR-629-5p, hsa-miR-335-3p, hsa-miR-500a-3p, hsa-miR-671-5p, hsa-miR-1307-5p, hsa-miR-2110, hsa-miR-320c, hsa-miR-3615, hsa-miR-378i, hsa-miR-1-3p, hsa-miR-133a-3p, hsa-miR-320a-3p
USP47	19	hsa-miR-148a-3p, hsa-miR-20b-5p, hsa-miR-335-5p, hsa-miR-7-5p, hsa-miR-138-5p, hsa-miR-361-5p, hsa-miR-188-5p, hsa-miR-320d, hsa-miR-629-5p, hsa-miR-29c-5p, hsa-miR-361-3p, hsa-miR-425-3p, hsa-miR-500a-3p, hsa-miR-671-5p, hsa-miR-1307-3p, hsa-miR-2110, hsa-miR-320c, hsa-miR-320a-3p, hsa-miR-190b-5p
VEGFA	19	hsa-miR-20b-5p, hsa-miR-335-5p, hsa-miR-361-5p, hsa-miR-4497, hsa-miR-1-3p, hsa-miR-133a-3p, hsa-miR-148a-3p, hsa-miR-7-5p, hsa-miR-9-3p, hsa-miR-138-5p, hsa-miR-339-5p, hsa-miR-574-5p, hsa-miR-335-3p, hsa-miR-425-3p, hsa-miR-500a-3p, hsa-miR-1307-5p, hsa-miR-3168, hsa-miR-320a-3p, hsa-miR-147b-3p
WEE1	19	hsa-miR-20b-5p, hsa-miR-1-3p, hsa-miR-148a-3p, hsa-miR-335-5p, hsa-miR-7-5p, hsa-miR-138-5p, hsa-miR-361-5p, hsa-miR-339-5p, hsa-miR-574-5p, hsa-miR-589-5p, hsa-miR-320d, hsa-miR-629-5p, hsa-miR-342-5p, hsa-miR-425-3p, hsa-miR-500a-3p, hsa-miR-2110, hsa-miR-320c, hsa-miR-378i, hsa-miR-320a-3p
ZBTB18	19	hsa-miR-20b-5p, hsa-miR-361-5p, hsa-miR-629-5p, hsa-miR-148a-3p, hsa-miR-7-5p, hsa-miR-9-3p, hsa-miR-138-5p, hsa-miR-589-5p, hsa-miR-188-5p, hsa-miR-320d, hsa-miR-1275, hsa-miR-29c-5p, hsa-miR-335-3p, hsa-miR-500a-3p, hsa-miR-671-5p, hsa-miR-320c, hsa-miR-1-3p, hsa-miR-320a-3p, hsa-miR-147b-3p
ZFHX4	19	hsa-miR-148a-3p, hsa-miR-20b-5p, hsa-miR-335-5p, hsa-miR-7-5p, hsa-miR-138-5p, hsa-miR-361-5p, hsa-miR-339-5p, hsa-miR-589-5p, hsa-miR-769-5p, hsa-miR-188-5p, hsa-miR-320d, hsa-miR-335-3p, hsa-miR-500a-3p, hsa-miR-671-5p, hsa-miR-1307-3p, hsa-miR-320c, hsa-miR-378i, hsa-miR-1-3p, hsa-miR-320a-3p
ZFP36L2	19	hsa-miR-671-5p, hsa-miR-20b-5p, hsa-miR-335-5p, hsa-miR-7-5p, hsa-miR-138-5p, hsa-miR-574-5p, hsa-miR-589-5p, hsa-miR-769-5p, hsa-miR-320d, hsa-miR-361-3p, hsa-miR-335-3p, hsa-miR-500a-3p, hsa-miR-1307-3p, hsa-miR-2110, hsa-miR-320c, hsa-miR-874-3p, hsa-miR-1-3p, hsa-miR-887-3p, hsa-miR-320a-3p

ZNF217	19	hsa-miR-148a-3p, hsa-miR-20b-5p, hsa-miR-335-5p, hsa-miR-138-5p, hsa-miR-574-5p, hsa-miR-589-5p, hsa-miR-769-5p, hsa-miR-629-5p, hsa-miR-342-5p, hsa-miR-335-3p, hsa-miR-671-5p, hsa-miR-1307-3p, hsa-miR-675-3p, hsa-miR-2110, hsa-miR-320c, hsa-miR-1-3p, hsa-miR-887-3p, hsa-miR-320a-3p, hsa-miR-147b-3p
ZNF609	19	hsa-miR-335-5p, hsa-miR-378i, hsa-miR-148a-3p, hsa-miR-20b-5p, hsa-miR-7-5p, hsa-miR-138-5p, hsa-miR-361-5p, hsa-miR-574-5p, hsa-miR-769-5p, hsa-miR-1275, hsa-miR-29c-5p, hsa-miR-361-3p, hsa-miR-342-5p, hsa-miR-1307-5p, hsa-miR-2110, hsa-miR-320c, hsa-miR-1-3p, hsa-miR-320a-3p, hsa-miR-147b-3p
	18	hsa-miR-20b-5p, hsa-miR-335-5p, hsa-miR-7-5p, hsa-miR-9-3p, hsa-miR-138-5p, hsa-miR-339-5p, hsa-miR-574-5p, hsa-miR-769-5p, hsa-miR-188-5p, hsa-miR-320d, hsa-miR-671-5p, hsa-miR-1307-3p, hsa-miR-675-3p, hsa-miR-320c, hsa-miR-4791, hsa-miR-1468-5p, hsa-miR-1-3p, hsa-miR-133a-3p
ACACA	18	hsa-miR-335-5p, hsa-miR-339-5p, hsa-miR-361-3p, hsa-miR-378i, hsa-miR-148a-3p, hsa-miR-7-5p, hsa-miR-138-5p, hsa-miR-361-5p, hsa-miR-574-5p, hsa-miR-769-5p, hsa-miR-342-5p, hsa-miR-335-3p, hsa-miR-500a-3p, hsa-miR-2110, hsa-miR-1-3p, hsa-miR-133a-3p, hsa-miR-320a-3p, hsa-miR-147b-3p
AKAP11	18	hsa-miR-20b-5p, hsa-miR-7-5p, hsa-miR-148a-3p, hsa-miR-335-5p, hsa-miR-9-3p, hsa-miR-138-5p, hsa-miR-361-5p, hsa-miR-589-5p, hsa-miR-1275, hsa-miR-335-3p, hsa-miR-500a-3p, hsa-miR-551b-3p, hsa-miR-2110, hsa-miR-320c, hsa-miR-1-3p, hsa-miR-133a-3p, hsa-miR-887-3p, hsa-miR-320a-3p
ANKIB1	18	hsa-miR-1-3p, hsa-miR-20b-5p, hsa-miR-335-5p, hsa-miR-148a-3p, hsa-miR-7-5p, hsa-miR-138-5p, hsa-miR-361-5p, hsa-miR-574-5p, hsa-miR-320d, hsa-miR-629-5p, hsa-miR-335-3p, hsa-miR-500a-3p, hsa-miR-671-5p, hsa-miR-2110, hsa-miR-320c, hsa-miR-378i, hsa-miR-320a-3p, hsa-miR-147b-3p
ANKRD50	18	hsa-miR-20b-5p, hsa-miR-551b-3p, hsa-miR-148a-3p, hsa-miR-7-5p, hsa-miR-9-3p, hsa-miR-138-5p, hsa-miR-361-5p, hsa-miR-339-5p, hsa-miR-574-5p, hsa-miR-629-5p, hsa-miR-342-5p, hsa-miR-335-3p, hsa-miR-671-5p, hsa-miR-320c, hsa-miR-1-3p, hsa-miR-133a-3p, hsa-miR-320a-3p, hsa-miR-147b-3p

ANKRD52	18	hsa-miR-20b-5p, hsa-miR-339-5p, hsa-miR-4516, hsa-miR-148a-3p, hsa-miR-335-5p, hsa-miR-7-5p, hsa-miR-138-5p, hsa-miR-361-5p, hsa-miR-574-5p, hsa-miR-1275, hsa-miR-342-5p, hsa-miR-425-3p, hsa-miR-671-5p, hsa-miR-2110, hsa-miR-320c, hsa-miR-378i, hsa-miR-320a-3p, hsa-miR-147b-3p
ANXA11	18	hsa-miR-335-5p, hsa-miR-7-5p, hsa-miR-148a-3p, hsa-miR-20b-5p, hsa-miR-138-5p, hsa-miR-361-5p, hsa-miR-339-5p, hsa-miR-1275, hsa-miR-361-3p, hsa-miR-342-5p, hsa-miR-500a-3p, hsa-miR-671-5p, hsa-miR-320c, hsa-miR-378i, hsa-miR-874-3p, hsa-miR-1-3p, hsa-miR-320a-3p, hsa-miR-147b-3p
AP3M1	18	hsa-miR-7-5p, hsa-miR-148a-3p, hsa-miR-20b-5p, hsa-miR-335-5p, hsa-miR-9-3p, hsa-miR-138-5p, hsa-miR-320d, hsa-miR-361-3p, hsa-miR-425-3p, hsa-miR-500a-3p, hsa-miR-671-5p, hsa-miR-2110, hsa-miR-320c, hsa-miR-378i, hsa-miR-1-3p, hsa-miR-133a-3p, hsa-miR-320a-3p, hsa-miR-147b-3p
APP	18	hsa-miR-148a-3p, hsa-miR-20b-5p, hsa-miR-335-5p, hsa-miR-7-5p, hsa-miR-9-3p, hsa-miR-138-5p, hsa-miR-361-5p, hsa-miR-574-5p, hsa-miR-769-5p, hsa-miR-629-5p, hsa-miR-361-3p, hsa-miR-342-5p, hsa-miR-335-3p, hsa-miR-425-3p, hsa-miR-671-5p, hsa-miR-378i, hsa-miR-320a-3p, hsa-miR-147b-3p
ARHGAP35	18	hsa-miR-20b-5p, hsa-miR-574-5p, hsa-miR-148a-3p, hsa-miR-7-5p, hsa-miR-138-5p, hsa-miR-361-5p, hsa-miR-339-5p, hsa-miR-769-5p, hsa-miR-629-5p, hsa-miR-361-3p, hsa-miR-335-3p, hsa-miR-500a-3p, hsa-miR-2110, hsa-miR-320c, hsa-miR-3615, hsa-miR-1-3p, hsa-miR-320a-3p, hsa-miR-147b-3p
ARHGAP5	18	hsa-miR-335-5p, hsa-miR-148a-3p, hsa-miR-20b-5p, hsa-miR-7-5p, hsa-miR-9-3p, hsa-miR-138-5p, hsa-miR-361-5p, hsa-miR-589-5p, hsa-miR-769-5p, hsa-miR-320d, hsa-miR-629-5p, hsa-miR-335-3p, hsa-miR-425-3p, hsa-miR-671-5p, hsa-miR-320c, hsa-miR-3615, hsa-miR-1468-5p, hsa-miR-320a-3p
ARHGEF12	18	hsa-miR-7-5p, hsa-miR-148a-3p, hsa-miR-20b-5p, hsa-miR-335-5p, hsa-miR-138-5p, hsa-miR-361-5p, hsa-miR-574-5p, hsa-miR-589-5p, hsa-miR-29c-5p, hsa-miR-342-5p, hsa-miR-335-3p, hsa-miR-2110, hsa-miR-320c, hsa-miR-3615, hsa-miR-874-3p, hsa-miR-1-3p, hsa-miR-320a-3p, hsa-miR-147b-3p

ATL3	18	hsa-miR-20b-5p, hsa-miR-1-3p, hsa-miR-148a-3p, hsa-miR-335-5p, hsa-miR-7-5p, hsa-miR-361-5p, hsa-miR-574-5p, hsa-miR-589-5p, hsa-miR-769-5p, hsa-miR-320d, hsa-miR-1275, hsa-miR-361-3p, hsa-miR-335-3p, hsa-miR-671-5p, hsa-miR-320c, hsa-miR-887-3p, hsa-miR-320a-3p, hsa-miR-147b-3p
ATP2B1	18	hsa-miR-20b-5p, hsa-miR-148a-3p, hsa-miR-335-5p, hsa-miR-7-5p, hsa-miR-138-5p, hsa-miR-339-5p, hsa-miR-574-5p, hsa-miR-769-5p, hsa-miR-320d, hsa-miR-629-5p, hsa-miR-29c-5p, hsa-miR-500a-3p, hsa-miR-671-5p, hsa-miR-2110, hsa-miR-320c, hsa-miR-1-3p, hsa-miR-133a-3p, hsa-miR-320a-3p
ATP6V1A	18	hsa-miR-361-5p, hsa-miR-1-3p, hsa-miR-148a-3p, hsa-miR-20b-5p, hsa-miR-7-5p, hsa-miR-138-5p, hsa-miR-1275, hsa-miR-629-5p, hsa-miR-29c-5p, hsa-miR-342-5p, hsa-miR-335-3p, hsa-miR-500a-3p, hsa-miR-671-5p, hsa-miR-320c, hsa-miR-3615, hsa-miR-378i, hsa-miR-133a-3p, hsa-miR-320a-3p
BPTF	18	hsa-miR-335-5p, hsa-miR-148a-3p, hsa-miR-20b-5p, hsa-miR-7-5p, hsa-miR-138-5p, hsa-miR-361-5p, hsa-miR-339-5p, hsa-miR-589-5p, hsa-miR-335-3p, hsa-miR-425-3p, hsa-miR-500a-3p, hsa-miR-1307-3p, hsa-miR-2110, hsa-miR-378i, hsa-miR-1-3p, hsa-miR-133a-3p, hsa-miR-320a-3p, hsa-miR-147b-3p
BTG2	18	hsa-miR-20b-5p, hsa-miR-7-5p, hsa-miR-339-5p, hsa-miR-148a-3p, hsa-miR-138-5p, hsa-miR-589-5p, hsa-miR-769-5p, hsa-miR-320d, hsa-miR-629-5p, hsa-miR-361-3p, hsa-miR-335-3p, hsa-miR-425-3p, hsa-miR-500a-3p, hsa-miR-551b-3p, hsa-miR-671-5p, hsa-miR-1307-3p, hsa-miR-320c, hsa-miR-320a-3p
CDH1	18	hsa-miR-9-3p, hsa-miR-138-5p, hsa-miR-148a-3p, hsa-miR-20b-5p, hsa-miR-335-5p, hsa-miR-7-5p, hsa-miR-361-5p, hsa-miR-589-5p, hsa-miR-769-5p, hsa-miR-320d, hsa-miR-335-3p, hsa-miR-425-3p, hsa-miR-500a-3p, hsa-miR-1307-5p, hsa-miR-2110, hsa-miR-320c, hsa-miR-320a-3p, hsa-miR-147b-3p
CHD3	18	hsa-miR-148a-3p, hsa-miR-20b-5p, hsa-miR-335-5p, hsa-miR-7-5p, hsa-miR-138-5p, hsa-miR-339-5p, hsa-miR-589-5p, hsa-miR-769-5p, hsa-miR-361-3p, hsa-miR-335-3p, hsa-miR-500a-3p, hsa-miR-671-5p, hsa-miR-1307-3p, hsa-miR-2110, hsa-miR-3615, hsa-miR-378i, hsa-miR-1-3p, hsa-miR-320a-3p

CHD8	18	hsa-miR-148a-3p, hsa-miR-20b-5p, hsa-miR-335-5p, hsa-miR-7-5p, hsa-miR-138-5p, hsa-miR-361-5p, hsa-miR-589-5p, hsa-miR-769-5p, hsa-miR-629-5p, hsa-miR-361-3p, hsa-miR-335-3p, hsa-miR-500a-3p, hsa-miR-2110, hsa-miR-320c, hsa-miR-378i, hsa-miR-1-3p, hsa-miR-320a-3p, hsa-miR-147b-3p
CPEB3	18	hsa-miR-148a-3p, hsa-miR-20b-5p, hsa-miR-7-5p, hsa-miR-9-3p, hsa-miR-138-5p, hsa-miR-361-5p, hsa-miR-589-5p, hsa-miR-1275, hsa-miR-29c-5p, hsa-miR-361-3p, hsa-miR-335-3p, hsa-miR-425-3p, hsa-miR-500a-3p, hsa-miR-551b-3p, hsa-miR-671-5p, hsa-miR-320c, hsa-miR-1-3p, hsa-miR-320a-3p
CREB1	18	hsa-miR-20b-5p, hsa-miR-1275, hsa-miR-148a-3p, hsa-miR-335-5p, hsa-miR-7-5p, hsa-miR-361-5p, hsa-miR-339-5p, hsa-miR-574-5p, hsa-miR-769-5p, hsa-miR-342-5p, hsa-miR-335-3p, hsa-miR-500a-3p, hsa-miR-1307-3p, hsa-miR-2110, hsa-miR-320c, hsa-miR-1-3p, hsa-miR-320a-3p, hsa-miR-190b-5p
CSNK1E	18	hsa-miR-629-5p, hsa-miR-148a-3p, hsa-miR-20b-5p, hsa-miR-335-5p, hsa-miR-7-5p, hsa-miR-138-5p, hsa-miR-574-5p, hsa-miR-769-5p, hsa-miR-361-3p, hsa-miR-425-3p, hsa-miR-500a-3p, hsa-miR-671-5p, hsa-miR-1307-3p, hsa-miR-2110, hsa-miR-874-3p, hsa-miR-1-3p, hsa-miR-133a-3p, hsa-miR-320a-3p
CTNNA1	18	hsa-miR-148a-3p, hsa-miR-335-5p, hsa-miR-7-5p, hsa-miR-138-5p, hsa-miR-361-5p, hsa-miR-339-5p, hsa-miR-589-5p, hsa-miR-769-5p, hsa-miR-629-5p, hsa-miR-335-3p, hsa-miR-500a-3p, hsa-miR-671-5p, hsa-miR-3168, hsa-miR-2110, hsa-miR-378i, hsa-miR-874-3p, hsa-miR-320a-3p, hsa-miR-147b-3p
CUX1	18	hsa-miR-148a-3p, hsa-miR-20b-5p, hsa-miR-335-5p, hsa-miR-7-5p, hsa-miR-9-3p, hsa-miR-138-5p, hsa-miR-574-5p, hsa-miR-589-5p, hsa-miR-320d, hsa-miR-629-5p, hsa-miR-342-5p, hsa-miR-335-3p, hsa-miR-500a-3p, hsa-miR-2110, hsa-miR-320c, hsa-miR-378i, hsa-miR-320a-3p, hsa-miR-147b-3p
CYFIP2	18	hsa-miR-335-3p, hsa-miR-148a-3p, hsa-miR-20b-5p, hsa-miR-7-5p, hsa-miR-9-3p, hsa-miR-138-5p, hsa-miR-361-5p, hsa-miR-339-5p, hsa-miR-769-5p, hsa-miR-188-5p, hsa-miR-342-5p, hsa-miR-500a-3p, hsa-miR-671-5p, hsa-miR-2110, hsa-miR-320c, hsa-miR-1-3p, hsa-miR-133a-3p, hsa-miR-320a-3p

DDHD1	18	hsa-miR-20b-5p, hsa-miR-574-5p, hsa-miR-148a-3p, hsa-miR-335-5p, hsa-miR-7-5p, hsa-miR-361-5p, hsa-miR-589-5p, hsa-miR-188-5p, hsa-miR-320d, hsa-miR-1275, hsa-miR-29c-5p, hsa-miR-335-3p, hsa-miR-671-5p, hsa-miR-2110, hsa-miR-320c, hsa-miR-1-3p, hsa-miR-133a-3p, hsa-miR-320a-3p
DDX5	18	hsa-miR-1-3p, hsa-miR-20b-5p, hsa-miR-148a-3p, hsa-miR-335-5p, hsa-miR-7-5p, hsa-miR-138-5p, hsa-miR-339-5p, hsa-miR-574-5p, hsa-miR-769-5p, hsa-miR-361-3p, hsa-miR-342-5p, hsa-miR-335-3p, hsa-miR-425-3p, hsa-miR-500a-3p, hsa-miR-675-3p, hsa-miR-3615, hsa-miR-320a-3p, hsa-miR-147b-3p
DLG1	18	hsa-miR-9-3p, hsa-miR-7-5p, hsa-miR-138-5p, hsa-miR-361-5p, hsa-miR-574-5p, hsa-miR-589-5p, hsa-miR-769-5p, hsa-miR-629-5p, hsa-miR-342-5p, hsa-miR-335-3p, hsa-miR-425-3p, hsa-miR-500a-3p, hsa-miR-671-5p, hsa-miR-2110, hsa-miR-320c, hsa-miR-1-3p, hsa-miR-320a-3p, hsa-miR-147b-3p
DMXL2	18	hsa-miR-148a-3p, hsa-miR-20b-5p, hsa-miR-7-5p, hsa-miR-138-5p, hsa-miR-361-5p, hsa-miR-574-5p, hsa-miR-769-5p, hsa-miR-188-5p, hsa-miR-342-5p, hsa-miR-335-3p, hsa-miR-500a-3p, hsa-miR-551b-3p, hsa-miR-671-5p, hsa-miR-2110, hsa-miR-320c, hsa-miR-1-3p, hsa-miR-887-3p, hsa-miR-320a-3p
E2F7	18	hsa-miR-148a-3p, hsa-miR-20b-5p, hsa-miR-335-5p, hsa-miR-7-5p, hsa-miR-9-3p, hsa-miR-361-5p, hsa-miR-589-5p, hsa-miR-320d, hsa-miR-1275, hsa-miR-335-3p, hsa-miR-500a-3p, hsa-miR-671-5p, hsa-miR-320c, hsa-miR-378i, hsa-miR-874-3p, hsa-miR-1-3p, hsa-miR-887-3p, hsa-miR-320a-3p
EGFR	18	hsa-miR-7-5p, hsa-miR-335-5p, hsa-miR-9-3p, hsa-miR-138-5p, hsa-miR-1-3p, hsa-miR-133a-3p, hsa-miR-148a-3p, hsa-miR-20b-5p, hsa-miR-361-5p, hsa-miR-574-5p, hsa-miR-769-5p, hsa-miR-320d, hsa-miR-335-3p, hsa-miR-500a-3p, hsa-miR-671-5p, hsa-miR-320c, hsa-miR-378i, hsa-miR-320a-3p
ELK4	18	hsa-miR-20b-5p, hsa-miR-335-3p, hsa-miR-148a-3p, hsa-miR-7-5p, hsa-miR-574-5p, hsa-miR-589-5p, hsa-miR-769-5p, hsa-miR-320d, hsa-miR-629-5p, hsa-miR-29c-5p, hsa-miR-361-3p, hsa-miR-551b-3p, hsa-miR-671-5p, hsa-miR-1307-3p, hsa-miR-2110, hsa-miR-320c, hsa-miR-133a-3p, hsa-miR-320a-3p

FBN1	18	hsa-miR-1-3p, hsa-miR-133a-3p, hsa-miR-148a-3p, hsa-miR-20b-5p, hsa-miR-7-5p, hsa-miR-138-5p, hsa-miR-574-5p, hsa-miR-589-5p, hsa-miR-769-5p, hsa-miR-1275, hsa-miR-29c-5p, hsa-miR-342-5p, hsa-miR-500a-3p, hsa-miR-671-5p, hsa-miR-320c, hsa-miR-378i, hsa-miR-1468-5p, hsa-miR-320a-3p
FLNB	18	hsa-miR-1-3p, hsa-miR-148a-3p, hsa-miR-20b-5p, hsa-miR-335-5p, hsa-miR-7-5p, hsa-miR-138-5p, hsa-miR-361-5p, hsa-miR-339-5p, hsa-miR-574-5p, hsa-miR-769-5p, hsa-miR-342-5p, hsa-miR-335-3p, hsa-miR-500a-3p, hsa-miR-3615, hsa-miR-378i, hsa-miR-320a-3p, hsa-miR-147b-3p, hsa-miR-190b-5p
FMNL2	18	hsa-miR-20b-5p, hsa-miR-1-3p, hsa-miR-148a-3p, hsa-miR-7-5p, hsa-miR-138-5p, hsa-miR-361-5p, hsa-miR-574-5p, hsa-miR-589-5p, hsa-miR-188-5p, hsa-miR-629-5p, hsa-miR-335-3p, hsa-miR-425-3p, hsa-miR-551b-3p, hsa-miR-320c, hsa-miR-133a-3p, hsa-miR-887-3p, hsa-miR-320a-3p, hsa-miR-147b-3p
FOSL2	18	hsa-miR-133a-3p, hsa-miR-148a-3p, hsa-miR-20b-5p, hsa-miR-7-5p, hsa-miR-138-5p, hsa-miR-361-5p, hsa-miR-339-5p, hsa-miR-574-5p, hsa-miR-629-5p, hsa-miR-361-3p, hsa-miR-342-5p, hsa-miR-425-3p, hsa-miR-500a-3p, hsa-miR-671-5p, hsa-miR-378i, hsa-miR-1-3p, hsa-miR-320a-3p, hsa-miR-147b-3p
FOXP1	18	hsa-miR-1-3p, hsa-miR-148a-3p, hsa-miR-335-5p, hsa-miR-335-3p, hsa-miR-20b-5p, hsa-miR-7-5p, hsa-miR-138-5p, hsa-miR-361-5p, hsa-miR-589-5p, hsa-miR-320d, hsa-miR-629-5p, hsa-miR-500a-3p, hsa-miR-671-5p, hsa-miR-320c, hsa-miR-874-3p, hsa-miR-1468-5p, hsa-miR-320a-3p, hsa-miR-147b-3p
FRS2	18	hsa-miR-20b-5p, hsa-miR-7-5p, hsa-miR-9-3p, hsa-miR-1-3p, hsa-miR-148a-3p, hsa-miR-138-5p, hsa-miR-361-5p, hsa-miR-339-5p, hsa-miR-320d, hsa-miR-1275, hsa-miR-629-5p, hsa-miR-335-3p, hsa-miR-1307-3p, hsa-miR-2110, hsa-miR-320c, hsa-miR-887-3p, hsa-miR-320a-3p, hsa-miR-147b-3p
GLCCI1	18	hsa-miR-335-5p, hsa-miR-675-3p, hsa-miR-20b-5p, hsa-miR-361-5p, hsa-miR-574-5p, hsa-miR-769-5p, hsa-miR-1275, hsa-miR-629-5p, hsa-miR-342-5p, hsa-miR-425-3p, hsa-miR-500a-3p, hsa-miR-671-5p, hsa-miR-2110, hsa-miR-320c, hsa-miR-1-3p, hsa-miR-887-3p, hsa-miR-320a-3p, hsa-miR-147b-3p

GLS	18	hsa-miR-335-5p, hsa-miR-7-5p, hsa-miR-148a-3p, hsa-miR-20b-5p, hsa-miR-9-3p, hsa-miR-361-5p, hsa-miR-769-5p, hsa-miR-320d, hsa-miR-335-3p, hsa-miR-425-3p, hsa-miR-500a-3p, hsa-miR-2110, hsa-miR-320c, hsa-miR-378i, hsa-miR-874-3p, hsa-miR-1-3p, hsa-miR-133a-3p, hsa-miR-320a-3p
GSK3B	18	hsa-miR-769-5p, hsa-miR-148a-3p, hsa-miR-20b-5p, hsa-miR-7-5p, hsa-miR-138-5p, hsa-miR-574-5p, hsa-miR-320d, hsa-miR-1275, hsa-miR-629-5p, hsa-miR-361-3p, hsa-miR-335-3p, hsa-miR-671-5p, hsa-miR-3168, hsa-miR-320c, hsa-miR-874-3p, hsa-miR-1-3p, hsa-miR-320a-3p, hsa-miR-147b-3p
GSPT1	18	hsa-miR-361-5p, hsa-miR-769-5p, hsa-miR-148a-3p, hsa-miR-20b-5p, hsa-miR-335-5p, hsa-miR-7-5p, hsa-miR-138-5p, hsa-miR-574-5p, hsa-miR-320d, hsa-miR-335-3p, hsa-miR-425-3p, hsa-miR-500a-3p, hsa-miR-671-5p, hsa-miR-675-3p, hsa-miR-320c, hsa-miR-378i, hsa-miR-133a-3p, hsa-miR-320a-3p
HCFC1	18	hsa-miR-148a-3p, hsa-miR-20b-5p, hsa-miR-7-5p, hsa-miR-138-5p, hsa-miR-339-5p, hsa-miR-574-5p, hsa-miR-769-5p, hsa-miR-188-5p, hsa-miR-29c-5p, hsa-miR-342-5p, hsa-miR-335-3p, hsa-miR-1307-5p, hsa-miR-3615, hsa-miR-1-3p, hsa-miR-133a-3p, hsa-miR-887-3p, hsa-miR-320a-3p, hsa-miR-147b-3p
HMGB1	18	hsa-miR-148a-3p, hsa-miR-20b-5p, hsa-miR-574-5p, hsa-miR-188-5p, hsa-miR-1307-3p, hsa-miR-7-5p, hsa-miR-9-3p, hsa-miR-138-5p, hsa-miR-361-5p, hsa-miR-589-5p, hsa-miR-361-3p, hsa-miR-342-5p, hsa-miR-335-3p, hsa-miR-551b-3p, hsa-miR-2110, hsa-miR-320c, hsa-miR-320a-3p, hsa-miR-190b-5p
HNRNPF	18	hsa-miR-339-5p, hsa-miR-361-3p, hsa-miR-874-3p, hsa-miR-148a-3p, hsa-miR-20b-5p, hsa-miR-335-5p, hsa-miR-7-5p, hsa-miR-138-5p, hsa-miR-29c-5p, hsa-miR-335-3p, hsa-miR-500a-3p, hsa-miR-551b-3p, hsa-miR-671-5p, hsa-miR-2110, hsa-miR-320c, hsa-miR-378i, hsa-miR-133a-3p, hsa-miR-320a-3p
HNRNPK	18	hsa-miR-148a-3p, hsa-miR-20b-5p, hsa-miR-335-5p, hsa-miR-7-5p, hsa-miR-361-5p, hsa-miR-589-5p, hsa-miR-335-3p, hsa-miR-500a-3p, hsa-miR-1307-3p, hsa-miR-1307-5p, hsa-miR-3168, hsa-miR-2110, hsa-miR-320c, hsa-miR-378i, hsa-miR-1-3p, hsa-miR-133a-3p, hsa-miR-887-3p, hsa-miR-320a-3p

IQGAP1	18	hsa-miR-148a-3p, hsa-miR-20b-5p, hsa-miR-7-5p, hsa-miR-138-5p, hsa-miR-361-5p, hsa-miR-574-5p, hsa-miR-769-5p, hsa-miR-629-5p, hsa-miR-361-3p, hsa-miR-335-3p, hsa-miR-500a-3p, hsa-miR-1307-3p, hsa-miR-320c, hsa-miR-378i, hsa-miR-1-3p, hsa-miR-133a-3p, hsa-miR-320a-3p, hsa-miR-147b-3p
IRS2	18	hsa-miR-7-5p, hsa-miR-148a-3p, hsa-miR-20b-5p, hsa-miR-335-5p, hsa-miR-138-5p, hsa-miR-361-5p, hsa-miR-574-5p, hsa-miR-769-5p, hsa-miR-335-3p, hsa-miR-425-3p, hsa-miR-500a-3p, hsa-miR-671-5p, hsa-miR-1307-3p, hsa-miR-1307-5p, hsa-miR-320c, hsa-miR-1-3p, hsa-miR-133a-3p, hsa-miR-320a-3p
ITSN2	18	hsa-miR-320c, hsa-miR-148a-3p, hsa-miR-20b-5p, hsa-miR-335-5p, hsa-miR-7-5p, hsa-miR-9-3p, hsa-miR-138-5p, hsa-miR-361-5p, hsa-miR-589-5p, hsa-miR-629-5p, hsa-miR-342-5p, hsa-miR-335-3p, hsa-miR-500a-3p, hsa-miR-671-5p, hsa-miR-2110, hsa-miR-133a-3p, hsa-miR-320a-3p, hsa-miR-147b-3p
KBTBD2	18	hsa-miR-148a-3p, hsa-miR-20b-5p, hsa-miR-335-5p, hsa-miR-7-5p, hsa-miR-9-3p, hsa-miR-138-5p, hsa-miR-361-5p, hsa-miR-574-5p, hsa-miR-589-5p, hsa-miR-769-5p, hsa-miR-335-3p, hsa-miR-425-3p, hsa-miR-500a-3p, hsa-miR-671-5p, hsa-miR-675-3p, hsa-miR-320c, hsa-miR-1-3p, hsa-miR-320a-3p
KDM5C	18	hsa-miR-138-5p, hsa-miR-425-3p, hsa-miR-4516, hsa-miR-133a-3p, hsa-miR-148a-3p, hsa-miR-20b-5p, hsa-miR-335-5p, hsa-miR-7-5p, hsa-miR-361-5p, hsa-miR-574-5p, hsa-miR-589-5p, hsa-miR-769-5p, hsa-miR-335-3p, hsa-miR-500a-3p, hsa-miR-671-5p, hsa-miR-1307-3p, hsa-miR-2110, hsa-miR-1-3p
KHDC4	18	hsa-miR-148a-3p, hsa-miR-20b-5p, hsa-miR-335-5p, hsa-miR-7-5p, hsa-miR-138-5p, hsa-miR-361-5p, hsa-miR-339-5p, hsa-miR-574-5p, hsa-miR-188-5p, hsa-miR-320d, hsa-miR-342-5p, hsa-miR-500a-3p, hsa-miR-671-5p, hsa-miR-2110, hsa-miR-320c, hsa-miR-378i, hsa-miR-1-3p, hsa-miR-320a-3p
KIF1B	18	hsa-miR-574-5p, hsa-miR-148a-3p, hsa-miR-20b-5p, hsa-miR-7-5p, hsa-miR-138-5p, hsa-miR-361-5p, hsa-miR-769-5p, hsa-miR-188-5p, hsa-miR-342-5p, hsa-miR-335-3p, hsa-miR-425-3p, hsa-miR-500a-3p, hsa-miR-1307-3p, hsa-miR-675-3p, hsa-miR-320c, hsa-miR-3615, hsa-miR-320a-3p, hsa-miR-147b-3p

KLF6	18	hsa-miR-148a-3p, hsa-miR-20b-5p, hsa-miR-335-5p, hsa-miR-7-5p, hsa-miR-138-5p, hsa-miR-574-5p, hsa-miR-589-5p, hsa-miR-769-5p, hsa-miR-320d, hsa-miR-29c-5p, hsa-miR-425-3p, hsa-miR-500a-3p, hsa-miR-671-5p, hsa-miR-2110, hsa-miR-320c, hsa-miR-1-3p, hsa-miR-133a-3p, hsa-miR-320a-3p
MAP4	18	hsa-miR-339-5p, hsa-miR-551b-3p, hsa-miR-148a-3p, hsa-miR-20b-5p, hsa-miR-7-5p, hsa-miR-138-5p, hsa-miR-361-5p, hsa-miR-589-5p, hsa-miR-769-5p, hsa-miR-320d, hsa-miR-361-3p, hsa-miR-342-5p, hsa-miR-335-3p, hsa-miR-425-3p, hsa-miR-671-5p, hsa-miR-2110, hsa-miR-320c, hsa-miR-320a-3p
MEF2D	18	hsa-miR-335-5p, hsa-miR-7-5p, hsa-miR-7704, hsa-miR-148a-3p, hsa-miR-20b-5p, hsa-miR-138-5p, hsa-miR-574-5p, hsa-miR-320d, hsa-miR-1275, hsa-miR-629-5p, hsa-miR-342-5p, hsa-miR-335-3p, hsa-miR-500a-3p, hsa-miR-671-5p, hsa-miR-874-3p, hsa-miR-1-3p, hsa-miR-887-3p, hsa-miR-320a-3p
MIER3	18	hsa-miR-2110, hsa-miR-148a-3p, hsa-miR-20b-5p, hsa-miR-7-5p, hsa-miR-9-3p, hsa-miR-361-5p, hsa-miR-339-5p, hsa-miR-574-5p, hsa-miR-188-5p, hsa-miR-320d, hsa-miR-629-5p, hsa-miR-342-5p, hsa-miR-335-3p, hsa-miR-425-3p, hsa-miR-320c, hsa-miR-378i, hsa-miR-1-3p, hsa-miR-320a-3p
MSL1	18	hsa-miR-148a-3p, hsa-miR-20b-5p, hsa-miR-335-5p, hsa-miR-7-5p, hsa-miR-361-5p, hsa-miR-589-5p, hsa-miR-769-5p, hsa-miR-342-5p, hsa-miR-335-3p, hsa-miR-425-3p, hsa-miR-500a-3p, hsa-miR-1307-5p, hsa-miR-2110, hsa-miR-320c, hsa-miR-3615, hsa-miR-1-3p, hsa-miR-320a-3p, hsa-miR-147b-3p
MT-CO1	18	hsa-miR-361-5p, hsa-miR-1-3p, hsa-miR-148a-3p, hsa-miR-20b-5p, hsa-miR-7-5p, hsa-miR-138-5p, hsa-miR-339-5p, hsa-miR-574-5p, hsa-miR-188-5p, hsa-miR-320d, hsa-miR-629-5p, hsa-miR-335-3p, hsa-miR-425-3p, hsa-miR-671-5p, hsa-miR-1307-3p, hsa-miR-2110, hsa-miR-320c, hsa-miR-320a-3p
MT-ND4	18	hsa-miR-148a-3p, hsa-miR-335-5p, hsa-miR-7-5p, hsa-miR-138-5p, hsa-miR-361-5p, hsa-miR-339-5p, hsa-miR-574-5p, hsa-miR-589-5p, hsa-miR-188-5p, hsa-miR-320d, hsa-miR-1275, hsa-miR-629-5p, hsa-miR-342-5p, hsa-miR-671-5p, hsa-miR-320c, hsa-miR-3615, hsa-miR-1-3p, hsa-miR-320a-3p

MTDH	18	hsa-miR-148a-3p, hsa-miR-20b-5p, hsa-miR-7-5p, hsa-miR-9-3p, hsa-miR-138-5p, hsa-miR-361-5p, hsa-miR-320d, hsa-miR-1275, hsa-miR-629-5p, hsa-miR-361-3p, hsa-miR-335-3p, hsa-miR-500a-3p, hsa-miR-671-5p, hsa-miR-2110, hsa-miR-320c, hsa-miR-378i, hsa-miR-887-3p, hsa-miR-320a-3p
MYOF	18	hsa-miR-148a-3p, hsa-miR-20b-5p, hsa-miR-335-5p, hsa-miR-7-5p, hsa-miR-138-5p, hsa-miR-361-5p, hsa-miR-574-5p, hsa-miR-769-5p, hsa-miR-629-5p, hsa-miR-335-3p, hsa-miR-500a-3p, hsa-miR-671-5p, hsa-miR-320c, hsa-miR-3615, hsa-miR-1-3p, hsa-miR-133a-3p, hsa-miR-320a-3p, hsa-miR-147b-3p
NAA50	18	hsa-miR-20b-5p, hsa-miR-1275, hsa-miR-342-5p, hsa-miR-148a-3p, hsa-miR-335-5p, hsa-miR-7-5p, hsa-miR-138-5p, hsa-miR-361-5p, hsa-miR-589-5p, hsa-miR-629-5p, hsa-miR-335-3p, hsa-miR-425-3p, hsa-miR-500a-3p, hsa-miR-2110, hsa-miR-320c, hsa-miR-3615, hsa-miR-1-3p, hsa-miR-320a-3p
NCKAP1	18	hsa-miR-335-3p, hsa-miR-148a-3p, hsa-miR-20b-5p, hsa-miR-335-5p, hsa-miR-7-5p, hsa-miR-361-5p, hsa-miR-589-5p, hsa-miR-320d, hsa-miR-500a-3p, hsa-miR-671-5p, hsa-miR-2110, hsa-miR-320c, hsa-miR-378i, hsa-miR-874-3p, hsa-miR-1-3p, hsa-miR-133a-3p, hsa-miR-320a-3p, hsa-miR-147b-3p
NDFIP1	18	hsa-miR-148a-3p, hsa-miR-20b-5p, hsa-miR-335-5p, hsa-miR-7-5p, hsa-miR-9-3p, hsa-miR-138-5p, hsa-miR-361-5p, hsa-miR-769-5p, hsa-miR-1275, hsa-miR-335-3p, hsa-miR-500a-3p, hsa-miR-671-5p, hsa-miR-1307-5p, hsa-miR-320c, hsa-miR-1-3p, hsa-miR-133a-3p, hsa-miR-320a-3p, hsa-miR-147b-3p
NEO1	18	hsa-miR-148a-3p, hsa-miR-20b-5p, hsa-miR-7-5p, hsa-miR-138-5p, hsa-miR-361-5p, hsa-miR-188-5p, hsa-miR-320d, hsa-miR-29c-5p, hsa-miR-335-3p, hsa-miR-551b-3p, hsa-miR-671-5p, hsa-miR-2110, hsa-miR-320c, hsa-miR-378i, hsa-miR-1-3p, hsa-miR-133a-3p, hsa-miR-320a-3p, hsa-miR-147b-3p
NFIA	18	hsa-miR-335-3p, hsa-miR-148a-3p, hsa-miR-20b-5p, hsa-miR-7-5p, hsa-miR-361-5p, hsa-miR-339-5p, hsa-miR-574-5p, hsa-miR-342-5p, hsa-miR-425-3p, hsa-miR-500a-3p, hsa-miR-671-5p, hsa-miR-2110, hsa-miR-320c, hsa-miR-1-3p, hsa-miR-133a-3p, hsa-miR-320a-3p, hsa-miR-147b-3p, hsa-miR-190b-5p

NFIX	18	hsa-miR-1275, hsa-miR-148a-3p, hsa-miR-20b-5p, hsa-miR-335-5p, hsa-miR-7-5p, hsa-miR-138-5p, hsa-miR-361-5p, hsa-miR-339-5p, hsa-miR-589-5p, hsa-miR-769-5p, hsa-miR-342-5p, hsa-miR-335-3p, hsa-miR-671-5p, hsa-miR-378i, hsa-miR-874-3p, hsa-miR-1-3p, hsa-miR-320a-3p, hsa-miR-147b-3p
NR1D2	18	hsa-miR-148a-3p, hsa-miR-20b-5p, hsa-miR-335-5p, hsa-miR-7-5p, hsa-miR-9-3p, hsa-miR-361-5p, hsa-miR-574-5p, hsa-miR-629-5p, hsa-miR-361-3p, hsa-miR-335-3p, hsa-miR-500a-3p, hsa-miR-671-5p, hsa-miR-2110, hsa-miR-320c, hsa-miR-378i, hsa-miR-1-3p, hsa-miR-320a-3p, hsa-miR-147b-3p
NRBP1	18	hsa-miR-20b-5p, hsa-miR-339-5p, hsa-miR-148a-3p, hsa-miR-335-5p, hsa-miR-7-5p, hsa-miR-138-5p, hsa-miR-574-5p, hsa-miR-1275, hsa-miR-342-5p, hsa-miR-335-3p, hsa-miR-425-3p, hsa-miR-500a-3p, hsa-miR-671-5p, hsa-miR-2110, hsa-miR-133a-3p, hsa-miR-320a-3p, hsa-miR-147b-3p, hsa-miR-190b-5p
NUP98	18	hsa-miR-20b-5p, hsa-miR-148a-3p, hsa-miR-335-5p, hsa-miR-7-5p, hsa-miR-9-3p, hsa-miR-138-5p, hsa-miR-361-5p, hsa-miR-769-5p, hsa-miR-342-5p, hsa-miR-425-3p, hsa-miR-500a-3p, hsa-miR-671-5p, hsa-miR-1307-3p, hsa-miR-320c, hsa-miR-3615, hsa-miR-378i, hsa-miR-320a-3p, hsa-miR-147b-3p
OSBP	18	hsa-miR-335-5p, hsa-miR-148a-3p, hsa-miR-20b-5p, hsa-miR-7-5p, hsa-miR-138-5p, hsa-miR-361-5p, hsa-miR-361-3p, hsa-miR-342-5p, hsa-miR-335-3p, hsa-miR-500a-3p, hsa-miR-671-5p, hsa-miR-2110, hsa-miR-320c, hsa-miR-378i, hsa-miR-1-3p, hsa-miR-133a-3p, hsa-miR-320a-3p, hsa-miR-147b-3p
OSBPL8	18	hsa-miR-7-5p, hsa-miR-20b-5p, hsa-miR-335-5p, hsa-miR-138-5p, hsa-miR-361-5p, hsa-miR-574-5p, hsa-miR-589-5p, hsa-miR-320d, hsa-miR-629-5p, hsa-miR-361-3p, hsa-miR-342-5p, hsa-miR-500a-3p, hsa-miR-671-5p, hsa-miR-2110, hsa-miR-320c, hsa-miR-1-3p, hsa-miR-133a-3p, hsa-miR-320a-3p
PAN3	18	hsa-miR-148a-3p, hsa-miR-20b-5p, hsa-miR-7-5p, hsa-miR-9-3p, hsa-miR-138-5p, hsa-miR-361-5p, hsa-miR-574-5p, hsa-miR-769-5p, hsa-miR-1275, hsa-miR-342-5p, hsa-miR-335-3p, hsa-miR-500a-3p, hsa-miR-2110, hsa-miR-320c, hsa-miR-1-3p, hsa-miR-133a-3p, hsa-miR-320a-3p, hsa-miR-190b-5p

PAPOLA	18	hsa-miR-148a-3p, hsa-miR-20b-5p, hsa-miR-7-5p, hsa-miR-138-5p, hsa-miR-361-5p, hsa-miR-589-5p, hsa-miR-769-5p, hsa-miR-320d, hsa-miR-1275, hsa-miR-629-5p, hsa-miR-342-5p, hsa-miR-335-3p, hsa-miR-425-3p, hsa-miR-671-5p, hsa-miR-2110, hsa-miR-320c, hsa-miR-1-3p, hsa-miR-320a-3p
PCNX1	18	hsa-miR-148a-3p, hsa-miR-20b-5p, hsa-miR-335-5p, hsa-miR-7-5p, hsa-miR-9-3p, hsa-miR-138-5p, hsa-miR-361-5p, hsa-miR-574-5p, hsa-miR-769-5p, hsa-miR-188-5p, hsa-miR-320d, hsa-miR-629-5p, hsa-miR-361-3p, hsa-miR-335-3p, hsa-miR-500a-3p, hsa-miR-2110, hsa-miR-133a-3p, hsa-miR-320a-3p
PHC3	18	hsa-miR-148a-3p, hsa-miR-20b-5p, hsa-miR-7-5p, hsa-miR-138-5p, hsa-miR-361-5p, hsa-miR-574-5p, hsa-miR-589-5p, hsa-miR-629-5p, hsa-miR-342-5p, hsa-miR-425-3p, hsa-miR-500a-3p, hsa-miR-671-5p, hsa-miR-320c, hsa-miR-1-3p, hsa-miR-133a-3p, hsa-miR-887-3p, hsa-miR-320a-3p, hsa-miR-147b-3p
PICALM	18	hsa-miR-1-3p, hsa-miR-148a-3p, hsa-miR-20b-5p, hsa-miR-7-5p, hsa-miR-138-5p, hsa-miR-361-5p, hsa-miR-574-5p, hsa-miR-589-5p, hsa-miR-320d, hsa-miR-1275, hsa-miR-629-5p, hsa-miR-361-3p, hsa-miR-500a-3p, hsa-miR-671-5p, hsa-miR-2110, hsa-miR-320c, hsa-miR-133a-3p, hsa-miR-320a-3p
PPP1CB	18	hsa-miR-148a-3p, hsa-miR-20b-5p, hsa-miR-7-5p, hsa-miR-9-3p, hsa-miR-138-5p, hsa-miR-361-5p, hsa-miR-339-5p, hsa-miR-574-5p, hsa-miR-589-5p, hsa-miR-769-5p, hsa-miR-629-5p, hsa-miR-342-5p, hsa-miR-335-3p, hsa-miR-425-3p, hsa-miR-500a-3p, hsa-miR-1-3p, hsa-miR-320a-3p, hsa-miR-147b-3p
PTBP3	18	hsa-miR-148a-3p, hsa-miR-20b-5p, hsa-miR-335-5p, hsa-miR-7-5p, hsa-miR-138-5p, hsa-miR-361-5p, hsa-miR-574-5p, hsa-miR-589-5p, hsa-miR-769-5p, hsa-miR-320d, hsa-miR-335-3p, hsa-miR-500a-3p, hsa-miR-671-5p, hsa-miR-2110, hsa-miR-320c, hsa-miR-133a-3p, hsa-miR-320a-3p, hsa-miR-147b-3p
RAB10	18	hsa-miR-148a-3p, hsa-miR-20b-5p, hsa-miR-1307-3p, hsa-miR-7-5p, hsa-miR-138-5p, hsa-miR-361-5p, hsa-miR-339-5p, hsa-miR-574-5p, hsa-miR-589-5p, hsa-miR-769-5p, hsa-miR-629-5p, hsa-miR-335-3p, hsa-miR-500a-3p, hsa-miR-2110, hsa-miR-320c, hsa-miR-378i, hsa-miR-320a-3p, hsa-miR-147b-3p

RAB14	18	hsa-miR-148a-3p, hsa-miR-20b-5p, hsa-miR-7-5p, hsa-miR-9-3p, hsa-miR-138-5p, hsa-miR-339-5p, hsa-miR-589-5p, hsa-miR-320d, hsa-miR-1275, hsa-miR-629-5p, hsa-miR-361-3p, hsa-miR-500a-3p, hsa-miR-2110, hsa-miR-320c, hsa-miR-3615, hsa-miR-378i, hsa-miR-1-3p, hsa-miR-320a-3p
RAD23B	18	hsa-miR-361-5p, hsa-miR-148a-3p, hsa-miR-20b-5p, hsa-miR-335-5p, hsa-miR-7-5p, hsa-miR-9-3p, hsa-miR-138-5p, hsa-miR-574-5p, hsa-miR-769-5p, hsa-miR-320d, hsa-miR-1275, hsa-miR-29c-5p, hsa-miR-361-3p, hsa-miR-500a-3p, hsa-miR-671-5p, hsa-miR-320c, hsa-miR-874-3p, hsa-miR-320a-3p
RAI1	18	hsa-miR-148a-3p, hsa-miR-20b-5p, hsa-miR-335-5p, hsa-miR-7-5p, hsa-miR-138-5p, hsa-miR-361-5p, hsa-miR-339-5p, hsa-miR-574-5p, hsa-miR-589-5p, hsa-miR-629-5p, hsa-miR-342-5p, hsa-miR-335-3p, hsa-miR-500a-3p, hsa-miR-1307-3p, hsa-miR-2110, hsa-miR-320c, hsa-miR-1-3p, hsa-miR-320a-3p
RBM26	18	hsa-miR-148a-3p, hsa-miR-20b-5p, hsa-miR-335-5p, hsa-miR-7-5p, hsa-miR-138-5p, hsa-miR-361-5p, hsa-miR-339-5p, hsa-miR-574-5p, hsa-miR-769-5p, hsa-miR-320d, hsa-miR-1275, hsa-miR-29c-5p, hsa-miR-342-5p, hsa-miR-335-3p, hsa-miR-500a-3p, hsa-miR-2110, hsa-miR-320c, hsa-miR-320a-3p
RBM33	18	hsa-miR-188-5p, hsa-miR-148a-3p, hsa-miR-20b-5p, hsa-miR-335-5p, hsa-miR-7-5p, hsa-miR-339-5p, hsa-miR-574-5p, hsa-miR-769-5p, hsa-miR-335-3p, hsa-miR-500a-3p, hsa-miR-1307-3p, hsa-miR-1307-5p, hsa-miR-320c, hsa-miR-1-3p, hsa-miR-133a-3p, hsa-miR-320a-3p, hsa-miR-147b-3p, hsa-miR-190b-5p
RC3H1	18	hsa-miR-148a-3p, hsa-miR-20b-5p, hsa-miR-335-5p, hsa-miR-7-5p, hsa-miR-138-5p, hsa-miR-361-5p, hsa-miR-589-5p, hsa-miR-320d, hsa-miR-335-3p, hsa-miR-500a-3p, hsa-miR-671-5p, hsa-miR-320c, hsa-miR-378i, hsa-miR-1468-5p, hsa-miR-1-3p, hsa-miR-133a-3p, hsa-miR-320a-3p, hsa-miR-147b-3p
REST	18	hsa-miR-20b-5p, hsa-miR-335-5p, hsa-miR-9-3p, hsa-miR-4516, hsa-miR-378i, hsa-miR-148a-3p, hsa-miR-7-5p, hsa-miR-589-5p, hsa-miR-769-5p, hsa-miR-188-5p, hsa-miR-1275, hsa-miR-335-3p, hsa-miR-500a-3p, hsa-miR-671-5p, hsa-miR-675-3p, hsa-miR-2110, hsa-miR-320a-3p, hsa-miR-147b-3p

RFX7	18	hsa-miR-148a-3p, hsa-miR-20b-5p, hsa-miR-335-5p, hsa-miR-7-5p, hsa-miR-9-3p, hsa-miR-361-5p, hsa-miR-339-5p, hsa-miR-1275, hsa-miR-629-5p, hsa-miR-29c-5p, hsa-miR-342-5p, hsa-miR-335-3p, hsa-miR-320c, hsa-miR-378i, hsa-miR-1-3p, hsa-miR-133a-3p, hsa-miR-320a-3p, hsa-miR-147b-3p
RMND5A	18	hsa-miR-138-5p, hsa-miR-148a-3p, hsa-miR-20b-5p, hsa-miR-335-5p, hsa-miR-7-5p, hsa-miR-9-3p, hsa-miR-361-5p, hsa-miR-589-5p, hsa-miR-188-5p, hsa-miR-320d, hsa-miR-629-5p, hsa-miR-29c-5p, hsa-miR-335-3p, hsa-miR-500a-3p, hsa-miR-320c, hsa-miR-1-3p, hsa-miR-320a-3p, hsa-miR-147b-3p
RPS6	18	hsa-miR-148a-3p, hsa-miR-20b-5p, hsa-miR-335-5p, hsa-miR-361-5p, hsa-miR-589-5p, hsa-miR-769-5p, hsa-miR-335-3p, hsa-miR-425-3p, hsa-miR-500a-3p, hsa-miR-1307-3p, hsa-miR-675-3p, hsa-miR-320c, hsa-miR-3615, hsa-miR-1468-5p, hsa-miR-1-3p, hsa-miR-133a-3p, hsa-miR-320a-3p, hsa-miR-147b-3p
SCAF11	18	hsa-miR-1-3p, hsa-miR-148a-3p, hsa-miR-20b-5p, hsa-miR-7-5p, hsa-miR-138-5p, hsa-miR-361-5p, hsa-miR-574-5p, hsa-miR-589-5p, hsa-miR-188-5p, hsa-miR-320d, hsa-miR-342-5p, hsa-miR-335-3p, hsa-miR-425-3p, hsa-miR-1307-3p, hsa-miR-2110, hsa-miR-320c, hsa-miR-133a-3p, hsa-miR-320a-3p
SEC24A	18	hsa-miR-9-3p, hsa-miR-320d, hsa-miR-320c, hsa-miR-20b-5p, hsa-miR-7-5p, hsa-miR-138-5p, hsa-miR-361-5p, hsa-miR-574-5p, hsa-miR-589-5p, hsa-miR-188-5p, hsa-miR-629-5p, hsa-miR-342-5p, hsa-miR-500a-3p, hsa-miR-671-5p, hsa-miR-2110, hsa-miR-1-3p, hsa-miR-887-3p, hsa-miR-320a-3p
SETD2	18	hsa-miR-148a-3p, hsa-miR-20b-5p, hsa-miR-335-5p, hsa-miR-7-5p, hsa-miR-138-5p, hsa-miR-361-5p, hsa-miR-339-5p, hsa-miR-589-5p, hsa-miR-629-5p, hsa-miR-335-3p, hsa-miR-425-3p, hsa-miR-500a-3p, hsa-miR-671-5p, hsa-miR-320c, hsa-miR-3615, hsa-miR-874-3p, hsa-miR-320a-3p, hsa-miR-147b-3p
SHOC2	18	hsa-miR-20b-5p, hsa-miR-335-5p, hsa-miR-2110, hsa-miR-148a-3p, hsa-miR-7-5p, hsa-miR-9-3p, hsa-miR-138-5p, hsa-miR-361-5p, hsa-miR-574-5p, hsa-miR-589-5p, hsa-miR-320d, hsa-miR-629-5p, hsa-miR-671-5p, hsa-miR-3168, hsa-miR-320c, hsa-miR-3615, hsa-miR-1-3p, hsa-miR-320a-3p

SIN3A	18	hsa-miR-1-3p, hsa-miR-148a-3p, hsa-miR-20b-5p, hsa-miR-7-5p, hsa-miR-138-5p, hsa-miR-361-5p, hsa-miR-339-5p, hsa-miR-589-5p, hsa-miR-1275, hsa-miR-29c-5p, hsa-miR-361-3p, hsa-miR-335-3p, hsa-miR-500a-3p, hsa-miR-2110, hsa-miR-320c, hsa-miR-3615, hsa-miR-320a-3p, hsa-miR-190b-5p
SLC38A1	18	hsa-miR-320d, hsa-miR-335-3p, hsa-miR-148a-3p, hsa-miR-20b-5p, hsa-miR-335-5p, hsa-miR-7-5p, hsa-miR-138-5p, hsa-miR-361-5p, hsa-miR-589-5p, hsa-miR-769-5p, hsa-miR-629-5p, hsa-miR-500a-3p, hsa-miR-671-5p, hsa-miR-2110, hsa-miR-320c, hsa-miR-378i, hsa-miR-1-3p, hsa-miR-320a-3p
SMAD5	18	hsa-miR-20b-5p, hsa-miR-675-3p, hsa-miR-148a-3p, hsa-miR-7-5p, hsa-miR-138-5p, hsa-miR-574-5p, hsa-miR-320d, hsa-miR-629-5p, hsa-miR-342-5p, hsa-miR-335-3p, hsa-miR-500a-3p, hsa-miR-671-5p, hsa-miR-3168, hsa-miR-2110, hsa-miR-320c, hsa-miR-1-3p, hsa-miR-133a-3p, hsa-miR-320a-3p
SRPRA	18	hsa-miR-148a-3p, hsa-miR-335-5p, hsa-miR-7-5p, hsa-miR-138-5p, hsa-miR-361-5p, hsa-miR-574-5p, hsa-miR-589-5p, hsa-miR-769-5p, hsa-miR-1275, hsa-miR-629-5p, hsa-miR-342-5p, hsa-miR-335-3p, hsa-miR-500a-3p, hsa-miR-671-5p, hsa-miR-2110, hsa-miR-320c, hsa-miR-320a-3p, hsa-miR-147b-3p
SYNE1	18	hsa-miR-1-3p, hsa-miR-148a-3p, hsa-miR-20b-5p, hsa-miR-335-5p, hsa-miR-7-5p, hsa-miR-138-5p, hsa-miR-361-5p, hsa-miR-589-5p, hsa-miR-769-5p, hsa-miR-188-5p, hsa-miR-629-5p, hsa-miR-29c-5p, hsa-miR-425-3p, hsa-miR-320c, hsa-miR-378i, hsa-miR-133a-3p, hsa-miR-887-3p, hsa-miR-320a-3p
TAOK2	18	hsa-miR-769-5p, hsa-miR-148a-3p, hsa-miR-20b-5p, hsa-miR-335-5p, hsa-miR-7-5p, hsa-miR-138-5p, hsa-miR-339-5p, hsa-miR-589-5p, hsa-miR-188-5p, hsa-miR-1275, hsa-miR-361-3p, hsa-miR-335-3p, hsa-miR-500a-3p, hsa-miR-1307-5p, hsa-miR-2110, hsa-miR-320c, hsa-miR-320a-3p, hsa-miR-147b-3p
TASOR2	18	hsa-miR-320d, hsa-miR-320c, hsa-miR-148a-3p, hsa-miR-20b-5p, hsa-miR-7-5p, hsa-miR-9-3p, hsa-miR-138-5p, hsa-miR-361-5p, hsa-miR-589-5p, hsa-miR-335-3p, hsa-miR-500a-3p, hsa-miR-671-5p, hsa-miR-3168, hsa-miR-2110, hsa-miR-378i, hsa-miR-1-3p, hsa-miR-887-3p, hsa-miR-320a-3p

TCF3	18	hsa-miR-342-5p, hsa-miR-148a-3p, hsa-miR-20b-5p, hsa-miR-335-5p, hsa-miR-7-5p, hsa-miR-138-5p, hsa-miR-339-5p, hsa-miR-769-5p, hsa-miR-361-3p, hsa-miR-335-3p, hsa-miR-500a-3p, hsa-miR-671-5p, hsa-miR-2110, hsa-miR-3615, hsa-miR-378i, hsa-miR-133a-3p, hsa-miR-887-3p, hsa-miR-147b-3p
TET2	18	hsa-miR-335-5p, hsa-miR-7-5p, hsa-miR-148a-3p, hsa-miR-20b-5p, hsa-miR-361-5p, hsa-miR-574-5p, hsa-miR-320d, hsa-miR-1275, hsa-miR-629-5p, hsa-miR-361-3p, hsa-miR-1307-5p, hsa-miR-3168, hsa-miR-2110, hsa-miR-320c, hsa-miR-1-3p, hsa-miR-320a-3p, hsa-miR-147b-3p, hsa-miR-190b-5p
TLN1	18	hsa-miR-1275, hsa-miR-148a-3p, hsa-miR-20b-5p, hsa-miR-335-5p, hsa-miR-7-5p, hsa-miR-138-5p, hsa-miR-339-5p, hsa-miR-574-5p, hsa-miR-589-5p, hsa-miR-769-5p, hsa-miR-629-5p, hsa-miR-335-3p, hsa-miR-500a-3p, hsa-miR-671-5p, hsa-miR-1307-3p, hsa-miR-378i, hsa-miR-133a-3p, hsa-miR-147b-3p
TRA2B	18	hsa-miR-339-5p, hsa-miR-1-3p, hsa-miR-148a-3p, hsa-miR-20b-5p, hsa-miR-574-5p, hsa-miR-589-5p, hsa-miR-769-5p, hsa-miR-1275, hsa-miR-335-3p, hsa-miR-1307-5p, hsa-miR-320c, hsa-miR-378i, hsa-miR-874-3p, hsa-miR-133a-3p, hsa-miR-887-3p, hsa-miR-320a-3p, hsa-miR-147b-3p, hsa-miR-190b-5p
TSC22D2	18	hsa-miR-574-5p, hsa-miR-188-5p, hsa-miR-148a-3p, hsa-miR-20b-5p, hsa-miR-7-5p, hsa-miR-138-5p, hsa-miR-361-5p, hsa-miR-339-5p, hsa-miR-589-5p, hsa-miR-320d, hsa-miR-342-5p, hsa-miR-335-3p, hsa-miR-500a-3p, hsa-miR-320c, hsa-miR-3615, hsa-miR-1-3p, hsa-miR-320a-3p, hsa-miR-190b-5p
TUBA1B	18	hsa-miR-148a-3p, hsa-miR-20b-5p, hsa-miR-335-5p, hsa-miR-7-5p, hsa-miR-138-5p, hsa-miR-361-5p, hsa-miR-574-5p, hsa-miR-589-5p, hsa-miR-361-3p, hsa-miR-342-5p, hsa-miR-335-3p, hsa-miR-671-5p, hsa-miR-2110, hsa-miR-320c, hsa-miR-874-3p, hsa-miR-1-3p, hsa-miR-320a-3p, hsa-miR-147b-3p
UBXN7	18	hsa-miR-335-5p, hsa-miR-339-5p, hsa-miR-148a-3p, hsa-miR-20b-5p, hsa-miR-7-5p, hsa-miR-138-5p, hsa-miR-361-5p, hsa-miR-574-5p, hsa-miR-589-5p, hsa-miR-320d, hsa-miR-342-5p, hsa-miR-335-3p, hsa-miR-671-5p, hsa-miR-2110, hsa-miR-320c, hsa-miR-1-3p, hsa-miR-133a-3p, hsa-miR-320a-3p

UGCG	18	hsa-miR-20b-5p, hsa-miR-335-5p, hsa-miR-335-3p, hsa-miR-148a-3p, hsa-miR-7-5p, hsa-miR-9-3p, hsa-miR-361-5p, hsa-miR-574-5p, hsa-miR-589-5p, hsa-miR-320d, hsa-miR-500a-3p, hsa-miR-671-5p, hsa-miR-1307-5p, hsa-miR-320c, hsa-miR-378i, hsa-miR-1-3p, hsa-miR-133a-3p, hsa-miR-320a-3p
USP31	18	hsa-miR-7-5p, hsa-miR-148a-3p, hsa-miR-20b-5p, hsa-miR-335-5p, hsa-miR-9-3p, hsa-miR-361-5p, hsa-miR-574-5p, hsa-miR-589-5p, hsa-miR-320d, hsa-miR-1275, hsa-miR-342-5p, hsa-miR-335-3p, hsa-miR-425-3p, hsa-miR-500a-3p, hsa-miR-320c, hsa-miR-874-3p, hsa-miR-1-3p, hsa-miR-320a-3p
VCPIP1	18	hsa-miR-361-5p, hsa-miR-148a-3p, hsa-miR-20b-5p, hsa-miR-335-5p, hsa-miR-7-5p, hsa-miR-589-5p, hsa-miR-1275, hsa-miR-335-3p, hsa-miR-425-3p, hsa-miR-500a-3p, hsa-miR-671-5p, hsa-miR-1307-3p, hsa-miR-2110, hsa-miR-320c, hsa-miR-378i, hsa-miR-1-3p, hsa-miR-320a-3p, hsa-miR-147b-3p
WDFY3	18	hsa-miR-148a-3p, hsa-miR-20b-5p, hsa-miR-335-5p, hsa-miR-7-5p, hsa-miR-9-3p, hsa-miR-361-5p, hsa-miR-574-5p, hsa-miR-589-5p, hsa-miR-769-5p, hsa-miR-188-5p, hsa-miR-361-3p, hsa-miR-342-5p, hsa-miR-500a-3p, hsa-miR-671-5p, hsa-miR-320c, hsa-miR-378i, hsa-miR-1-3p, hsa-miR-320a-3p
ZBTB10	18	hsa-miR-335-5p, hsa-miR-361-5p, hsa-miR-20b-5p, hsa-miR-7-5p, hsa-miR-9-3p, hsa-miR-138-5p, hsa-miR-339-5p, hsa-miR-574-5p, hsa-miR-589-5p, hsa-miR-1275, hsa-miR-335-3p, hsa-miR-500a-3p, hsa-miR-671-5p, hsa-miR-320c, hsa-miR-3615, hsa-miR-1-3p, hsa-miR-133a-3p, hsa-miR-320a-3p
ZBTB4	18	hsa-miR-20b-5p, hsa-miR-671-5p, hsa-miR-148a-3p, hsa-miR-7-5p, hsa-miR-138-5p, hsa-miR-361-5p, hsa-miR-339-5p, hsa-miR-574-5p, hsa-miR-769-5p, hsa-miR-1275, hsa-miR-629-5p, hsa-miR-29c-5p, hsa-miR-500a-3p, hsa-miR-2110, hsa-miR-3615, hsa-miR-378i, hsa-miR-320a-3p, hsa-miR-147b-3p
ZBTB41	18	hsa-miR-148a-3p, hsa-miR-20b-5p, hsa-miR-335-5p, hsa-miR-9-3p, hsa-miR-138-5p, hsa-miR-361-5p, hsa-miR-574-5p, hsa-miR-320d, hsa-miR-629-5p, hsa-miR-29c-5p, hsa-miR-342-5p, hsa-miR-335-3p, hsa-miR-3168, hsa-miR-2110, hsa-miR-320c, hsa-miR-1-3p, hsa-miR-887-3p, hsa-miR-320a-3p

ZFP91	18	hsa-miR-4516, hsa-miR-148a-3p, hsa-miR-20b-5p, hsa-miR-335-5p, hsa-miR-7-5p, hsa-miR-9-3p, hsa-miR-138-5p, hsa-miR-361-5p, hsa-miR-574-5p, hsa-miR-589-5p, hsa-miR-769-5p, hsa-miR-188-5p, hsa-miR-320d, hsa-miR-361-3p, hsa-miR-671-5p, hsa-miR-320c, hsa-miR-1-3p, hsa-miR-320a-3p
ZNF451	18	hsa-miR-335-5p, hsa-miR-9-3p, hsa-miR-320d, hsa-miR-320c, hsa-miR-148a-3p, hsa-miR-20b-5p, hsa-miR-7-5p, hsa-miR-138-5p, hsa-miR-589-5p, hsa-miR-342-5p, hsa-miR-335-3p, hsa-miR-425-3p, hsa-miR-671-5p, hsa-miR-2110, hsa-miR-378i, hsa-miR-1-3p, hsa-miR-320a-3p, hsa-miR-147b-3p
ZNF462	18	hsa-miR-148a-3p, hsa-miR-20b-5p, hsa-miR-335-5p, hsa-miR-7-5p, hsa-miR-9-3p, hsa-miR-361-5p, hsa-miR-339-5p, hsa-miR-574-5p, hsa-miR-769-5p, hsa-miR-629-5p, hsa-miR-342-5p, hsa-miR-335-3p, hsa-miR-500a-3p, hsa-miR-671-5p, hsa-miR-2110, hsa-miR-320c, hsa-miR-320a-3p, hsa-miR-147b-3p
ZNF644	18	hsa-miR-148a-3p, hsa-miR-20b-5p, hsa-miR-335-5p, hsa-miR-7-5p, hsa-miR-361-5p, hsa-miR-769-5p, hsa-miR-629-5p, hsa-miR-335-3p, hsa-miR-500a-3p, hsa-miR-671-5p, hsa-miR-2110, hsa-miR-3615, hsa-miR-378i, hsa-miR-874-3p, hsa-miR-1-3p, hsa-miR-133a-3p, hsa-miR-887-3p, hsa-miR-320a-3p
ZNF652	18	hsa-miR-20b-5p, hsa-miR-769-5p, hsa-miR-1307-3p, hsa-miR-148a-3p, hsa-miR-7-5p, hsa-miR-138-5p, hsa-miR-361-5p, hsa-miR-320d, hsa-miR-629-5p, hsa-miR-342-5p, hsa-miR-335-3p, hsa-miR-500a-3p, hsa-miR-671-5p, hsa-miR-2110, hsa-miR-378i, hsa-miR-320a-3p, hsa-miR-147b-3p, hsa-miR-190b-5p
ABHD2	17	hsa-miR-20b-5p, hsa-miR-335-5p, hsa-miR-1307-3p, hsa-miR-148a-3p, hsa-miR-7-5p, hsa-miR-138-5p, hsa-miR-361-5p, hsa-miR-589-5p, hsa-miR-769-5p, hsa-miR-1275, hsa-miR-342-5p, hsa-miR-335-3p, hsa-miR-425-3p, hsa-miR-671-5p, hsa-miR-320c, hsa-miR-1-3p, hsa-miR-320a-3p
ACLY	17	hsa-miR-361-5p, hsa-miR-148a-3p, hsa-miR-20b-5p, hsa-miR-7-5p, hsa-miR-138-5p, hsa-miR-589-5p, hsa-miR-769-5p, hsa-miR-29c-5p, hsa-miR-361-3p, hsa-miR-342-5p, hsa-miR-335-3p, hsa-miR-671-5p, hsa-miR-3615, hsa-miR-378i, hsa-miR-1-3p, hsa-miR-133a-3p, hsa-miR-147b-3p

AGFG1	17	hsa-miR-148a-3p, hsa-miR-20b-5p, hsa-miR-335-5p, hsa-miR-7-5p, hsa-miR-9-3p, hsa-miR-138-5p, hsa-miR-361-5p, hsa-miR-574-5p, hsa-miR-589-5p, hsa-miR-320d, hsa-miR-629-5p, hsa-miR-500a-3p, hsa-miR-2110, hsa-miR-320c, hsa-miR-1-3p, hsa-miR-320a-3p, hsa-miR-147b-3p
AHNAK	17	hsa-miR-335-5p, hsa-miR-148a-3p, hsa-miR-20b-5p, hsa-miR-7-5p, hsa-miR-138-5p, hsa-miR-361-5p, hsa-miR-574-5p, hsa-miR-769-5p, hsa-miR-335-3p, hsa-miR-425-3p, hsa-miR-500a-3p, hsa-miR-671-5p, hsa-miR-1307-3p, hsa-miR-3615, hsa-miR-378i, hsa-miR-320a-3p, hsa-miR-147b-3p
AKIRIN1	17	hsa-miR-148a-3p, hsa-miR-20b-5p, hsa-miR-7-5p, hsa-miR-9-3p, hsa-miR-138-5p, hsa-miR-361-5p, hsa-miR-339-5p, hsa-miR-574-5p, hsa-miR-589-5p, hsa-miR-769-5p, hsa-miR-500a-3p, hsa-miR-1307-5p, hsa-miR-2110, hsa-miR-320c, hsa-miR-320a-3p, hsa-miR-147b-3p, hsa-miR-190b-5p
APLP2	17	hsa-miR-148a-3p, hsa-miR-7-5p, hsa-miR-4516, hsa-miR-20b-5p, hsa-miR-138-5p, hsa-miR-361-5p, hsa-miR-320d, hsa-miR-361-3p, hsa-miR-335-3p, hsa-miR-500a-3p, hsa-miR-671-5p, hsa-miR-2110, hsa-miR-320c, hsa-miR-3615, hsa-miR-1-3p, hsa-miR-320a-3p, hsa-miR-147b-3p
ARID5B	17	hsa-miR-188-5p, hsa-miR-320d, hsa-miR-320c, hsa-miR-148a-3p, hsa-miR-20b-5p, hsa-miR-7-5p, hsa-miR-138-5p, hsa-miR-769-5p, hsa-miR-629-5p, hsa-miR-342-5p, hsa-miR-335-3p, hsa-miR-500a-3p, hsa-miR-671-5p, hsa-miR-2110, hsa-miR-378i, hsa-miR-133a-3p, hsa-miR-320a-3p
ARL8B	17	hsa-miR-148a-3p, hsa-miR-378i, hsa-miR-20b-5p, hsa-miR-7-5p, hsa-miR-9-3p, hsa-miR-574-5p, hsa-miR-589-5p, hsa-miR-629-5p, hsa-miR-335-3p, hsa-miR-425-3p, hsa-miR-671-5p, hsa-miR-2110, hsa-miR-320c, hsa-miR-3615, hsa-miR-1-3p, hsa-miR-320a-3p, hsa-miR-147b-3p
ATXN7L3B	17	hsa-miR-20b-5p, hsa-miR-320c, hsa-miR-148a-3p, hsa-miR-335-5p, hsa-miR-7-5p, hsa-miR-574-5p, hsa-miR-589-5p, hsa-miR-188-5p, hsa-miR-320d, hsa-miR-1275, hsa-miR-629-5p, hsa-miR-342-5p, hsa-miR-335-3p, hsa-miR-500a-3p, hsa-miR-671-5p, hsa-miR-2110, hsa-miR-320a-3p
BHLHE40	17	hsa-miR-335-5p, hsa-miR-148a-3p, hsa-miR-20b-5p, hsa-miR-7-5p, hsa-miR-138-5p, hsa-miR-361-5p, hsa-miR-574-5p, hsa-miR-589-5p, hsa-miR-769-5p, hsa-miR-320d, hsa-miR-629-5p, hsa-miR-335-3p, hsa-miR-425-3p, hsa-miR-500a-3p, hsa-miR-320c, hsa-miR-887-3p, hsa-miR-320a-3p

BRD4	17	hsa-miR-2110, hsa-miR-148a-3p, hsa-miR-20b-5p, hsa-miR-335-5p, hsa-miR-7-5p, hsa-miR-138-5p, hsa-miR-589-5p, hsa-miR-769-5p, hsa-miR-320d, hsa-miR-629-5p, hsa-miR-342-5p, hsa-miR-335-3p, hsa-miR-500a-3p, hsa-miR-1307-3p, hsa-miR-1-3p, hsa-miR-320a-3p, hsa-miR-147b-3p
BTBD3	17	hsa-miR-148a-3p, hsa-miR-20b-5p, hsa-miR-7-5p, hsa-miR-9-3p, hsa-miR-361-5p, hsa-miR-769-5p, hsa-miR-320d, hsa-miR-425-3p, hsa-miR-500a-3p, hsa-miR-2110, hsa-miR-320c, hsa-miR-378i, hsa-miR-1-3p, hsa-miR-887-3p, hsa-miR-320a-3p, hsa-miR-147b-3p, hsa-miR-190b-5p
CAPZA1	17	hsa-miR-7-5p, hsa-miR-9-3p, hsa-miR-4516, hsa-miR-1-3p, hsa-miR-20b-5p, hsa-miR-138-5p, hsa-miR-361-5p, hsa-miR-574-5p, hsa-miR-589-5p, hsa-miR-629-5p, hsa-miR-342-5p, hsa-miR-335-3p, hsa-miR-500a-3p, hsa-miR-671-5p, hsa-miR-2110, hsa-miR-320a-3p, hsa-miR-147b-3p
CDKN1A	17	hsa-miR-148a-3p, hsa-miR-20b-5p, hsa-miR-335-5p, hsa-miR-574-5p, hsa-miR-4516, hsa-miR-133a-3p, hsa-miR-7704, hsa-miR-7-5p, hsa-miR-589-5p, hsa-miR-769-5p, hsa-miR-629-5p, hsa-miR-342-5p, hsa-miR-335-3p, hsa-miR-671-5p, hsa-miR-1307-5p, hsa-miR-2110, hsa-miR-887-3p
CELSR2	17	hsa-miR-148a-3p, hsa-miR-20b-5p, hsa-miR-335-5p, hsa-miR-7-5p, hsa-miR-138-5p, hsa-miR-361-5p, hsa-miR-574-5p, hsa-miR-589-5p, hsa-miR-769-5p, hsa-miR-629-5p, hsa-miR-342-5p, hsa-miR-500a-3p, hsa-miR-2110, hsa-miR-320c, hsa-miR-133a-3p, hsa-miR-320a-3p, hsa-miR-147b-3p
CEP350	17	hsa-miR-148a-3p, hsa-miR-20b-5p, hsa-miR-335-5p, hsa-miR-7-5p, hsa-miR-138-5p, hsa-miR-361-5p, hsa-miR-574-5p, hsa-miR-589-5p, hsa-miR-769-5p, hsa-miR-629-5p, hsa-miR-335-3p, hsa-miR-500a-3p, hsa-miR-320c, hsa-miR-378i, hsa-miR-1-3p, hsa-miR-133a-3p, hsa-miR-320a-3p
CLIC4	17	hsa-miR-20b-5p, hsa-miR-335-5p, hsa-miR-148a-3p, hsa-miR-7-5p, hsa-miR-138-5p, hsa-miR-574-5p, hsa-miR-1275, hsa-miR-629-5p, hsa-miR-342-5p, hsa-miR-500a-3p, hsa-miR-675-3p, hsa-miR-2110, hsa-miR-320c, hsa-miR-1468-5p, hsa-miR-1-3p, hsa-miR-133a-3p, hsa-miR-320a-3p
CNTNAP1	17	hsa-miR-148a-3p, hsa-miR-20b-5p, hsa-miR-335-5p, hsa-miR-7-5p, hsa-miR-138-5p, hsa-miR-361-5p, hsa-miR-339-5p, hsa-miR-574-5p, hsa-

		miR-589-5p, hsa-miR-188-5p, hsa-miR-500a-3p, hsa-miR-320c, hsa-miR-1-3p, hsa-miR-133a-3p, hsa-miR-887-3p, hsa-miR-320a-3p, hsa-miR-147b-3p
COPA	17	hsa-miR-335-5p, hsa-miR-589-5p, hsa-miR-148a-3p, hsa-miR-20b-5p, hsa-miR-7-5p, hsa-miR-138-5p, hsa-miR-361-5p, hsa-miR-339-5p, hsa-miR-769-5p, hsa-miR-361-3p, hsa-miR-335-3p, hsa-miR-320c, hsa-miR-378i, hsa-miR-1-3p, hsa-miR-320a-3p, hsa-miR-147b-3p, hsa-miR-190b-5p
CRKL	17	hsa-miR-335-5p, hsa-miR-7-5p, hsa-miR-148a-3p, hsa-miR-20b-5p, hsa-miR-138-5p, hsa-miR-361-5p, hsa-miR-574-5p, hsa-miR-589-5p, hsa-miR-769-5p, hsa-miR-320d, hsa-miR-1275, hsa-miR-335-3p, hsa-miR-3168, hsa-miR-320c, hsa-miR-1-3p, hsa-miR-320a-3p, hsa-miR-147b-3p
CTNND1	17	hsa-miR-335-5p, hsa-miR-148a-3p, hsa-miR-20b-5p, hsa-miR-7-5p, hsa-miR-574-5p, hsa-miR-769-5p, hsa-miR-320d, hsa-miR-361-3p, hsa-miR-335-3p, hsa-miR-425-3p, hsa-miR-500a-3p, hsa-miR-671-5p, hsa-miR-320c, hsa-miR-874-3p, hsa-miR-1-3p, hsa-miR-133a-3p, hsa-miR-320a-3p
DAZAP1	17	hsa-miR-148a-3p, hsa-miR-20b-5p, hsa-miR-7-5p, hsa-miR-9-3p, hsa-miR-138-5p, hsa-miR-339-5p, hsa-miR-320d, hsa-miR-361-3p, hsa-miR-425-3p, hsa-miR-671-5p, hsa-miR-2110, hsa-miR-320c, hsa-miR-3615, hsa-miR-874-3p, hsa-miR-1-3p, hsa-miR-320a-3p, hsa-miR-147b-3p
DCAF7	17	hsa-miR-7-5p, hsa-miR-335-3p, hsa-miR-4516, hsa-miR-148a-3p, hsa-miR-20b-5p, hsa-miR-335-5p, hsa-miR-138-5p, hsa-miR-361-5p, hsa-miR-339-5p, hsa-miR-629-5p, hsa-miR-342-5p, hsa-miR-500a-3p, hsa-miR-2110, hsa-miR-378i, hsa-miR-1-3p, hsa-miR-320a-3p, hsa-miR-147b-3p
DCBLD2	17	hsa-miR-20b-5p, hsa-miR-148a-3p, hsa-miR-335-5p, hsa-miR-7-5p, hsa-miR-9-3p, hsa-miR-138-5p, hsa-miR-769-5p, hsa-miR-320d, hsa-miR-29c-5p, hsa-miR-335-3p, hsa-miR-671-5p, hsa-miR-320c, hsa-miR-378i, hsa-miR-874-3p, hsa-miR-1-3p, hsa-miR-320a-3p, hsa-miR-147b-3p
DDX24	17	hsa-miR-769-5p, hsa-miR-148a-3p, hsa-miR-20b-5p, hsa-miR-335-5p, hsa-miR-7-5p, hsa-miR-138-5p, hsa-miR-361-5p, hsa-miR-574-5p, hsa-miR-589-5p, hsa-miR-335-3p, hsa-miR-425-3p, hsa-miR-500a-3p, hsa-miR-671-5p, hsa-miR-320c, hsa-miR-3615, hsa-miR-378i, hsa-miR-320a-3p

DDX39B	17	hsa-miR-342-5p, hsa-miR-671-5p, hsa-miR-148a-3p, hsa-miR-20b-5p, hsa-miR-335-5p, hsa-miR-7-5p, hsa-miR-361-5p, hsa-miR-361-3p, hsa-miR-335-3p, hsa-miR-500a-3p, hsa-miR-1307-3p, hsa-miR-1307-5p, hsa-miR-320c, hsa-miR-378i, hsa-miR-1468-5p, hsa-miR-320a-3p, hsa-miR-147b-3p
DKK1	17	hsa-miR-335-5p, hsa-miR-1-3p, hsa-miR-148a-3p, hsa-miR-20b-5p, hsa-miR-7-5p, hsa-miR-9-3p, hsa-miR-138-5p, hsa-miR-361-5p, hsa-miR-574-5p, hsa-miR-589-5p, hsa-miR-769-5p, hsa-miR-629-5p, hsa-miR-335-3p, hsa-miR-671-5p, hsa-miR-320c, hsa-miR-3615, hsa-miR-320a-3p
DSP	17	hsa-miR-148a-3p, hsa-miR-20b-5p, hsa-miR-335-5p, hsa-miR-7-5p, hsa-miR-361-5p, hsa-miR-339-5p, hsa-miR-589-5p, hsa-miR-769-5p, hsa-miR-361-3p, hsa-miR-335-3p, hsa-miR-500a-3p, hsa-miR-2110, hsa-miR-320c, hsa-miR-3615, hsa-miR-1-3p, hsa-miR-133a-3p, hsa-miR-320a-3p
DUSP16	17	hsa-miR-335-5p, hsa-miR-20b-5p, hsa-miR-7-5p, hsa-miR-138-5p, hsa-miR-361-5p, hsa-miR-589-5p, hsa-miR-769-5p, hsa-miR-629-5p, hsa-miR-361-3p, hsa-miR-335-3p, hsa-miR-425-3p, hsa-miR-2110, hsa-miR-320c, hsa-miR-378i, hsa-miR-1468-5p, hsa-miR-887-3p, hsa-miR-320a-3p
DYNLL2	17	hsa-miR-148a-3p, hsa-miR-20b-5p, hsa-miR-335-5p, hsa-miR-9-3p, hsa-miR-361-5p, hsa-miR-589-5p, hsa-miR-769-5p, hsa-miR-188-5p, hsa-miR-342-5p, hsa-miR-335-3p, hsa-miR-551b-3p, hsa-miR-1307-5p, hsa-miR-2110, hsa-miR-378i, hsa-miR-874-3p, hsa-miR-1-3p, hsa-miR-320a-3p
E2F3	17	hsa-miR-20b-5p, hsa-miR-2110, hsa-miR-874-3p, hsa-miR-148a-3p, hsa-miR-7-5p, hsa-miR-9-3p, hsa-miR-138-5p, hsa-miR-589-5p, hsa-miR-320d, hsa-miR-342-5p, hsa-miR-335-3p, hsa-miR-671-5p, hsa-miR-320c, hsa-miR-3615, hsa-miR-1-3p, hsa-miR-320a-3p, hsa-miR-147b-3p
EEA1	17	hsa-miR-20b-5p, hsa-miR-335-5p, hsa-miR-574-5p, hsa-miR-148a-3p, hsa-miR-7-5p, hsa-miR-9-3p, hsa-miR-138-5p, hsa-miR-589-5p, hsa-miR-335-3p, hsa-miR-671-5p, hsa-miR-2110, hsa-miR-320c, hsa-miR-3615, hsa-miR-874-3p, hsa-miR-133a-3p, hsa-miR-887-3p, hsa-miR-320a-3p
EFTUD2	17	hsa-miR-1-3p, hsa-miR-20b-5p, hsa-miR-335-5p, hsa-miR-7-5p, hsa-miR-9-3p, hsa-miR-138-5p, hsa-miR-361-5p, hsa-miR-589-5p, hsa-miR-

		320d, hsa-miR-361-3p, hsa-miR-335-3p, hsa-miR-500a-3p, hsa-miR-671-5p, hsa-miR-320c, hsa-miR-133a-3p, hsa-miR-320a-3p, hsa-miR-147b-3p
ELAVL1	17	hsa-miR-148a-3p, hsa-miR-20b-5p, hsa-miR-7-5p, hsa-miR-9-3p, hsa-miR-138-5p, hsa-miR-361-5p, hsa-miR-1275, hsa-miR-342-5p, hsa-miR-335-3p, hsa-miR-671-5p, hsa-miR-2110, hsa-miR-320c, hsa-miR-3615, hsa-miR-133a-3p, hsa-miR-887-3p, hsa-miR-320a-3p, hsa-miR-190b-5p
ELOA	17	hsa-miR-769-5p, hsa-miR-148a-3p, hsa-miR-20b-5p, hsa-miR-335-5p, hsa-miR-7-5p, hsa-miR-138-5p, hsa-miR-361-5p, hsa-miR-320d, hsa-miR-629-5p, hsa-miR-342-5p, hsa-miR-335-3p, hsa-miR-500a-3p, hsa-miR-671-5p, hsa-miR-320c, hsa-miR-3615, hsa-miR-320a-3p, hsa-miR-147b-3p
ENC1	17	hsa-miR-148a-3p, hsa-miR-20b-5p, hsa-miR-335-5p, hsa-miR-7-5p, hsa-miR-138-5p, hsa-miR-361-5p, hsa-miR-574-5p, hsa-miR-769-5p, hsa-miR-1275, hsa-miR-335-3p, hsa-miR-500a-3p, hsa-miR-671-5p, hsa-miR-2110, hsa-miR-320c, hsa-miR-378i, hsa-miR-133a-3p, hsa-miR-320a-3p
ETV3	17	hsa-miR-1275, hsa-miR-148a-3p, hsa-miR-20b-5p, hsa-miR-335-5p, hsa-miR-7-5p, hsa-miR-138-5p, hsa-miR-574-5p, hsa-miR-589-5p, hsa-miR-769-5p, hsa-miR-629-5p, hsa-miR-335-3p, hsa-miR-425-3p, hsa-miR-500a-3p, hsa-miR-671-5p, hsa-miR-378i, hsa-miR-1-3p, hsa-miR-320a-3p
FAT3	17	hsa-miR-148a-3p, hsa-miR-20b-5p, hsa-miR-335-5p, hsa-miR-7-5p, hsa-miR-138-5p, hsa-miR-361-5p, hsa-miR-188-5p, hsa-miR-629-5p, hsa-miR-361-3p, hsa-miR-342-5p, hsa-miR-335-3p, hsa-miR-500a-3p, hsa-miR-671-5p, hsa-miR-320c, hsa-miR-1-3p, hsa-miR-887-3p, hsa-miR-320a-3p
FBXL18	17	hsa-miR-342-5p, hsa-miR-4497, hsa-miR-148a-3p, hsa-miR-20b-5p, hsa-miR-335-5p, hsa-miR-7-5p, hsa-miR-138-5p, hsa-miR-361-5p, hsa-miR-339-5p, hsa-miR-589-5p, hsa-miR-769-5p, hsa-miR-500a-3p, hsa-miR-1307-5p, hsa-miR-320c, hsa-miR-3615, hsa-miR-320a-3p, hsa-miR-147b-3p
FNDC3B	17	hsa-miR-335-5p, hsa-miR-9-3p, hsa-miR-1-3p, hsa-miR-20b-5p, hsa-miR-7-5p, hsa-miR-138-5p, hsa-miR-361-5p, hsa-miR-574-5p, hsa-miR-320d, hsa-miR-629-5p, hsa-miR-342-5p, hsa-miR-335-3p, hsa-miR-

		500a-3p, hsa-miR-3168, hsa-miR-320c, hsa-miR-320a-3p, hsa-miR-147b-3p
FOXK2	17	hsa-miR-148a-3p, hsa-miR-20b-5p, hsa-miR-7-5p, hsa-miR-769-5p, hsa-miR-335-5p, hsa-miR-138-5p, hsa-miR-361-5p, hsa-miR-574-5p, hsa-miR-589-5p, hsa-miR-1275, hsa-miR-629-5p, hsa-miR-335-3p, hsa-miR-425-3p, hsa-miR-320c, hsa-miR-1-3p, hsa-miR-320a-3p, hsa-miR-147b-3p
FOXN2	17	hsa-miR-188-5p, hsa-miR-4791, hsa-miR-148a-3p, hsa-miR-20b-5p, hsa-miR-335-5p, hsa-miR-7-5p, hsa-miR-138-5p, hsa-miR-361-5p, hsa-miR-1275, hsa-miR-629-5p, hsa-miR-342-5p, hsa-miR-335-3p, hsa-miR-500a-3p, hsa-miR-320c, hsa-miR-1-3p, hsa-miR-887-3p, hsa-miR-320a-3p
GAPVD1	17	hsa-miR-148a-3p, hsa-miR-20b-5p, hsa-miR-7-5p, hsa-miR-9-3p, hsa-miR-138-5p, hsa-miR-361-5p, hsa-miR-574-5p, hsa-miR-589-5p, hsa-miR-629-5p, hsa-miR-29c-5p, hsa-miR-335-3p, hsa-miR-425-3p, hsa-miR-500a-3p, hsa-miR-671-5p, hsa-miR-378i, hsa-miR-1468-5p, hsa-miR-320a-3p
GNAI2	17	hsa-miR-138-5p, hsa-miR-1-3p, hsa-miR-148a-3p, hsa-miR-20b-5p, hsa-miR-7-5p, hsa-miR-361-5p, hsa-miR-339-5p, hsa-miR-589-5p, hsa-miR-320d, hsa-miR-361-3p, hsa-miR-425-3p, hsa-miR-671-5p, hsa-miR-2110, hsa-miR-320c, hsa-miR-378i, hsa-miR-320a-3p, hsa-miR-147b-3p
GNS	17	hsa-miR-20b-5p, hsa-miR-335-5p, hsa-miR-148a-3p, hsa-miR-9-3p, hsa-miR-361-5p, hsa-miR-574-5p, hsa-miR-769-5p, hsa-miR-629-5p, hsa-miR-29c-5p, hsa-miR-361-3p, hsa-miR-425-3p, hsa-miR-320c, hsa-miR-378i, hsa-miR-1-3p, hsa-miR-133a-3p, hsa-miR-887-3p, hsa-miR-320a-3p
HECTD4	17	hsa-miR-335-5p, hsa-miR-148a-3p, hsa-miR-20b-5p, hsa-miR-7-5p, hsa-miR-138-5p, hsa-miR-361-5p, hsa-miR-574-5p, hsa-miR-589-5p, hsa-miR-500a-3p, hsa-miR-2110, hsa-miR-320c, hsa-miR-378i, hsa-miR-1-3p, hsa-miR-133a-3p, hsa-miR-887-3p, hsa-miR-320a-3p, hsa-miR-147b-3p
HNRNPU	17	hsa-miR-7-5p, hsa-miR-4516, hsa-miR-1-3p, hsa-miR-148a-3p, hsa-miR-20b-5p, hsa-miR-335-5p, hsa-miR-320d, hsa-miR-1275, hsa-miR-629-5p, hsa-miR-361-3p, hsa-miR-335-3p, hsa-miR-500a-3p, hsa-miR-671-5p, hsa-miR-2110, hsa-miR-320c, hsa-miR-3615, hsa-miR-320a-3p

HSP90B1	17	hsa-miR-148a-3p, hsa-miR-335-5p, hsa-miR-4516, hsa-miR-1-3p, hsa-miR-20b-5p, hsa-miR-7-5p, hsa-miR-9-3p, hsa-miR-361-5p, hsa-miR-574-5p, hsa-miR-320d, hsa-miR-335-3p, hsa-miR-671-5p, hsa-miR-1307-5p, hsa-miR-2110, hsa-miR-3615, hsa-miR-378i, hsa-miR-320a-3p
HTT	17	hsa-miR-148a-3p, hsa-miR-20b-5p, hsa-miR-335-5p, hsa-miR-7-5p, hsa-miR-138-5p, hsa-miR-361-5p, hsa-miR-769-5p, hsa-miR-342-5p, hsa-miR-335-3p, hsa-miR-500a-3p, hsa-miR-1307-5p, hsa-miR-2110, hsa-miR-320c, hsa-miR-1-3p, hsa-miR-887-3p, hsa-miR-12136, hsa-miR-320a-3p
IRS1	17	hsa-miR-7-5p, hsa-miR-148a-3p, hsa-miR-20b-5p, hsa-miR-138-5p, hsa-miR-361-5p, hsa-miR-339-5p, hsa-miR-589-5p, hsa-miR-769-5p, hsa-miR-29c-5p, hsa-miR-361-3p, hsa-miR-342-5p, hsa-miR-500a-3p, hsa-miR-1307-3p, hsa-miR-1307-5p, hsa-miR-320c, hsa-miR-320a-3p, hsa-miR-147b-3p
JADE1	17	hsa-miR-342-5p, hsa-miR-148a-3p, hsa-miR-20b-5p, hsa-miR-7-5p, hsa-miR-9-3p, hsa-miR-138-5p, hsa-miR-361-5p, hsa-miR-574-5p, hsa-miR-320d, hsa-miR-629-5p, hsa-miR-425-3p, hsa-miR-500a-3p, hsa-miR-671-5p, hsa-miR-2110, hsa-miR-320c, hsa-miR-874-3p, hsa-miR-320a-3p
KALRN	17	hsa-miR-335-5p, hsa-miR-148a-3p, hsa-miR-20b-5p, hsa-miR-7-5p, hsa-miR-138-5p, hsa-miR-574-5p, hsa-miR-769-5p, hsa-miR-29c-5p, hsa-miR-500a-3p, hsa-miR-671-5p, hsa-miR-2110, hsa-miR-320c, hsa-miR-378i, hsa-miR-1-3p, hsa-miR-133a-3p, hsa-miR-320a-3p, hsa-miR-190b-5p
KANSL1	17	hsa-miR-148a-3p, hsa-miR-20b-5p, hsa-miR-335-5p, hsa-miR-7-5p, hsa-miR-9-3p, hsa-miR-138-5p, hsa-miR-361-5p, hsa-miR-769-5p, hsa-miR-629-5p, hsa-miR-29c-5p, hsa-miR-342-5p, hsa-miR-500a-3p, hsa-miR-671-5p, hsa-miR-1307-3p, hsa-miR-320c, hsa-miR-887-3p, hsa-miR-320a-3p
KDM5B	17	hsa-miR-335-5p, hsa-miR-148a-3p, hsa-miR-20b-5p, hsa-miR-7-5p, hsa-miR-138-5p, hsa-miR-574-5p, hsa-miR-320d, hsa-miR-335-3p, hsa-miR-500a-3p, hsa-miR-671-5p, hsa-miR-2110, hsa-miR-320c, hsa-miR-1-3p, hsa-miR-133a-3p, hsa-miR-887-3p, hsa-miR-320a-3p, hsa-miR-147b-3p
LARP4	17	hsa-miR-1-3p, hsa-miR-148a-3p, hsa-miR-20b-5p, hsa-miR-335-5p, hsa-miR-7-5p, hsa-miR-9-3p, hsa-miR-138-5p, hsa-miR-361-5p, hsa-miR-574-5p, hsa-miR-769-5p, hsa-miR-320d, hsa-miR-29c-5p, hsa-miR-

		500a-3p, hsa-miR-551b-3p, hsa-miR-320c, hsa-miR-320a-3p, hsa-miR-147b-3p
LDHA	17	hsa-miR-320c, hsa-miR-20b-5p, hsa-miR-335-5p, hsa-miR-7-5p, hsa-miR-138-5p, hsa-miR-361-5p, hsa-miR-574-5p, hsa-miR-589-5p, hsa-miR-769-5p, hsa-miR-361-3p, hsa-miR-335-3p, hsa-miR-425-3p, hsa-miR-500a-3p, hsa-miR-2110, hsa-miR-378i, hsa-miR-320a-3p, hsa-miR-190b-5p
LDLR	17	hsa-miR-148a-3p, hsa-miR-20b-5p, hsa-miR-335-5p, hsa-miR-1307-3p, hsa-miR-7704, hsa-miR-7-5p, hsa-miR-138-5p, hsa-miR-574-5p, hsa-miR-589-5p, hsa-miR-769-5p, hsa-miR-188-5p, hsa-miR-320d, hsa-miR-1275, hsa-miR-335-3p, hsa-miR-671-5p, hsa-miR-378i, hsa-miR-147b-3p
LEMD3	17	hsa-miR-335-5p, hsa-miR-1-3p, hsa-miR-20b-5p, hsa-miR-7-5p, hsa-miR-9-3p, hsa-miR-138-5p, hsa-miR-361-5p, hsa-miR-574-5p, hsa-miR-188-5p, hsa-miR-335-3p, hsa-miR-500a-3p, hsa-miR-671-5p, hsa-miR-675-3p, hsa-miR-2110, hsa-miR-320c, hsa-miR-378i, hsa-miR-320a-3p
LMTK2	17	hsa-miR-335-5p, hsa-miR-148a-3p, hsa-miR-20b-5p, hsa-miR-7-5p, hsa-miR-138-5p, hsa-miR-574-5p, hsa-miR-589-5p, hsa-miR-769-5p, hsa-miR-320d, hsa-miR-1275, hsa-miR-335-3p, hsa-miR-671-5p, hsa-miR-1307-5p, hsa-miR-320c, hsa-miR-874-3p, hsa-miR-1-3p, hsa-miR-320a-3p
LUC7L2	17	hsa-miR-7-5p, hsa-miR-335-3p, hsa-miR-1307-3p, hsa-miR-148a-3p, hsa-miR-20b-5p, hsa-miR-339-5p, hsa-miR-769-5p, hsa-miR-1275, hsa-miR-629-5p, hsa-miR-342-5p, hsa-miR-671-5p, hsa-miR-2110, hsa-miR-320c, hsa-miR-378i, hsa-miR-874-3p, hsa-miR-1-3p, hsa-miR-320a-3p
LUZP1	17	hsa-miR-7-5p, hsa-miR-148a-3p, hsa-miR-20b-5p, hsa-miR-138-5p, hsa-miR-361-5p, hsa-miR-589-5p, hsa-miR-769-5p, hsa-miR-342-5p, hsa-miR-335-3p, hsa-miR-500a-3p, hsa-miR-671-5p, hsa-miR-1307-3p, hsa-miR-2110, hsa-miR-320c, hsa-miR-1-3p, hsa-miR-320a-3p, hsa-miR-147b-3p
MAP2	17	hsa-miR-335-5p, hsa-miR-574-5p, hsa-miR-4516, hsa-miR-148a-3p, hsa-miR-20b-5p, hsa-miR-7-5p, hsa-miR-9-3p, hsa-miR-138-5p, hsa-miR-361-5p, hsa-miR-589-5p, hsa-miR-769-5p, hsa-miR-188-5p, hsa-miR-342-5p, hsa-miR-671-5p, hsa-miR-320c, hsa-miR-1-3p, hsa-miR-320a-3p

MICAL3	17	hsa-miR-148a-3p, hsa-miR-20b-5p, hsa-miR-335-5p, hsa-miR-7-5p, hsa-miR-138-5p, hsa-miR-361-5p, hsa-miR-339-5p, hsa-miR-574-5p, hsa-miR-769-5p, hsa-miR-335-3p, hsa-miR-500a-3p, hsa-miR-671-5p, hsa-miR-378i, hsa-miR-1-3p, hsa-miR-887-3p, hsa-miR-320a-3p, hsa-miR-147b-3p
MLEC	17	hsa-miR-148a-3p, hsa-miR-133a-3p, hsa-miR-20b-5p, hsa-miR-335-5p, hsa-miR-7-5p, hsa-miR-589-5p, hsa-miR-769-5p, hsa-miR-320d, hsa-miR-629-5p, hsa-miR-29c-5p, hsa-miR-361-3p, hsa-miR-335-3p, hsa-miR-500a-3p, hsa-miR-671-5p, hsa-miR-320c, hsa-miR-1-3p, hsa-miR-320a-3p
MTF1	17	hsa-miR-20b-5p, hsa-miR-148a-3p, hsa-miR-7-5p, hsa-miR-138-5p, hsa-miR-574-5p, hsa-miR-320d, hsa-miR-1275, hsa-miR-629-5p, hsa-miR-342-5p, hsa-miR-335-3p, hsa-miR-500a-3p, hsa-miR-671-5p, hsa-miR-2110, hsa-miR-378i, hsa-miR-1468-5p, hsa-miR-1-3p, hsa-miR-320a-3p
MTF2	17	hsa-miR-148a-3p, hsa-miR-20b-5p, hsa-miR-335-5p, hsa-miR-7-5p, hsa-miR-9-3p, hsa-miR-574-5p, hsa-miR-769-5p, hsa-miR-320d, hsa-miR-629-5p, hsa-miR-335-3p, hsa-miR-425-3p, hsa-miR-671-5p, hsa-miR-2110, hsa-miR-320c, hsa-miR-133a-3p, hsa-miR-320a-3p, hsa-miR-147b-3p
MYO5A	17	hsa-miR-574-5p, hsa-miR-148a-3p, hsa-miR-20b-5p, hsa-miR-7-5p, hsa-miR-138-5p, hsa-miR-339-5p, hsa-miR-320d, hsa-miR-29c-5p, hsa-miR-361-3p, hsa-miR-500a-3p, hsa-miR-2110, hsa-miR-320c, hsa-miR-378i, hsa-miR-1-3p, hsa-miR-320a-3p, hsa-miR-147b-3p, hsa-miR-190b-5p
NAV1	17	hsa-miR-7-5p, hsa-miR-574-5p, hsa-miR-148a-3p, hsa-miR-20b-5p, hsa-miR-335-5p, hsa-miR-138-5p, hsa-miR-361-5p, hsa-miR-769-5p, hsa-miR-188-5p, hsa-miR-342-5p, hsa-miR-671-5p, hsa-miR-1307-5p, hsa-miR-320c, hsa-miR-1-3p, hsa-miR-887-3p, hsa-miR-320a-3p, hsa-miR-147b-3p
NCAPD2	17	hsa-miR-20b-5p, hsa-miR-335-5p, hsa-miR-7-5p, hsa-miR-138-5p, hsa-miR-361-5p, hsa-miR-574-5p, hsa-miR-589-5p, hsa-miR-769-5p, hsa-miR-320d, hsa-miR-342-5p, hsa-miR-335-3p, hsa-miR-425-3p, hsa-miR-671-5p, hsa-miR-320c, hsa-miR-378i, hsa-miR-320a-3p, hsa-miR-147b-3p
NCOR1	17	hsa-miR-148a-3p, hsa-miR-20b-5p, hsa-miR-335-5p, hsa-miR-7-5p, hsa-miR-138-5p, hsa-miR-361-5p, hsa-miR-320d, hsa-miR-342-5p, hsa-miR-

		335-3p, hsa-miR-425-3p, hsa-miR-500a-3p, hsa-miR-671-5p, hsa-miR-2110, hsa-miR-320c, hsa-miR-378i, hsa-miR-320a-3p, hsa-miR-147b-3p
NIN	17	hsa-miR-20b-5p, hsa-miR-574-5p, hsa-miR-148a-3p, hsa-miR-7-5p, hsa-miR-138-5p, hsa-miR-589-5p, hsa-miR-320d, hsa-miR-629-5p, hsa-miR-342-5p, hsa-miR-335-3p, hsa-miR-500a-3p, hsa-miR-3168, hsa-miR-2110, hsa-miR-320c, hsa-miR-1468-5p, hsa-miR-1-3p, hsa-miR-320a-3p
NIPBL	17	hsa-miR-148a-3p, hsa-miR-20b-5p, hsa-miR-335-5p, hsa-miR-7-5p, hsa-miR-138-5p, hsa-miR-361-5p, hsa-miR-339-5p, hsa-miR-589-5p, hsa-miR-188-5p, hsa-miR-320d, hsa-miR-629-5p, hsa-miR-361-3p, hsa-miR-335-3p, hsa-miR-320c, hsa-miR-1-3p, hsa-miR-133a-3p, hsa-miR-320a-3p
NMT1	17	hsa-miR-148a-3p, hsa-miR-20b-5p, hsa-miR-335-5p, hsa-miR-7-5p, hsa-miR-138-5p, hsa-miR-589-5p, hsa-miR-769-5p, hsa-miR-320d, hsa-miR-629-5p, hsa-miR-335-3p, hsa-miR-1307-5p, hsa-miR-2110, hsa-miR-320c, hsa-miR-3615, hsa-miR-378i, hsa-miR-1-3p, hsa-miR-320a-3p
NOLC1	17	hsa-miR-148a-3p, hsa-miR-20b-5p, hsa-miR-335-5p, hsa-miR-7-5p, hsa-miR-361-5p, hsa-miR-589-5p, hsa-miR-769-5p, hsa-miR-629-5p, hsa-miR-361-3p, hsa-miR-335-3p, hsa-miR-425-3p, hsa-miR-500a-3p, hsa-miR-671-5p, hsa-miR-320c, hsa-miR-378i, hsa-miR-133a-3p, hsa-miR-320a-3p
NR3C1	17	hsa-miR-20b-5p, hsa-miR-138-5p, hsa-miR-148a-3p, hsa-miR-335-5p, hsa-miR-7-5p, hsa-miR-9-3p, hsa-miR-361-5p, hsa-miR-589-5p, hsa-miR-1275, hsa-miR-629-5p, hsa-miR-342-5p, hsa-miR-335-3p, hsa-miR-671-5p, hsa-miR-2110, hsa-miR-320c, hsa-miR-320a-3p, hsa-miR-147b-3p
NRP1	17	hsa-miR-148a-3p, hsa-miR-335-5p, hsa-miR-1-3p, hsa-miR-20b-5p, hsa-miR-7-5p, hsa-miR-9-3p, hsa-miR-361-5p, hsa-miR-574-5p, hsa-miR-589-5p, hsa-miR-320d, hsa-miR-361-3p, hsa-miR-335-3p, hsa-miR-425-3p, hsa-miR-500a-3p, hsa-miR-320c, hsa-miR-320a-3p, hsa-miR-147b-3p
NSD3	17	hsa-miR-148a-3p, hsa-miR-20b-5p, hsa-miR-335-5p, hsa-miR-7-5p, hsa-miR-138-5p, hsa-miR-361-5p, hsa-miR-574-5p, hsa-miR-629-5p, hsa-miR-361-3p, hsa-miR-425-3p, hsa-miR-500a-3p, hsa-miR-671-5p, hsa-miR-320c, hsa-miR-378i, hsa-miR-874-3p, hsa-miR-1-3p, hsa-miR-320a-3p

NUDT21	17	hsa-miR-1-3p, hsa-miR-148a-3p, hsa-miR-20b-5p, hsa-miR-7-5p, hsa-miR-138-5p, hsa-miR-361-5p, hsa-miR-574-5p, hsa-miR-589-5p, hsa-miR-320d, hsa-miR-335-3p, hsa-miR-500a-3p, hsa-miR-2110, hsa-miR-320c, hsa-miR-133a-3p, hsa-miR-320a-3p, hsa-miR-147b-3p, hsa-miR-190b-5p
PCBP2	17	hsa-miR-20b-5p, hsa-miR-7-5p, hsa-miR-138-5p, hsa-miR-361-5p, hsa-miR-589-5p, hsa-miR-769-5p, hsa-miR-629-5p, hsa-miR-425-3p, hsa-miR-500a-3p, hsa-miR-671-5p, hsa-miR-2110, hsa-miR-320c, hsa-miR-1-3p, hsa-miR-133a-3p, hsa-miR-887-3p, hsa-miR-320a-3p, hsa-miR-147b-3p
PIK3R1	17	hsa-miR-148a-3p, hsa-miR-20b-5p, hsa-miR-335-5p, hsa-miR-7-5p, hsa-miR-9-3p, hsa-miR-138-5p, hsa-miR-361-5p, hsa-miR-339-5p, hsa-miR-589-5p, hsa-miR-769-5p, hsa-miR-629-5p, hsa-miR-335-3p, hsa-miR-500a-3p, hsa-miR-671-5p, hsa-miR-320c, hsa-miR-1-3p, hsa-miR-320a-3p
PKN2	17	hsa-miR-148a-3p, hsa-miR-20b-5p, hsa-miR-335-5p, hsa-miR-7-5p, hsa-miR-9-3p, hsa-miR-138-5p, hsa-miR-361-5p, hsa-miR-574-5p, hsa-miR-589-5p, hsa-miR-629-5p, hsa-miR-342-5p, hsa-miR-335-3p, hsa-miR-500a-3p, hsa-miR-320c, hsa-miR-1-3p, hsa-miR-320a-3p, hsa-miR-147b-3p
PLAG1	17	hsa-miR-574-5p, hsa-miR-188-5p, hsa-miR-20b-5p, hsa-miR-7-5p, hsa-miR-589-5p, hsa-miR-320d, hsa-miR-342-5p, hsa-miR-335-3p, hsa-miR-500a-3p, hsa-miR-671-5p, hsa-miR-2110, hsa-miR-320c, hsa-miR-378i, hsa-miR-1-3p, hsa-miR-887-3p, hsa-miR-320a-3p, hsa-miR-147b-3p
PRPF19	17	hsa-miR-148a-3p, hsa-miR-20b-5p, hsa-miR-335-5p, hsa-miR-7-5p, hsa-miR-138-5p, hsa-miR-361-5p, hsa-miR-339-5p, hsa-miR-574-5p, hsa-miR-769-5p, hsa-miR-342-5p, hsa-miR-425-3p, hsa-miR-671-5p, hsa-miR-1307-5p, hsa-miR-1468-5p, hsa-miR-1-3p, hsa-miR-320a-3p, hsa-miR-147b-3p
PSMD3	17	hsa-miR-769-5p, hsa-miR-148a-3p, hsa-miR-20b-5p, hsa-miR-335-5p, hsa-miR-7-5p, hsa-miR-138-5p, hsa-miR-361-5p, hsa-miR-574-5p, hsa-miR-320d, hsa-miR-29c-5p, hsa-miR-361-3p, hsa-miR-425-3p, hsa-miR-500a-3p, hsa-miR-671-5p, hsa-miR-887-3p, hsa-miR-320a-3p, hsa-miR-147b-3p
PSME4	17	hsa-miR-148a-3p, hsa-miR-20b-5p, hsa-miR-335-5p, hsa-miR-7-5p, hsa-miR-361-5p, hsa-miR-574-5p, hsa-miR-589-5p, hsa-miR-188-5p, hsa-

		miR-1275, hsa-miR-335-3p, hsa-miR-500a-3p, hsa-miR-1307-3p, hsa-miR-378i, hsa-miR-1-3p, hsa-miR-133a-3p, hsa-miR-887-3p, hsa-miR-320a-3p
PTPRJ	17	hsa-miR-335-5p, hsa-miR-148a-3p, hsa-miR-20b-5p, hsa-miR-7-5p, hsa-miR-589-5p, hsa-miR-629-5p, hsa-miR-29c-5p, hsa-miR-361-3p, hsa-miR-342-5p, hsa-miR-335-3p, hsa-miR-425-3p, hsa-miR-500a-3p, hsa-miR-671-5p, hsa-miR-2110, hsa-miR-320c, hsa-miR-1-3p, hsa-miR-320a-3p
PXN	17	hsa-miR-335-5p, hsa-miR-148a-3p, hsa-miR-20b-5p, hsa-miR-7-5p, hsa-miR-138-5p, hsa-miR-574-5p, hsa-miR-589-5p, hsa-miR-769-5p, hsa-miR-1275, hsa-miR-629-5p, hsa-miR-361-3p, hsa-miR-500a-3p, hsa-miR-671-5p, hsa-miR-2110, hsa-miR-320c, hsa-miR-320a-3p, hsa-miR-147b-3p
RAB11FIP2	17	hsa-miR-1-3p, hsa-miR-148a-3p, hsa-miR-20b-5p, hsa-miR-335-5p, hsa-miR-7-5p, hsa-miR-9-3p, hsa-miR-361-5p, hsa-miR-769-5p, hsa-miR-320d, hsa-miR-629-5p, hsa-miR-361-3p, hsa-miR-500a-3p, hsa-miR-320c, hsa-miR-874-3p, hsa-miR-887-3p, hsa-miR-320a-3p, hsa-miR-147b-3p
RHOBTB3	17	hsa-miR-342-5p, hsa-miR-148a-3p, hsa-miR-20b-5p, hsa-miR-7-5p, hsa-miR-9-3p, hsa-miR-138-5p, hsa-miR-574-5p, hsa-miR-188-5p, hsa-miR-320d, hsa-miR-1275, hsa-miR-629-5p, hsa-miR-425-3p, hsa-miR-500a-3p, hsa-miR-320c, hsa-miR-1-3p, hsa-miR-133a-3p, hsa-miR-320a-3p
RIC1	17	hsa-miR-148a-3p, hsa-miR-20b-5p, hsa-miR-335-5p, hsa-miR-7-5p, hsa-miR-9-3p, hsa-miR-361-5p, hsa-miR-574-5p, hsa-miR-589-5p, hsa-miR-1275, hsa-miR-425-3p, hsa-miR-671-5p, hsa-miR-320c, hsa-miR-378i, hsa-miR-1-3p, hsa-miR-133a-3p, hsa-miR-887-3p, hsa-miR-320a-3p
RNF11	17	hsa-miR-7-5p, hsa-miR-1307-3p, hsa-miR-2110, hsa-miR-20b-5p, hsa-miR-335-5p, hsa-miR-138-5p, hsa-miR-574-5p, hsa-miR-769-5p, hsa-miR-361-3p, hsa-miR-335-3p, hsa-miR-500a-3p, hsa-miR-671-5p, hsa-miR-3168, hsa-miR-320c, hsa-miR-133a-3p, hsa-miR-320a-3p, hsa-miR-147b-3p
RPS6KA3	17	hsa-miR-9-3p, hsa-miR-148a-3p, hsa-miR-20b-5p, hsa-miR-7-5p, hsa-miR-138-5p, hsa-miR-361-5p, hsa-miR-339-5p, hsa-miR-574-5p, hsa-miR-589-5p, hsa-miR-320d, hsa-miR-342-5p, hsa-miR-335-3p, hsa-miR-671-5p, hsa-miR-2110, hsa-miR-320c, hsa-miR-1-3p, hsa-miR-320a-3p

RYR2	17	hsa-miR-335-5p, hsa-miR-148a-3p, hsa-miR-20b-5p, hsa-miR-7-5p, hsa-miR-138-5p, hsa-miR-589-5p, hsa-miR-769-5p, hsa-miR-425-3p, hsa-miR-500a-3p, hsa-miR-551b-3p, hsa-miR-671-5p, hsa-miR-2110, hsa-miR-1-3p, hsa-miR-133a-3p, hsa-miR-887-3p, hsa-miR-320a-3p, hsa-miR-190b-5p
SACS	17	hsa-miR-20b-5p, hsa-miR-148a-3p, hsa-miR-335-5p, hsa-miR-7-5p, hsa-miR-138-5p, hsa-miR-361-5p, hsa-miR-769-5p, hsa-miR-1275, hsa-miR-335-3p, hsa-miR-425-3p, hsa-miR-500a-3p, hsa-miR-671-5p, hsa-miR-3168, hsa-miR-2110, hsa-miR-874-3p, hsa-miR-1-3p, hsa-miR-320a-3p
SAMD4A	17	hsa-miR-148a-3p, hsa-miR-20b-5p, hsa-miR-335-5p, hsa-miR-7-5p, hsa-miR-138-5p, hsa-miR-361-5p, hsa-miR-339-5p, hsa-miR-574-5p, hsa-miR-589-5p, hsa-miR-335-3p, hsa-miR-500a-3p, hsa-miR-671-5p, hsa-miR-320c, hsa-miR-1-3p, hsa-miR-320a-3p, hsa-miR-147b-3p, hsa-miR-190b-5p
SET	17	hsa-miR-4516, hsa-miR-133a-3p, hsa-miR-148a-3p, hsa-miR-20b-5p, hsa-miR-335-5p, hsa-miR-361-5p, hsa-miR-339-5p, hsa-miR-589-5p, hsa-miR-769-5p, hsa-miR-335-3p, hsa-miR-500a-3p, hsa-miR-1307-3p, hsa-miR-2110, hsa-miR-320c, hsa-miR-378i, hsa-miR-1468-5p, hsa-miR-320a-3p
SF3B2	17	hsa-miR-148a-3p, hsa-miR-20b-5p, hsa-miR-335-5p, hsa-miR-138-5p, hsa-miR-361-5p, hsa-miR-339-5p, hsa-miR-361-3p, hsa-miR-342-5p, hsa-miR-335-3p, hsa-miR-500a-3p, hsa-miR-671-5p, hsa-miR-320c, hsa-miR-3615, hsa-miR-874-3p, hsa-miR-133a-3p, hsa-miR-320a-3p, hsa-miR-147b-3p
SIX4	17	hsa-miR-148a-3p, hsa-miR-20b-5p, hsa-miR-335-5p, hsa-miR-7-5p, hsa-miR-138-5p, hsa-miR-361-5p, hsa-miR-769-5p, hsa-miR-320d, hsa-miR-629-5p, hsa-miR-29c-5p, hsa-miR-335-3p, hsa-miR-500a-3p, hsa-miR-671-5p, hsa-miR-320c, hsa-miR-378i, hsa-miR-320a-3p, hsa-miR-147b-3p
SLC39A14	17	hsa-miR-1-3p, hsa-miR-148a-3p, hsa-miR-20b-5p, hsa-miR-335-5p, hsa-miR-7-5p, hsa-miR-9-3p, hsa-miR-138-5p, hsa-miR-361-5p, hsa-miR-339-5p, hsa-miR-574-5p, hsa-miR-342-5p, hsa-miR-335-3p, hsa-miR-671-5p, hsa-miR-320c, hsa-miR-3615, hsa-miR-378i, hsa-miR-320a-3p
SMAD2	17	hsa-miR-148a-3p, hsa-miR-361-5p, hsa-miR-188-5p, hsa-miR-20b-5p, hsa-miR-7-5p, hsa-miR-9-3p, hsa-miR-138-5p, hsa-miR-574-5p, hsa-miR-589-5p, hsa-miR-769-5p, hsa-miR-629-5p, hsa-miR-342-5p, hsa-

		miR-335-3p, hsa-miR-500a-3p, hsa-miR-671-5p, hsa-miR-3168, hsa-miR-1-3p
SMARCA4	17	hsa-miR-342-5p, hsa-miR-20b-5p, hsa-miR-7-5p, hsa-miR-138-5p, hsa-miR-361-5p, hsa-miR-339-5p, hsa-miR-574-5p, hsa-miR-589-5p, hsa-miR-769-5p, hsa-miR-335-3p, hsa-miR-500a-3p, hsa-miR-1307-3p, hsa-miR-2110, hsa-miR-378i, hsa-miR-874-3p, hsa-miR-1-3p, hsa-miR-147b-3p
SMCR8	17	hsa-miR-574-5p, hsa-miR-148a-3p, hsa-miR-20b-5p, hsa-miR-7-5p, hsa-miR-138-5p, hsa-miR-361-5p, hsa-miR-589-5p, hsa-miR-769-5p, hsa-miR-629-5p, hsa-miR-335-3p, hsa-miR-671-5p, hsa-miR-2110, hsa-miR-320c, hsa-miR-378i, hsa-miR-1-3p, hsa-miR-320a-3p, hsa-miR-147b-3p
SND1	17	hsa-miR-361-5p, hsa-miR-4516, hsa-miR-148a-3p, hsa-miR-20b-5p, hsa-miR-335-5p, hsa-miR-138-5p, hsa-miR-339-5p, hsa-miR-589-5p, hsa-miR-769-5p, hsa-miR-188-5p, hsa-miR-335-3p, hsa-miR-500a-3p, hsa-miR-320c, hsa-miR-133a-3p, hsa-miR-887-3p, hsa-miR-320a-3p, hsa-miR-147b-3p
SORL1	17	hsa-miR-148a-3p, hsa-miR-20b-5p, hsa-miR-335-5p, hsa-miR-7-5p, hsa-miR-9-3p, hsa-miR-138-5p, hsa-miR-361-5p, hsa-miR-574-5p, hsa-miR-29c-5p, hsa-miR-361-3p, hsa-miR-335-3p, hsa-miR-425-3p, hsa-miR-671-5p, hsa-miR-320c, hsa-miR-1-3p, hsa-miR-133a-3p, hsa-miR-320a-3p
SP3	17	hsa-miR-148a-3p, hsa-miR-7-5p, hsa-miR-361-5p, hsa-miR-574-5p, hsa-miR-589-5p, hsa-miR-769-5p, hsa-miR-361-3p, hsa-miR-342-5p, hsa-miR-335-3p, hsa-miR-2110, hsa-miR-320c, hsa-miR-874-3p, hsa-miR-1-3p, hsa-miR-133a-3p, hsa-miR-887-3p, hsa-miR-320a-3p, hsa-miR-147b-3p
SRF	17	hsa-miR-1-3p, hsa-miR-148a-3p, hsa-miR-20b-5p, hsa-miR-335-5p, hsa-miR-7-5p, hsa-miR-574-5p, hsa-miR-769-5p, hsa-miR-188-5p, hsa-miR-629-5p, hsa-miR-342-5p, hsa-miR-500a-3p, hsa-miR-671-5p, hsa-miR-320c, hsa-miR-378i, hsa-miR-133a-3p, hsa-miR-887-3p, hsa-miR-320a-3p
SRRM1	17	hsa-miR-1-3p, hsa-miR-148a-3p, hsa-miR-20b-5p, hsa-miR-7-5p, hsa-miR-138-5p, hsa-miR-574-5p, hsa-miR-769-5p, hsa-miR-1275, hsa-miR-335-3p, hsa-miR-425-3p, hsa-miR-671-5p, hsa-miR-1307-3p, hsa-miR-1307-5p, hsa-miR-320c, hsa-miR-133a-3p, hsa-miR-320a-3p, hsa-miR-147b-3p

STK35	17	hsa-miR-2110, hsa-miR-1-3p, hsa-miR-148a-3p, hsa-miR-20b-5p, hsa-miR-335-5p, hsa-miR-7-5p, hsa-miR-9-3p, hsa-miR-138-5p, hsa-miR-361-5p, hsa-miR-769-5p, hsa-miR-320d, hsa-miR-1275, hsa-miR-342-5p, hsa-miR-500a-3p, hsa-miR-874-3p, hsa-miR-320a-3p, hsa-miR-147b-3p
SYMPK	17	hsa-miR-1-3p, hsa-miR-148a-3p, hsa-miR-20b-5p, hsa-miR-335-5p, hsa-miR-7-5p, hsa-miR-574-5p, hsa-miR-589-5p, hsa-miR-769-5p, hsa-miR-361-3p, hsa-miR-342-5p, hsa-miR-335-3p, hsa-miR-500a-3p, hsa-miR-671-5p, hsa-miR-1307-3p, hsa-miR-2110, hsa-miR-3615, hsa-miR-378i
SYNE2	17	hsa-miR-1-3p, hsa-miR-148a-3p, hsa-miR-20b-5p, hsa-miR-335-5p, hsa-miR-7-5p, hsa-miR-138-5p, hsa-miR-361-5p, hsa-miR-629-5p, hsa-miR-342-5p, hsa-miR-335-3p, hsa-miR-500a-3p, hsa-miR-671-5p, hsa-miR-1307-5p, hsa-miR-2110, hsa-miR-320c, hsa-miR-378i, hsa-miR-320a-3p
TCERG1	17	hsa-miR-769-5p, hsa-miR-148a-3p, hsa-miR-20b-5p, hsa-miR-335-5p, hsa-miR-7-5p, hsa-miR-361-5p, hsa-miR-339-5p, hsa-miR-589-5p, hsa-miR-188-5p, hsa-miR-335-3p, hsa-miR-425-3p, hsa-miR-500a-3p, hsa-miR-3615, hsa-miR-1468-5p, hsa-miR-1-3p, hsa-miR-320a-3p, hsa-miR-190b-5p
TEAD1	17	hsa-miR-148a-3p, hsa-miR-20b-5p, hsa-miR-335-5p, hsa-miR-7-5p, hsa-miR-9-3p, hsa-miR-361-5p, hsa-miR-339-5p, hsa-miR-574-5p, hsa-miR-589-5p, hsa-miR-769-5p, hsa-miR-629-5p, hsa-miR-335-3p, hsa-miR-500a-3p, hsa-miR-671-5p, hsa-miR-320c, hsa-miR-133a-3p, hsa-miR-320a-3p
TGFBR1	17	hsa-miR-148a-3p, hsa-miR-20b-5p, hsa-miR-7-5p, hsa-miR-9-3p, hsa-miR-138-5p, hsa-miR-361-5p, hsa-miR-589-5p, hsa-miR-769-5p, hsa-miR-629-5p, hsa-miR-335-3p, hsa-miR-500a-3p, hsa-miR-551b-3p, hsa-miR-675-3p, hsa-miR-2110, hsa-miR-320c, hsa-miR-1-3p, hsa-miR-320a-3p
TIMP2	17	hsa-miR-148a-3p, hsa-miR-20b-5p, hsa-miR-335-5p, hsa-miR-7-5p, hsa-miR-138-5p, hsa-miR-361-5p, hsa-miR-769-5p, hsa-miR-335-3p, hsa-miR-500a-3p, hsa-miR-671-5p, hsa-miR-320c, hsa-miR-378i, hsa-miR-874-3p, hsa-miR-1-3p, hsa-miR-133a-3p, hsa-miR-320a-3p, hsa-miR-147b-3p
TJP1	17	hsa-miR-148a-3p, hsa-miR-20b-5p, hsa-miR-335-5p, hsa-miR-7-5p, hsa-miR-138-5p, hsa-miR-361-5p, hsa-miR-339-5p, hsa-miR-629-5p, hsa-miR-335-3p, hsa-miR-425-3p, hsa-miR-500a-3p, hsa-miR-320c, hsa-

		miR-1-3p, hsa-miR-133a-3p, hsa-miR-887-3p, hsa-miR-320a-3p, hsa-miR-147b-3p
TMED10	17	hsa-miR-7-5p, hsa-miR-342-5p, hsa-miR-148a-3p, hsa-miR-20b-5p, hsa-miR-335-5p, hsa-miR-9-3p, hsa-miR-138-5p, hsa-miR-574-5p, hsa-miR-769-5p, hsa-miR-629-5p, hsa-miR-29c-5p, hsa-miR-361-3p, hsa-miR-335-3p, hsa-miR-671-5p, hsa-miR-1-3p, hsa-miR-320a-3p, hsa-miR-147b-3p
TP53INP2	17	hsa-miR-138-5p, hsa-miR-629-5p, hsa-miR-20b-5p, hsa-miR-335-5p, hsa-miR-7-5p, hsa-miR-361-5p, hsa-miR-574-5p, hsa-miR-769-5p, hsa-miR-320d, hsa-miR-342-5p, hsa-miR-425-3p, hsa-miR-500a-3p, hsa-miR-671-5p, hsa-miR-320c, hsa-miR-1-3p, hsa-miR-320a-3p, hsa-miR-147b-3p
TPX2	17	hsa-miR-148a-3p, hsa-miR-20b-5p, hsa-miR-335-5p, hsa-miR-7-5p, hsa-miR-138-5p, hsa-miR-361-5p, hsa-miR-589-5p, hsa-miR-188-5p, hsa-miR-629-5p, hsa-miR-342-5p, hsa-miR-335-3p, hsa-miR-500a-3p, hsa-miR-320c, hsa-miR-3615, hsa-miR-378i, hsa-miR-1-3p, hsa-miR-320a-3p
TRIM28	17	hsa-miR-342-5p, hsa-miR-148a-3p, hsa-miR-20b-5p, hsa-miR-335-5p, hsa-miR-7-5p, hsa-miR-138-5p, hsa-miR-589-5p, hsa-miR-769-5p, hsa-miR-335-3p, hsa-miR-500a-3p, hsa-miR-671-5p, hsa-miR-1307-3p, hsa-miR-1307-5p, hsa-miR-3615, hsa-miR-378i, hsa-miR-133a-3p, hsa-miR-320a-3p
TRIM33	17	hsa-miR-629-5p, hsa-miR-20b-5p, hsa-miR-7-5p, hsa-miR-138-5p, hsa-miR-361-5p, hsa-miR-29c-5p, hsa-miR-361-3p, hsa-miR-335-3p, hsa-miR-425-3p, hsa-miR-500a-3p, hsa-miR-2110, hsa-miR-320c, hsa-miR-874-3p, hsa-miR-1468-5p, hsa-miR-1-3p, hsa-miR-320a-3p, hsa-miR-147b-3p
TRIO	17	hsa-miR-148a-3p, hsa-miR-20b-5p, hsa-miR-335-5p, hsa-miR-7-5p, hsa-miR-138-5p, hsa-miR-361-5p, hsa-miR-769-5p, hsa-miR-320d, hsa-miR-29c-5p, hsa-miR-361-3p, hsa-miR-500a-3p, hsa-miR-671-5p, hsa-miR-2110, hsa-miR-320c, hsa-miR-1-3p, hsa-miR-133a-3p, hsa-miR-320a-3p
UBA1	17	hsa-miR-148a-3p, hsa-miR-20b-5p, hsa-miR-7-5p, hsa-miR-138-5p, hsa-miR-361-5p, hsa-miR-339-5p, hsa-miR-589-5p, hsa-miR-769-5p, hsa-miR-335-3p, hsa-miR-500a-3p, hsa-miR-671-5p, hsa-miR-2110, hsa-miR-320c, hsa-miR-378i, hsa-miR-1-3p, hsa-miR-320a-3p, hsa-miR-147b-3p

UBE2G1	17	hsa-miR-4516, hsa-miR-148a-3p, hsa-miR-20b-5p, hsa-miR-335-5p, hsa-miR-7-5p, hsa-miR-361-5p, hsa-miR-589-5p, hsa-miR-320d, hsa-miR-1275, hsa-miR-629-5p, hsa-miR-342-5p, hsa-miR-335-3p, hsa-miR-2110, hsa-miR-320c, hsa-miR-887-3p, hsa-miR-320a-3p, hsa-miR-147b-3p
USP9X	17	hsa-miR-339-5p, hsa-miR-148a-3p, hsa-miR-20b-5p, hsa-miR-335-5p, hsa-miR-7-5p, hsa-miR-361-5p, hsa-miR-589-5p, hsa-miR-629-5p, hsa-miR-342-5p, hsa-miR-500a-3p, hsa-miR-671-5p, hsa-miR-3168, hsa-miR-2110, hsa-miR-1-3p, hsa-miR-320a-3p, hsa-miR-147b-3p, hsa-miR-190b-5p
VPS13C	17	hsa-miR-20b-5p, hsa-miR-148a-3p, hsa-miR-335-5p, hsa-miR-7-5p, hsa-miR-9-3p, hsa-miR-361-5p, hsa-miR-574-5p, hsa-miR-188-5p, hsa-miR-320d, hsa-miR-629-5p, hsa-miR-361-3p, hsa-miR-335-3p, hsa-miR-500a-3p, hsa-miR-671-5p, hsa-miR-2110, hsa-miR-1-3p, hsa-miR-133a-3p
WASHC4	17	hsa-miR-335-5p, hsa-miR-148a-3p, hsa-miR-20b-5p, hsa-miR-7-5p, hsa-miR-138-5p, hsa-miR-361-5p, hsa-miR-339-5p, hsa-miR-320d, hsa-miR-342-5p, hsa-miR-335-3p, hsa-miR-500a-3p, hsa-miR-671-5p, hsa-miR-320c, hsa-miR-1-3p, hsa-miR-320a-3p, hsa-miR-147b-3p, hsa-miR-190b-5p
WIPF2	17	hsa-miR-20b-5p, hsa-miR-361-5p, hsa-miR-148a-3p, hsa-miR-7-5p, hsa-miR-138-5p, hsa-miR-574-5p, hsa-miR-589-5p, hsa-miR-1275, hsa-miR-361-3p, hsa-miR-425-3p, hsa-miR-1307-5p, hsa-miR-320c, hsa-miR-3615, hsa-miR-1-3p, hsa-miR-133a-3p, hsa-miR-320a-3p, hsa-miR-147b-3p
XPO1	17	hsa-miR-148a-3p, hsa-miR-20b-5p, hsa-miR-335-5p, hsa-miR-7-5p, hsa-miR-574-5p, hsa-miR-769-5p, hsa-miR-29c-5p, hsa-miR-361-3p, hsa-miR-342-5p, hsa-miR-335-3p, hsa-miR-500a-3p, hsa-miR-671-5p, hsa-miR-320c, hsa-miR-378i, hsa-miR-133a-3p, hsa-miR-320a-3p, hsa-miR-147b-3p
YLPM1	17	hsa-miR-148a-3p, hsa-miR-20b-5p, hsa-miR-335-5p, hsa-miR-7-5p, hsa-miR-138-5p, hsa-miR-339-5p, hsa-miR-589-5p, hsa-miR-769-5p, hsa-miR-188-5p, hsa-miR-320d, hsa-miR-335-3p, hsa-miR-671-5p, hsa-miR-320c, hsa-miR-1-3p, hsa-miR-133a-3p, hsa-miR-320a-3p, hsa-miR-147b-3p

YWHAZ	17	hsa-miR-20b-5p, hsa-miR-574-5p, hsa-miR-335-3p, hsa-miR-671-5p, hsa-miR-4516, hsa-miR-1-3p, hsa-miR-335-5p, hsa-miR-7-5p, hsa-miR-9-3p, hsa-miR-339-5p, hsa-miR-589-5p, hsa-miR-188-5p, hsa-miR-320d, hsa-miR-342-5p, hsa-miR-500a-3p, hsa-miR-320c, hsa-miR-320a-3p
YY1	17	hsa-miR-7-5p, hsa-miR-378i, hsa-miR-1-3p, hsa-miR-148a-3p, hsa-miR-20b-5p, hsa-miR-335-5p, hsa-miR-361-5p, hsa-miR-574-5p, hsa-miR-589-5p, hsa-miR-629-5p, hsa-miR-342-5p, hsa-miR-335-3p, hsa-miR-500a-3p, hsa-miR-320c, hsa-miR-3615, hsa-miR-320a-3p, hsa-miR-147b-3p
ZSWIM6	17	hsa-miR-20b-5p, hsa-miR-335-5p, hsa-miR-7-5p, hsa-miR-9-3p, hsa-miR-138-5p, hsa-miR-361-5p, hsa-miR-339-5p, hsa-miR-574-5p, hsa-miR-769-5p, hsa-miR-361-3p, hsa-miR-342-5p, hsa-miR-335-3p, hsa-miR-2110, hsa-miR-320c, hsa-miR-133a-3p, hsa-miR-320a-3p, hsa-miR-147b-3p
ACTG1	16	hsa-miR-361-5p, hsa-miR-1307-3p, hsa-miR-148a-3p, hsa-miR-20b-5p, hsa-miR-335-5p, hsa-miR-9-3p, hsa-miR-138-5p, hsa-miR-339-5p, hsa-miR-574-5p, hsa-miR-335-3p, hsa-miR-425-3p, hsa-miR-671-5p, hsa-miR-3615, hsa-miR-378i, hsa-miR-320a-3p, hsa-miR-147b-3p
ACTN4	16	hsa-miR-339-5p, hsa-miR-769-5p, hsa-miR-1-3p, hsa-miR-20b-5p, hsa-miR-7-5p, hsa-miR-138-5p, hsa-miR-361-5p, hsa-miR-574-5p, hsa-miR-629-5p, hsa-miR-335-3p, hsa-miR-425-3p, hsa-miR-500a-3p, hsa-miR-1307-3p, hsa-miR-3615, hsa-miR-320a-3p, hsa-miR-147b-3p
ADAM10	16	hsa-miR-148a-3p, hsa-miR-20b-5p, hsa-miR-335-5p, hsa-miR-7-5p, hsa-miR-138-5p, hsa-miR-361-5p, hsa-miR-574-5p, hsa-miR-589-5p, hsa-miR-1275, hsa-miR-629-5p, hsa-miR-361-3p, hsa-miR-335-3p, hsa-miR-320c, hsa-miR-378i, hsa-miR-1-3p, hsa-miR-320a-3p
ADGRL2	16	hsa-miR-335-5p, hsa-miR-589-5p, hsa-miR-20b-5p, hsa-miR-7-5p, hsa-miR-9-3p, hsa-miR-361-5p, hsa-miR-320d, hsa-miR-629-5p, hsa-miR-335-3p, hsa-miR-425-3p, hsa-miR-500a-3p, hsa-miR-1307-3p, hsa-miR-320c, hsa-miR-378i, hsa-miR-320a-3p, hsa-miR-147b-3p
AFTPH	16	hsa-miR-133a-3p, hsa-miR-148a-3p, hsa-miR-20b-5p, hsa-miR-7-5p, hsa-miR-138-5p, hsa-miR-361-5p, hsa-miR-589-5p, hsa-miR-1275, hsa-miR-342-5p, hsa-miR-500a-3p, hsa-miR-671-5p, hsa-miR-3168, hsa-miR-2110, hsa-miR-320c, hsa-miR-378i, hsa-miR-320a-3p
AHCYL1	16	hsa-miR-148a-3p, hsa-miR-20b-5p, hsa-miR-335-5p, hsa-miR-9-3p, hsa-miR-138-5p, hsa-miR-574-5p, hsa-miR-589-5p, hsa-miR-769-5p, hsa-

		miR-188-5p, hsa-miR-629-5p, hsa-miR-335-3p, hsa-miR-500a-3p, hsa-miR-1307-3p, hsa-miR-378i, hsa-miR-133a-3p, hsa-miR-320a-3p
AKAP13	16	hsa-miR-335-5p, hsa-miR-148a-3p, hsa-miR-20b-5p, hsa-miR-7-5p, hsa-miR-138-5p, hsa-miR-361-5p, hsa-miR-589-5p, hsa-miR-769-5p, hsa-miR-188-5p, hsa-miR-335-3p, hsa-miR-500a-3p, hsa-miR-671-5p, hsa-miR-2110, hsa-miR-320c, hsa-miR-378i, hsa-miR-320a-3p
ALKBH5	16	hsa-miR-4516, hsa-miR-148a-3p, hsa-miR-20b-5p, hsa-miR-7-5p, hsa-miR-361-5p, hsa-miR-339-5p, hsa-miR-574-5p, hsa-miR-342-5p, hsa-miR-335-3p, hsa-miR-1307-3p, hsa-miR-1307-5p, hsa-miR-320c, hsa-miR-3615, hsa-miR-1-3p, hsa-miR-320a-3p, hsa-miR-147b-3p
ALMS1	16	hsa-miR-148a-3p, hsa-miR-20b-5p, hsa-miR-335-5p, hsa-miR-7-5p, hsa-miR-138-5p, hsa-miR-361-5p, hsa-miR-589-5p, hsa-miR-769-5p, hsa-miR-1275, hsa-miR-629-5p, hsa-miR-335-3p, hsa-miR-2110, hsa-miR-320c, hsa-miR-1-3p, hsa-miR-320a-3p, hsa-miR-147b-3p
AMMECR1L	16	hsa-miR-148a-3p, hsa-miR-20b-5p, hsa-miR-335-5p, hsa-miR-7-5p, hsa-miR-138-5p, hsa-miR-574-5p, hsa-miR-1275, hsa-miR-342-5p, hsa-miR-335-3p, hsa-miR-425-3p, hsa-miR-500a-3p, hsa-miR-671-5p, hsa-miR-320c, hsa-miR-378i, hsa-miR-1-3p, hsa-miR-320a-3p
ANLN	16	hsa-miR-148a-3p, hsa-miR-20b-5p, hsa-miR-335-5p, hsa-miR-7-5p, hsa-miR-9-3p, hsa-miR-574-5p, hsa-miR-589-5p, hsa-miR-769-5p, hsa-miR-188-5p, hsa-miR-629-5p, hsa-miR-335-3p, hsa-miR-500a-3p, hsa-miR-671-5p, hsa-miR-3615, hsa-miR-320a-3p, hsa-miR-147b-3p
APPBP2	16	hsa-miR-148a-3p, hsa-miR-4497, hsa-miR-20b-5p, hsa-miR-335-5p, hsa-miR-7-5p, hsa-miR-138-5p, hsa-miR-361-5p, hsa-miR-769-5p, hsa-miR-342-5p, hsa-miR-335-3p, hsa-miR-500a-3p, hsa-miR-671-5p, hsa-miR-320c, hsa-miR-1-3p, hsa-miR-133a-3p, hsa-miR-320a-3p
ARF1	16	hsa-miR-320d, hsa-miR-320c, hsa-miR-148a-3p, hsa-miR-20b-5p, hsa-miR-335-5p, hsa-miR-7-5p, hsa-miR-138-5p, hsa-miR-361-5p, hsa-miR-188-5p, hsa-miR-342-5p, hsa-miR-335-3p, hsa-miR-500a-3p, hsa-miR-1307-3p, hsa-miR-1-3p, hsa-miR-320a-3p, hsa-miR-147b-3p
ARHGAP11A	16	hsa-miR-148a-3p, hsa-miR-20b-5p, hsa-miR-335-5p, hsa-miR-7-5p, hsa-miR-361-5p, hsa-miR-589-5p, hsa-miR-320d, hsa-miR-629-5p, hsa-miR-342-5p, hsa-miR-335-3p, hsa-miR-671-5p, hsa-miR-2110, hsa-miR-320c, hsa-miR-1-3p, hsa-miR-320a-3p, hsa-miR-147b-3p

ARHGAP32	16	hsa-miR-20b-5p, hsa-miR-7-5p, hsa-miR-138-5p, hsa-miR-361-5p, hsa-miR-339-5p, hsa-miR-574-5p, hsa-miR-188-5p, hsa-miR-320d, hsa-miR-335-3p, hsa-miR-425-3p, hsa-miR-2110, hsa-miR-320c, hsa-miR-378i, hsa-miR-1-3p, hsa-miR-320a-3p, hsa-miR-147b-3p
ARID2	16	hsa-miR-1-3p, hsa-miR-20b-5p, hsa-miR-335-5p, hsa-miR-7-5p, hsa-miR-138-5p, hsa-miR-361-5p, hsa-miR-339-5p, hsa-miR-589-5p, hsa-miR-188-5p, hsa-miR-629-5p, hsa-miR-342-5p, hsa-miR-335-3p, hsa-miR-671-5p, hsa-miR-320c, hsa-miR-320a-3p, hsa-miR-147b-3p
ASAP1	16	hsa-miR-148a-3p, hsa-miR-20b-5p, hsa-miR-7-5p, hsa-miR-138-5p, hsa-miR-589-5p, hsa-miR-769-5p, hsa-miR-320d, hsa-miR-629-5p, hsa-miR-335-3p, hsa-miR-500a-3p, hsa-miR-671-5p, hsa-miR-1307-3p, hsa-miR-2110, hsa-miR-320c, hsa-miR-378i, hsa-miR-320a-3p
ASB6	16	hsa-miR-148a-3p, hsa-miR-9-3p, hsa-miR-320d, hsa-miR-342-5p, hsa-miR-320c, hsa-miR-20b-5p, hsa-miR-7-5p, hsa-miR-361-5p, hsa-miR-339-5p, hsa-miR-589-5p, hsa-miR-769-5p, hsa-miR-361-3p, hsa-miR-335-3p, hsa-miR-500a-3p, hsa-miR-1-3p, hsa-miR-320a-3p
ASCC3	16	hsa-miR-148a-3p, hsa-miR-20b-5p, hsa-miR-7-5p, hsa-miR-9-3p, hsa-miR-361-5p, hsa-miR-769-5p, hsa-miR-1275, hsa-miR-629-5p, hsa-miR-29c-5p, hsa-miR-335-3p, hsa-miR-425-3p, hsa-miR-671-5p, hsa-miR-2110, hsa-miR-320c, hsa-miR-320a-3p, hsa-miR-147b-3p
ASXL2	16	hsa-miR-1-3p, hsa-miR-148a-3p, hsa-miR-20b-5p, hsa-miR-7-5p, hsa-miR-138-5p, hsa-miR-574-5p, hsa-miR-769-5p, hsa-miR-320d, hsa-miR-629-5p, hsa-miR-29c-5p, hsa-miR-361-3p, hsa-miR-335-3p, hsa-miR-500a-3p, hsa-miR-320c, hsa-miR-874-3p, hsa-miR-320a-3p
ATL2	16	hsa-miR-148a-3p, hsa-miR-20b-5p, hsa-miR-7-5p, hsa-miR-361-5p, hsa-miR-339-5p, hsa-miR-769-5p, hsa-miR-320d, hsa-miR-629-5p, hsa-miR-342-5p, hsa-miR-335-3p, hsa-miR-500a-3p, hsa-miR-3168, hsa-miR-2110, hsa-miR-320c, hsa-miR-1-3p, hsa-miR-320a-3p
ATP5F1B	16	hsa-miR-148a-3p, hsa-miR-20b-5p, hsa-miR-335-5p, hsa-miR-7-5p, hsa-miR-361-5p, hsa-miR-589-5p, hsa-miR-188-5p, hsa-miR-320d, hsa-miR-629-5p, hsa-miR-361-3p, hsa-miR-342-5p, hsa-miR-335-3p, hsa-miR-500a-3p, hsa-miR-671-5p, hsa-miR-320c, hsa-miR-320a-3p
ATXN7L3	16	hsa-miR-361-3p, hsa-miR-148a-3p, hsa-miR-20b-5p, hsa-miR-335-5p, hsa-miR-7-5p, hsa-miR-361-5p, hsa-miR-339-5p, hsa-miR-574-5p, hsa-

		miR-589-5p, hsa-miR-769-5p, hsa-miR-500a-3p, hsa-miR-671-5p, hsa-miR-2110, hsa-miR-320c, hsa-miR-320a-3p, hsa-miR-147b-3p
AUP1	16	hsa-miR-7-5p, hsa-miR-148a-3p, hsa-miR-20b-5p, hsa-miR-574-5p, hsa-miR-589-5p, hsa-miR-769-5p, hsa-miR-335-3p, hsa-miR-425-3p, hsa-miR-500a-3p, hsa-miR-671-5p, hsa-miR-1307-3p, hsa-miR-2110, hsa-miR-320c, hsa-miR-378i, hsa-miR-887-3p, hsa-miR-320a-3p
AVL9	16	hsa-miR-148a-3p, hsa-miR-20b-5p, hsa-miR-7-5p, hsa-miR-138-5p, hsa-miR-361-5p, hsa-miR-589-5p, hsa-miR-769-5p, hsa-miR-188-5p, hsa-miR-342-5p, hsa-miR-335-3p, hsa-miR-500a-3p, hsa-miR-671-5p, hsa-miR-1-3p, hsa-miR-133a-3p, hsa-miR-887-3p, hsa-miR-320a-3p
B4GALT5	16	hsa-miR-335-5p, hsa-miR-148a-3p, hsa-miR-20b-5p, hsa-miR-7-5p, hsa-miR-138-5p, hsa-miR-769-5p, hsa-miR-320d, hsa-miR-629-5p, hsa-miR-335-3p, hsa-miR-500a-3p, hsa-miR-671-5p, hsa-miR-2110, hsa-miR-320c, hsa-miR-378i, hsa-miR-1-3p, hsa-miR-320a-3p
BAG6	16	hsa-miR-148a-3p, hsa-miR-20b-5p, hsa-miR-335-5p, hsa-miR-7-5p, hsa-miR-138-5p, hsa-miR-361-5p, hsa-miR-335-3p, hsa-miR-500a-3p, hsa-miR-671-5p, hsa-miR-1307-3p, hsa-miR-1307-5p, hsa-miR-2110, hsa-miR-1468-5p, hsa-miR-133a-3p, hsa-miR-320a-3p, hsa-miR-147b-3p
BCLAF1	16	hsa-miR-342-5p, hsa-miR-148a-3p, hsa-miR-20b-5p, hsa-miR-7-5p, hsa-miR-9-3p, hsa-miR-574-5p, hsa-miR-589-5p, hsa-miR-769-5p, hsa-miR-188-5p, hsa-miR-335-3p, hsa-miR-425-3p, hsa-miR-2110, hsa-miR-320c, hsa-miR-378i, hsa-miR-133a-3p, hsa-miR-320a-3p
BCORL1	16	hsa-miR-148a-3p, hsa-miR-20b-5p, hsa-miR-335-5p, hsa-miR-7-5p, hsa-miR-138-5p, hsa-miR-361-5p, hsa-miR-339-5p, hsa-miR-574-5p, hsa-miR-769-5p, hsa-miR-1275, hsa-miR-335-3p, hsa-miR-425-3p, hsa-miR-500a-3p, hsa-miR-671-5p, hsa-miR-378i, hsa-miR-1-3p
BICRAL	16	hsa-miR-335-5p, hsa-miR-148a-3p, hsa-miR-20b-5p, hsa-miR-7-5p, hsa-miR-138-5p, hsa-miR-339-5p, hsa-miR-589-5p, hsa-miR-188-5p, hsa-miR-320d, hsa-miR-629-5p, hsa-miR-335-3p, hsa-miR-671-5p, hsa-miR-320c, hsa-miR-1-3p, hsa-miR-133a-3p, hsa-miR-320a-3p
BLTP1	16	hsa-miR-335-5p, hsa-miR-148a-3p, hsa-miR-20b-5p, hsa-miR-7-5p, hsa-miR-138-5p, hsa-miR-361-5p, hsa-miR-769-5p, hsa-miR-29c-5p, hsa-miR-342-5p, hsa-miR-500a-3p, hsa-miR-2110, hsa-miR-320c, hsa-miR-378i, hsa-miR-1-3p, hsa-miR-133a-3p, hsa-miR-320a-3p

BMI1	16	hsa-miR-148a-3p, hsa-miR-20b-5p, hsa-miR-138-5p, hsa-miR-629-5p, hsa-miR-29c-5p, hsa-miR-335-3p, hsa-miR-425-3p, hsa-miR-500a-3p, hsa-miR-671-5p, hsa-miR-1307-5p, hsa-miR-2110, hsa-miR-320c, hsa-miR-1-3p, hsa-miR-12136, hsa-miR-320a-3p, hsa-miR-147b-3p
CAMTA1	16	hsa-miR-9-3p, hsa-miR-20b-5p, hsa-miR-148a-3p, hsa-miR-7-5p, hsa-miR-138-5p, hsa-miR-361-5p, hsa-miR-574-5p, hsa-miR-589-5p, hsa-miR-342-5p, hsa-miR-335-3p, hsa-miR-425-3p, hsa-miR-500a-3p, hsa-miR-2110, hsa-miR-320c, hsa-miR-1-3p, hsa-miR-320a-3p
CAP1	16	hsa-miR-1-3p, hsa-miR-148a-3p, hsa-miR-20b-5p, hsa-miR-335-5p, hsa-miR-138-5p, hsa-miR-361-5p, hsa-miR-339-5p, hsa-miR-29c-5p, hsa-miR-361-3p, hsa-miR-500a-3p, hsa-miR-320c, hsa-miR-378i, hsa-miR-874-3p, hsa-miR-1468-5p, hsa-miR-133a-3p, hsa-miR-320a-3p
CBL	16	hsa-miR-148a-3p, hsa-miR-20b-5p, hsa-miR-335-5p, hsa-miR-7-5p, hsa-miR-9-3p, hsa-miR-138-5p, hsa-miR-769-5p, hsa-miR-1275, hsa-miR-629-5p, hsa-miR-335-3p, hsa-miR-551b-3p, hsa-miR-671-5p, hsa-miR-2110, hsa-miR-320c, hsa-miR-1-3p, hsa-miR-320a-3p
CBLL1	16	hsa-miR-148a-3p, hsa-miR-20b-5p, hsa-miR-7-5p, hsa-miR-138-5p, hsa-miR-361-5p, hsa-miR-574-5p, hsa-miR-589-5p, hsa-miR-1275, hsa-miR-629-5p, hsa-miR-342-5p, hsa-miR-500a-3p, hsa-miR-2110, hsa-miR-320c, hsa-miR-378i, hsa-miR-1-3p, hsa-miR-320a-3p
CCAR2	16	hsa-miR-138-5p, hsa-miR-148a-3p, hsa-miR-20b-5p, hsa-miR-335-5p, hsa-miR-7-5p, hsa-miR-361-5p, hsa-miR-574-5p, hsa-miR-769-5p, hsa-miR-188-5p, hsa-miR-361-3p, hsa-miR-342-5p, hsa-miR-335-3p, hsa-miR-500a-3p, hsa-miR-1307-5p, hsa-miR-1-3p, hsa-miR-320a-3p
CEMIP2	16	hsa-miR-551b-3p, hsa-miR-148a-3p, hsa-miR-20b-5p, hsa-miR-335-5p, hsa-miR-9-3p, hsa-miR-138-5p, hsa-miR-589-5p, hsa-miR-188-5p, hsa-miR-342-5p, hsa-miR-500a-3p, hsa-miR-671-5p, hsa-miR-320c, hsa-miR-1-3p, hsa-miR-133a-3p, hsa-miR-320a-3p, hsa-miR-147b-3p
CENPF	16	hsa-miR-1-3p, hsa-miR-148a-3p, hsa-miR-20b-5p, hsa-miR-335-5p, hsa-miR-7-5p, hsa-miR-138-5p, hsa-miR-361-5p, hsa-miR-339-5p, hsa-miR-589-5p, hsa-miR-188-5p, hsa-miR-342-5p, hsa-miR-335-3p, hsa-miR-500a-3p, hsa-miR-320c, hsa-miR-3615, hsa-miR-320a-3p
CHEK1	16	hsa-miR-148a-3p, hsa-miR-335-5p, hsa-miR-7-5p, hsa-miR-138-5p, hsa-miR-769-5p, hsa-miR-629-5p, hsa-miR-29c-5p, hsa-miR-342-5p, hsa-

		miR-335-3p, hsa-miR-425-3p, hsa-miR-2110, hsa-miR-320c, hsa-miR-378i, hsa-miR-1-3p, hsa-miR-320a-3p, hsa-miR-147b-3p
CIC	16	hsa-miR-1307-3p, hsa-miR-148a-3p, hsa-miR-20b-5p, hsa-miR-335-5p, hsa-miR-7-5p, hsa-miR-138-5p, hsa-miR-339-5p, hsa-miR-589-5p, hsa-miR-769-5p, hsa-miR-629-5p, hsa-miR-335-3p, hsa-miR-500a-3p, hsa-miR-671-5p, hsa-miR-320c, hsa-miR-320a-3p, hsa-miR-147b-3p
CLSTN1	16	hsa-miR-148a-3p, hsa-miR-20b-5p, hsa-miR-7-5p, hsa-miR-138-5p, hsa-miR-574-5p, hsa-miR-589-5p, hsa-miR-769-5p, hsa-miR-320d, hsa-miR-629-5p, hsa-miR-29c-5p, hsa-miR-500a-3p, hsa-miR-1307-3p, hsa-miR-1307-5p, hsa-miR-2110, hsa-miR-1-3p, hsa-miR-320a-3p
CMIP	16	hsa-miR-148a-3p, hsa-miR-20b-5p, hsa-miR-335-5p, hsa-miR-7-5p, hsa-miR-339-5p, hsa-miR-574-5p, hsa-miR-589-5p, hsa-miR-769-5p, hsa-miR-361-3p, hsa-miR-342-5p, hsa-miR-335-3p, hsa-miR-320c, hsa-miR-3615, hsa-miR-378i, hsa-miR-320a-3p, hsa-miR-147b-3p
CNBP	16	hsa-miR-769-5p, hsa-miR-1275, hsa-miR-148a-3p, hsa-miR-361-5p, hsa-miR-339-5p, hsa-miR-574-5p, hsa-miR-320d, hsa-miR-629-5p, hsa-miR-29c-5p, hsa-miR-361-3p, hsa-miR-335-3p, hsa-miR-500a-3p, hsa-miR-320c, hsa-miR-3615, hsa-miR-874-3p, hsa-miR-320a-3p
CNOT9	16	hsa-miR-148a-3p, hsa-miR-20b-5p, hsa-miR-7-5p, hsa-miR-138-5p, hsa-miR-589-5p, hsa-miR-629-5p, hsa-miR-342-5p, hsa-miR-335-3p, hsa-miR-500a-3p, hsa-miR-671-5p, hsa-miR-320c, hsa-miR-378i, hsa-miR-1-3p, hsa-miR-133a-3p, hsa-miR-320a-3p, hsa-miR-147b-3p
CPSF7	16	hsa-miR-1275, hsa-miR-148a-3p, hsa-miR-20b-5p, hsa-miR-7-5p, hsa-miR-361-5p, hsa-miR-769-5p, hsa-miR-320d, hsa-miR-629-5p, hsa-miR-335-3p, hsa-miR-500a-3p, hsa-miR-671-5p, hsa-miR-320c, hsa-miR-378i, hsa-miR-874-3p, hsa-miR-887-3p, hsa-miR-320a-3p
CRIM1	16	hsa-miR-20b-5p, hsa-miR-7-5p, hsa-miR-3168, hsa-miR-148a-3p, hsa-miR-9-3p, hsa-miR-138-5p, hsa-miR-589-5p, hsa-miR-769-5p, hsa-miR-335-3p, hsa-miR-425-3p, hsa-miR-500a-3p, hsa-miR-320c, hsa-miR-1-3p, hsa-miR-887-3p, hsa-miR-320a-3p, hsa-miR-147b-3p
CSNK1G1	16	hsa-miR-148a-3p, hsa-miR-20b-5p, hsa-miR-335-5p, hsa-miR-7-5p, hsa-miR-339-5p, hsa-miR-589-5p, hsa-miR-188-5p, hsa-miR-1275, hsa-miR-29c-5p, hsa-miR-335-3p, hsa-miR-500a-3p, hsa-miR-671-5p, hsa-miR-320c, hsa-miR-1-3p, hsa-miR-320a-3p, hsa-miR-190b-5p

DDX17	16	hsa-miR-148a-3p, hsa-miR-20b-5p, hsa-miR-335-5p, hsa-miR-138-5p, hsa-miR-361-5p, hsa-miR-769-5p, hsa-miR-188-5p, hsa-miR-629-5p, hsa-miR-342-5p, hsa-miR-335-3p, hsa-miR-671-5p, hsa-miR-2110, hsa-miR-320c, hsa-miR-378i, hsa-miR-320a-3p, hsa-miR-147b-3p
DDX6	16	hsa-miR-148a-3p, hsa-miR-335-5p, hsa-miR-1275, hsa-miR-1-3p, hsa-miR-20b-5p, hsa-miR-7-5p, hsa-miR-138-5p, hsa-miR-361-5p, hsa-miR-769-5p, hsa-miR-188-5p, hsa-miR-320d, hsa-miR-629-5p, hsa-miR-335-3p, hsa-miR-320c, hsa-miR-320a-3p, hsa-miR-147b-3p
DHCR24	16	hsa-miR-335-5p, hsa-miR-148a-3p, hsa-miR-20b-5p, hsa-miR-7-5p, hsa-miR-138-5p, hsa-miR-589-5p, hsa-miR-769-5p, hsa-miR-335-3p, hsa-miR-425-3p, hsa-miR-500a-3p, hsa-miR-671-5p, hsa-miR-1307-5p, hsa-miR-378i, hsa-miR-1-3p, hsa-miR-320a-3p, hsa-miR-147b-3p
DIP2C	16	hsa-miR-335-5p, hsa-miR-148a-3p, hsa-miR-20b-5p, hsa-miR-7-5p, hsa-miR-138-5p, hsa-miR-361-5p, hsa-miR-339-5p, hsa-miR-589-5p, hsa-miR-188-5p, hsa-miR-500a-3p, hsa-miR-671-5p, hsa-miR-2110, hsa-miR-320c, hsa-miR-1-3p, hsa-miR-320a-3p, hsa-miR-147b-3p
DLG2	16	hsa-miR-335-5p, hsa-miR-148a-3p, hsa-miR-20b-5p, hsa-miR-7-5p, hsa-miR-9-3p, hsa-miR-574-5p, hsa-miR-769-5p, hsa-miR-188-5p, hsa-miR-29c-5p, hsa-miR-500a-3p, hsa-miR-671-5p, hsa-miR-2110, hsa-miR-320c, hsa-miR-133a-3p, hsa-miR-887-3p, hsa-miR-320a-3p
DNMT1	16	hsa-miR-148a-3p, hsa-miR-20b-5p, hsa-miR-335-5p, hsa-miR-7-5p, hsa-miR-138-5p, hsa-miR-589-5p, hsa-miR-629-5p, hsa-miR-361-3p, hsa-miR-335-3p, hsa-miR-500a-3p, hsa-miR-1307-3p, hsa-miR-320c, hsa-miR-378i, hsa-miR-887-3p, hsa-miR-320a-3p, hsa-miR-147b-3p
DR1	16	hsa-miR-148a-3p, hsa-miR-20b-5p, hsa-miR-7-5p, hsa-miR-9-3p, hsa-miR-138-5p, hsa-miR-361-5p, hsa-miR-574-5p, hsa-miR-589-5p, hsa-miR-335-3p, hsa-miR-671-5p, hsa-miR-2110, hsa-miR-320c, hsa-miR-378i, hsa-miR-1-3p, hsa-miR-887-3p, hsa-miR-320a-3p
DYNC1LI2	16	hsa-miR-20b-5p, hsa-miR-425-3p, hsa-miR-500a-3p, hsa-miR-148a-3p, hsa-miR-7-5p, hsa-miR-9-3p, hsa-miR-138-5p, hsa-miR-361-5p, hsa-miR-589-5p, hsa-miR-1275, hsa-miR-671-5p, hsa-miR-2110, hsa-miR-320c, hsa-miR-1-3p, hsa-miR-133a-3p, hsa-miR-320a-3p
EDEM3	16	hsa-miR-335-5p, hsa-miR-1307-5p, hsa-miR-148a-3p, hsa-miR-20b-5p, hsa-miR-7-5p, hsa-miR-138-5p, hsa-miR-361-5p, hsa-miR-589-5p, hsa-

		miR-500a-3p, hsa-miR-551b-3p, hsa-miR-671-5p, hsa-miR-3168, hsa-miR-320c, hsa-miR-378i, hsa-miR-1-3p, hsa-miR-320a-3p
EIF5	16	hsa-miR-148a-3p, hsa-miR-20b-5p, hsa-miR-7-5p, hsa-miR-138-5p, hsa-miR-361-5p, hsa-miR-574-5p, hsa-miR-188-5p, hsa-miR-1275, hsa-miR-629-5p, hsa-miR-335-3p, hsa-miR-500a-3p, hsa-miR-671-5p, hsa-miR-320c, hsa-miR-1468-5p, hsa-miR-1-3p, hsa-miR-320a-3p
ELOVL5	16	hsa-miR-9-3p, hsa-miR-148a-3p, hsa-miR-20b-5p, hsa-miR-7-5p, hsa-miR-138-5p, hsa-miR-361-5p, hsa-miR-574-5p, hsa-miR-589-5p, hsa-miR-320d, hsa-miR-629-5p, hsa-miR-320c, hsa-miR-1-3p, hsa-miR-133a-3p, hsa-miR-887-3p, hsa-miR-320a-3p, hsa-miR-147b-3p
EPC1	16	hsa-miR-148a-3p, hsa-miR-20b-5p, hsa-miR-361-5p, hsa-miR-188-5p, hsa-miR-1275, hsa-miR-629-5p, hsa-miR-335-3p, hsa-miR-425-3p, hsa-miR-500a-3p, hsa-miR-671-5p, hsa-miR-2110, hsa-miR-320c, hsa-miR-3615, hsa-miR-378i, hsa-miR-874-3p, hsa-miR-320a-3p
EPC2	16	hsa-miR-148a-3p, hsa-miR-20b-5p, hsa-miR-7-5p, hsa-miR-9-3p, hsa-miR-138-5p, hsa-miR-361-5p, hsa-miR-589-5p, hsa-miR-320d, hsa-miR-629-5p, hsa-miR-425-3p, hsa-miR-500a-3p, hsa-miR-2110, hsa-miR-320c, hsa-miR-1-3p, hsa-miR-320a-3p, hsa-miR-190b-5p
ERC1	16	hsa-miR-335-5p, hsa-miR-1307-3p, hsa-miR-20b-5p, hsa-miR-7-5p, hsa-miR-9-3p, hsa-miR-361-5p, hsa-miR-339-5p, hsa-miR-574-5p, hsa-miR-589-5p, hsa-miR-335-3p, hsa-miR-500a-3p, hsa-miR-671-5p, hsa-miR-3615, hsa-miR-1-3p, hsa-miR-133a-3p, hsa-miR-320a-3p
FAM135A	16	hsa-miR-335-5p, hsa-miR-148a-3p, hsa-miR-20b-5p, hsa-miR-7-5p, hsa-miR-9-3p, hsa-miR-188-5p, hsa-miR-320d, hsa-miR-1275, hsa-miR-629-5p, hsa-miR-361-3p, hsa-miR-335-3p, hsa-miR-500a-3p, hsa-miR-671-5p, hsa-miR-320c, hsa-miR-1-3p, hsa-miR-320a-3p
FBRSL1	16	hsa-miR-574-5p, hsa-miR-148a-3p, hsa-miR-20b-5p, hsa-miR-7-5p, hsa-miR-138-5p, hsa-miR-769-5p, hsa-miR-320d, hsa-miR-629-5p, hsa-miR-361-3p, hsa-miR-335-3p, hsa-miR-500a-3p, hsa-miR-671-5p, hsa-miR-1307-3p, hsa-miR-3615, hsa-miR-320a-3p, hsa-miR-147b-3p
FBXO21	16	hsa-miR-20b-5p, hsa-miR-335-5p, hsa-miR-7-5p, hsa-miR-9-3p, hsa-miR-138-5p, hsa-miR-361-5p, hsa-miR-589-5p, hsa-miR-769-5p, hsa-miR-1275, hsa-miR-29c-5p, hsa-miR-500a-3p, hsa-miR-671-5p, hsa-miR-2110, hsa-miR-1-3p, hsa-miR-133a-3p, hsa-miR-887-3p

FEM1C	16	hsa-miR-20b-5p, hsa-miR-148a-3p, hsa-miR-335-5p, hsa-miR-138-5p, hsa-miR-574-5p, hsa-miR-589-5p, hsa-miR-769-5p, hsa-miR-320d, hsa-miR-335-3p, hsa-miR-500a-3p, hsa-miR-671-5p, hsa-miR-2110, hsa-miR-320c, hsa-miR-874-3p, hsa-miR-320a-3p, hsa-miR-147b-3p
FNDC3A	16	hsa-miR-1-3p, hsa-miR-148a-3p, hsa-miR-20b-5p, hsa-miR-7-5p, hsa-miR-138-5p, hsa-miR-361-5p, hsa-miR-574-5p, hsa-miR-589-5p, hsa-miR-629-5p, hsa-miR-335-3p, hsa-miR-2110, hsa-miR-320c, hsa-miR-378i, hsa-miR-874-3p, hsa-miR-320a-3p, hsa-miR-190b-5p
FUBP1	16	hsa-miR-1-3p, hsa-miR-148a-3p, hsa-miR-20b-5p, hsa-miR-7-5p, hsa-miR-9-3p, hsa-miR-361-5p, hsa-miR-339-5p, hsa-miR-574-5p, hsa-miR-629-5p, hsa-miR-361-3p, hsa-miR-500a-3p, hsa-miR-671-5p, hsa-miR-675-3p, hsa-miR-320c, hsa-miR-378i, hsa-miR-320a-3p
FUBP3	16	hsa-miR-1-3p, hsa-miR-20b-5p, hsa-miR-7-5p, hsa-miR-138-5p, hsa-miR-574-5p, hsa-miR-589-5p, hsa-miR-769-5p, hsa-miR-188-5p, hsa-miR-629-5p, hsa-miR-335-3p, hsa-miR-500a-3p, hsa-miR-2110, hsa-miR-320c, hsa-miR-378i, hsa-miR-1468-5p, hsa-miR-320a-3p
GAPDH	16	hsa-miR-342-5p, hsa-miR-20b-5p, hsa-miR-7-5p, hsa-miR-138-5p, hsa-miR-361-5p, hsa-miR-339-5p, hsa-miR-574-5p, hsa-miR-589-5p, hsa-miR-769-5p, hsa-miR-188-5p, hsa-miR-335-3p, hsa-miR-500a-3p, hsa-miR-671-5p, hsa-miR-1307-5p, hsa-miR-320a-3p, hsa-miR-147b-3p
GIGYF2	16	hsa-miR-148a-3p, hsa-miR-20b-5p, hsa-miR-7-5p, hsa-miR-138-5p, hsa-miR-361-5p, hsa-miR-339-5p, hsa-miR-769-5p, hsa-miR-188-5p, hsa-miR-361-3p, hsa-miR-335-3p, hsa-miR-1307-5p, hsa-miR-2110, hsa-miR-320c, hsa-miR-378i, hsa-miR-320a-3p, hsa-miR-190b-5p
GPATCH8	16	hsa-miR-148a-3p, hsa-miR-20b-5p, hsa-miR-7-5p, hsa-miR-9-3p, hsa-miR-138-5p, hsa-miR-361-5p, hsa-miR-339-5p, hsa-miR-574-5p, hsa-miR-320d, hsa-miR-342-5p, hsa-miR-335-3p, hsa-miR-500a-3p, hsa-miR-2110, hsa-miR-320c, hsa-miR-320a-3p, hsa-miR-147b-3p
GPCPD1	16	hsa-miR-769-5p, hsa-miR-148a-3p, hsa-miR-20b-5p, hsa-miR-7-5p, hsa-miR-138-5p, hsa-miR-574-5p, hsa-miR-589-5p, hsa-miR-320d, hsa-miR-335-3p, hsa-miR-671-5p, hsa-miR-2110, hsa-miR-320c, hsa-miR-1-3p, hsa-miR-887-3p, hsa-miR-320a-3p, hsa-miR-147b-3p
GTF3C2	16	hsa-miR-148a-3p, hsa-miR-20b-5p, hsa-miR-335-5p, hsa-miR-7-5p, hsa-miR-361-5p, hsa-miR-769-5p, hsa-miR-29c-5p, hsa-miR-361-3p, hsa-

		miR-342-5p, hsa-miR-425-3p, hsa-miR-500a-3p, hsa-miR-671-5p, hsa-miR-2110, hsa-miR-320c, hsa-miR-1-3p, hsa-miR-320a-3p
HELZ	16	hsa-miR-20b-5p, hsa-miR-335-5p, hsa-miR-7-5p, hsa-miR-138-5p, hsa-miR-361-5p, hsa-miR-339-5p, hsa-miR-574-5p, hsa-miR-1275, hsa-miR-335-3p, hsa-miR-500a-3p, hsa-miR-2110, hsa-miR-320c, hsa-miR-1-3p, hsa-miR-133a-3p, hsa-miR-320a-3p, hsa-miR-147b-3p
HIVEP2	16	hsa-miR-148a-3p, hsa-miR-20b-5p, hsa-miR-138-5p, hsa-miR-361-5p, hsa-miR-339-5p, hsa-miR-574-5p, hsa-miR-188-5p, hsa-miR-320d, hsa-miR-629-5p, hsa-miR-361-3p, hsa-miR-335-3p, hsa-miR-2110, hsa-miR-320c, hsa-miR-378i, hsa-miR-1-3p, hsa-miR-320a-3p
HNRNPA0	16	hsa-miR-20b-5p, hsa-miR-7-5p, hsa-miR-9-3p, hsa-miR-138-5p, hsa-miR-769-5p, hsa-miR-188-5p, hsa-miR-335-3p, hsa-miR-425-3p, hsa-miR-500a-3p, hsa-miR-671-5p, hsa-miR-1307-5p, hsa-miR-2110, hsa-miR-1-3p, hsa-miR-133a-3p, hsa-miR-320a-3p, hsa-miR-147b-3p
HNRNPL	16	hsa-miR-148a-3p, hsa-miR-20b-5p, hsa-miR-335-5p, hsa-miR-7-5p, hsa-miR-574-5p, hsa-miR-769-5p, hsa-miR-320d, hsa-miR-425-3p, hsa-miR-500a-3p, hsa-miR-551b-3p, hsa-miR-671-5p, hsa-miR-2110, hsa-miR-320c, hsa-miR-3615, hsa-miR-133a-3p, hsa-miR-320a-3p
HSP90AA1	16	hsa-miR-148a-3p, hsa-miR-361-5p, hsa-miR-20b-5p, hsa-miR-7-5p, hsa-miR-589-5p, hsa-miR-769-5p, hsa-miR-361-3p, hsa-miR-335-3p, hsa-miR-2110, hsa-miR-320c, hsa-miR-3615, hsa-miR-378i, hsa-miR-133a-3p, hsa-miR-887-3p, hsa-miR-320a-3p, hsa-miR-147b-3p
HSPA9	16	hsa-miR-339-5p, hsa-miR-148a-3p, hsa-miR-20b-5p, hsa-miR-335-5p, hsa-miR-7-5p, hsa-miR-361-5p, hsa-miR-589-5p, hsa-miR-769-5p, hsa-miR-188-5p, hsa-miR-320d, hsa-miR-342-5p, hsa-miR-335-3p, hsa-miR-425-3p, hsa-miR-2110, hsa-miR-133a-3p, hsa-miR-320a-3p
HSPG2	16	hsa-miR-335-5p, hsa-miR-148a-3p, hsa-miR-20b-5p, hsa-miR-7-5p, hsa-miR-9-3p, hsa-miR-138-5p, hsa-miR-361-5p, hsa-miR-769-5p, hsa-miR-188-5p, hsa-miR-342-5p, hsa-miR-500a-3p, hsa-miR-671-5p, hsa-miR-1307-5p, hsa-miR-675-3p, hsa-miR-320a-3p, hsa-miR-147b-3p
HSPH1	16	hsa-miR-20b-5p, hsa-miR-335-5p, hsa-miR-7-5p, hsa-miR-138-5p, hsa-miR-361-5p, hsa-miR-629-5p, hsa-miR-342-5p, hsa-miR-335-3p, hsa-miR-500a-3p, hsa-miR-671-5p, hsa-miR-2110, hsa-miR-1468-5p, hsa-miR-133a-3p, hsa-miR-887-3p, hsa-miR-320a-3p, hsa-miR-147b-3p

HYOU1	16	hsa-miR-335-5p, hsa-miR-148a-3p, hsa-miR-20b-5p, hsa-miR-7-5p, hsa-miR-138-5p, hsa-miR-339-5p, hsa-miR-589-5p, hsa-miR-769-5p, hsa-miR-361-3p, hsa-miR-335-3p, hsa-miR-500a-3p, hsa-miR-671-5p, hsa-miR-378i, hsa-miR-1468-5p, hsa-miR-133a-3p, hsa-miR-320a-3p
ITPR1	16	hsa-miR-335-5p, hsa-miR-148a-3p, hsa-miR-20b-5p, hsa-miR-7-5p, hsa-miR-138-5p, hsa-miR-361-5p, hsa-miR-574-5p, hsa-miR-342-5p, hsa-miR-425-3p, hsa-miR-500a-3p, hsa-miR-671-5p, hsa-miR-2110, hsa-miR-320c, hsa-miR-1-3p, hsa-miR-133a-3p, hsa-miR-320a-3p
KAT2A	16	hsa-miR-1-3p, hsa-miR-148a-3p, hsa-miR-20b-5p, hsa-miR-335-5p, hsa-miR-7-5p, hsa-miR-138-5p, hsa-miR-361-5p, hsa-miR-769-5p, hsa-miR-1275, hsa-miR-335-3p, hsa-miR-500a-3p, hsa-miR-671-5p, hsa-miR-1307-3p, hsa-miR-2110, hsa-miR-887-3p, hsa-miR-320a-3p
KPNA1	16	hsa-miR-148a-3p, hsa-miR-20b-5p, hsa-miR-7-5p, hsa-miR-138-5p, hsa-miR-361-5p, hsa-miR-574-5p, hsa-miR-1275, hsa-miR-629-5p, hsa-miR-335-3p, hsa-miR-500a-3p, hsa-miR-320c, hsa-miR-378i, hsa-miR-874-3p, hsa-miR-1-3p, hsa-miR-320a-3p, hsa-miR-147b-3p
LGR4	16	hsa-miR-148a-3p, hsa-miR-20b-5p, hsa-miR-335-5p, hsa-miR-7-5p, hsa-miR-361-5p, hsa-miR-589-5p, hsa-miR-320d, hsa-miR-629-5p, hsa-miR-361-3p, hsa-miR-335-3p, hsa-miR-671-5p, hsa-miR-2110, hsa-miR-320c, hsa-miR-1-3p, hsa-miR-887-3p, hsa-miR-320a-3p
LMNA	16	hsa-miR-148a-3p, hsa-miR-335-5p, hsa-miR-7-5p, hsa-miR-339-5p, hsa-miR-574-5p, hsa-miR-589-5p, hsa-miR-769-5p, hsa-miR-361-3p, hsa-miR-335-3p, hsa-miR-425-3p, hsa-miR-500a-3p, hsa-miR-671-5p, hsa-miR-3615, hsa-miR-378i, hsa-miR-133a-3p, hsa-miR-320a-3p
LMNB1	16	hsa-miR-1-3p, hsa-miR-148a-3p, hsa-miR-20b-5p, hsa-miR-335-5p, hsa-miR-7-5p, hsa-miR-138-5p, hsa-miR-361-5p, hsa-miR-589-5p, hsa-miR-188-5p, hsa-miR-342-5p, hsa-miR-335-3p, hsa-miR-500a-3p, hsa-miR-378i, hsa-miR-1468-5p, hsa-miR-133a-3p, hsa-miR-320a-3p
MAP3K2	16	hsa-miR-20b-5p, hsa-miR-574-5p, hsa-miR-148a-3p, hsa-miR-335-5p, hsa-miR-7-5p, hsa-miR-9-3p, hsa-miR-138-5p, hsa-miR-589-5p, hsa-miR-769-5p, hsa-miR-320d, hsa-miR-629-5p, hsa-miR-2110, hsa-miR-320c, hsa-miR-1-3p, hsa-miR-320a-3p, hsa-miR-190b-5p
MAP3K3	16	hsa-miR-20b-5p, hsa-miR-188-5p, hsa-miR-148a-3p, hsa-miR-7-5p, hsa-miR-138-5p, hsa-miR-361-5p, hsa-miR-589-5p, hsa-miR-769-5p, hsa-

		miR-361-3p, hsa-miR-335-3p, hsa-miR-500a-3p, hsa-miR-671-5p, hsa-miR-320c, hsa-miR-1-3p, hsa-miR-133a-3p, hsa-miR-320a-3p
MAPK1IP1L	16	hsa-miR-320d, hsa-miR-320c, hsa-miR-20b-5p, hsa-miR-335-5p, hsa-miR-7-5p, hsa-miR-361-5p, hsa-miR-589-5p, hsa-miR-769-5p, hsa-miR-342-5p, hsa-miR-335-3p, hsa-miR-500a-3p, hsa-miR-671-5p, hsa-miR-378i, hsa-miR-874-3p, hsa-miR-320a-3p, hsa-miR-147b-3p
MAZ	16	hsa-miR-7-5p, hsa-miR-7704, hsa-miR-148a-3p, hsa-miR-20b-5p, hsa-miR-335-5p, hsa-miR-138-5p, hsa-miR-339-5p, hsa-miR-425-3p, hsa-miR-500a-3p, hsa-miR-671-5p, hsa-miR-1307-3p, hsa-miR-1307-5p, hsa-miR-320c, hsa-miR-378i, hsa-miR-320a-3p, hsa-miR-147b-3p
MED1	16	hsa-miR-148a-3p, hsa-miR-20b-5p, hsa-miR-335-5p, hsa-miR-7-5p, hsa-miR-138-5p, hsa-miR-361-5p, hsa-miR-589-5p, hsa-miR-629-5p, hsa-miR-29c-5p, hsa-miR-361-3p, hsa-miR-342-5p, hsa-miR-500a-3p, hsa-miR-2110, hsa-miR-320c, hsa-miR-874-3p, hsa-miR-320a-3p
MINK1	16	hsa-miR-20b-5p, hsa-miR-1275, hsa-miR-671-5p, hsa-miR-148a-3p, hsa-miR-335-5p, hsa-miR-7-5p, hsa-miR-138-5p, hsa-miR-339-5p, hsa-miR-574-5p, hsa-miR-769-5p, hsa-miR-335-3p, hsa-miR-500a-3p, hsa-miR-2110, hsa-miR-378i, hsa-miR-320a-3p, hsa-miR-147b-3p
MT-ATP6	16	hsa-miR-148a-3p, hsa-miR-7-5p, hsa-miR-138-5p, hsa-miR-361-5p, hsa-miR-339-5p, hsa-miR-574-5p, hsa-miR-320d, hsa-miR-629-5p, hsa-miR-342-5p, hsa-miR-500a-3p, hsa-miR-671-5p, hsa-miR-1307-3p, hsa-miR-1-3p, hsa-miR-887-3p, hsa-miR-320a-3p, hsa-miR-147b-3p
MTA2	16	hsa-miR-335-5p, hsa-miR-148a-3p, hsa-miR-20b-5p, hsa-miR-7-5p, hsa-miR-9-3p, hsa-miR-361-5p, hsa-miR-339-5p, hsa-miR-574-5p, hsa-miR-589-5p, hsa-miR-769-5p, hsa-miR-29c-5p, hsa-miR-500a-3p, hsa-miR-3615, hsa-miR-378i, hsa-miR-320a-3p, hsa-miR-147b-3p
MTMR4	16	hsa-miR-148a-3p, hsa-miR-20b-5p, hsa-miR-335-5p, hsa-miR-7-5p, hsa-miR-361-5p, hsa-miR-574-5p, hsa-miR-589-5p, hsa-miR-769-5p, hsa-miR-500a-3p, hsa-miR-671-5p, hsa-miR-2110, hsa-miR-320c, hsa-miR-378i, hsa-miR-1-3p, hsa-miR-133a-3p, hsa-miR-320a-3p
MXI1	16	hsa-miR-20b-5p, hsa-miR-335-5p, hsa-miR-148a-3p, hsa-miR-7-5p, hsa-miR-138-5p, hsa-miR-361-5p, hsa-miR-339-5p, hsa-miR-574-5p, hsa-miR-589-5p, hsa-miR-320d, hsa-miR-629-5p, hsa-miR-342-5p, hsa-miR-425-3p, hsa-miR-320c, hsa-miR-874-3p, hsa-miR-320a-3p

NAP1L1	16	hsa-miR-148a-3p, hsa-miR-20b-5p, hsa-miR-335-5p, hsa-miR-7-5p, hsa-miR-9-3p, hsa-miR-361-5p, hsa-miR-589-5p, hsa-miR-629-5p, hsa-miR-29c-5p, hsa-miR-335-3p, hsa-miR-425-3p, hsa-miR-500a-3p, hsa-miR-671-5p, hsa-miR-320c, hsa-miR-1-3p, hsa-miR-320a-3p
NAV2	16	hsa-miR-335-3p, hsa-miR-148a-3p, hsa-miR-20b-5p, hsa-miR-7-5p, hsa-miR-9-3p, hsa-miR-138-5p, hsa-miR-361-5p, hsa-miR-629-5p, hsa-miR-29c-5p, hsa-miR-671-5p, hsa-miR-1307-3p, hsa-miR-320c, hsa-miR-3615, hsa-miR-887-3p, hsa-miR-320a-3p, hsa-miR-147b-3p
NUP210	16	hsa-miR-335-5p, hsa-miR-1-3p, hsa-miR-148a-3p, hsa-miR-20b-5p, hsa-miR-7-5p, hsa-miR-138-5p, hsa-miR-339-5p, hsa-miR-574-5p, hsa-miR-589-5p, hsa-miR-342-5p, hsa-miR-425-3p, hsa-miR-500a-3p, hsa-miR-320c, hsa-miR-378i, hsa-miR-320a-3p, hsa-miR-147b-3p
OGT	16	hsa-miR-671-5p, hsa-miR-148a-3p, hsa-miR-20b-5p, hsa-miR-335-5p, hsa-miR-7-5p, hsa-miR-138-5p, hsa-miR-361-5p, hsa-miR-574-5p, hsa-miR-589-5p, hsa-miR-188-5p, hsa-miR-629-5p, hsa-miR-335-3p, hsa-miR-500a-3p, hsa-miR-1307-5p, hsa-miR-1-3p, hsa-miR-147b-3p
PATL1	16	hsa-miR-148a-3p, hsa-miR-20b-5p, hsa-miR-335-5p, hsa-miR-7-5p, hsa-miR-138-5p, hsa-miR-589-5p, hsa-miR-335-3p, hsa-miR-500a-3p, hsa-miR-1307-5p, hsa-miR-675-3p, hsa-miR-2110, hsa-miR-3615, hsa-miR-378i, hsa-miR-1-3p, hsa-miR-320a-3p, hsa-miR-147b-3p
PDLIM5	16	hsa-miR-133a-3p, hsa-miR-148a-3p, hsa-miR-20b-5p, hsa-miR-7-5p, hsa-miR-138-5p, hsa-miR-361-5p, hsa-miR-574-5p, hsa-miR-589-5p, hsa-miR-320d, hsa-miR-425-3p, hsa-miR-500a-3p, hsa-miR-671-5p, hsa-miR-320c, hsa-miR-1-3p, hsa-miR-320a-3p, hsa-miR-147b-3p
PHF20L1	16	hsa-miR-148a-3p, hsa-miR-20b-5p, hsa-miR-7-5p, hsa-miR-138-5p, hsa-miR-361-5p, hsa-miR-574-5p, hsa-miR-589-5p, hsa-miR-320d, hsa-miR-342-5p, hsa-miR-500a-3p, hsa-miR-671-5p, hsa-miR-2110, hsa-miR-320c, hsa-miR-1-3p, hsa-miR-320a-3p, hsa-miR-147b-3p
PHF21A	16	hsa-miR-335-5p, hsa-miR-148a-3p, hsa-miR-20b-5p, hsa-miR-7-5p, hsa-miR-138-5p, hsa-miR-574-5p, hsa-miR-361-3p, hsa-miR-335-3p, hsa-miR-671-5p, hsa-miR-2110, hsa-miR-320c, hsa-miR-378i, hsa-miR-1-3p, hsa-miR-133a-3p, hsa-miR-887-3p, hsa-miR-320a-3p
PIAS1	16	hsa-miR-20b-5p, hsa-miR-7-5p, hsa-miR-9-3p, hsa-miR-138-5p, hsa-miR-361-5p, hsa-miR-589-5p, hsa-miR-1275, hsa-miR-342-5p, hsa-miR-

		500a-3p, hsa-miR-671-5p, hsa-miR-2110, hsa-miR-320c, hsa-miR-378i, hsa-miR-874-3p, hsa-miR-1-3p, hsa-miR-320a-3p
PLS3	16	hsa-miR-1-3p, hsa-miR-148a-3p, hsa-miR-20b-5p, hsa-miR-7-5p, hsa-miR-138-5p, hsa-miR-361-5p, hsa-miR-339-5p, hsa-miR-574-5p, hsa-miR-342-5p, hsa-miR-335-3p, hsa-miR-425-3p, hsa-miR-500a-3p, hsa-miR-320c, hsa-miR-874-3p, hsa-miR-887-3p, hsa-miR-320a-3p
PLXNB2	16	hsa-miR-138-5p, hsa-miR-1-3p, hsa-miR-148a-3p, hsa-miR-20b-5p, hsa-miR-335-5p, hsa-miR-7-5p, hsa-miR-361-5p, hsa-miR-339-5p, hsa-miR-574-5p, hsa-miR-589-5p, hsa-miR-342-5p, hsa-miR-335-3p, hsa-miR-500a-3p, hsa-miR-1307-3p, hsa-miR-3615, hsa-miR-133a-3p
PNISR	16	hsa-miR-7-5p, hsa-miR-148a-3p, hsa-miR-20b-5p, hsa-miR-335-5p, hsa-miR-138-5p, hsa-miR-361-5p, hsa-miR-339-5p, hsa-miR-574-5p, hsa-miR-589-5p, hsa-miR-188-5p, hsa-miR-629-5p, hsa-miR-335-3p, hsa-miR-425-3p, hsa-miR-671-5p, hsa-miR-2110, hsa-miR-3615
PPP1R15B	16	hsa-miR-20b-5p, hsa-miR-675-3p, hsa-miR-148a-3p, hsa-miR-7-5p, hsa-miR-9-3p, hsa-miR-361-5p, hsa-miR-574-5p, hsa-miR-320d, hsa-miR-335-3p, hsa-miR-500a-3p, hsa-miR-671-5p, hsa-miR-320c, hsa-miR-3615, hsa-miR-1-3p, hsa-miR-133a-3p, hsa-miR-320a-3p
PPP2CB	16	hsa-miR-20b-5p, hsa-miR-335-5p, hsa-miR-7-5p, hsa-miR-9-3p, hsa-miR-138-5p, hsa-miR-361-5p, hsa-miR-589-5p, hsa-miR-320d, hsa-miR-629-5p, hsa-miR-500a-3p, hsa-miR-551b-3p, hsa-miR-1307-3p, hsa-miR-320c, hsa-miR-1-3p, hsa-miR-887-3p, hsa-miR-320a-3p
PPP6C	16	hsa-miR-20b-5p, hsa-miR-148a-3p, hsa-miR-335-5p, hsa-miR-7-5p, hsa-miR-138-5p, hsa-miR-361-5p, hsa-miR-339-5p, hsa-miR-574-5p, hsa-miR-629-5p, hsa-miR-335-3p, hsa-miR-2110, hsa-miR-320c, hsa-miR-133a-3p, hsa-miR-887-3p, hsa-miR-320a-3p, hsa-miR-190b-5p
PPTC7	16	hsa-miR-148a-3p, hsa-miR-20b-5p, hsa-miR-7-5p, hsa-miR-9-3p, hsa-miR-138-5p, hsa-miR-574-5p, hsa-miR-335-3p, hsa-miR-500a-3p, hsa-miR-671-5p, hsa-miR-675-3p, hsa-miR-320c, hsa-miR-3615, hsa-miR-1-3p, hsa-miR-133a-3p, hsa-miR-320a-3p, hsa-miR-190b-5p
PRKAR1A	16	hsa-miR-20b-5p, hsa-miR-148a-3p, hsa-miR-335-5p, hsa-miR-7-5p, hsa-miR-9-3p, hsa-miR-361-5p, hsa-miR-589-5p, hsa-miR-769-5p, hsa-miR-320d, hsa-miR-361-3p, hsa-miR-335-3p, hsa-miR-500a-3p, hsa-miR-2110, hsa-miR-1-3p, hsa-miR-320a-3p, hsa-miR-147b-3p

PRR12	16	hsa-miR-148a-3p, hsa-miR-20b-5p, hsa-miR-138-5p, hsa-miR-339-5p, hsa-miR-769-5p, hsa-miR-629-5p, hsa-miR-361-3p, hsa-miR-342-5p, hsa-miR-335-3p, hsa-miR-500a-3p, hsa-miR-671-5p, hsa-miR-1307-3p, hsa-miR-1307-5p, hsa-miR-1-3p, hsa-miR-320a-3p, hsa-miR-147b-3p
PRR14L	16	hsa-miR-20b-5p, hsa-miR-500a-3p, hsa-miR-671-5p, hsa-miR-1-3p, hsa-miR-335-5p, hsa-miR-7-5p, hsa-miR-361-5p, hsa-miR-574-5p, hsa-miR-1275, hsa-miR-629-5p, hsa-miR-335-3p, hsa-miR-425-3p, hsa-miR-2110, hsa-miR-378i, hsa-miR-320a-3p, hsa-miR-147b-3p
PTAR1	16	hsa-miR-7-5p, hsa-miR-1-3p, hsa-miR-148a-3p, hsa-miR-20b-5p, hsa-miR-335-5p, hsa-miR-138-5p, hsa-miR-574-5p, hsa-miR-320d, hsa-miR-1275, hsa-miR-500a-3p, hsa-miR-671-5p, hsa-miR-3168, hsa-miR-2110, hsa-miR-320c, hsa-miR-320a-3p, hsa-miR-147b-3p
PTK2	16	hsa-miR-7-5p, hsa-miR-138-5p, hsa-miR-148a-3p, hsa-miR-20b-5p, hsa-miR-361-5p, hsa-miR-574-5p, hsa-miR-629-5p, hsa-miR-29c-5p, hsa-miR-361-3p, hsa-miR-335-3p, hsa-miR-425-3p, hsa-miR-500a-3p, hsa-miR-320c, hsa-miR-1468-5p, hsa-miR-133a-3p, hsa-miR-320a-3p
PTPN1	16	hsa-miR-1-3p, hsa-miR-148a-3p, hsa-miR-20b-5p, hsa-miR-7-5p, hsa-miR-138-5p, hsa-miR-361-5p, hsa-miR-574-5p, hsa-miR-769-5p, hsa-miR-335-3p, hsa-miR-425-3p, hsa-miR-500a-3p, hsa-miR-671-5p, hsa-miR-2110, hsa-miR-320c, hsa-miR-3615, hsa-miR-320a-3p
PTPRF	16	hsa-miR-1275, hsa-miR-425-3p, hsa-miR-1-3p, hsa-miR-148a-3p, hsa-miR-20b-5p, hsa-miR-335-5p, hsa-miR-7-5p, hsa-miR-138-5p, hsa-miR-574-5p, hsa-miR-769-5p, hsa-miR-188-5p, hsa-miR-500a-3p, hsa-miR-1307-5p, hsa-miR-320c, hsa-miR-320a-3p, hsa-miR-147b-3p
PTPRG	16	hsa-miR-342-5p, hsa-miR-148a-3p, hsa-miR-20b-5p, hsa-miR-7-5p, hsa-miR-138-5p, hsa-miR-361-5p, hsa-miR-339-5p, hsa-miR-335-3p, hsa-miR-500a-3p, hsa-miR-671-5p, hsa-miR-320c, hsa-miR-378i, hsa-miR-1-3p, hsa-miR-133a-3p, hsa-miR-320a-3p, hsa-miR-147b-3p
PTPRZ1	16	hsa-miR-148a-3p, hsa-miR-20b-5p, hsa-miR-7-5p, hsa-miR-138-5p, hsa-miR-361-5p, hsa-miR-574-5p, hsa-miR-342-5p, hsa-miR-671-5p, hsa-miR-2110, hsa-miR-320c, hsa-miR-378i, hsa-miR-1-3p, hsa-miR-133a-3p, hsa-miR-887-3p, hsa-miR-320a-3p, hsa-miR-190b-5p
RAC1	16	hsa-miR-361-5p, hsa-miR-425-3p, hsa-miR-2110, hsa-miR-148a-3p, hsa-miR-20b-5p, hsa-miR-7-5p, hsa-miR-339-5p, hsa-miR-361-3p, hsa-

		miR-335-3p, hsa-miR-500a-3p, hsa-miR-671-5p, hsa-miR-320c, hsa-miR-3615, hsa-miR-1-3p, hsa-miR-320a-3p, hsa-miR-147b-3p
RALGAPB	16	hsa-miR-574-5p, hsa-miR-148a-3p, hsa-miR-20b-5p, hsa-miR-335-5p, hsa-miR-7-5p, hsa-miR-138-5p, hsa-miR-361-5p, hsa-miR-188-5p, hsa-miR-342-5p, hsa-miR-335-3p, hsa-miR-425-3p, hsa-miR-671-5p, hsa-miR-2110, hsa-miR-320c, hsa-miR-1468-5p, hsa-miR-320a-3p
RGP1	16	hsa-miR-1275, hsa-miR-2110, hsa-miR-1-3p, hsa-miR-148a-3p, hsa-miR-20b-5p, hsa-miR-7-5p, hsa-miR-138-5p, hsa-miR-361-5p, hsa-miR-339-5p, hsa-miR-574-5p, hsa-miR-361-3p, hsa-miR-342-5p, hsa-miR-335-3p, hsa-miR-320c, hsa-miR-320a-3p, hsa-miR-147b-3p
RHOA	16	hsa-miR-133a-3p, hsa-miR-320c, hsa-miR-148a-3p, hsa-miR-20b-5p, hsa-miR-335-5p, hsa-miR-9-3p, hsa-miR-138-5p, hsa-miR-361-5p, hsa-miR-589-5p, hsa-miR-769-5p, hsa-miR-320d, hsa-miR-335-3p, hsa-miR-500a-3p, hsa-miR-2110, hsa-miR-3615, hsa-miR-320a-3p
RIF1	16	hsa-miR-342-5p, hsa-miR-148a-3p, hsa-miR-20b-5p, hsa-miR-335-5p, hsa-miR-7-5p, hsa-miR-138-5p, hsa-miR-361-5p, hsa-miR-574-5p, hsa-miR-589-5p, hsa-miR-629-5p, hsa-miR-29c-5p, hsa-miR-335-3p, hsa-miR-671-5p, hsa-miR-2110, hsa-miR-320c, hsa-miR-320a-3p
RLF	16	hsa-miR-20b-5p, hsa-miR-335-5p, hsa-miR-7-5p, hsa-miR-138-5p, hsa-miR-361-5p, hsa-miR-339-5p, hsa-miR-589-5p, hsa-miR-769-5p, hsa-miR-1275, hsa-miR-335-3p, hsa-miR-500a-3p, hsa-miR-671-5p, hsa-miR-675-3p, hsa-miR-2110, hsa-miR-1-3p, hsa-miR-133a-3p
RNF19A	16	hsa-miR-148a-3p, hsa-miR-20b-5p, hsa-miR-7-5p, hsa-miR-138-5p, hsa-miR-361-5p, hsa-miR-574-5p, hsa-miR-769-5p, hsa-miR-320d, hsa-miR-342-5p, hsa-miR-335-3p, hsa-miR-425-3p, hsa-miR-500a-3p, hsa-miR-2110, hsa-miR-320c, hsa-miR-1-3p, hsa-miR-320a-3p
RNF44	16	hsa-miR-335-5p, hsa-miR-148a-3p, hsa-miR-20b-5p, hsa-miR-7-5p, hsa-miR-9-3p, hsa-miR-574-5p, hsa-miR-589-5p, hsa-miR-769-5p, hsa-miR-425-3p, hsa-miR-500a-3p, hsa-miR-671-5p, hsa-miR-2110, hsa-miR-874-3p, hsa-miR-1-3p, hsa-miR-133a-3p, hsa-miR-320a-3p
RPA1	16	hsa-miR-138-5p, hsa-miR-339-5p, hsa-miR-20b-5p, hsa-miR-7-5p, hsa-miR-9-3p, hsa-miR-361-5p, hsa-miR-769-5p, hsa-miR-1275, hsa-miR-629-5p, hsa-miR-361-3p, hsa-miR-335-3p, hsa-miR-2110, hsa-miR-3615, hsa-miR-378i, hsa-miR-1-3p, hsa-miR-320a-3p

RRM1	16	hsa-miR-1-3p, hsa-miR-148a-3p, hsa-miR-20b-5p, hsa-miR-7-5p, hsa-miR-9-3p, hsa-miR-138-5p, hsa-miR-589-5p, hsa-miR-320d, hsa-miR-629-5p, hsa-miR-342-5p, hsa-miR-335-3p, hsa-miR-425-3p, hsa-miR-671-5p, hsa-miR-320c, hsa-miR-3615, hsa-miR-320a-3p
SATB2	16	hsa-miR-671-5p, hsa-miR-1468-5p, hsa-miR-148a-3p, hsa-miR-7-5p, hsa-miR-9-3p, hsa-miR-138-5p, hsa-miR-361-5p, hsa-miR-574-5p, hsa-miR-188-5p, hsa-miR-629-5p, hsa-miR-1307-3p, hsa-miR-2110, hsa-miR-320c, hsa-miR-378i, hsa-miR-1-3p, hsa-miR-320a-3p
SCRN1	16	hsa-miR-320c, hsa-miR-148a-3p, hsa-miR-20b-5p, hsa-miR-7-5p, hsa-miR-138-5p, hsa-miR-320d, hsa-miR-29c-5p, hsa-miR-361-3p, hsa-miR-500a-3p, hsa-miR-671-5p, hsa-miR-1307-3p, hsa-miR-1307-5p, hsa-miR-2110, hsa-miR-1-3p, hsa-miR-320a-3p, hsa-miR-147b-3p
SEC24D	16	hsa-miR-335-5p, hsa-miR-20b-5p, hsa-miR-7-5p, hsa-miR-138-5p, hsa-miR-361-5p, hsa-miR-574-5p, hsa-miR-589-5p, hsa-miR-1275, hsa-miR-342-5p, hsa-miR-335-3p, hsa-miR-500a-3p, hsa-miR-671-5p, hsa-miR-320c, hsa-miR-887-3p, hsa-miR-320a-3p, hsa-miR-147b-3p
SECISBP2L	16	hsa-miR-148a-3p, hsa-miR-7-5p, hsa-miR-1275, hsa-miR-20b-5p, hsa-miR-138-5p, hsa-miR-188-5p, hsa-miR-320d, hsa-miR-629-5p, hsa-miR-342-5p, hsa-miR-335-3p, hsa-miR-2110, hsa-miR-320c, hsa-miR-1-3p, hsa-miR-133a-3p, hsa-miR-887-3p, hsa-miR-320a-3p
SERBP1	16	hsa-miR-589-5p, hsa-miR-1275, hsa-miR-148a-3p, hsa-miR-20b-5p, hsa-miR-7-5p, hsa-miR-361-5p, hsa-miR-629-5p, hsa-miR-361-3p, hsa-miR-342-5p, hsa-miR-335-3p, hsa-miR-500a-3p, hsa-miR-1307-5p, hsa-miR-320c, hsa-miR-1-3p, hsa-miR-133a-3p, hsa-miR-320a-3p
SERINC3	16	hsa-miR-148a-3p, hsa-miR-7-5p, hsa-miR-9-3p, hsa-miR-138-5p, hsa-miR-574-5p, hsa-miR-769-5p, hsa-miR-320d, hsa-miR-1275, hsa-miR-629-5p, hsa-miR-335-3p, hsa-miR-671-5p, hsa-miR-320c, hsa-miR-1-3p, hsa-miR-887-3p, hsa-miR-320a-3p, hsa-miR-147b-3p
SERP1	16	hsa-miR-1-3p, hsa-miR-7-5p, hsa-miR-20b-5p, hsa-miR-9-3p, hsa-miR-138-5p, hsa-miR-361-5p, hsa-miR-339-5p, hsa-miR-589-5p, hsa-miR-769-5p, hsa-miR-629-5p, hsa-miR-335-3p, hsa-miR-425-3p, hsa-miR-500a-3p, hsa-miR-671-5p, hsa-miR-320c, hsa-miR-320a-3p
SETD7	16	hsa-miR-7-5p, hsa-miR-148a-3p, hsa-miR-20b-5p, hsa-miR-9-3p, hsa-miR-589-5p, hsa-miR-769-5p, hsa-miR-320d, hsa-miR-342-5p, hsa-miR-

		335-3p, hsa-miR-425-3p, hsa-miR-671-5p, hsa-miR-2110, hsa-miR-320c, hsa-miR-1-3p, hsa-miR-320a-3p, hsa-miR-147b-3p
SH3PXD2A	16	hsa-miR-148a-3p, hsa-miR-335-5p, hsa-miR-1275, hsa-miR-2110, hsa-miR-20b-5p, hsa-miR-7-5p, hsa-miR-138-5p, hsa-miR-339-5p, hsa-miR-769-5p, hsa-miR-629-5p, hsa-miR-29c-5p, hsa-miR-361-3p, hsa-miR-342-5p, hsa-miR-500a-3p, hsa-miR-320c, hsa-miR-320a-3p
SLAIN2	16	hsa-miR-20b-5p, hsa-miR-148a-3p, hsa-miR-335-5p, hsa-miR-7-5p, hsa-miR-138-5p, hsa-miR-361-5p, hsa-miR-574-5p, hsa-miR-589-5p, hsa-miR-320d, hsa-miR-629-5p, hsa-miR-342-5p, hsa-miR-335-3p, hsa-miR-671-5p, hsa-miR-320c, hsa-miR-320a-3p, hsa-miR-190b-5p
SLC16A1	16	hsa-miR-188-5p, hsa-miR-20b-5p, hsa-miR-335-5p, hsa-miR-7-5p, hsa-miR-138-5p, hsa-miR-589-5p, hsa-miR-769-5p, hsa-miR-320d, hsa-miR-1275, hsa-miR-335-3p, hsa-miR-671-5p, hsa-miR-2110, hsa-miR-320c, hsa-miR-1-3p, hsa-miR-133a-3p, hsa-miR-320a-3p
SLC23A2	16	hsa-miR-148a-3p, hsa-miR-20b-5p, hsa-miR-335-5p, hsa-miR-7-5p, hsa-miR-574-5p, hsa-miR-188-5p, hsa-miR-629-5p, hsa-miR-29c-5p, hsa-miR-342-5p, hsa-miR-335-3p, hsa-miR-425-3p, hsa-miR-671-5p, hsa-miR-320c, hsa-miR-1-3p, hsa-miR-133a-3p, hsa-miR-320a-3p
SLC2A1	16	hsa-miR-4516, hsa-miR-148a-3p, hsa-miR-20b-5p, hsa-miR-7-5p, hsa-miR-9-3p, hsa-miR-138-5p, hsa-miR-574-5p, hsa-miR-320d, hsa-miR-361-3p, hsa-miR-335-3p, hsa-miR-671-5p, hsa-miR-2110, hsa-miR-3615, hsa-miR-378i, hsa-miR-320a-3p, hsa-miR-147b-3p
SLC7A2	16	hsa-miR-574-5p, hsa-miR-188-5p, hsa-miR-148a-3p, hsa-miR-20b-5p, hsa-miR-7-5p, hsa-miR-9-3p, hsa-miR-138-5p, hsa-miR-361-5p, hsa-miR-589-5p, hsa-miR-342-5p, hsa-miR-335-3p, hsa-miR-320c, hsa-miR-3615, hsa-miR-1-3p, hsa-miR-320a-3p, hsa-miR-147b-3p
SMARCA2	16	hsa-miR-1-3p, hsa-miR-148a-3p, hsa-miR-20b-5p, hsa-miR-335-5p, hsa-miR-7-5p, hsa-miR-138-5p, hsa-miR-361-5p, hsa-miR-769-5p, hsa-miR-335-3p, hsa-miR-500a-3p, hsa-miR-671-5p, hsa-miR-2110, hsa-miR-320c, hsa-miR-133a-3p, hsa-miR-887-3p, hsa-miR-320a-3p
SMG7	16	hsa-miR-335-5p, hsa-miR-148a-3p, hsa-miR-20b-5p, hsa-miR-7-5p, hsa-miR-138-5p, hsa-miR-1275, hsa-miR-629-5p, hsa-miR-361-3p, hsa-miR-335-3p, hsa-miR-500a-3p, hsa-miR-671-5p, hsa-miR-2110, hsa-miR-320c, hsa-miR-378i, hsa-miR-1-3p, hsa-miR-320a-3p

SOCS4	16	hsa-miR-148a-3p, hsa-miR-20b-5p, hsa-miR-7-5p, hsa-miR-9-3p, hsa-miR-188-5p, hsa-miR-320d, hsa-miR-1275, hsa-miR-629-5p, hsa-miR-342-5p, hsa-miR-335-3p, hsa-miR-500a-3p, hsa-miR-671-5p, hsa-miR-320c, hsa-miR-1-3p, hsa-miR-320a-3p, hsa-miR-190b-5p
SPAG9	16	hsa-miR-335-5p, hsa-miR-148a-3p, hsa-miR-20b-5p, hsa-miR-7-5p, hsa-miR-138-5p, hsa-miR-361-5p, hsa-miR-339-5p, hsa-miR-574-5p, hsa-miR-589-5p, hsa-miR-342-5p, hsa-miR-335-3p, hsa-miR-500a-3p, hsa-miR-2110, hsa-miR-320c, hsa-miR-1-3p, hsa-miR-320a-3p
SPRED1	16	hsa-miR-20b-5p, hsa-miR-335-5p, hsa-miR-4791, hsa-miR-1-3p, hsa-miR-148a-3p, hsa-miR-7-5p, hsa-miR-9-3p, hsa-miR-138-5p, hsa-miR-361-5p, hsa-miR-339-5p, hsa-miR-589-5p, hsa-miR-320d, hsa-miR-671-5p, hsa-miR-320c, hsa-miR-378i, hsa-miR-320a-3p
SRRT	16	hsa-miR-20b-5p, hsa-miR-7-5p, hsa-miR-361-5p, hsa-miR-339-5p, hsa-miR-589-5p, hsa-miR-769-5p, hsa-miR-629-5p, hsa-miR-335-3p, hsa-miR-425-3p, hsa-miR-500a-3p, hsa-miR-1307-3p, hsa-miR-320c, hsa-miR-378i, hsa-miR-1-3p, hsa-miR-320a-3p, hsa-miR-147b-3p
SRSF11	16	hsa-miR-148a-3p, hsa-miR-20b-5p, hsa-miR-335-5p, hsa-miR-7-5p, hsa-miR-138-5p, hsa-miR-361-5p, hsa-miR-769-5p, hsa-miR-188-5p, hsa-miR-320d, hsa-miR-629-5p, hsa-miR-29c-5p, hsa-miR-335-3p, hsa-miR-671-5p, hsa-miR-1-3p, hsa-miR-320a-3p, hsa-miR-147b-3p
SRSF2	16	hsa-miR-20b-5p, hsa-miR-675-3p, hsa-miR-1468-5p, hsa-miR-148a-3p, hsa-miR-335-5p, hsa-miR-7-5p, hsa-miR-138-5p, hsa-miR-361-5p, hsa-miR-769-5p, hsa-miR-335-3p, hsa-miR-500a-3p, hsa-miR-1307-5p, hsa-miR-320c, hsa-miR-1-3p, hsa-miR-320a-3p, hsa-miR-147b-3p
SSRP1	16	hsa-miR-148a-3p, hsa-miR-20b-5p, hsa-miR-335-5p, hsa-miR-7-5p, hsa-miR-361-5p, hsa-miR-339-5p, hsa-miR-629-5p, hsa-miR-335-3p, hsa-miR-425-3p, hsa-miR-500a-3p, hsa-miR-1307-3p, hsa-miR-3168, hsa-miR-3615, hsa-miR-874-3p, hsa-miR-133a-3p, hsa-miR-147b-3p
STAT3	16	hsa-miR-148a-3p, hsa-miR-20b-5p, hsa-miR-551b-3p, hsa-miR-4516, hsa-miR-874-3p, hsa-miR-9-3p, hsa-miR-138-5p, hsa-miR-361-5p, hsa-miR-589-5p, hsa-miR-769-5p, hsa-miR-425-3p, hsa-miR-671-5p, hsa-miR-2110, hsa-miR-320c, hsa-miR-320a-3p, hsa-miR-147b-3p
STK4	16	hsa-miR-1275, hsa-miR-148a-3p, hsa-miR-20b-5p, hsa-miR-335-5p, hsa-miR-7-5p, hsa-miR-361-5p, hsa-miR-339-5p, hsa-miR-574-5p, hsa-

		miR-29c-5p, hsa-miR-335-3p, hsa-miR-500a-3p, hsa-miR-320c, hsa-miR-1-3p, hsa-miR-133a-3p, hsa-miR-887-3p, hsa-miR-320a-3p
TCF4	16	hsa-miR-20b-5p, hsa-miR-148a-3p, hsa-miR-7-5p, hsa-miR-9-3p, hsa-miR-138-5p, hsa-miR-574-5p, hsa-miR-320d, hsa-miR-629-5p, hsa-miR-342-5p, hsa-miR-335-3p, hsa-miR-671-5p, hsa-miR-2110, hsa-miR-320c, hsa-miR-1-3p, hsa-miR-133a-3p, hsa-miR-320a-3p
TENT4A	16	hsa-miR-629-5p, hsa-miR-4791, hsa-miR-20b-5p, hsa-miR-335-5p, hsa-miR-7-5p, hsa-miR-138-5p, hsa-miR-361-5p, hsa-miR-29c-5p, hsa-miR-342-5p, hsa-miR-335-3p, hsa-miR-500a-3p, hsa-miR-671-5p, hsa-miR-320c, hsa-miR-1-3p, hsa-miR-320a-3p, hsa-miR-147b-3p
TFRC	16	hsa-miR-7-5p, hsa-miR-769-5p, hsa-miR-148a-3p, hsa-miR-20b-5p, hsa-miR-335-5p, hsa-miR-361-5p, hsa-miR-574-5p, hsa-miR-589-5p, hsa-miR-320d, hsa-miR-29c-5p, hsa-miR-335-3p, hsa-miR-320c, hsa-miR-874-3p, hsa-miR-133a-3p, hsa-miR-320a-3p, hsa-miR-147b-3p
TGM2	16	hsa-miR-7-5p, hsa-miR-20b-5p, hsa-miR-335-5p, hsa-miR-138-5p, hsa-miR-361-5p, hsa-miR-574-5p, hsa-miR-320d, hsa-miR-335-3p, hsa-miR-500a-3p, hsa-miR-671-5p, hsa-miR-1307-5p, hsa-miR-378i, hsa-miR-874-3p, hsa-miR-1-3p, hsa-miR-320a-3p, hsa-miR-147b-3p
TMBIM6	16	hsa-miR-20b-5p, hsa-miR-335-3p, hsa-miR-148a-3p, hsa-miR-335-5p, hsa-miR-7-5p, hsa-miR-138-5p, hsa-miR-361-5p, hsa-miR-320d, hsa-miR-1275, hsa-miR-629-5p, hsa-miR-361-3p, hsa-miR-342-5p, hsa-miR-500a-3p, hsa-miR-671-5p, hsa-miR-2110, hsa-miR-320c
TMED2	16	hsa-miR-7-5p, hsa-miR-148a-3p, hsa-miR-20b-5p, hsa-miR-335-5p, hsa-miR-361-5p, hsa-miR-589-5p, hsa-miR-1275, hsa-miR-29c-5p, hsa-miR-335-3p, hsa-miR-425-3p, hsa-miR-500a-3p, hsa-miR-551b-3p, hsa-miR-671-5p, hsa-miR-2110, hsa-miR-1468-5p, hsa-miR-320a-3p
TMEM30A	16	hsa-miR-148a-3p, hsa-miR-20b-5p, hsa-miR-335-5p, hsa-miR-7-5p, hsa-miR-9-3p, hsa-miR-138-5p, hsa-miR-339-5p, hsa-miR-574-5p, hsa-miR-769-5p, hsa-miR-320d, hsa-miR-335-3p, hsa-miR-500a-3p, hsa-miR-2110, hsa-miR-320c, hsa-miR-1-3p, hsa-miR-320a-3p
TMPO	16	hsa-miR-148a-3p, hsa-miR-20b-5p, hsa-miR-335-5p, hsa-miR-7-5p, hsa-miR-138-5p, hsa-miR-361-5p, hsa-miR-629-5p, hsa-miR-335-3p, hsa-miR-425-3p, hsa-miR-671-5p, hsa-miR-2110, hsa-miR-320c, hsa-miR-378i, hsa-miR-1468-5p, hsa-miR-1-3p, hsa-miR-320a-3p

TOP2B	16	hsa-miR-148a-3p, hsa-miR-20b-5p, hsa-miR-7-5p, hsa-miR-138-5p, hsa-miR-361-5p, hsa-miR-629-5p, hsa-miR-29c-5p, hsa-miR-361-3p, hsa-miR-425-3p, hsa-miR-500a-3p, hsa-miR-671-5p, hsa-miR-2110, hsa-miR-320c, hsa-miR-378i, hsa-miR-1-3p, hsa-miR-320a-3p
TPI1	16	hsa-miR-148a-3p, hsa-miR-20b-5p, hsa-miR-7-5p, hsa-miR-361-5p, hsa-miR-574-5p, hsa-miR-769-5p, hsa-miR-188-5p, hsa-miR-361-3p, hsa-miR-335-3p, hsa-miR-500a-3p, hsa-miR-671-5p, hsa-miR-2110, hsa-miR-378i, hsa-miR-1-3p, hsa-miR-887-3p, hsa-miR-320a-3p
TPM4	16	hsa-miR-1-3p, hsa-miR-20b-5p, hsa-miR-148a-3p, hsa-miR-7-5p, hsa-miR-138-5p, hsa-miR-769-5p, hsa-miR-320d, hsa-miR-342-5p, hsa-miR-335-3p, hsa-miR-500a-3p, hsa-miR-1307-3p, hsa-miR-1307-5p, hsa-miR-2110, hsa-miR-320c, hsa-miR-12136, hsa-miR-320a-3p
TRAF7	16	hsa-miR-148a-3p, hsa-miR-335-5p, hsa-miR-7-5p, hsa-miR-138-5p, hsa-miR-339-5p, hsa-miR-574-5p, hsa-miR-589-5p, hsa-miR-320d, hsa-miR-342-5p, hsa-miR-335-3p, hsa-miR-500a-3p, hsa-miR-671-5p, hsa-miR-320c, hsa-miR-1-3p, hsa-miR-133a-3p, hsa-miR-320a-3p
TRAM1	16	hsa-miR-138-5p, hsa-miR-2110, hsa-miR-148a-3p, hsa-miR-20b-5p, hsa-miR-7-5p, hsa-miR-361-5p, hsa-miR-589-5p, hsa-miR-320d, hsa-miR-1275, hsa-miR-335-3p, hsa-miR-425-3p, hsa-miR-500a-3p, hsa-miR-378i, hsa-miR-1-3p, hsa-miR-133a-3p, hsa-miR-320a-3p
TSC1	16	hsa-miR-335-5p, hsa-miR-148a-3p, hsa-miR-20b-5p, hsa-miR-7-5p, hsa-miR-138-5p, hsa-miR-361-5p, hsa-miR-320d, hsa-miR-1275, hsa-miR-629-5p, hsa-miR-361-3p, hsa-miR-342-5p, hsa-miR-425-3p, hsa-miR-500a-3p, hsa-miR-671-5p, hsa-miR-2110, hsa-miR-320a-3p
TTC3	16	hsa-miR-335-5p, hsa-miR-148a-3p, hsa-miR-20b-5p, hsa-miR-7-5p, hsa-miR-138-5p, hsa-miR-361-5p, hsa-miR-769-5p, hsa-miR-361-3p, hsa-miR-342-5p, hsa-miR-335-3p, hsa-miR-500a-3p, hsa-miR-671-5p, hsa-miR-1307-5p, hsa-miR-320c, hsa-miR-1-3p, hsa-miR-320a-3p
TXNIP	16	hsa-miR-148a-3p, hsa-miR-20b-5p, hsa-miR-335-5p, hsa-miR-629-5p, hsa-miR-7-5p, hsa-miR-138-5p, hsa-miR-574-5p, hsa-miR-769-5p, hsa-miR-320d, hsa-miR-335-3p, hsa-miR-425-3p, hsa-miR-671-5p, hsa-miR-2110, hsa-miR-320c, hsa-miR-1-3p, hsa-miR-320a-3p
UBAP1	16	hsa-miR-148a-3p, hsa-miR-20b-5p, hsa-miR-335-5p, hsa-miR-7-5p, hsa-miR-9-3p, hsa-miR-138-5p, hsa-miR-361-5p, hsa-miR-574-5p, hsa-miR-

		589-5p, hsa-miR-320d, hsa-miR-629-5p, hsa-miR-335-3p, hsa-miR-500a-3p, hsa-miR-320c, hsa-miR-1-3p, hsa-miR-320a-3p
UBE2O	16	hsa-miR-335-5p, hsa-miR-148a-3p, hsa-miR-20b-5p, hsa-miR-7-5p, hsa-miR-138-5p, hsa-miR-361-5p, hsa-miR-574-5p, hsa-miR-769-5p, hsa-miR-188-5p, hsa-miR-500a-3p, hsa-miR-671-5p, hsa-miR-1307-3p, hsa-miR-3615, hsa-miR-1468-5p, hsa-miR-320a-3p, hsa-miR-147b-3p
UBE2R2	16	hsa-miR-7-5p, hsa-miR-148a-3p, hsa-miR-20b-5p, hsa-miR-335-5p, hsa-miR-138-5p, hsa-miR-361-5p, hsa-miR-574-5p, hsa-miR-769-5p, hsa-miR-335-3p, hsa-miR-500a-3p, hsa-miR-671-5p, hsa-miR-1307-3p, hsa-miR-1307-5p, hsa-miR-133a-3p, hsa-miR-887-3p, hsa-miR-320a-3p
UBTF	16	hsa-miR-1275, hsa-miR-342-5p, hsa-miR-1-3p, hsa-miR-148a-3p, hsa-miR-20b-5p, hsa-miR-7-5p, hsa-miR-138-5p, hsa-miR-339-5p, hsa-miR-769-5p, hsa-miR-335-3p, hsa-miR-425-3p, hsa-miR-3168, hsa-miR-378i, hsa-miR-133a-3p, hsa-miR-320a-3p, hsa-miR-147b-3p
USP14	16	hsa-miR-148a-3p, hsa-miR-20b-5p, hsa-miR-7-5p, hsa-miR-138-5p, hsa-miR-361-5p, hsa-miR-188-5p, hsa-miR-320d, hsa-miR-629-5p, hsa-miR-500a-3p, hsa-miR-671-5p, hsa-miR-320c, hsa-miR-378i, hsa-miR-1-3p, hsa-miR-887-3p, hsa-miR-320a-3p, hsa-miR-147b-3p
USP46	16	hsa-miR-148a-3p, hsa-miR-20b-5p, hsa-miR-335-5p, hsa-miR-7-5p, hsa-miR-9-3p, hsa-miR-320d, hsa-miR-629-5p, hsa-miR-29c-5p, hsa-miR-335-3p, hsa-miR-500a-3p, hsa-miR-671-5p, hsa-miR-320c, hsa-miR-378i, hsa-miR-874-3p, hsa-miR-1-3p, hsa-miR-320a-3p
VAMP2	16	hsa-miR-148a-3p, hsa-miR-20b-5p, hsa-miR-335-5p, hsa-miR-7-5p, hsa-miR-574-5p, hsa-miR-769-5p, hsa-miR-629-5p, hsa-miR-335-3p, hsa-miR-425-3p, hsa-miR-500a-3p, hsa-miR-671-5p, hsa-miR-1307-3p, hsa-miR-320c, hsa-miR-1-3p, hsa-miR-133a-3p, hsa-miR-320a-3p
VAPA	16	hsa-miR-148a-3p, hsa-miR-20b-5p, hsa-miR-335-5p, hsa-miR-7-5p, hsa-miR-138-5p, hsa-miR-361-5p, hsa-miR-339-5p, hsa-miR-574-5p, hsa-miR-589-5p, hsa-miR-1275, hsa-miR-629-5p, hsa-miR-335-3p, hsa-miR-1-3p, hsa-miR-133a-3p, hsa-miR-320a-3p, hsa-miR-147b-3p
VCL	16	hsa-miR-20b-5p, hsa-miR-7-5p, hsa-miR-9-3p, hsa-miR-138-5p, hsa-miR-574-5p, hsa-miR-769-5p, hsa-miR-320d, hsa-miR-335-3p, hsa-miR-425-3p, hsa-miR-500a-3p, hsa-miR-1307-5p, hsa-miR-3615, hsa-miR-378i, hsa-miR-874-3p, hsa-miR-320a-3p, hsa-miR-147b-3p

VCP	16	hsa-miR-361-5p, hsa-miR-361-3p, hsa-miR-148a-3p, hsa-miR-20b-5p, hsa-miR-335-5p, hsa-miR-7-5p, hsa-miR-138-5p, hsa-miR-339-5p, hsa-miR-629-5p, hsa-miR-29c-5p, hsa-miR-335-3p, hsa-miR-425-3p, hsa-miR-500a-3p, hsa-miR-1307-3p, hsa-miR-3615, hsa-miR-320a-3p
VGLL4	16	hsa-miR-671-5p, hsa-miR-20b-5p, hsa-miR-335-5p, hsa-miR-7-5p, hsa-miR-574-5p, hsa-miR-589-5p, hsa-miR-769-5p, hsa-miR-320d, hsa-miR-361-3p, hsa-miR-335-3p, hsa-miR-320c, hsa-miR-874-3p, hsa-miR-1-3p, hsa-miR-133a-3p, hsa-miR-320a-3p, hsa-miR-147b-3p
VKORC1L1	16	hsa-miR-148a-3p, hsa-miR-20b-5p, hsa-miR-335-5p, hsa-miR-7-5p, hsa-miR-9-3p, hsa-miR-361-5p, hsa-miR-589-5p, hsa-miR-629-5p, hsa-miR-29c-5p, hsa-miR-500a-3p, hsa-miR-2110, hsa-miR-320c, hsa-miR-1468-5p, hsa-miR-1-3p, hsa-miR-320a-3p, hsa-miR-147b-3p
WASL	16	hsa-miR-148a-3p, hsa-miR-20b-5p, hsa-miR-335-5p, hsa-miR-7-5p, hsa-miR-9-3p, hsa-miR-138-5p, hsa-miR-574-5p, hsa-miR-1275, hsa-miR-335-3p, hsa-miR-500a-3p, hsa-miR-2110, hsa-miR-874-3p, hsa-miR-1468-5p, hsa-miR-1-3p, hsa-miR-147b-3p, hsa-miR-190b-5p
WDR1	16	hsa-miR-20b-5p, hsa-miR-148a-3p, hsa-miR-7-5p, hsa-miR-9-3p, hsa-miR-629-5p, hsa-miR-29c-5p, hsa-miR-361-3p, hsa-miR-335-3p, hsa-miR-425-3p, hsa-miR-500a-3p, hsa-miR-378i, hsa-miR-1-3p, hsa-miR-133a-3p, hsa-miR-320a-3p, hsa-miR-147b-3p, hsa-miR-190b-5p
WDR33	16	hsa-miR-378i, hsa-miR-1-3p, hsa-miR-20b-5p, hsa-miR-335-5p, hsa-miR-7-5p, hsa-miR-361-5p, hsa-miR-339-5p, hsa-miR-589-5p, hsa-miR-769-5p, hsa-miR-188-5p, hsa-miR-29c-5p, hsa-miR-335-3p, hsa-miR-425-3p, hsa-miR-671-5p, hsa-miR-2110, hsa-miR-320a-3p
WDR45B	16	hsa-miR-20b-5p, hsa-miR-7-5p, hsa-miR-138-5p, hsa-miR-361-5p, hsa-miR-574-5p, hsa-miR-589-5p, hsa-miR-769-5p, hsa-miR-1275, hsa-miR-361-3p, hsa-miR-425-3p, hsa-miR-500a-3p, hsa-miR-2110, hsa-miR-378i, hsa-miR-874-3p, hsa-miR-1-3p, hsa-miR-320a-3p
WDR6	16	hsa-miR-148a-3p, hsa-miR-20b-5p, hsa-miR-335-5p, hsa-miR-7-5p, hsa-miR-138-5p, hsa-miR-361-5p, hsa-miR-339-5p, hsa-miR-574-5p, hsa-miR-188-5p, hsa-miR-629-5p, hsa-miR-361-3p, hsa-miR-671-5p, hsa-miR-1307-5p, hsa-miR-1-3p, hsa-miR-133a-3p, hsa-miR-147b-3p
XPO4	16	hsa-miR-671-5p, hsa-miR-148a-3p, hsa-miR-20b-5p, hsa-miR-7-5p, hsa-miR-9-3p, hsa-miR-138-5p, hsa-miR-361-5p, hsa-miR-589-5p, hsa-miR-

		769-5p, hsa-miR-320d, hsa-miR-500a-3p, hsa-miR-2110, hsa-miR-320c, hsa-miR-1-3p, hsa-miR-133a-3p, hsa-miR-320a-3p
XPO7	16	hsa-miR-148a-3p, hsa-miR-20b-5p, hsa-miR-335-5p, hsa-miR-7-5p, hsa-miR-9-3p, hsa-miR-138-5p, hsa-miR-361-5p, hsa-miR-320d, hsa-miR-629-5p, hsa-miR-335-3p, hsa-miR-671-5p, hsa-miR-2110, hsa-miR-320c, hsa-miR-1-3p, hsa-miR-320a-3p, hsa-miR-147b-3p
XRN2	16	hsa-miR-20b-5p, hsa-miR-335-5p, hsa-miR-7-5p, hsa-miR-9-3p, hsa-miR-138-5p, hsa-miR-361-5p, hsa-miR-589-5p, hsa-miR-188-5p, hsa-miR-629-5p, hsa-miR-29c-5p, hsa-miR-335-3p, hsa-miR-671-5p, hsa-miR-320c, hsa-miR-1-3p, hsa-miR-320a-3p, hsa-miR-147b-3p
YEATS2	16	hsa-miR-148a-3p, hsa-miR-20b-5p, hsa-miR-335-5p, hsa-miR-138-5p, hsa-miR-361-5p, hsa-miR-339-5p, hsa-miR-574-5p, hsa-miR-589-5p, hsa-miR-1275, hsa-miR-335-3p, hsa-miR-500a-3p, hsa-miR-671-5p, hsa-miR-2110, hsa-miR-378i, hsa-miR-1-3p, hsa-miR-147b-3p
YTHDF2	16	hsa-miR-1-3p, hsa-miR-148a-3p, hsa-miR-20b-5p, hsa-miR-335-5p, hsa-miR-7-5p, hsa-miR-589-5p, hsa-miR-769-5p, hsa-miR-1275, hsa-miR-629-5p, hsa-miR-500a-3p, hsa-miR-671-5p, hsa-miR-2110, hsa-miR-320c, hsa-miR-378i, hsa-miR-1468-5p, hsa-miR-320a-3p
YWHAE	16	hsa-miR-769-5p, hsa-miR-148a-3p, hsa-miR-20b-5p, hsa-miR-9-3p, hsa-miR-589-5p, hsa-miR-188-5p, hsa-miR-320d, hsa-miR-1275, hsa-miR-629-5p, hsa-miR-335-3p, hsa-miR-671-5p, hsa-miR-3168, hsa-miR-2110, hsa-miR-378i, hsa-miR-1-3p, hsa-miR-320a-3p
ZBTB7A	16	hsa-miR-20b-5p, hsa-miR-361-5p, hsa-miR-629-5p, hsa-miR-342-5p, hsa-miR-335-3p, hsa-miR-3615, hsa-miR-148a-3p, hsa-miR-7-5p, hsa-miR-9-3p, hsa-miR-574-5p, hsa-miR-320d, hsa-miR-361-3p, hsa-miR-1307-5p, hsa-miR-1-3p, hsa-miR-887-3p, hsa-miR-320a-3p
ZC3H13	16	hsa-miR-20b-5p, hsa-miR-335-5p, hsa-miR-7-5p, hsa-miR-361-5p, hsa-miR-339-5p, hsa-miR-769-5p, hsa-miR-335-3p, hsa-miR-425-3p, hsa-miR-500a-3p, hsa-miR-1307-3p, hsa-miR-320c, hsa-miR-3615, hsa-miR-1-3p, hsa-miR-133a-3p, hsa-miR-887-3p, hsa-miR-320a-3p
ZC3H14	16	hsa-miR-769-5p, hsa-miR-148a-3p, hsa-miR-20b-5p, hsa-miR-335-5p, hsa-miR-7-5p, hsa-miR-361-5p, hsa-miR-574-5p, hsa-miR-589-5p, hsa-miR-342-5p, hsa-miR-335-3p, hsa-miR-425-3p, hsa-miR-500a-3p, hsa-miR-2110, hsa-miR-1-3p, hsa-miR-133a-3p, hsa-miR-320a-3p

ZEB2	16	hsa-miR-335-5p, hsa-miR-138-5p, hsa-miR-148a-3p, hsa-miR-20b-5p, hsa-miR-7-5p, hsa-miR-361-5p, hsa-miR-574-5p, hsa-miR-769-5p, hsa-miR-629-5p, hsa-miR-342-5p, hsa-miR-425-3p, hsa-miR-500a-3p, hsa-miR-671-5p, hsa-miR-1-3p, hsa-miR-320a-3p, hsa-miR-190b-5p
ZFC3H1	16	hsa-miR-148a-3p, hsa-miR-20b-5p, hsa-miR-335-5p, hsa-miR-7-5p, hsa-miR-361-5p, hsa-miR-574-5p, hsa-miR-769-5p, hsa-miR-320d, hsa-miR-1275, hsa-miR-629-5p, hsa-miR-335-3p, hsa-miR-425-3p, hsa-miR-500a-3p, hsa-miR-320c, hsa-miR-3615, hsa-miR-320a-3p
ZFP36	16	hsa-miR-1275, hsa-miR-148a-3p, hsa-miR-20b-5p, hsa-miR-335-5p, hsa-miR-7-5p, hsa-miR-138-5p, hsa-miR-361-5p, hsa-miR-574-5p, hsa-miR-769-5p, hsa-miR-629-5p, hsa-miR-342-5p, hsa-miR-425-3p, hsa-miR-671-5p, hsa-miR-2110, hsa-miR-320a-3p, hsa-miR-190b-5p
ZMYM2	16	hsa-miR-20b-5p, hsa-miR-335-5p, hsa-miR-7-5p, hsa-miR-138-5p, hsa-miR-361-5p, hsa-miR-589-5p, hsa-miR-188-5p, hsa-miR-320d, hsa-miR-361-3p, hsa-miR-342-5p, hsa-miR-335-3p, hsa-miR-320c, hsa-miR-378i, hsa-miR-874-3p, hsa-miR-1-3p, hsa-miR-320a-3p
ZNF106	16	hsa-miR-7-5p, hsa-miR-148a-3p, hsa-miR-20b-5p, hsa-miR-138-5p, hsa-miR-361-5p, hsa-miR-188-5p, hsa-miR-342-5p, hsa-miR-335-3p, hsa-miR-425-3p, hsa-miR-671-5p, hsa-miR-1307-5p, hsa-miR-320c, hsa-miR-378i, hsa-miR-1-3p, hsa-miR-887-3p, hsa-miR-320a-3p
ZNF107	16	hsa-miR-20b-5p, hsa-miR-675-3p, hsa-miR-148a-3p, hsa-miR-7-5p, hsa-miR-9-3p, hsa-miR-589-5p, hsa-miR-769-5p, hsa-miR-320d, hsa-miR-1275, hsa-miR-335-3p, hsa-miR-500a-3p, hsa-miR-2110, hsa-miR-320c, hsa-miR-3615, hsa-miR-320a-3p, hsa-miR-147b-3p
ZNF148	16	hsa-miR-335-5p, hsa-miR-9-3p, hsa-miR-320d, hsa-miR-320c, hsa-miR-148a-3p, hsa-miR-20b-5p, hsa-miR-7-5p, hsa-miR-361-5p, hsa-miR-589-5p, hsa-miR-335-3p, hsa-miR-671-5p, hsa-miR-2110, hsa-miR-1-3p, hsa-miR-133a-3p, hsa-miR-320a-3p, hsa-miR-147b-3p
ZNF292	16	hsa-miR-335-5p, hsa-miR-148a-3p, hsa-miR-20b-5p, hsa-miR-9-3p, hsa-miR-138-5p, hsa-miR-361-5p, hsa-miR-339-5p, hsa-miR-574-5p, hsa-miR-29c-5p, hsa-miR-342-5p, hsa-miR-335-3p, hsa-miR-320c, hsa-miR-378i, hsa-miR-1-3p, hsa-miR-887-3p, hsa-miR-320a-3p
ZNFX1	16	hsa-miR-20b-5p, hsa-miR-148a-3p, hsa-miR-335-5p, hsa-miR-7-5p, hsa-miR-361-5p, hsa-miR-574-5p, hsa-miR-589-5p, hsa-miR-769-5p, hsa-

		miR-320d, hsa-miR-629-5p, hsa-miR-342-5p, hsa-miR-335-3p, hsa-miR-500a-3p, hsa-miR-2110, hsa-miR-320a-3p, hsa-miR-147b-3p
ABCF2	15	hsa-miR-148a-3p, hsa-miR-20b-5p, hsa-miR-335-5p, hsa-miR-7-5p, hsa-miR-361-5p, hsa-miR-589-5p, hsa-miR-1275, hsa-miR-629-5p, hsa-miR-342-5p, hsa-miR-335-3p, hsa-miR-500a-3p, hsa-miR-1307-5p, hsa-miR-320c, hsa-miR-887-3p, hsa-miR-320a-3p
ABL2	15	hsa-miR-148a-3p, hsa-miR-20b-5p, hsa-miR-335-5p, hsa-miR-7-5p, hsa-miR-138-5p, hsa-miR-574-5p, hsa-miR-589-5p, hsa-miR-769-5p, hsa-miR-1275, hsa-miR-342-5p, hsa-miR-500a-3p, hsa-miR-671-5p, hsa-miR-320c, hsa-miR-320a-3p, hsa-miR-147b-3p
ACSL4	15	hsa-miR-20b-5p, hsa-miR-148a-3p, hsa-miR-7-5p, hsa-miR-9-3p, hsa-miR-361-5p, hsa-miR-574-5p, hsa-miR-188-5p, hsa-miR-342-5p, hsa-miR-335-3p, hsa-miR-671-5p, hsa-miR-320c, hsa-miR-1-3p, hsa-miR-320a-3p, hsa-miR-147b-3p, hsa-miR-190b-5p
ACTN1	15	hsa-miR-1-3p, hsa-miR-20b-5p, hsa-miR-335-5p, hsa-miR-7-5p, hsa-miR-138-5p, hsa-miR-574-5p, hsa-miR-188-5p, hsa-miR-320d, hsa-miR-335-3p, hsa-miR-500a-3p, hsa-miR-671-5p, hsa-miR-2110, hsa-miR-378i, hsa-miR-320a-3p, hsa-miR-147b-3p
ACVR2B	15	hsa-miR-335-5p, hsa-miR-574-5p, hsa-miR-361-3p, hsa-miR-148a-3p, hsa-miR-20b-5p, hsa-miR-7-5p, hsa-miR-9-3p, hsa-miR-138-5p, hsa-miR-361-5p, hsa-miR-320d, hsa-miR-342-5p, hsa-miR-500a-3p, hsa-miR-320c, hsa-miR-320a-3p, hsa-miR-147b-3p
AFDN	15	hsa-miR-335-5p, hsa-miR-148a-3p, hsa-miR-20b-5p, hsa-miR-7-5p, hsa-miR-9-3p, hsa-miR-138-5p, hsa-miR-361-5p, hsa-miR-29c-5p, hsa-miR-335-3p, hsa-miR-500a-3p, hsa-miR-2110, hsa-miR-378i, hsa-miR-1-3p, hsa-miR-320a-3p, hsa-miR-147b-3p
AFF1	15	hsa-miR-20b-5p, hsa-miR-361-5p, hsa-miR-148a-3p, hsa-miR-335-5p, hsa-miR-7-5p, hsa-miR-138-5p, hsa-miR-339-5p, hsa-miR-589-5p, hsa-miR-769-5p, hsa-miR-320d, hsa-miR-2110, hsa-miR-320c, hsa-miR-378i, hsa-miR-320a-3p, hsa-miR-147b-3p
AKAP9	15	hsa-miR-20b-5p, hsa-miR-7-5p, hsa-miR-138-5p, hsa-miR-361-5p, hsa-miR-339-5p, hsa-miR-769-5p, hsa-miR-188-5p, hsa-miR-320d, hsa-miR-29c-5p, hsa-miR-335-3p, hsa-miR-320c, hsa-miR-378i, hsa-miR-874-3p, hsa-miR-1-3p, hsa-miR-320a-3p

AMBRA1	15	hsa-miR-339-5p, hsa-miR-148a-3p, hsa-miR-20b-5p, hsa-miR-7-5p, hsa-miR-361-5p, hsa-miR-574-5p, hsa-miR-769-5p, hsa-miR-629-5p, hsa-miR-342-5p, hsa-miR-500a-3p, hsa-miR-671-5p, hsa-miR-320c, hsa-miR-1-3p, hsa-miR-320a-3p, hsa-miR-147b-3p
ANKHD1	15	hsa-miR-148a-3p, hsa-miR-20b-5p, hsa-miR-7-5p, hsa-miR-138-5p, hsa-miR-361-5p, hsa-miR-589-5p, hsa-miR-320d, hsa-miR-629-5p, hsa-miR-342-5p, hsa-miR-335-3p, hsa-miR-500a-3p, hsa-miR-320c, hsa-miR-378i, hsa-miR-133a-3p, hsa-miR-320a-3p
ANP32E	15	hsa-miR-339-5p, hsa-miR-3168, hsa-miR-1-3p, hsa-miR-20b-5p, hsa-miR-335-5p, hsa-miR-7-5p, hsa-miR-769-5p, hsa-miR-1275, hsa-miR-335-3p, hsa-miR-500a-3p, hsa-miR-671-5p, hsa-miR-2110, hsa-miR-320c, hsa-miR-3615, hsa-miR-320a-3p
ARCN1	15	hsa-miR-1-3p, hsa-miR-20b-5p, hsa-miR-361-5p, hsa-miR-148a-3p, hsa-miR-335-5p, hsa-miR-7-5p, hsa-miR-138-5p, hsa-miR-339-5p, hsa-miR-320d, hsa-miR-335-3p, hsa-miR-500a-3p, hsa-miR-671-5p, hsa-miR-320c, hsa-miR-3615, hsa-miR-320a-3p
ARF6	15	hsa-miR-3615, hsa-miR-20b-5p, hsa-miR-335-5p, hsa-miR-7-5p, hsa-miR-339-5p, hsa-miR-574-5p, hsa-miR-769-5p, hsa-miR-29c-5p, hsa-miR-361-3p, hsa-miR-335-3p, hsa-miR-671-5p, hsa-miR-2110, hsa-miR-320c, hsa-miR-133a-3p, hsa-miR-320a-3p
ARFGEF2	15	hsa-miR-335-3p, hsa-miR-148a-3p, hsa-miR-20b-5p, hsa-miR-7-5p, hsa-miR-138-5p, hsa-miR-361-5p, hsa-miR-574-5p, hsa-miR-589-5p, hsa-miR-500a-3p, hsa-miR-671-5p, hsa-miR-320c, hsa-miR-378i, hsa-miR-1-3p, hsa-miR-887-3p, hsa-miR-320a-3p
ATP11A	15	hsa-miR-769-5p, hsa-miR-148a-3p, hsa-miR-20b-5p, hsa-miR-7-5p, hsa-miR-138-5p, hsa-miR-361-5p, hsa-miR-574-5p, hsa-miR-589-5p, hsa-miR-320d, hsa-miR-361-3p, hsa-miR-500a-3p, hsa-miR-671-5p, hsa-miR-320c, hsa-miR-1-3p, hsa-miR-320a-3p
ATP1B1	15	hsa-miR-148a-3p, hsa-miR-20b-5p, hsa-miR-7-5p, hsa-miR-9-3p, hsa-miR-574-5p, hsa-miR-589-5p, hsa-miR-320d, hsa-miR-629-5p, hsa-miR-342-5p, hsa-miR-335-3p, hsa-miR-425-3p, hsa-miR-500a-3p, hsa-miR-1-3p, hsa-miR-133a-3p, hsa-miR-320a-3p
ATP2B4	15	hsa-miR-335-5p, hsa-miR-1-3p, hsa-miR-148a-3p, hsa-miR-20b-5p, hsa-miR-7-5p, hsa-miR-138-5p, hsa-miR-361-5p, hsa-miR-339-5p, hsa-miR-

		589-5p, hsa-miR-335-3p, hsa-miR-671-5p, hsa-miR-1307-3p, hsa-miR-378i, hsa-miR-320a-3p, hsa-miR-147b-3p
ATP9A	15	hsa-miR-335-5p, hsa-miR-3615, hsa-miR-148a-3p, hsa-miR-20b-5p, hsa-miR-7-5p, hsa-miR-361-5p, hsa-miR-589-5p, hsa-miR-320d, hsa-miR-425-3p, hsa-miR-500a-3p, hsa-miR-671-5p, hsa-miR-320c, hsa-miR-1-3p, hsa-miR-320a-3p, hsa-miR-147b-3p
ATRN	15	hsa-miR-3168, hsa-miR-148a-3p, hsa-miR-20b-5p, hsa-miR-335-5p, hsa-miR-7-5p, hsa-miR-361-5p, hsa-miR-574-5p, hsa-miR-320d, hsa-miR-1275, hsa-miR-335-3p, hsa-miR-671-5p, hsa-miR-1307-5p, hsa-miR-2110, hsa-miR-320a-3p, hsa-miR-147b-3p
ATXN2L	15	hsa-miR-148a-3p, hsa-miR-20b-5p, hsa-miR-7-5p, hsa-miR-138-5p, hsa-miR-339-5p, hsa-miR-320d, hsa-miR-361-3p, hsa-miR-335-3p, hsa-miR-671-5p, hsa-miR-3168, hsa-miR-320c, hsa-miR-378i, hsa-miR-133a-3p, hsa-miR-320a-3p, hsa-miR-147b-3p
BCL7B	15	hsa-miR-589-5p, hsa-miR-320d, hsa-miR-320c, hsa-miR-1-3p, hsa-miR-148a-3p, hsa-miR-20b-5p, hsa-miR-7-5p, hsa-miR-361-5p, hsa-miR-29c-5p, hsa-miR-342-5p, hsa-miR-425-3p, hsa-miR-500a-3p, hsa-miR-874-3p, hsa-miR-320a-3p, hsa-miR-147b-3p
BICD2	15	hsa-miR-20b-5p, hsa-miR-769-5p, hsa-miR-148a-3p, hsa-miR-335-5p, hsa-miR-7-5p, hsa-miR-138-5p, hsa-miR-361-5p, hsa-miR-188-5p, hsa-miR-671-5p, hsa-miR-320c, hsa-miR-378i, hsa-miR-1-3p, hsa-miR-887-3p, hsa-miR-320a-3p, hsa-miR-147b-3p
BRI3BP	15	hsa-miR-20b-5p, hsa-miR-574-5p, hsa-miR-1-3p, hsa-miR-148a-3p, hsa-miR-335-5p, hsa-miR-9-3p, hsa-miR-138-5p, hsa-miR-361-5p, hsa-miR-769-5p, hsa-miR-629-5p, hsa-miR-671-5p, hsa-miR-1307-5p, hsa-miR-320c, hsa-miR-133a-3p, hsa-miR-320a-3p
BRWD1	15	hsa-miR-589-5p, hsa-miR-148a-3p, hsa-miR-20b-5p, hsa-miR-335-5p, hsa-miR-7-5p, hsa-miR-138-5p, hsa-miR-574-5p, hsa-miR-320d, hsa-miR-361-3p, hsa-miR-335-3p, hsa-miR-500a-3p, hsa-miR-320c, hsa-miR-133a-3p, hsa-miR-320a-3p, hsa-miR-190b-5p
BRWD3	15	hsa-miR-574-5p, hsa-miR-148a-3p, hsa-miR-20b-5p, hsa-miR-7-5p, hsa-miR-9-3p, hsa-miR-138-5p, hsa-miR-361-5p, hsa-miR-320d, hsa-miR-425-3p, hsa-miR-500a-3p, hsa-miR-671-5p, hsa-miR-320c, hsa-miR-1468-5p, hsa-miR-320a-3p, hsa-miR-147b-3p

C5orf24	15	hsa-miR-148a-3p, hsa-miR-20b-5p, hsa-miR-335-5p, hsa-miR-7-5p, hsa-miR-9-3p, hsa-miR-361-5p, hsa-miR-589-5p, hsa-miR-769-5p, hsa-miR-1275, hsa-miR-335-3p, hsa-miR-500a-3p, hsa-miR-320c, hsa-miR-1-3p, hsa-miR-320a-3p, hsa-miR-147b-3p
C6orf62	15	hsa-miR-148a-3p, hsa-miR-20b-5p, hsa-miR-7-5p, hsa-miR-138-5p, hsa-miR-589-5p, hsa-miR-335-3p, hsa-miR-425-3p, hsa-miR-551b-3p, hsa-miR-671-5p, hsa-miR-3168, hsa-miR-2110, hsa-miR-320c, hsa-miR-1-3p, hsa-miR-320a-3p, hsa-miR-147b-3p
CALM1	15	hsa-miR-1-3p, hsa-miR-148a-3p, hsa-miR-20b-5p, hsa-miR-7-5p, hsa-miR-361-5p, hsa-miR-320d, hsa-miR-629-5p, hsa-miR-335-3p, hsa-miR-671-5p, hsa-miR-1307-3p, hsa-miR-2110, hsa-miR-320c, hsa-miR-378i, hsa-miR-320a-3p, hsa-miR-147b-3p
CALR	15	hsa-miR-1275, hsa-miR-1-3p, hsa-miR-148a-3p, hsa-miR-20b-5p, hsa-miR-339-5p, hsa-miR-589-5p, hsa-miR-629-5p, hsa-miR-335-3p, hsa-miR-425-3p, hsa-miR-1307-3p, hsa-miR-2110, hsa-miR-3615, hsa-miR-874-3p, hsa-miR-133a-3p, hsa-miR-320a-3p
CALU	15	hsa-miR-7-5p, hsa-miR-148a-3p, hsa-miR-20b-5p, hsa-miR-335-5p, hsa-miR-769-5p, hsa-miR-320d, hsa-miR-500a-3p, hsa-miR-671-5p, hsa-miR-2110, hsa-miR-320c, hsa-miR-3615, hsa-miR-1-3p, hsa-miR-133a-3p, hsa-miR-320a-3p, hsa-miR-147b-3p
CAPN2	15	hsa-miR-7-5p, hsa-miR-148a-3p, hsa-miR-20b-5p, hsa-miR-335-5p, hsa-miR-9-3p, hsa-miR-138-5p, hsa-miR-361-5p, hsa-miR-574-5p, hsa-miR-335-3p, hsa-miR-671-5p, hsa-miR-1307-5p, hsa-miR-320c, hsa-miR-1-3p, hsa-miR-320a-3p, hsa-miR-147b-3p
CCAR1	15	hsa-miR-20b-5p, hsa-miR-335-5p, hsa-miR-7-5p, hsa-miR-361-5p, hsa-miR-339-5p, hsa-miR-629-5p, hsa-miR-335-3p, hsa-miR-1307-3p, hsa-miR-1307-5p, hsa-miR-320c, hsa-miR-3615, hsa-miR-378i, hsa-miR-133a-3p, hsa-miR-320a-3p, hsa-miR-147b-3p
CCT3	15	hsa-miR-148a-3p, hsa-miR-20b-5p, hsa-miR-7-5p, hsa-miR-9-3p, hsa-miR-138-5p, hsa-miR-361-5p, hsa-miR-574-5p, hsa-miR-589-5p, hsa-miR-342-5p, hsa-miR-335-3p, hsa-miR-671-5p, hsa-miR-1307-5p, hsa-miR-3615, hsa-miR-378i, hsa-miR-1-3p
CDC42SE1	15	hsa-miR-4516, hsa-miR-1-3p, hsa-miR-20b-5p, hsa-miR-574-5p, hsa-miR-589-5p, hsa-miR-769-5p, hsa-miR-320d, hsa-miR-361-3p, hsa-miR-

		500a-3p, hsa-miR-671-5p, hsa-miR-2110, hsa-miR-320c, hsa-miR-378i, hsa-miR-887-3p, hsa-miR-320a-3p
CDC73	15	hsa-miR-148a-3p, hsa-miR-20b-5p, hsa-miR-7-5p, hsa-miR-589-5p, hsa-miR-320d, hsa-miR-1275, hsa-miR-629-5p, hsa-miR-361-3p, hsa-miR-342-5p, hsa-miR-335-3p, hsa-miR-671-5p, hsa-miR-378i, hsa-miR-1468-5p, hsa-miR-1-3p, hsa-miR-147b-3p
CDS2	15	hsa-miR-7704, hsa-miR-148a-3p, hsa-miR-20b-5p, hsa-miR-335-5p, hsa-miR-7-5p, hsa-miR-138-5p, hsa-miR-361-5p, hsa-miR-589-5p, hsa-miR-769-5p, hsa-miR-188-5p, hsa-miR-342-5p, hsa-miR-320c, hsa-miR-378i, hsa-miR-1-3p, hsa-miR-320a-3p
CHD1	15	hsa-miR-148a-3p, hsa-miR-7-5p, hsa-miR-361-5p, hsa-miR-339-5p, hsa-miR-769-5p, hsa-miR-629-5p, hsa-miR-29c-5p, hsa-miR-335-3p, hsa-miR-671-5p, hsa-miR-1307-3p, hsa-miR-2110, hsa-miR-320c, hsa-miR-1-3p, hsa-miR-320a-3p, hsa-miR-147b-3p
CHD7	15	hsa-miR-148a-3p, hsa-miR-20b-5p, hsa-miR-7-5p, hsa-miR-138-5p, hsa-miR-361-5p, hsa-miR-339-5p, hsa-miR-574-5p, hsa-miR-589-5p, hsa-miR-629-5p, hsa-miR-342-5p, hsa-miR-335-3p, hsa-miR-425-3p, hsa-miR-320c, hsa-miR-320a-3p, hsa-miR-190b-5p
CHERP	15	hsa-miR-342-5p, hsa-miR-335-3p, hsa-miR-671-5p, hsa-miR-148a-3p, hsa-miR-20b-5p, hsa-miR-335-5p, hsa-miR-7-5p, hsa-miR-138-5p, hsa-miR-188-5p, hsa-miR-425-3p, hsa-miR-500a-3p, hsa-miR-1307-5p, hsa-miR-1-3p, hsa-miR-320a-3p, hsa-miR-147b-3p
CHTF8	15	hsa-miR-148a-3p, hsa-miR-20b-5p, hsa-miR-335-5p, hsa-miR-7-5p, hsa-miR-138-5p, hsa-miR-589-5p, hsa-miR-335-3p, hsa-miR-500a-3p, hsa-miR-671-5p, hsa-miR-2110, hsa-miR-320c, hsa-miR-378i, hsa-miR-1-3p, hsa-miR-133a-3p, hsa-miR-320a-3p
CIT	15	hsa-miR-20b-5p, hsa-miR-148a-3p, hsa-miR-335-5p, hsa-miR-7-5p, hsa-miR-138-5p, hsa-miR-361-5p, hsa-miR-339-5p, hsa-miR-769-5p, hsa-miR-342-5p, hsa-miR-335-3p, hsa-miR-425-3p, hsa-miR-500a-3p, hsa-miR-671-5p, hsa-miR-1-3p, hsa-miR-320a-3p
CIZ1	15	hsa-miR-148a-3p, hsa-miR-20b-5p, hsa-miR-335-5p, hsa-miR-7-5p, hsa-miR-138-5p, hsa-miR-339-5p, hsa-miR-361-3p, hsa-miR-335-3p, hsa-miR-500a-3p, hsa-miR-671-5p, hsa-miR-1307-5p, hsa-miR-2110, hsa-miR-1-3p, hsa-miR-320a-3p, hsa-miR-147b-3p

CLOCK	15	hsa-miR-20b-5p, hsa-miR-148a-3p, hsa-miR-335-5p, hsa-miR-7-5p, hsa-miR-9-3p, hsa-miR-138-5p, hsa-miR-589-5p, hsa-miR-320d, hsa-miR-335-3p, hsa-miR-671-5p, hsa-miR-2110, hsa-miR-320c, hsa-miR-378i, hsa-miR-1-3p, hsa-miR-320a-3p
COL12A1	15	hsa-miR-1-3p, hsa-miR-20b-5p, hsa-miR-7-5p, hsa-miR-361-5p, hsa-miR-574-5p, hsa-miR-589-5p, hsa-miR-769-5p, hsa-miR-188-5p, hsa-miR-629-5p, hsa-miR-29c-5p, hsa-miR-500a-3p, hsa-miR-675-3p, hsa-miR-2110, hsa-miR-320c, hsa-miR-320a-3p
COPG1	15	hsa-miR-335-5p, hsa-miR-1-3p, hsa-miR-148a-3p, hsa-miR-7-5p, hsa-miR-138-5p, hsa-miR-361-5p, hsa-miR-574-5p, hsa-miR-1275, hsa-miR-361-3p, hsa-miR-342-5p, hsa-miR-335-3p, hsa-miR-2110, hsa-miR-320c, hsa-miR-887-3p, hsa-miR-320a-3p
CPEB4	15	hsa-miR-335-5p, hsa-miR-29c-5p, hsa-miR-874-3p, hsa-miR-148a-3p, hsa-miR-20b-5p, hsa-miR-7-5p, hsa-miR-138-5p, hsa-miR-361-5p, hsa-miR-574-5p, hsa-miR-320d, hsa-miR-1275, hsa-miR-2110, hsa-miR-320c, hsa-miR-320a-3p, hsa-miR-147b-3p
CREB3L2	15	hsa-miR-574-5p, hsa-miR-1-3p, hsa-miR-148a-3p, hsa-miR-20b-5p, hsa-miR-335-5p, hsa-miR-7-5p, hsa-miR-138-5p, hsa-miR-320d, hsa-miR-342-5p, hsa-miR-671-5p, hsa-miR-2110, hsa-miR-320c, hsa-miR-1468-5p, hsa-miR-320a-3p, hsa-miR-147b-3p
CS	15	hsa-miR-148a-3p, hsa-miR-20b-5p, hsa-miR-7-5p, hsa-miR-138-5p, hsa-miR-361-5p, hsa-miR-574-5p, hsa-miR-769-5p, hsa-miR-629-5p, hsa-miR-29c-5p, hsa-miR-361-3p, hsa-miR-335-3p, hsa-miR-2110, hsa-miR-1468-5p, hsa-miR-320a-3p, hsa-miR-147b-3p
CSTF2T	15	hsa-miR-148a-3p, hsa-miR-20b-5p, hsa-miR-335-5p, hsa-miR-9-3p, hsa-miR-138-5p, hsa-miR-361-5p, hsa-miR-769-5p, hsa-miR-188-5p, hsa-miR-425-3p, hsa-miR-500a-3p, hsa-miR-671-5p, hsa-miR-320c, hsa-miR-378i, hsa-miR-1-3p, hsa-miR-320a-3p
CUL3	15	hsa-miR-339-5p, hsa-miR-148a-3p, hsa-miR-20b-5p, hsa-miR-335-5p, hsa-miR-7-5p, hsa-miR-138-5p, hsa-miR-361-5p, hsa-miR-574-5p, hsa-miR-589-5p, hsa-miR-335-3p, hsa-miR-500a-3p, hsa-miR-671-5p, hsa-miR-1-3p, hsa-miR-887-3p, hsa-miR-320a-3p
DCTN4	15	hsa-miR-148a-3p, hsa-miR-20b-5p, hsa-miR-7-5p, hsa-miR-9-3p, hsa-miR-138-5p, hsa-miR-361-5p, hsa-miR-589-5p, hsa-miR-769-5p, hsa-

		miR-1275, hsa-miR-629-5p, hsa-miR-671-5p, hsa-miR-2110, hsa-miR-320c, hsa-miR-1-3p, hsa-miR-320a-3p
DENND5B	15	hsa-miR-20b-5p, hsa-miR-1307-3p, hsa-miR-148a-3p, hsa-miR-7-5p, hsa-miR-9-3p, hsa-miR-138-5p, hsa-miR-361-5p, hsa-miR-589-5p, hsa-miR-671-5p, hsa-miR-2110, hsa-miR-320c, hsa-miR-133a-3p, hsa-miR-887-3p, hsa-miR-320a-3p, hsa-miR-147b-3p
DHX9	15	hsa-miR-20b-5p, hsa-miR-335-5p, hsa-miR-7-5p, hsa-miR-9-3p, hsa-miR-138-5p, hsa-miR-361-5p, hsa-miR-320d, hsa-miR-335-3p, hsa-miR-671-5p, hsa-miR-2110, hsa-miR-320c, hsa-miR-378i, hsa-miR-1-3p, hsa-miR-887-3p, hsa-miR-320a-3p
DNAJB1	15	hsa-miR-1-3p, hsa-miR-20b-5p, hsa-miR-335-5p, hsa-miR-7-5p, hsa-miR-361-5p, hsa-miR-574-5p, hsa-miR-188-5p, hsa-miR-361-3p, hsa-miR-342-5p, hsa-miR-335-3p, hsa-miR-425-3p, hsa-miR-500a-3p, hsa-miR-671-5p, hsa-miR-320a-3p, hsa-miR-147b-3p
DNAJB14	15	hsa-miR-335-5p, hsa-miR-148a-3p, hsa-miR-20b-5p, hsa-miR-7-5p, hsa-miR-361-5p, hsa-miR-339-5p, hsa-miR-574-5p, hsa-miR-589-5p, hsa-miR-320d, hsa-miR-2110, hsa-miR-320c, hsa-miR-1-3p, hsa-miR-133a-3p, hsa-miR-12136, hsa-miR-320a-3p
DOCK7	15	hsa-miR-1307-3p, hsa-miR-148a-3p, hsa-miR-20b-5p, hsa-miR-335-5p, hsa-miR-7-5p, hsa-miR-138-5p, hsa-miR-361-5p, hsa-miR-574-5p, hsa-miR-188-5p, hsa-miR-29c-5p, hsa-miR-335-3p, hsa-miR-1307-5p, hsa-miR-1-3p, hsa-miR-320a-3p, hsa-miR-147b-3p
DOT1L	15	hsa-miR-148a-3p, hsa-miR-335-5p, hsa-miR-7-5p, hsa-miR-138-5p, hsa-miR-574-5p, hsa-miR-589-5p, hsa-miR-769-5p, hsa-miR-320d, hsa-miR-335-3p, hsa-miR-500a-3p, hsa-miR-1307-3p, hsa-miR-1307-5p, hsa-miR-320c, hsa-miR-3615, hsa-miR-320a-3p
ECPAS	15	hsa-miR-20b-5p, hsa-miR-335-5p, hsa-miR-7-5p, hsa-miR-9-3p, hsa-miR-138-5p, hsa-miR-361-5p, hsa-miR-339-5p, hsa-miR-589-5p, hsa-miR-320d, hsa-miR-335-3p, hsa-miR-500a-3p, hsa-miR-671-5p, hsa-miR-320c, hsa-miR-1-3p, hsa-miR-320a-3p
EGR1	15	hsa-miR-148a-3p, hsa-miR-20b-5p, hsa-miR-7-5p, hsa-miR-574-5p, hsa-miR-188-5p, hsa-miR-1275, hsa-miR-361-3p, hsa-miR-342-5p, hsa-miR-425-3p, hsa-miR-671-5p, hsa-miR-1307-3p, hsa-miR-2110, hsa-miR-887-3p, hsa-miR-320a-3p, hsa-miR-147b-3p

EIF4A2	15	hsa-miR-20b-5p, hsa-miR-148a-3p, hsa-miR-335-5p, hsa-miR-7-5p, hsa-miR-9-3p, hsa-miR-361-5p, hsa-miR-574-5p, hsa-miR-29c-5p, hsa-miR-335-3p, hsa-miR-1307-5p, hsa-miR-320c, hsa-miR-1468-5p, hsa-miR-1-3p, hsa-miR-133a-3p, hsa-miR-320a-3p
ELF1	15	hsa-miR-148a-3p, hsa-miR-20b-5p, hsa-miR-335-5p, hsa-miR-7-5p, hsa-miR-138-5p, hsa-miR-361-5p, hsa-miR-339-5p, hsa-miR-29c-5p, hsa-miR-335-3p, hsa-miR-500a-3p, hsa-miR-671-5p, hsa-miR-378i, hsa-miR-1-3p, hsa-miR-320a-3p, hsa-miR-147b-3p
ELOVL1	15	hsa-miR-148a-3p, hsa-miR-20b-5p, hsa-miR-335-5p, hsa-miR-7-5p, hsa-miR-361-5p, hsa-miR-339-5p, hsa-miR-574-5p, hsa-miR-589-5p, hsa-miR-629-5p, hsa-miR-361-3p, hsa-miR-342-5p, hsa-miR-500a-3p, hsa-miR-2110, hsa-miR-1-3p, hsa-miR-147b-3p
EP400	15	hsa-miR-148a-3p, hsa-miR-20b-5p, hsa-miR-335-5p, hsa-miR-7-5p, hsa-miR-138-5p, hsa-miR-339-5p, hsa-miR-574-5p, hsa-miR-589-5p, hsa-miR-335-3p, hsa-miR-671-5p, hsa-miR-320c, hsa-miR-874-3p, hsa-miR-1-3p, hsa-miR-320a-3p, hsa-miR-147b-3p
EPG5	15	hsa-miR-20b-5p, hsa-miR-335-5p, hsa-miR-7-5p, hsa-miR-138-5p, hsa-miR-361-5p, hsa-miR-589-5p, hsa-miR-1275, hsa-miR-342-5p, hsa-miR-335-3p, hsa-miR-671-5p, hsa-miR-320c, hsa-miR-378i, hsa-miR-887-3p, hsa-miR-320a-3p, hsa-miR-147b-3p
ETF1	15	hsa-miR-20b-5p, hsa-miR-335-5p, hsa-miR-7-5p, hsa-miR-138-5p, hsa-miR-361-5p, hsa-miR-574-5p, hsa-miR-589-5p, hsa-miR-769-5p, hsa-miR-320d, hsa-miR-29c-5p, hsa-miR-335-3p, hsa-miR-500a-3p, hsa-miR-320c, hsa-miR-887-3p, hsa-miR-320a-3p
ETNK1	15	hsa-miR-1-3p, hsa-miR-148a-3p, hsa-miR-20b-5p, hsa-miR-335-5p, hsa-miR-7-5p, hsa-miR-9-3p, hsa-miR-361-5p, hsa-miR-769-5p, hsa-miR-188-5p, hsa-miR-500a-3p, hsa-miR-551b-3p, hsa-miR-2110, hsa-miR-320c, hsa-miR-320a-3p, hsa-miR-147b-3p
F11R	15	hsa-miR-335-5p, hsa-miR-1-3p, hsa-miR-148a-3p, hsa-miR-7-5p, hsa-miR-361-5p, hsa-miR-339-5p, hsa-miR-574-5p, hsa-miR-769-5p, hsa-miR-320d, hsa-miR-335-3p, hsa-miR-425-3p, hsa-miR-500a-3p, hsa-miR-2110, hsa-miR-320c, hsa-miR-320a-3p
FADS2	15	hsa-miR-335-5p, hsa-miR-500a-3p, hsa-miR-148a-3p, hsa-miR-20b-5p, hsa-miR-7-5p, hsa-miR-138-5p, hsa-miR-361-5p, hsa-miR-188-5p, hsa-

		miR-320d, hsa-miR-629-5p, hsa-miR-425-3p, hsa-miR-1-3p, hsa-miR-133a-3p, hsa-miR-320a-3p, hsa-miR-147b-3p
FAF2	15	hsa-miR-20b-5p, hsa-miR-148a-3p, hsa-miR-335-5p, hsa-miR-7-5p, hsa-miR-361-5p, hsa-miR-589-5p, hsa-miR-188-5p, hsa-miR-629-5p, hsa-miR-335-3p, hsa-miR-500a-3p, hsa-miR-671-5p, hsa-miR-320c, hsa-miR-1-3p, hsa-miR-320a-3p, hsa-miR-147b-3p
FBRS	15	hsa-miR-1275, hsa-miR-342-5p, hsa-miR-671-5p, hsa-miR-887-3p, hsa-miR-148a-3p, hsa-miR-20b-5p, hsa-miR-335-5p, hsa-miR-7-5p, hsa-miR-138-5p, hsa-miR-769-5p, hsa-miR-361-3p, hsa-miR-425-3p, hsa-miR-1307-3p, hsa-miR-3615, hsa-miR-378i
FMR1	15	hsa-miR-148a-3p, hsa-miR-20b-5p, hsa-miR-335-5p, hsa-miR-7-5p, hsa-miR-138-5p, hsa-miR-361-5p, hsa-miR-574-5p, hsa-miR-320d, hsa-miR-29c-5p, hsa-miR-335-3p, hsa-miR-2110, hsa-miR-320c, hsa-miR-1-3p, hsa-miR-320a-3p, hsa-miR-147b-3p
FNIP1	15	hsa-miR-148a-3p, hsa-miR-20b-5p, hsa-miR-7-5p, hsa-miR-9-3p, hsa-miR-138-5p, hsa-miR-361-5p, hsa-miR-769-5p, hsa-miR-188-5p, hsa-miR-320d, hsa-miR-629-5p, hsa-miR-2110, hsa-miR-320c, hsa-miR-1-3p, hsa-miR-887-3p, hsa-miR-320a-3p
FOXK1	15	hsa-miR-20b-5p, hsa-miR-425-3p, hsa-miR-1307-3p, hsa-miR-335-5p, hsa-miR-7-5p, hsa-miR-361-5p, hsa-miR-574-5p, hsa-miR-589-5p, hsa-miR-320d, hsa-miR-1275, hsa-miR-361-3p, hsa-miR-335-3p, hsa-miR-671-5p, hsa-miR-320a-3p, hsa-miR-147b-3p
FOXM1	15	hsa-miR-671-5p, hsa-miR-148a-3p, hsa-miR-20b-5p, hsa-miR-7-5p, hsa-miR-138-5p, hsa-miR-361-5p, hsa-miR-769-5p, hsa-miR-320d, hsa-miR-361-3p, hsa-miR-500a-3p, hsa-miR-1307-5p, hsa-miR-2110, hsa-miR-320c, hsa-miR-1-3p, hsa-miR-320a-3p
FRY	15	hsa-miR-148a-3p, hsa-miR-20b-5p, hsa-miR-335-5p, hsa-miR-7-5p, hsa-miR-138-5p, hsa-miR-574-5p, hsa-miR-589-5p, hsa-miR-1275, hsa-miR-29c-5p, hsa-miR-500a-3p, hsa-miR-671-5p, hsa-miR-2110, hsa-miR-320c, hsa-miR-1-3p, hsa-miR-320a-3p
FRYL	15	hsa-miR-148a-3p, hsa-miR-20b-5p, hsa-miR-335-5p, hsa-miR-7-5p, hsa-miR-138-5p, hsa-miR-361-5p, hsa-miR-589-5p, hsa-miR-769-5p, hsa-miR-629-5p, hsa-miR-671-5p, hsa-miR-2110, hsa-miR-320c, hsa-miR-133a-3p, hsa-miR-320a-3p, hsa-miR-147b-3p

FXR2	15	hsa-miR-4516, hsa-miR-148a-3p, hsa-miR-20b-5p, hsa-miR-335-5p, hsa-miR-7-5p, hsa-miR-138-5p, hsa-miR-361-5p, hsa-miR-629-5p, hsa-miR-335-3p, hsa-miR-425-3p, hsa-miR-500a-3p, hsa-miR-1307-5p, hsa-miR-378i, hsa-miR-133a-3p, hsa-miR-320a-3p
FZD3	15	hsa-miR-148a-3p, hsa-miR-20b-5p, hsa-miR-7-5p, hsa-miR-361-5p, hsa-miR-589-5p, hsa-miR-188-5p, hsa-miR-29c-5p, hsa-miR-342-5p, hsa-miR-425-3p, hsa-miR-671-5p, hsa-miR-320c, hsa-miR-1-3p, hsa-miR-133a-3p, hsa-miR-887-3p, hsa-miR-320a-3p
GALNT2	15	hsa-miR-7-5p, hsa-miR-20b-5p, hsa-miR-138-5p, hsa-miR-361-5p, hsa-miR-574-5p, hsa-miR-589-5p, hsa-miR-769-5p, hsa-miR-320d, hsa-miR-629-5p, hsa-miR-1307-5p, hsa-miR-320c, hsa-miR-3615, hsa-miR-1-3p, hsa-miR-320a-3p, hsa-miR-147b-3p
GFPT1	15	hsa-miR-335-5p, hsa-miR-769-5p, hsa-miR-148a-3p, hsa-miR-20b-5p, hsa-miR-7-5p, hsa-miR-9-3p, hsa-miR-138-5p, hsa-miR-361-5p, hsa-miR-574-5p, hsa-miR-342-5p, hsa-miR-335-3p, hsa-miR-1307-3p, hsa-miR-320c, hsa-miR-320a-3p, hsa-miR-147b-3p
GHITM	15	hsa-miR-148a-3p, hsa-miR-20b-5p, hsa-miR-7-5p, hsa-miR-9-3p, hsa-miR-589-5p, hsa-miR-769-5p, hsa-miR-1275, hsa-miR-342-5p, hsa-miR-335-3p, hsa-miR-500a-3p, hsa-miR-320c, hsa-miR-1-3p, hsa-miR-133a-3p, hsa-miR-887-3p, hsa-miR-320a-3p
GLG1	15	hsa-miR-335-5p, hsa-miR-148a-3p, hsa-miR-20b-5p, hsa-miR-7-5p, hsa-miR-138-5p, hsa-miR-361-5p, hsa-miR-574-5p, hsa-miR-589-5p, hsa-miR-769-5p, hsa-miR-335-3p, hsa-miR-500a-3p, hsa-miR-671-5p, hsa-miR-1-3p, hsa-miR-320a-3p, hsa-miR-147b-3p
GNAI1	15	hsa-miR-9-3p, hsa-miR-320d, hsa-miR-320c, hsa-miR-1-3p, hsa-miR-148a-3p, hsa-miR-20b-5p, hsa-miR-138-5p, hsa-miR-361-5p, hsa-miR-339-5p, hsa-miR-574-5p, hsa-miR-589-5p, hsa-miR-335-3p, hsa-miR-3615, hsa-miR-887-3p, hsa-miR-320a-3p
GPD2	15	hsa-miR-335-5p, hsa-miR-1-3p, hsa-miR-148a-3p, hsa-miR-20b-5p, hsa-miR-9-3p, hsa-miR-138-5p, hsa-miR-361-5p, hsa-miR-574-5p, hsa-miR-320d, hsa-miR-629-5p, hsa-miR-671-5p, hsa-miR-2110, hsa-miR-320c, hsa-miR-133a-3p, hsa-miR-320a-3p
GPHN	15	hsa-miR-148a-3p, hsa-miR-20b-5p, hsa-miR-335-5p, hsa-miR-7-5p, hsa-miR-138-5p, hsa-miR-361-5p, hsa-miR-589-5p, hsa-miR-29c-5p, hsa-

		miR-425-3p, hsa-miR-500a-3p, hsa-miR-2110, hsa-miR-378i, hsa-miR-887-3p, hsa-miR-320a-3p, hsa-miR-190b-5p
GREB1	15	hsa-miR-335-5p, hsa-miR-148a-3p, hsa-miR-7-5p, hsa-miR-138-5p, hsa-miR-361-5p, hsa-miR-629-5p, hsa-miR-342-5p, hsa-miR-335-3p, hsa-miR-425-3p, hsa-miR-671-5p, hsa-miR-2110, hsa-miR-320c, hsa-miR-378i, hsa-miR-1-3p, hsa-miR-320a-3p
GSE1	15	hsa-miR-335-5p, hsa-miR-769-5p, hsa-miR-29c-5p, hsa-miR-361-3p, hsa-miR-148a-3p, hsa-miR-20b-5p, hsa-miR-7-5p, hsa-miR-138-5p, hsa-miR-574-5p, hsa-miR-589-5p, hsa-miR-629-5p, hsa-miR-335-3p, hsa-miR-425-3p, hsa-miR-500a-3p, hsa-miR-147b-3p
GTF3C1	15	hsa-miR-20b-5p, hsa-miR-335-5p, hsa-miR-7-5p, hsa-miR-138-5p, hsa-miR-361-5p, hsa-miR-339-5p, hsa-miR-574-5p, hsa-miR-769-5p, hsa-miR-629-5p, hsa-miR-29c-5p, hsa-miR-335-3p, hsa-miR-500a-3p, hsa-miR-1-3p, hsa-miR-320a-3p, hsa-miR-147b-3p
GTF3C4	15	hsa-miR-188-5p, hsa-miR-148a-3p, hsa-miR-20b-5p, hsa-miR-7-5p, hsa-miR-138-5p, hsa-miR-361-5p, hsa-miR-769-5p, hsa-miR-1275, hsa-miR-342-5p, hsa-miR-335-3p, hsa-miR-671-5p, hsa-miR-2110, hsa-miR-320c, hsa-miR-874-3p, hsa-miR-320a-3p
HDGF	15	hsa-miR-20b-5p, hsa-miR-342-5p, hsa-miR-148a-3p, hsa-miR-7-5p, hsa-miR-138-5p, hsa-miR-361-5p, hsa-miR-335-3p, hsa-miR-425-3p, hsa-miR-500a-3p, hsa-miR-671-5p, hsa-miR-2110, hsa-miR-320c, hsa-miR-3615, hsa-miR-320a-3p, hsa-miR-147b-3p
HM13	15	hsa-miR-148a-3p, hsa-miR-20b-5p, hsa-miR-335-5p, hsa-miR-7-5p, hsa-miR-138-5p, hsa-miR-361-5p, hsa-miR-339-5p, hsa-miR-574-5p, hsa-miR-335-3p, hsa-miR-500a-3p, hsa-miR-671-5p, hsa-miR-320c, hsa-miR-1-3p, hsa-miR-320a-3p, hsa-miR-147b-3p
HMGA1	15	hsa-miR-335-5p, hsa-miR-138-5p, hsa-miR-671-5p, hsa-miR-148a-3p, hsa-miR-20b-5p, hsa-miR-7-5p, hsa-miR-320d, hsa-miR-629-5p, hsa-miR-361-3p, hsa-miR-500a-3p, hsa-miR-2110, hsa-miR-378i, hsa-miR-874-3p, hsa-miR-1-3p, hsa-miR-320a-3p
HNRNPDL	15	hsa-miR-339-5p, hsa-miR-148a-3p, hsa-miR-20b-5p, hsa-miR-335-5p, hsa-miR-7-5p, hsa-miR-574-5p, hsa-miR-188-5p, hsa-miR-629-5p, hsa-miR-361-3p, hsa-miR-335-3p, hsa-miR-671-5p, hsa-miR-1307-5p, hsa-miR-3615, hsa-miR-133a-3p, hsa-miR-320a-3p

HSPA8	15	hsa-miR-20b-5p, hsa-miR-7-5p, hsa-miR-138-5p, hsa-miR-361-5p, hsa-miR-188-5p, hsa-miR-629-5p, hsa-miR-29c-5p, hsa-miR-361-3p, hsa-miR-335-3p, hsa-miR-500a-3p, hsa-miR-1307-5p, hsa-miR-2110, hsa-miR-320c, hsa-miR-320a-3p, hsa-miR-147b-3p
HYCC2	15	hsa-miR-20b-5p, hsa-miR-7-5p, hsa-miR-769-5p, hsa-miR-335-3p, hsa-miR-148a-3p, hsa-miR-335-5p, hsa-miR-138-5p, hsa-miR-361-5p, hsa-miR-574-5p, hsa-miR-29c-5p, hsa-miR-500a-3p, hsa-miR-2110, hsa-miR-320c, hsa-miR-133a-3p, hsa-miR-320a-3p
IGF2BP2	15	hsa-miR-335-5p, hsa-miR-7-5p, hsa-miR-9-3p, hsa-miR-138-5p, hsa-miR-1275, hsa-miR-629-5p, hsa-miR-335-3p, hsa-miR-425-3p, hsa-miR-500a-3p, hsa-miR-671-5p, hsa-miR-1307-3p, hsa-miR-2110, hsa-miR-378i, hsa-miR-887-3p, hsa-miR-320a-3p
IGF2R	15	hsa-miR-148a-3p, hsa-miR-20b-5p, hsa-miR-7-5p, hsa-miR-138-5p, hsa-miR-339-5p, hsa-miR-589-5p, hsa-miR-188-5p, hsa-miR-1275, hsa-miR-335-3p, hsa-miR-500a-3p, hsa-miR-874-3p, hsa-miR-1-3p, hsa-miR-887-3p, hsa-miR-320a-3p, hsa-miR-147b-3p
IPO5	15	hsa-miR-148a-3p, hsa-miR-20b-5p, hsa-miR-7-5p, hsa-miR-9-3p, hsa-miR-138-5p, hsa-miR-361-5p, hsa-miR-574-5p, hsa-miR-629-5p, hsa-miR-335-3p, hsa-miR-671-5p, hsa-miR-1307-5p, hsa-miR-320c, hsa-miR-378i, hsa-miR-133a-3p, hsa-miR-320a-3p
IQGAP3	15	hsa-miR-1-3p, hsa-miR-148a-3p, hsa-miR-20b-5p, hsa-miR-335-5p, hsa-miR-138-5p, hsa-miR-361-5p, hsa-miR-574-5p, hsa-miR-1275, hsa-miR-629-5p, hsa-miR-335-3p, hsa-miR-500a-3p, hsa-miR-671-5p, hsa-miR-378i, hsa-miR-320a-3p, hsa-miR-147b-3p
ITGA6	15	hsa-miR-1-3p, hsa-miR-148a-3p, hsa-miR-20b-5p, hsa-miR-335-5p, hsa-miR-7-5p, hsa-miR-361-5p, hsa-miR-1275, hsa-miR-29c-5p, hsa-miR-335-3p, hsa-miR-500a-3p, hsa-miR-671-5p, hsa-miR-2110, hsa-miR-3615, hsa-miR-378i, hsa-miR-147b-3p
JARID2	15	hsa-miR-148a-3p, hsa-miR-7-5p, hsa-miR-342-5p, hsa-miR-20b-5p, hsa-miR-9-3p, hsa-miR-361-5p, hsa-miR-188-5p, hsa-miR-29c-5p, hsa-miR-361-3p, hsa-miR-335-3p, hsa-miR-500a-3p, hsa-miR-671-5p, hsa-miR-1307-5p, hsa-miR-1-3p, hsa-miR-320a-3p
KIAA0232	15	hsa-miR-20b-5p, hsa-miR-148a-3p, hsa-miR-7-5p, hsa-miR-138-5p, hsa-miR-361-5p, hsa-miR-339-5p, hsa-miR-574-5p, hsa-miR-629-5p, hsa-

		miR-29c-5p, hsa-miR-425-3p, hsa-miR-500a-3p, hsa-miR-378i, hsa-miR-874-3p, hsa-miR-133a-3p, hsa-miR-320a-3p
KIF13A	15	hsa-miR-148a-3p, hsa-miR-20b-5p, hsa-miR-7-5p, hsa-miR-138-5p, hsa-miR-361-5p, hsa-miR-769-5p, hsa-miR-342-5p, hsa-miR-335-3p, hsa-miR-500a-3p, hsa-miR-320c, hsa-miR-378i, hsa-miR-1-3p, hsa-miR-887-3p, hsa-miR-320a-3p, hsa-miR-147b-3p
KIF3B	15	hsa-miR-874-3p, hsa-miR-20b-5p, hsa-miR-339-5p, hsa-miR-769-5p, hsa-miR-1275, hsa-miR-29c-5p, hsa-miR-335-3p, hsa-miR-2110, hsa-miR-320c, hsa-miR-378i, hsa-miR-1-3p, hsa-miR-133a-3p, hsa-miR-320a-3p, hsa-miR-147b-3p, hsa-miR-190b-5p
KIF5A	15	hsa-miR-148a-3p, hsa-miR-20b-5p, hsa-miR-7-5p, hsa-miR-138-5p, hsa-miR-361-5p, hsa-miR-574-5p, hsa-miR-1275, hsa-miR-671-5p, hsa-miR-2110, hsa-miR-320c, hsa-miR-378i, hsa-miR-1-3p, hsa-miR-133a-3p, hsa-miR-887-3p, hsa-miR-320a-3p
KLHL11	15	hsa-miR-335-5p, hsa-miR-9-3p, hsa-miR-769-5p, hsa-miR-500a-3p, hsa-miR-148a-3p, hsa-miR-20b-5p, hsa-miR-7-5p, hsa-miR-361-5p, hsa-miR-574-5p, hsa-miR-589-5p, hsa-miR-629-5p, hsa-miR-342-5p, hsa-miR-335-3p, hsa-miR-320c, hsa-miR-320a-3p
KLHL24	15	hsa-miR-335-5p, hsa-miR-20b-5p, hsa-miR-7-5p, hsa-miR-138-5p, hsa-miR-361-5p, hsa-miR-574-5p, hsa-miR-589-5p, hsa-miR-188-5p, hsa-miR-629-5p, hsa-miR-335-3p, hsa-miR-500a-3p, hsa-miR-671-5p, hsa-miR-2110, hsa-miR-1-3p, hsa-miR-320a-3p
LIMA1	15	hsa-miR-20b-5p, hsa-miR-339-5p, hsa-miR-7-5p, hsa-miR-9-3p, hsa-miR-361-5p, hsa-miR-574-5p, hsa-miR-769-5p, hsa-miR-320d, hsa-miR-629-5p, hsa-miR-342-5p, hsa-miR-500a-3p, hsa-miR-671-5p, hsa-miR-320c, hsa-miR-874-3p, hsa-miR-320a-3p
LONRF1	15	hsa-miR-335-5p, hsa-miR-574-5p, hsa-miR-769-5p, hsa-miR-148a-3p, hsa-miR-20b-5p, hsa-miR-7-5p, hsa-miR-9-3p, hsa-miR-589-5p, hsa-miR-320d, hsa-miR-629-5p, hsa-miR-335-3p, hsa-miR-671-5p, hsa-miR-320c, hsa-miR-887-3p, hsa-miR-320a-3p
LPP	15	hsa-miR-574-5p, hsa-miR-148a-3p, hsa-miR-20b-5p, hsa-miR-335-5p, hsa-miR-7-5p, hsa-miR-361-5p, hsa-miR-769-5p, hsa-miR-342-5p, hsa-miR-335-3p, hsa-miR-425-3p, hsa-miR-671-5p, hsa-miR-2110, hsa-miR-320c, hsa-miR-1-3p, hsa-miR-320a-3p

LRBA	15	hsa-miR-769-5p, hsa-miR-20b-5p, hsa-miR-7-5p, hsa-miR-138-5p, hsa-miR-361-5p, hsa-miR-574-5p, hsa-miR-320d, hsa-miR-335-3p, hsa-miR-500a-3p, hsa-miR-671-5p, hsa-miR-320c, hsa-miR-1-3p, hsa-miR-133a-3p, hsa-miR-320a-3p, hsa-miR-147b-3p
LRIG2	15	hsa-miR-1307-3p, hsa-miR-4516, hsa-miR-148a-3p, hsa-miR-20b-5p, hsa-miR-7-5p, hsa-miR-138-5p, hsa-miR-361-5p, hsa-miR-589-5p, hsa-miR-188-5p, hsa-miR-320d, hsa-miR-342-5p, hsa-miR-500a-3p, hsa-miR-2110, hsa-miR-320c, hsa-miR-320a-3p
LRP1B	15	hsa-miR-335-5p, hsa-miR-148a-3p, hsa-miR-20b-5p, hsa-miR-7-5p, hsa-miR-138-5p, hsa-miR-361-5p, hsa-miR-589-5p, hsa-miR-629-5p, hsa-miR-335-3p, hsa-miR-500a-3p, hsa-miR-2110, hsa-miR-320c, hsa-miR-1-3p, hsa-miR-133a-3p, hsa-miR-320a-3p
LRRC8A	15	hsa-miR-335-5p, hsa-miR-1-3p, hsa-miR-148a-3p, hsa-miR-20b-5p, hsa-miR-7-5p, hsa-miR-138-5p, hsa-miR-574-5p, hsa-miR-361-3p, hsa-miR-335-3p, hsa-miR-500a-3p, hsa-miR-1307-3p, hsa-miR-3615, hsa-miR-887-3p, hsa-miR-320a-3p, hsa-miR-147b-3p
LSM14A	15	hsa-miR-320c, hsa-miR-7-5p, hsa-miR-138-5p, hsa-miR-361-5p, hsa-miR-339-5p, hsa-miR-574-5p, hsa-miR-589-5p, hsa-miR-769-5p, hsa-miR-629-5p, hsa-miR-342-5p, hsa-miR-335-3p, hsa-miR-2110, hsa-miR-378i, hsa-miR-1-3p, hsa-miR-320a-3p
LTN1	15	hsa-miR-335-5p, hsa-miR-148a-3p, hsa-miR-20b-5p, hsa-miR-7-5p, hsa-miR-138-5p, hsa-miR-361-5p, hsa-miR-589-5p, hsa-miR-629-5p, hsa-miR-342-5p, hsa-miR-335-3p, hsa-miR-500a-3p, hsa-miR-320c, hsa-miR-3615, hsa-miR-1-3p, hsa-miR-320a-3p
LUC7L3	15	hsa-miR-9-3p, hsa-miR-148a-3p, hsa-miR-20b-5p, hsa-miR-7-5p, hsa-miR-361-5p, hsa-miR-574-5p, hsa-miR-769-5p, hsa-miR-188-5p, hsa-miR-2110, hsa-miR-320c, hsa-miR-378i, hsa-miR-1-3p, hsa-miR-133a-3p, hsa-miR-887-3p, hsa-miR-320a-3p
MAST4	15	hsa-miR-1-3p, hsa-miR-148a-3p, hsa-miR-20b-5p, hsa-miR-7-5p, hsa-miR-138-5p, hsa-miR-574-5p, hsa-miR-589-5p, hsa-miR-629-5p, hsa-miR-342-5p, hsa-miR-500a-3p, hsa-miR-671-5p, hsa-miR-1307-5p, hsa-miR-1468-5p, hsa-miR-320a-3p, hsa-miR-147b-3p
MBD3	15	hsa-miR-339-5p, hsa-miR-574-5p, hsa-miR-20b-5p, hsa-miR-335-5p, hsa-miR-7-5p, hsa-miR-188-5p, hsa-miR-335-3p, hsa-miR-500a-3p, hsa-

		miR-671-5p, hsa-miR-1307-5p, hsa-miR-3615, hsa-miR-1-3p, hsa-miR-133a-3p, hsa-miR-320a-3p, hsa-miR-147b-3p
MBNL2	15	hsa-miR-335-5p, hsa-miR-148a-3p, hsa-miR-20b-5p, hsa-miR-7-5p, hsa-miR-138-5p, hsa-miR-361-5p, hsa-miR-574-5p, hsa-miR-629-5p, hsa-miR-551b-3p, hsa-miR-671-5p, hsa-miR-2110, hsa-miR-1468-5p, hsa-miR-1-3p, hsa-miR-133a-3p, hsa-miR-190b-5p
MCMBP	15	hsa-miR-342-5p, hsa-miR-551b-3p, hsa-miR-20b-5p, hsa-miR-335-5p, hsa-miR-7-5p, hsa-miR-138-5p, hsa-miR-361-5p, hsa-miR-574-5p, hsa-miR-589-5p, hsa-miR-1275, hsa-miR-335-3p, hsa-miR-671-5p, hsa-miR-2110, hsa-miR-1-3p, hsa-miR-320a-3p
MKLN1	15	hsa-miR-148a-3p, hsa-miR-20b-5p, hsa-miR-335-5p, hsa-miR-7-5p, hsa-miR-574-5p, hsa-miR-188-5p, hsa-miR-629-5p, hsa-miR-335-3p, hsa-miR-425-3p, hsa-miR-671-5p, hsa-miR-1307-3p, hsa-miR-320c, hsa-miR-3615, hsa-miR-1-3p, hsa-miR-320a-3p
MMD	15	hsa-miR-1-3p, hsa-miR-335-5p, hsa-miR-148a-3p, hsa-miR-20b-5p, hsa-miR-7-5p, hsa-miR-138-5p, hsa-miR-361-5p, hsa-miR-574-5p, hsa-miR-589-5p, hsa-miR-335-3p, hsa-miR-671-5p, hsa-miR-2110, hsa-miR-320c, hsa-miR-133a-3p, hsa-miR-320a-3p
MSI2	15	hsa-miR-20b-5p, hsa-miR-138-5p, hsa-miR-574-5p, hsa-miR-769-5p, hsa-miR-320d, hsa-miR-629-5p, hsa-miR-342-5p, hsa-miR-2110, hsa-miR-320c, hsa-miR-378i, hsa-miR-1-3p, hsa-miR-887-3p, hsa-miR-320a-3p, hsa-miR-147b-3p, hsa-miR-190b-5p
MT-ND1	15	hsa-miR-320c, hsa-miR-1-3p, hsa-miR-148a-3p, hsa-miR-7-5p, hsa-miR-138-5p, hsa-miR-339-5p, hsa-miR-574-5p, hsa-miR-1275, hsa-miR-629-5p, hsa-miR-342-5p, hsa-miR-335-3p, hsa-miR-671-5p, hsa-miR-887-3p, hsa-miR-320a-3p, hsa-miR-147b-3p
MYH14	15	hsa-miR-1275, hsa-miR-335-5p, hsa-miR-7-5p, hsa-miR-138-5p, hsa-miR-589-5p, hsa-miR-769-5p, hsa-miR-320d, hsa-miR-361-3p, hsa-miR-425-3p, hsa-miR-500a-3p, hsa-miR-671-5p, hsa-miR-320c, hsa-miR-3615, hsa-miR-320a-3p, hsa-miR-147b-3p
MYO1C	15	hsa-miR-148a-3p, hsa-miR-20b-5p, hsa-miR-335-5p, hsa-miR-7-5p, hsa-miR-138-5p, hsa-miR-361-5p, hsa-miR-339-5p, hsa-miR-769-5p, hsa-miR-320d, hsa-miR-1275, hsa-miR-361-3p, hsa-miR-671-5p, hsa-miR-2110, hsa-miR-320c, hsa-miR-320a-3p

MYO1D	15	hsa-miR-20b-5p, hsa-miR-148a-3p, hsa-miR-7-5p, hsa-miR-138-5p, hsa-miR-361-5p, hsa-miR-589-5p, hsa-miR-769-5p, hsa-miR-188-5p, hsa-miR-335-3p, hsa-miR-2110, hsa-miR-320c, hsa-miR-378i, hsa-miR-1-3p, hsa-miR-133a-3p, hsa-miR-887-3p
NCDN	15	hsa-miR-335-5p, hsa-miR-574-5p, hsa-miR-629-5p, hsa-miR-133a-3p, hsa-miR-148a-3p, hsa-miR-20b-5p, hsa-miR-7-5p, hsa-miR-9-3p, hsa-miR-361-5p, hsa-miR-320d, hsa-miR-500a-3p, hsa-miR-671-5p, hsa-miR-2110, hsa-miR-320c, hsa-miR-320a-3p
NCOA1	15	hsa-miR-148a-3p, hsa-miR-20b-5p, hsa-miR-7-5p, hsa-miR-138-5p, hsa-miR-361-5p, hsa-miR-589-5p, hsa-miR-320d, hsa-miR-629-5p, hsa-miR-342-5p, hsa-miR-500a-3p, hsa-miR-671-5p, hsa-miR-320c, hsa-miR-378i, hsa-miR-874-3p, hsa-miR-320a-3p
NDRG1	15	hsa-miR-148a-3p, hsa-miR-335-5p, hsa-miR-20b-5p, hsa-miR-7-5p, hsa-miR-361-5p, hsa-miR-574-5p, hsa-miR-589-5p, hsa-miR-1275, hsa-miR-425-3p, hsa-miR-1307-5p, hsa-miR-1-3p, hsa-miR-133a-3p, hsa-miR-887-3p, hsa-miR-320a-3p, hsa-miR-147b-3p
NF1	15	hsa-miR-148a-3p, hsa-miR-20b-5p, hsa-miR-7-5p, hsa-miR-138-5p, hsa-miR-361-5p, hsa-miR-339-5p, hsa-miR-574-5p, hsa-miR-320d, hsa-miR-500a-3p, hsa-miR-671-5p, hsa-miR-320c, hsa-miR-378i, hsa-miR-1-3p, hsa-miR-320a-3p, hsa-miR-147b-3p
NFKB1	15	hsa-miR-9-3p, hsa-miR-138-5p, hsa-miR-148a-3p, hsa-miR-20b-5p, hsa-miR-335-5p, hsa-miR-7-5p, hsa-miR-361-5p, hsa-miR-320d, hsa-miR-335-3p, hsa-miR-500a-3p, hsa-miR-1307-3p, hsa-miR-320c, hsa-miR-1-3p, hsa-miR-133a-3p, hsa-miR-320a-3p
NKTR	15	hsa-miR-335-5p, hsa-miR-148a-3p, hsa-miR-20b-5p, hsa-miR-7-5p, hsa-miR-361-5p, hsa-miR-589-5p, hsa-miR-769-5p, hsa-miR-629-5p, hsa-miR-342-5p, hsa-miR-335-3p, hsa-miR-2110, hsa-miR-320c, hsa-miR-1-3p, hsa-miR-133a-3p, hsa-miR-320a-3p
NPAS2	15	hsa-miR-335-5p, hsa-miR-148a-3p, hsa-miR-20b-5p, hsa-miR-7-5p, hsa-miR-138-5p, hsa-miR-574-5p, hsa-miR-589-5p, hsa-miR-320d, hsa-miR-629-5p, hsa-miR-335-3p, hsa-miR-1307-5p, hsa-miR-320c, hsa-miR-1-3p, hsa-miR-320a-3p, hsa-miR-147b-3p
NPAT	15	hsa-miR-20b-5p, hsa-miR-148a-3p, hsa-miR-335-5p, hsa-miR-7-5p, hsa-miR-361-5p, hsa-miR-589-5p, hsa-miR-320d, hsa-miR-629-5p, hsa-miR-

		342-5p, hsa-miR-335-3p, hsa-miR-320c, hsa-miR-378i, hsa-miR-1-3p, hsa-miR-320a-3p, hsa-miR-147b-3p
NPEPPS	15	hsa-miR-148a-3p, hsa-miR-20b-5p, hsa-miR-335-5p, hsa-miR-7-5p, hsa-miR-138-5p, hsa-miR-574-5p, hsa-miR-188-5p, hsa-miR-361-3p, hsa-miR-335-3p, hsa-miR-500a-3p, hsa-miR-551b-3p, hsa-miR-671-5p, hsa-miR-2110, hsa-miR-133a-3p, hsa-miR-320a-3p
NRAS	15	hsa-miR-7-5p, hsa-miR-148a-3p, hsa-miR-20b-5p, hsa-miR-335-5p, hsa-miR-361-5p, hsa-miR-574-5p, hsa-miR-629-5p, hsa-miR-29c-5p, hsa-miR-335-3p, hsa-miR-425-3p, hsa-miR-500a-3p, hsa-miR-671-5p, hsa-miR-2110, hsa-miR-3615, hsa-miR-320a-3p
NT5DC2	15	hsa-miR-148a-3p, hsa-miR-20b-5p, hsa-miR-361-5p, hsa-miR-769-5p, hsa-miR-188-5p, hsa-miR-320d, hsa-miR-342-5p, hsa-miR-335-3p, hsa-miR-671-5p, hsa-miR-1307-3p, hsa-miR-320c, hsa-miR-1-3p, hsa-miR-133a-3p, hsa-miR-887-3p, hsa-miR-320a-3p
OTUD7B	15	hsa-miR-339-5p, hsa-miR-500a-3p, hsa-miR-4516, hsa-miR-148a-3p, hsa-miR-20b-5p, hsa-miR-335-5p, hsa-miR-7-5p, hsa-miR-9-3p, hsa-miR-361-5p, hsa-miR-629-5p, hsa-miR-335-3p, hsa-miR-425-3p, hsa-miR-671-5p, hsa-miR-2110, hsa-miR-320a-3p
OXA1L	15	hsa-miR-671-5p, hsa-miR-1307-3p, hsa-miR-148a-3p, hsa-miR-20b-5p, hsa-miR-335-5p, hsa-miR-7-5p, hsa-miR-769-5p, hsa-miR-629-5p, hsa-miR-342-5p, hsa-miR-335-3p, hsa-miR-1307-5p, hsa-miR-3168, hsa-miR-2110, hsa-miR-378i, hsa-miR-1-3p
PCBP1	15	hsa-miR-148a-3p, hsa-miR-20b-5p, hsa-miR-361-5p, hsa-miR-339-5p, hsa-miR-589-5p, hsa-miR-1275, hsa-miR-29c-5p, hsa-miR-361-3p, hsa-miR-335-3p, hsa-miR-500a-3p, hsa-miR-671-5p, hsa-miR-2110, hsa-miR-1-3p, hsa-miR-133a-3p, hsa-miR-320a-3p
PCF11	15	hsa-miR-148a-3p, hsa-miR-335-5p, hsa-miR-7-5p, hsa-miR-9-3p, hsa-miR-138-5p, hsa-miR-361-5p, hsa-miR-769-5p, hsa-miR-29c-5p, hsa-miR-335-3p, hsa-miR-500a-3p, hsa-miR-1307-3p, hsa-miR-320c, hsa-miR-378i, hsa-miR-1-3p, hsa-miR-320a-3p
PDS5A	15	hsa-miR-335-3p, hsa-miR-148a-3p, hsa-miR-20b-5p, hsa-miR-335-5p, hsa-miR-7-5p, hsa-miR-361-5p, hsa-miR-574-5p, hsa-miR-361-3p, hsa-miR-320c, hsa-miR-378i, hsa-miR-874-3p, hsa-miR-1-3p, hsa-miR-320a-3p, hsa-miR-147b-3p, hsa-miR-190b-5p

PDS5B	15	hsa-miR-148a-3p, hsa-miR-20b-5p, hsa-miR-335-5p, hsa-miR-7-5p, hsa-miR-138-5p, hsa-miR-361-5p, hsa-miR-342-5p, hsa-miR-335-3p, hsa-miR-500a-3p, hsa-miR-671-5p, hsa-miR-320c, hsa-miR-1-3p, hsa-miR-133a-3p, hsa-miR-887-3p, hsa-miR-320a-3p
PEG10	15	hsa-miR-148a-3p, hsa-miR-20b-5p, hsa-miR-7-5p, hsa-miR-9-3p, hsa-miR-361-5p, hsa-miR-320d, hsa-miR-629-5p, hsa-miR-342-5p, hsa-miR-335-3p, hsa-miR-500a-3p, hsa-miR-3168, hsa-miR-320c, hsa-miR-1-3p, hsa-miR-320a-3p, hsa-miR-147b-3p
PGD	15	hsa-miR-339-5p, hsa-miR-1-3p, hsa-miR-148a-3p, hsa-miR-7-5p, hsa-miR-138-5p, hsa-miR-361-5p, hsa-miR-574-5p, hsa-miR-320d, hsa-miR-335-3p, hsa-miR-500a-3p, hsa-miR-671-5p, hsa-miR-320c, hsa-miR-3615, hsa-miR-133a-3p, hsa-miR-320a-3p
PHB2	15	hsa-miR-1275, hsa-miR-361-3p, hsa-miR-148a-3p, hsa-miR-20b-5p, hsa-miR-335-5p, hsa-miR-138-5p, hsa-miR-361-5p, hsa-miR-574-5p, hsa-miR-589-5p, hsa-miR-335-3p, hsa-miR-425-3p, hsa-miR-500a-3p, hsa-miR-671-5p, hsa-miR-1307-3p, hsa-miR-320a-3p
PHC2	15	hsa-miR-7-5p, hsa-miR-4516, hsa-miR-148a-3p, hsa-miR-20b-5p, hsa-miR-335-5p, hsa-miR-574-5p, hsa-miR-589-5p, hsa-miR-1275, hsa-miR-361-3p, hsa-miR-500a-3p, hsa-miR-671-5p, hsa-miR-2110, hsa-miR-1-3p, hsa-miR-320a-3p, hsa-miR-147b-3p
PI4KA	15	hsa-miR-148a-3p, hsa-miR-20b-5p, hsa-miR-335-5p, hsa-miR-7-5p, hsa-miR-138-5p, hsa-miR-361-5p, hsa-miR-342-5p, hsa-miR-425-3p, hsa-miR-671-5p, hsa-miR-1307-3p, hsa-miR-2110, hsa-miR-320c, hsa-miR-378i, hsa-miR-1-3p, hsa-miR-320a-3p
PIK3CB	15	hsa-miR-7-5p, hsa-miR-148a-3p, hsa-miR-20b-5p, hsa-miR-335-5p, hsa-miR-361-5p, hsa-miR-339-5p, hsa-miR-574-5p, hsa-miR-589-5p, hsa-miR-629-5p, hsa-miR-500a-3p, hsa-miR-671-5p, hsa-miR-2110, hsa-miR-320c, hsa-miR-320a-3p, hsa-miR-147b-3p
PIM2	15	hsa-miR-4516, hsa-miR-148a-3p, hsa-miR-7-5p, hsa-miR-138-5p, hsa-miR-361-5p, hsa-miR-574-5p, hsa-miR-188-5p, hsa-miR-320d, hsa-miR-629-5p, hsa-miR-671-5p, hsa-miR-2110, hsa-miR-320c, hsa-miR-378i, hsa-miR-1-3p, hsa-miR-320a-3p
PJA2	15	hsa-miR-335-5p, hsa-miR-148a-3p, hsa-miR-20b-5p, hsa-miR-7-5p, hsa-miR-138-5p, hsa-miR-361-5p, hsa-miR-339-5p, hsa-miR-574-5p, hsa-

		miR-589-5p, hsa-miR-335-3p, hsa-miR-320c, hsa-miR-1-3p, hsa-miR-133a-3p, hsa-miR-320a-3p, hsa-miR-190b-5p
PLAGL2	15	hsa-miR-20b-5p, hsa-miR-335-5p, hsa-miR-7-5p, hsa-miR-138-5p, hsa-miR-671-5p, hsa-miR-1-3p, hsa-miR-148a-3p, hsa-miR-574-5p, hsa-miR-589-5p, hsa-miR-769-5p, hsa-miR-629-5p, hsa-miR-335-3p, hsa-miR-2110, hsa-miR-320c, hsa-miR-320a-3p
PLXNA2	15	hsa-miR-4516, hsa-miR-148a-3p, hsa-miR-20b-5p, hsa-miR-7-5p, hsa-miR-9-3p, hsa-miR-138-5p, hsa-miR-339-5p, hsa-miR-342-5p, hsa-miR-671-5p, hsa-miR-2110, hsa-miR-320c, hsa-miR-378i, hsa-miR-1-3p, hsa-miR-133a-3p, hsa-miR-320a-3p
POLR2B	15	hsa-miR-148a-3p, hsa-miR-20b-5p, hsa-miR-335-5p, hsa-miR-7-5p, hsa-miR-361-5p, hsa-miR-320d, hsa-miR-1275, hsa-miR-361-3p, hsa-miR-335-3p, hsa-miR-500a-3p, hsa-miR-1307-5p, hsa-miR-2110, hsa-miR-320c, hsa-miR-320a-3p, hsa-miR-190b-5p
PPP1R9B	15	hsa-miR-342-5p, hsa-miR-148a-3p, hsa-miR-20b-5p, hsa-miR-335-5p, hsa-miR-574-5p, hsa-miR-769-5p, hsa-miR-320d, hsa-miR-335-3p, hsa-miR-500a-3p, hsa-miR-671-5p, hsa-miR-1307-5p, hsa-miR-320c, hsa-miR-3615, hsa-miR-1-3p, hsa-miR-320a-3p
PPP3CA	15	hsa-miR-148a-3p, hsa-miR-20b-5p, hsa-miR-7-5p, hsa-miR-138-5p, hsa-miR-361-5p, hsa-miR-769-5p, hsa-miR-1275, hsa-miR-629-5p, hsa-miR-342-5p, hsa-miR-335-3p, hsa-miR-500a-3p, hsa-miR-671-5p, hsa-miR-378i, hsa-miR-1468-5p, hsa-miR-147b-3p
PPP4R3A	15	hsa-miR-1-3p, hsa-miR-148a-3p, hsa-miR-20b-5p, hsa-miR-7-5p, hsa-miR-138-5p, hsa-miR-361-5p, hsa-miR-574-5p, hsa-miR-589-5p, hsa-miR-335-3p, hsa-miR-500a-3p, hsa-miR-671-5p, hsa-miR-320c, hsa-miR-378i, hsa-miR-320a-3p, hsa-miR-147b-3p
PPP6R1	15	hsa-miR-148a-3p, hsa-miR-3615, hsa-miR-20b-5p, hsa-miR-335-5p, hsa-miR-7-5p, hsa-miR-138-5p, hsa-miR-361-5p, hsa-miR-574-5p, hsa-miR-769-5p, hsa-miR-629-5p, hsa-miR-335-3p, hsa-miR-671-5p, hsa-miR-378i, hsa-miR-1-3p, hsa-miR-147b-3p
PPP6R3	15	hsa-miR-20b-5p, hsa-miR-188-5p, hsa-miR-148a-3p, hsa-miR-335-5p, hsa-miR-7-5p, hsa-miR-138-5p, hsa-miR-361-5p, hsa-miR-1275, hsa-miR-335-3p, hsa-miR-500a-3p, hsa-miR-671-5p, hsa-miR-320c, hsa-miR-1-3p, hsa-miR-320a-3p, hsa-miR-147b-3p

PRDM1	15	hsa-miR-320c, hsa-miR-874-3p, hsa-miR-148a-3p, hsa-miR-20b-5p, hsa-miR-7-5p, hsa-miR-361-5p, hsa-miR-574-5p, hsa-miR-769-5p, hsa-miR-1275, hsa-miR-342-5p, hsa-miR-500a-3p, hsa-miR-671-5p, hsa-miR-2110, hsa-miR-320a-3p, hsa-miR-147b-3p
PRDM4	15	hsa-miR-20b-5p, hsa-miR-335-5p, hsa-miR-7-5p, hsa-miR-9-3p, hsa-miR-574-5p, hsa-miR-320d, hsa-miR-1275, hsa-miR-342-5p, hsa-miR-335-3p, hsa-miR-500a-3p, hsa-miR-671-5p, hsa-miR-1307-3p, hsa-miR-320c, hsa-miR-320a-3p, hsa-miR-147b-3p
PRKCE	15	hsa-miR-1-3p, hsa-miR-20b-5p, hsa-miR-7-5p, hsa-miR-361-5p, hsa-miR-574-5p, hsa-miR-589-5p, hsa-miR-769-5p, hsa-miR-188-5p, hsa-miR-500a-3p, hsa-miR-671-5p, hsa-miR-320c, hsa-miR-874-3p, hsa-miR-887-3p, hsa-miR-320a-3p, hsa-miR-147b-3p
PRPF4B	15	hsa-miR-1275, hsa-miR-148a-3p, hsa-miR-20b-5p, hsa-miR-335-5p, hsa-miR-7-5p, hsa-miR-9-3p, hsa-miR-361-5p, hsa-miR-589-5p, hsa-miR-769-5p, hsa-miR-335-3p, hsa-miR-500a-3p, hsa-miR-2110, hsa-miR-320c, hsa-miR-1-3p, hsa-miR-320a-3p
PSD3	15	hsa-miR-20b-5p, hsa-miR-148a-3p, hsa-miR-335-5p, hsa-miR-7-5p, hsa-miR-361-5p, hsa-miR-589-5p, hsa-miR-629-5p, hsa-miR-500a-3p, hsa-miR-2110, hsa-miR-320c, hsa-miR-1468-5p, hsa-miR-1-3p, hsa-miR-133a-3p, hsa-miR-320a-3p, hsa-miR-147b-3p
PSMD2	15	hsa-miR-361-5p, hsa-miR-148a-3p, hsa-miR-20b-5p, hsa-miR-335-5p, hsa-miR-138-5p, hsa-miR-339-5p, hsa-miR-589-5p, hsa-miR-769-5p, hsa-miR-361-3p, hsa-miR-335-3p, hsa-miR-500a-3p, hsa-miR-671-5p, hsa-miR-320c, hsa-miR-3615, hsa-miR-320a-3p
PTPN14	15	hsa-miR-335-5p, hsa-miR-629-5p, hsa-miR-2110, hsa-miR-4516, hsa-miR-148a-3p, hsa-miR-20b-5p, hsa-miR-7-5p, hsa-miR-9-3p, hsa-miR-138-5p, hsa-miR-574-5p, hsa-miR-769-5p, hsa-miR-335-3p, hsa-miR-1-3p, hsa-miR-887-3p, hsa-miR-320a-3p
PTPN4	15	hsa-miR-148a-3p, hsa-miR-20b-5p, hsa-miR-7-5p, hsa-miR-9-3p, hsa-miR-361-5p, hsa-miR-574-5p, hsa-miR-589-5p, hsa-miR-320d, hsa-miR-671-5p, hsa-miR-2110, hsa-miR-320c, hsa-miR-1-3p, hsa-miR-133a-3p, hsa-miR-320a-3p, hsa-miR-147b-3p
PTPRD	15	hsa-miR-4791, hsa-miR-1-3p, hsa-miR-148a-3p, hsa-miR-20b-5p, hsa-miR-7-5p, hsa-miR-138-5p, hsa-miR-574-5p, hsa-miR-188-5p, hsa-miR-

		629-5p, hsa-miR-500a-3p, hsa-miR-671-5p, hsa-miR-2110, hsa-miR-320c, hsa-miR-378i, hsa-miR-320a-3p
RAB2A	15	hsa-miR-20b-5p, hsa-miR-335-5p, hsa-miR-7-5p, hsa-miR-9-3p, hsa-miR-138-5p, hsa-miR-769-5p, hsa-miR-188-5p, hsa-miR-29c-5p, hsa-miR-342-5p, hsa-miR-335-3p, hsa-miR-2110, hsa-miR-320c, hsa-miR-1-3p, hsa-miR-320a-3p, hsa-miR-147b-3p
RAB3GAP1	15	hsa-miR-769-5p, hsa-miR-148a-3p, hsa-miR-138-5p, hsa-miR-589-5p, hsa-miR-188-5p, hsa-miR-29c-5p, hsa-miR-335-3p, hsa-miR-500a-3p, hsa-miR-671-5p, hsa-miR-2110, hsa-miR-320c, hsa-miR-378i, hsa-miR-1-3p, hsa-miR-320a-3p, hsa-miR-147b-3p
RAD54L2	15	hsa-miR-7-5p, hsa-miR-20b-5p, hsa-miR-335-5p, hsa-miR-138-5p, hsa-miR-1275, hsa-miR-29c-5p, hsa-miR-335-3p, hsa-miR-425-3p, hsa-miR-500a-3p, hsa-miR-671-5p, hsa-miR-2110, hsa-miR-3615, hsa-miR-133a-3p, hsa-miR-320a-3p, hsa-miR-147b-3p
RANBP2	15	hsa-miR-148a-3p, hsa-miR-20b-5p, hsa-miR-335-5p, hsa-miR-7-5p, hsa-miR-361-5p, hsa-miR-589-5p, hsa-miR-1275, hsa-miR-629-5p, hsa-miR-335-3p, hsa-miR-500a-3p, hsa-miR-2110, hsa-miR-320c, hsa-miR-874-3p, hsa-miR-1-3p, hsa-miR-320a-3p
RBBP4	15	hsa-miR-148a-3p, hsa-miR-20b-5p, hsa-miR-7-5p, hsa-miR-138-5p, hsa-miR-339-5p, hsa-miR-335-3p, hsa-miR-500a-3p, hsa-miR-671-5p, hsa-miR-675-3p, hsa-miR-2110, hsa-miR-378i, hsa-miR-874-3p, hsa-miR-1-3p, hsa-miR-133a-3p, hsa-miR-320a-3p
RBM25	15	hsa-miR-148a-3p, hsa-miR-20b-5p, hsa-miR-7-5p, hsa-miR-138-5p, hsa-miR-361-5p, hsa-miR-339-5p, hsa-miR-589-5p, hsa-miR-361-3p, hsa-miR-342-5p, hsa-miR-335-3p, hsa-miR-425-3p, hsa-miR-500a-3p, hsa-miR-3615, hsa-miR-1468-5p, hsa-miR-320a-3p
RBM47	15	hsa-miR-1-3p, hsa-miR-335-5p, hsa-miR-148a-3p, hsa-miR-7-5p, hsa-miR-9-3p, hsa-miR-320d, hsa-miR-342-5p, hsa-miR-335-3p, hsa-miR-425-3p, hsa-miR-2110, hsa-miR-320c, hsa-miR-378i, hsa-miR-320a-3p, hsa-miR-147b-3p, hsa-miR-190b-5p
RCC2	15	hsa-miR-148a-3p, hsa-miR-7-5p, hsa-miR-335-3p, hsa-miR-378i, hsa-miR-1-3p, hsa-miR-20b-5p, hsa-miR-335-5p, hsa-miR-9-3p, hsa-miR-361-5p, hsa-miR-500a-3p, hsa-miR-671-5p, hsa-miR-2110, hsa-miR-320c, hsa-miR-133a-3p, hsa-miR-320a-3p

REEP3	15	hsa-miR-20b-5p, hsa-miR-335-5p, hsa-miR-148a-3p, hsa-miR-7-5p, hsa-miR-9-3p, hsa-miR-361-5p, hsa-miR-574-5p, hsa-miR-335-3p, hsa-miR-425-3p, hsa-miR-671-5p, hsa-miR-675-3p, hsa-miR-320c, hsa-miR-1-3p, hsa-miR-320a-3p, hsa-miR-147b-3p
RERE	15	hsa-miR-148a-3p, hsa-miR-20b-5p, hsa-miR-335-5p, hsa-miR-7-5p, hsa-miR-138-5p, hsa-miR-361-5p, hsa-miR-574-5p, hsa-miR-1275, hsa-miR-500a-3p, hsa-miR-1307-3p, hsa-miR-3615, hsa-miR-1-3p, hsa-miR-133a-3p, hsa-miR-320a-3p, hsa-miR-147b-3p
RHOB	15	hsa-miR-335-5p, hsa-miR-342-5p, hsa-miR-148a-3p, hsa-miR-20b-5p, hsa-miR-7-5p, hsa-miR-138-5p, hsa-miR-361-5p, hsa-miR-574-5p, hsa-miR-361-3p, hsa-miR-500a-3p, hsa-miR-671-5p, hsa-miR-2110, hsa-miR-320c, hsa-miR-320a-3p, hsa-miR-147b-3p
RLIM	15	hsa-miR-20b-5p, hsa-miR-148a-3p, hsa-miR-7-5p, hsa-miR-361-5p, hsa-miR-574-5p, hsa-miR-769-5p, hsa-miR-629-5p, hsa-miR-342-5p, hsa-miR-335-3p, hsa-miR-425-3p, hsa-miR-671-5p, hsa-miR-1307-3p, hsa-miR-1-3p, hsa-miR-133a-3p, hsa-miR-320a-3p
RNF4	15	hsa-miR-361-5p, hsa-miR-20b-5p, hsa-miR-335-5p, hsa-miR-7-5p, hsa-miR-138-5p, hsa-miR-769-5p, hsa-miR-342-5p, hsa-miR-335-3p, hsa-miR-671-5p, hsa-miR-3168, hsa-miR-2110, hsa-miR-320c, hsa-miR-874-3p, hsa-miR-1-3p, hsa-miR-320a-3p
RNF40	15	hsa-miR-769-5p, hsa-miR-7704, hsa-miR-20b-5p, hsa-miR-335-5p, hsa-miR-7-5p, hsa-miR-138-5p, hsa-miR-339-5p, hsa-miR-574-5p, hsa-miR-335-3p, hsa-miR-500a-3p, hsa-miR-671-5p, hsa-miR-1307-3p, hsa-miR-1307-5p, hsa-miR-1-3p, hsa-miR-133a-3p
RSF1	15	hsa-miR-1-3p, hsa-miR-20b-5p, hsa-miR-335-5p, hsa-miR-7-5p, hsa-miR-9-3p, hsa-miR-138-5p, hsa-miR-339-5p, hsa-miR-574-5p, hsa-miR-589-5p, hsa-miR-320d, hsa-miR-335-3p, hsa-miR-425-3p, hsa-miR-500a-3p, hsa-miR-133a-3p, hsa-miR-320a-3p
RTN4	15	hsa-miR-148a-3p, hsa-miR-20b-5p, hsa-miR-335-5p, hsa-miR-9-3p, hsa-miR-361-5p, hsa-miR-339-5p, hsa-miR-574-5p, hsa-miR-769-5p, hsa-miR-320d, hsa-miR-335-3p, hsa-miR-500a-3p, hsa-miR-320c, hsa-miR-3615, hsa-miR-320a-3p, hsa-miR-147b-3p
SBF2	15	hsa-miR-335-5p, hsa-miR-148a-3p, hsa-miR-20b-5p, hsa-miR-7-5p, hsa-miR-138-5p, hsa-miR-574-5p, hsa-miR-320d, hsa-miR-335-3p, hsa-miR-

		500a-3p, hsa-miR-2110, hsa-miR-320c, hsa-miR-378i, hsa-miR-1-3p, hsa-miR-887-3p, hsa-miR-320a-3p
SCAF4	15	hsa-miR-148a-3p, hsa-miR-20b-5p, hsa-miR-7-5p, hsa-miR-138-5p, hsa-miR-361-5p, hsa-miR-574-5p, hsa-miR-589-5p, hsa-miR-769-5p, hsa-miR-188-5p, hsa-miR-629-5p, hsa-miR-1307-3p, hsa-miR-1-3p, hsa-miR-133a-3p, hsa-miR-320a-3p, hsa-miR-147b-3p
SDC1	15	hsa-miR-148a-3p, hsa-miR-20b-5p, hsa-miR-335-5p, hsa-miR-7-5p, hsa-miR-138-5p, hsa-miR-361-5p, hsa-miR-769-5p, hsa-miR-335-3p, hsa-miR-500a-3p, hsa-miR-671-5p, hsa-miR-1307-3p, hsa-miR-2110, hsa-miR-1-3p, hsa-miR-320a-3p, hsa-miR-147b-3p
SELENON	15	hsa-miR-342-5p, hsa-miR-148a-3p, hsa-miR-20b-5p, hsa-miR-7-5p, hsa-miR-138-5p, hsa-miR-574-5p, hsa-miR-589-5p, hsa-miR-769-5p, hsa-miR-320d, hsa-miR-335-3p, hsa-miR-671-5p, hsa-miR-1307-3p, hsa-miR-320c, hsa-miR-1-3p, hsa-miR-320a-3p
SENP2	15	hsa-miR-1275, hsa-miR-4516, hsa-miR-148a-3p, hsa-miR-20b-5p, hsa-miR-7-5p, hsa-miR-138-5p, hsa-miR-361-5p, hsa-miR-589-5p, hsa-miR-629-5p, hsa-miR-29c-5p, hsa-miR-361-3p, hsa-miR-500a-3p, hsa-miR-2110, hsa-miR-1-3p, hsa-miR-320a-3p
SFPQ	15	hsa-miR-148a-3p, hsa-miR-20b-5p, hsa-miR-7-5p, hsa-miR-138-5p, hsa-miR-361-5p, hsa-miR-339-5p, hsa-miR-574-5p, hsa-miR-320d, hsa-miR-1275, hsa-miR-335-3p, hsa-miR-425-3p, hsa-miR-671-5p, hsa-miR-320c, hsa-miR-3615, hsa-miR-147b-3p
SH3GL1	15	hsa-miR-2110, hsa-miR-148a-3p, hsa-miR-20b-5p, hsa-miR-7-5p, hsa-miR-138-5p, hsa-miR-574-5p, hsa-miR-769-5p, hsa-miR-1275, hsa-miR-335-3p, hsa-miR-671-5p, hsa-miR-320c, hsa-miR-3615, hsa-miR-1-3p, hsa-miR-133a-3p, hsa-miR-320a-3p
SHC1	15	hsa-miR-20b-5p, hsa-miR-335-5p, hsa-miR-138-5p, hsa-miR-361-5p, hsa-miR-769-5p, hsa-miR-1275, hsa-miR-361-3p, hsa-miR-335-3p, hsa-miR-671-5p, hsa-miR-320c, hsa-miR-378i, hsa-miR-874-3p, hsa-miR-1-3p, hsa-miR-320a-3p, hsa-miR-147b-3p
SLC25A3	15	hsa-miR-148a-3p, hsa-miR-20b-5p, hsa-miR-335-5p, hsa-miR-7-5p, hsa-miR-9-3p, hsa-miR-589-5p, hsa-miR-769-5p, hsa-miR-320d, hsa-miR-629-5p, hsa-miR-335-3p, hsa-miR-500a-3p, hsa-miR-671-5p, hsa-miR-320c, hsa-miR-320a-3p, hsa-miR-147b-3p

SLC25A36	15	hsa-miR-9-3p, hsa-miR-4516, hsa-miR-148a-3p, hsa-miR-20b-5p, hsa-miR-335-5p, hsa-miR-7-5p, hsa-miR-574-5p, hsa-miR-769-5p, hsa-miR-320d, hsa-miR-3168, hsa-miR-2110, hsa-miR-320c, hsa-miR-1-3p, hsa-miR-887-3p, hsa-miR-320a-3p
SLC25A5	15	hsa-miR-361-5p, hsa-miR-148a-3p, hsa-miR-20b-5p, hsa-miR-335-5p, hsa-miR-7-5p, hsa-miR-9-3p, hsa-miR-138-5p, hsa-miR-574-5p, hsa-miR-320d, hsa-miR-425-3p, hsa-miR-2110, hsa-miR-320c, hsa-miR-1-3p, hsa-miR-133a-3p, hsa-miR-320a-3p
SLC2A3	15	hsa-miR-148a-3p, hsa-miR-335-5p, hsa-miR-20b-5p, hsa-miR-9-3p, hsa-miR-138-5p, hsa-miR-361-5p, hsa-miR-574-5p, hsa-miR-589-5p, hsa-miR-320d, hsa-miR-551b-3p, hsa-miR-671-5p, hsa-miR-320c, hsa-miR-1-3p, hsa-miR-133a-3p, hsa-miR-320a-3p
SLC30A7	15	hsa-miR-20b-5p, hsa-miR-148a-3p, hsa-miR-7-5p, hsa-miR-9-3p, hsa-miR-361-5p, hsa-miR-574-5p, hsa-miR-188-5p, hsa-miR-320d, hsa-miR-29c-5p, hsa-miR-500a-3p, hsa-miR-671-5p, hsa-miR-3168, hsa-miR-2110, hsa-miR-320a-3p, hsa-miR-147b-3p
SLC44A2	15	hsa-miR-148a-3p, hsa-miR-20b-5p, hsa-miR-335-5p, hsa-miR-7-5p, hsa-miR-138-5p, hsa-miR-361-5p, hsa-miR-589-5p, hsa-miR-342-5p, hsa-miR-1307-5p, hsa-miR-320c, hsa-miR-378i, hsa-miR-1-3p, hsa-miR-887-3p, hsa-miR-320a-3p, hsa-miR-190b-5p
SMARCA5	15	hsa-miR-500a-3p, hsa-miR-148a-3p, hsa-miR-20b-5p, hsa-miR-335-5p, hsa-miR-7-5p, hsa-miR-361-5p, hsa-miR-629-5p, hsa-miR-335-3p, hsa-miR-3168, hsa-miR-3615, hsa-miR-378i, hsa-miR-1-3p, hsa-miR-133a-3p, hsa-miR-320a-3p, hsa-miR-147b-3p
SMARCC1	15	hsa-miR-320c, hsa-miR-1-3p, hsa-miR-20b-5p, hsa-miR-7-5p, hsa-miR-9-3p, hsa-miR-138-5p, hsa-miR-361-5p, hsa-miR-320d, hsa-miR-629-5p, hsa-miR-335-3p, hsa-miR-425-3p, hsa-miR-671-5p, hsa-miR-2110, hsa-miR-133a-3p, hsa-miR-320a-3p
SNX27	15	hsa-miR-148a-3p, hsa-miR-20b-5p, hsa-miR-7-5p, hsa-miR-138-5p, hsa-miR-574-5p, hsa-miR-188-5p, hsa-miR-320d, hsa-miR-1275, hsa-miR-29c-5p, hsa-miR-671-5p, hsa-miR-1307-3p, hsa-miR-2110, hsa-miR-320c, hsa-miR-1-3p, hsa-miR-320a-3p
SOCS7	15	hsa-miR-20b-5p, hsa-miR-148a-3p, hsa-miR-335-5p, hsa-miR-7-5p, hsa-miR-138-5p, hsa-miR-574-5p, hsa-miR-769-5p, hsa-miR-320d, hsa-miR-

		361-3p, hsa-miR-500a-3p, hsa-miR-2110, hsa-miR-320c, hsa-miR-3615, hsa-miR-320a-3p, hsa-miR-147b-3p
SORT1	15	hsa-miR-148a-3p, hsa-miR-20b-5p, hsa-miR-7-5p, hsa-miR-138-5p, hsa-miR-361-5p, hsa-miR-339-5p, hsa-miR-361-3p, hsa-miR-335-3p, hsa-miR-425-3p, hsa-miR-500a-3p, hsa-miR-671-5p, hsa-miR-320c, hsa-miR-1-3p, hsa-miR-320a-3p, hsa-miR-147b-3p
SOS1	15	hsa-miR-148a-3p, hsa-miR-20b-5p, hsa-miR-7-5p, hsa-miR-361-5p, hsa-miR-574-5p, hsa-miR-769-5p, hsa-miR-320d, hsa-miR-361-3p, hsa-miR-335-3p, hsa-miR-425-3p, hsa-miR-500a-3p, hsa-miR-2110, hsa-miR-320c, hsa-miR-1-3p, hsa-miR-320a-3p
SPRY4	15	hsa-miR-335-5p, hsa-miR-574-5p, hsa-miR-342-5p, hsa-miR-4516, hsa-miR-148a-3p, hsa-miR-20b-5p, hsa-miR-7-5p, hsa-miR-138-5p, hsa-miR-361-5p, hsa-miR-589-5p, hsa-miR-320d, hsa-miR-500a-3p, hsa-miR-671-5p, hsa-miR-320c, hsa-miR-320a-3p
SRSF4	15	hsa-miR-769-5p, hsa-miR-1-3p, hsa-miR-20b-5p, hsa-miR-335-5p, hsa-miR-7-5p, hsa-miR-361-5p, hsa-miR-1275, hsa-miR-335-3p, hsa-miR-671-5p, hsa-miR-1307-5p, hsa-miR-320c, hsa-miR-3615, hsa-miR-378i, hsa-miR-320a-3p, hsa-miR-147b-3p
SRSF7	15	hsa-miR-188-5p, hsa-miR-320d, hsa-miR-320c, hsa-miR-1-3p, hsa-miR-148a-3p, hsa-miR-20b-5p, hsa-miR-7-5p, hsa-miR-138-5p, hsa-miR-361-5p, hsa-miR-342-5p, hsa-miR-425-3p, hsa-miR-500a-3p, hsa-miR-1307-3p, hsa-miR-2110, hsa-miR-320a-3p
SS18	15	hsa-miR-148a-3p, hsa-miR-20b-5p, hsa-miR-335-5p, hsa-miR-7-5p, hsa-miR-574-5p, hsa-miR-188-5p, hsa-miR-320d, hsa-miR-1275, hsa-miR-2110, hsa-miR-320c, hsa-miR-874-3p, hsa-miR-1-3p, hsa-miR-133a-3p, hsa-miR-320a-3p, hsa-miR-147b-3p
STK24	15	hsa-miR-1-3p, hsa-miR-20b-5p, hsa-miR-335-5p, hsa-miR-7-5p, hsa-miR-9-3p, hsa-miR-361-5p, hsa-miR-339-5p, hsa-miR-574-5p, hsa-miR-1275, hsa-miR-335-3p, hsa-miR-500a-3p, hsa-miR-671-5p, hsa-miR-2110, hsa-miR-320c, hsa-miR-320a-3p
STRN4	15	hsa-miR-148a-3p, hsa-miR-20b-5p, hsa-miR-7-5p, hsa-miR-138-5p, hsa-miR-361-5p, hsa-miR-1275, hsa-miR-361-3p, hsa-miR-335-3p, hsa-miR-500a-3p, hsa-miR-671-5p, hsa-miR-320c, hsa-miR-1-3p, hsa-miR-133a-3p, hsa-miR-320a-3p, hsa-miR-147b-3p

STT3B	15	hsa-miR-148a-3p, hsa-miR-20b-5p, hsa-miR-335-5p, hsa-miR-7-5p, hsa-miR-138-5p, hsa-miR-361-5p, hsa-miR-339-5p, hsa-miR-589-5p, hsa-miR-188-5p, hsa-miR-29c-5p, hsa-miR-500a-3p, hsa-miR-1307-3p, hsa-miR-2110, hsa-miR-1-3p, hsa-miR-320a-3p
SUPT5H	15	hsa-miR-148a-3p, hsa-miR-20b-5p, hsa-miR-7-5p, hsa-miR-138-5p, hsa-miR-339-5p, hsa-miR-589-5p, hsa-miR-361-3p, hsa-miR-335-3p, hsa-miR-425-3p, hsa-miR-500a-3p, hsa-miR-671-5p, hsa-miR-3615, hsa-miR-378i, hsa-miR-1-3p, hsa-miR-320a-3p
SUPT6H	15	hsa-miR-148a-3p, hsa-miR-20b-5p, hsa-miR-335-5p, hsa-miR-7-5p, hsa-miR-138-5p, hsa-miR-339-5p, hsa-miR-574-5p, hsa-miR-589-5p, hsa-miR-335-3p, hsa-miR-500a-3p, hsa-miR-671-5p, hsa-miR-1307-3p, hsa-miR-1307-5p, hsa-miR-3615, hsa-miR-320a-3p
SYNRG	15	hsa-miR-1307-3p, hsa-miR-148a-3p, hsa-miR-20b-5p, hsa-miR-7-5p, hsa-miR-9-3p, hsa-miR-138-5p, hsa-miR-361-5p, hsa-miR-339-5p, hsa-miR-574-5p, hsa-miR-335-3p, hsa-miR-671-5p, hsa-miR-2110, hsa-miR-320c, hsa-miR-320a-3p, hsa-miR-147b-3p
SZRD1	15	hsa-miR-7-5p, hsa-miR-1275, hsa-miR-148a-3p, hsa-miR-20b-5p, hsa-miR-361-5p, hsa-miR-574-5p, hsa-miR-629-5p, hsa-miR-335-3p, hsa-miR-425-3p, hsa-miR-500a-3p, hsa-miR-671-5p, hsa-miR-2110, hsa-miR-3615, hsa-miR-378i, hsa-miR-1-3p
TAB3	15	hsa-miR-188-5p, hsa-miR-20b-5p, hsa-miR-7-5p, hsa-miR-9-3p, hsa-miR-361-5p, hsa-miR-574-5p, hsa-miR-589-5p, hsa-miR-320d, hsa-miR-1275, hsa-miR-342-5p, hsa-miR-335-3p, hsa-miR-320c, hsa-miR-1-3p, hsa-miR-887-3p, hsa-miR-320a-3p
TAF4	15	hsa-miR-7-5p, hsa-miR-148a-3p, hsa-miR-20b-5p, hsa-miR-574-5p, hsa-miR-589-5p, hsa-miR-769-5p, hsa-miR-342-5p, hsa-miR-500a-3p, hsa-miR-671-5p, hsa-miR-1307-5p, hsa-miR-320c, hsa-miR-887-3p, hsa-miR-12136, hsa-miR-320a-3p, hsa-miR-147b-3p
TAF5L	15	hsa-miR-148a-3p, hsa-miR-20b-5p, hsa-miR-7-5p, hsa-miR-138-5p, hsa-miR-361-5p, hsa-miR-339-5p, hsa-miR-589-5p, hsa-miR-769-5p, hsa-miR-335-3p, hsa-miR-500a-3p, hsa-miR-671-5p, hsa-miR-378i, hsa-miR-1-3p, hsa-miR-133a-3p, hsa-miR-147b-3p
TENT5C	15	hsa-miR-20b-5p, hsa-miR-335-5p, hsa-miR-7-5p, hsa-miR-9-3p, hsa-miR-361-5p, hsa-miR-574-5p, hsa-miR-320d, hsa-miR-629-5p, hsa-miR-

		29c-5p, hsa-miR-361-3p, hsa-miR-335-3p, hsa-miR-671-5p, hsa-miR-2110, hsa-miR-320c, hsa-miR-378i
TEX2	15	hsa-miR-148a-3p, hsa-miR-20b-5p, hsa-miR-7-5p, hsa-miR-138-5p, hsa-miR-361-5p, hsa-miR-574-5p, hsa-miR-320d, hsa-miR-361-3p, hsa-miR-425-3p, hsa-miR-320c, hsa-miR-378i, hsa-miR-1-3p, hsa-miR-133a-3p, hsa-miR-320a-3p, hsa-miR-147b-3p
TIPARP	15	hsa-miR-148a-3p, hsa-miR-20b-5p, hsa-miR-7-5p, hsa-miR-9-3p, hsa-miR-138-5p, hsa-miR-574-5p, hsa-miR-320d, hsa-miR-335-3p, hsa-miR-671-5p, hsa-miR-320c, hsa-miR-378i, hsa-miR-874-3p, hsa-miR-1-3p, hsa-miR-320a-3p, hsa-miR-147b-3p
TKT	15	hsa-miR-1-3p, hsa-miR-148a-3p, hsa-miR-20b-5p, hsa-miR-7-5p, hsa-miR-138-5p, hsa-miR-574-5p, hsa-miR-361-3p, hsa-miR-425-3p, hsa-miR-1307-3p, hsa-miR-1307-5p, hsa-miR-2110, hsa-miR-3615, hsa-miR-378i, hsa-miR-874-3p, hsa-miR-147b-3p
TM9SF3	15	hsa-miR-9-3p, hsa-miR-148a-3p, hsa-miR-20b-5p, hsa-miR-7-5p, hsa-miR-361-5p, hsa-miR-574-5p, hsa-miR-629-5p, hsa-miR-29c-5p, hsa-miR-335-3p, hsa-miR-500a-3p, hsa-miR-1307-3p, hsa-miR-2110, hsa-miR-1-3p, hsa-miR-133a-3p, hsa-miR-320a-3p
TMEM33	15	hsa-miR-335-3p, hsa-miR-4497, hsa-miR-148a-3p, hsa-miR-20b-5p, hsa-miR-335-5p, hsa-miR-7-5p, hsa-miR-589-5p, hsa-miR-320d, hsa-miR-629-5p, hsa-miR-425-3p, hsa-miR-671-5p, hsa-miR-320c, hsa-miR-1-3p, hsa-miR-320a-3p, hsa-miR-190b-5p
TMEM64	15	hsa-miR-20b-5p, hsa-miR-148a-3p, hsa-miR-335-5p, hsa-miR-9-3p, hsa-miR-138-5p, hsa-miR-361-5p, hsa-miR-339-5p, hsa-miR-320d, hsa-miR-629-5p, hsa-miR-342-5p, hsa-miR-335-3p, hsa-miR-671-5p, hsa-miR-320c, hsa-miR-1-3p, hsa-miR-320a-3p
TNKS2	15	hsa-miR-20b-5p, hsa-miR-335-5p, hsa-miR-1-3p, hsa-miR-148a-3p, hsa-miR-7-5p, hsa-miR-9-3p, hsa-miR-138-5p, hsa-miR-574-5p, hsa-miR-769-5p, hsa-miR-342-5p, hsa-miR-335-3p, hsa-miR-671-5p, hsa-miR-2110, hsa-miR-320c, hsa-miR-320a-3p
TNRC18	15	hsa-miR-1307-3p, hsa-miR-148a-3p, hsa-miR-20b-5p, hsa-miR-7-5p, hsa-miR-138-5p, hsa-miR-361-5p, hsa-miR-339-5p, hsa-miR-769-5p, hsa-miR-629-5p, hsa-miR-335-3p, hsa-miR-500a-3p, hsa-miR-671-5p, hsa-miR-320c, hsa-miR-3615, hsa-miR-320a-3p

TOP1	15	hsa-miR-148a-3p, hsa-miR-335-5p, hsa-miR-339-5p, hsa-miR-574-5p, hsa-miR-361-3p, hsa-miR-342-5p, hsa-miR-335-3p, hsa-miR-500a-3p, hsa-miR-1307-3p, hsa-miR-2110, hsa-miR-320c, hsa-miR-3615, hsa-miR-378i, hsa-miR-887-3p, hsa-miR-320a-3p
TOP2A	15	hsa-miR-7-5p, hsa-miR-148a-3p, hsa-miR-20b-5p, hsa-miR-335-5p, hsa-miR-9-3p, hsa-miR-138-5p, hsa-miR-361-5p, hsa-miR-629-5p, hsa-miR-335-3p, hsa-miR-320c, hsa-miR-378i, hsa-miR-1-3p, hsa-miR-133a-3p, hsa-miR-320a-3p, hsa-miR-147b-3p
TRAM2	15	hsa-miR-7-5p, hsa-miR-148a-3p, hsa-miR-20b-5p, hsa-miR-335-5p, hsa-miR-138-5p, hsa-miR-361-5p, hsa-miR-1275, hsa-miR-629-5p, hsa-miR-500a-3p, hsa-miR-671-5p, hsa-miR-2110, hsa-miR-320c, hsa-miR-1-3p, hsa-miR-320a-3p, hsa-miR-147b-3p
TSR1	15	hsa-miR-20b-5p, hsa-miR-361-5p, hsa-miR-335-3p, hsa-miR-7-5p, hsa-miR-138-5p, hsa-miR-339-5p, hsa-miR-574-5p, hsa-miR-320d, hsa-miR-500a-3p, hsa-miR-671-5p, hsa-miR-2110, hsa-miR-320c, hsa-miR-1-3p, hsa-miR-320a-3p, hsa-miR-147b-3p
TXLNA	15	hsa-miR-20b-5p, hsa-miR-148a-3p, hsa-miR-7-5p, hsa-miR-138-5p, hsa-miR-589-5p, hsa-miR-320d, hsa-miR-335-3p, hsa-miR-500a-3p, hsa-miR-671-5p, hsa-miR-2110, hsa-miR-320c, hsa-miR-1-3p, hsa-miR-133a-3p, hsa-miR-320a-3p, hsa-miR-147b-3p
TXNRD1	15	hsa-miR-148a-3p, hsa-miR-20b-5p, hsa-miR-335-5p, hsa-miR-7-5p, hsa-miR-339-5p, hsa-miR-589-5p, hsa-miR-769-5p, hsa-miR-361-3p, hsa-miR-342-5p, hsa-miR-335-3p, hsa-miR-2110, hsa-miR-320c, hsa-miR-1-3p, hsa-miR-320a-3p, hsa-miR-147b-3p
U2SURP	15	hsa-miR-20b-5p, hsa-miR-361-5p, hsa-miR-148a-3p, hsa-miR-335-5p, hsa-miR-7-5p, hsa-miR-9-3p, hsa-miR-138-5p, hsa-miR-574-5p, hsa-miR-589-5p, hsa-miR-769-5p, hsa-miR-342-5p, hsa-miR-500a-3p, hsa-miR-2110, hsa-miR-1-3p, hsa-miR-320a-3p
UBE2I	15	hsa-miR-188-5p, hsa-miR-361-3p, hsa-miR-1-3p, hsa-miR-148a-3p, hsa-miR-20b-5p, hsa-miR-7-5p, hsa-miR-361-5p, hsa-miR-339-5p, hsa-miR-574-5p, hsa-miR-769-5p, hsa-miR-500a-3p, hsa-miR-3168, hsa-miR-320c, hsa-miR-133a-3p, hsa-miR-320a-3p
UBQLN1	15	hsa-miR-3168, hsa-miR-148a-3p, hsa-miR-20b-5p, hsa-miR-335-5p, hsa-miR-7-5p, hsa-miR-361-5p, hsa-miR-339-5p, hsa-miR-589-5p, hsa-

		miR-629-5p, hsa-miR-320c, hsa-miR-378i, hsa-miR-1-3p, hsa-miR-133a-3p, hsa-miR-887-3p, hsa-miR-320a-3p
UNC13A	15	hsa-miR-148a-3p, hsa-miR-20b-5p, hsa-miR-7-5p, hsa-miR-138-5p, hsa-miR-629-5p, hsa-miR-29c-5p, hsa-miR-342-5p, hsa-miR-335-3p, hsa-miR-500a-3p, hsa-miR-1307-3p, hsa-miR-320c, hsa-miR-1-3p, hsa-miR-133a-3p, hsa-miR-887-3p, hsa-miR-320a-3p
UPF2	15	hsa-miR-148a-3p, hsa-miR-20b-5p, hsa-miR-335-5p, hsa-miR-7-5p, hsa-miR-9-3p, hsa-miR-138-5p, hsa-miR-769-5p, hsa-miR-361-3p, hsa-miR-335-3p, hsa-miR-500a-3p, hsa-miR-671-5p, hsa-miR-320c, hsa-miR-1-3p, hsa-miR-320a-3p, hsa-miR-190b-5p
USP11	15	hsa-miR-20b-5p, hsa-miR-335-5p, hsa-miR-7-5p, hsa-miR-138-5p, hsa-miR-769-5p, hsa-miR-342-5p, hsa-miR-335-3p, hsa-miR-500a-3p, hsa-miR-671-5p, hsa-miR-2110, hsa-miR-320c, hsa-miR-1-3p, hsa-miR-133a-3p, hsa-miR-887-3p, hsa-miR-320a-3p
USP36	15	hsa-miR-342-5p, hsa-miR-4516, hsa-miR-148a-3p, hsa-miR-20b-5p, hsa-miR-7-5p, hsa-miR-138-5p, hsa-miR-574-5p, hsa-miR-589-5p, hsa-miR-769-5p, hsa-miR-335-3p, hsa-miR-671-5p, hsa-miR-2110, hsa-miR-378i, hsa-miR-320a-3p, hsa-miR-147b-3p
USP7	15	hsa-miR-148a-3p, hsa-miR-20b-5p, hsa-miR-7-5p, hsa-miR-138-5p, hsa-miR-361-5p, hsa-miR-574-5p, hsa-miR-29c-5p, hsa-miR-335-3p, hsa-miR-425-3p, hsa-miR-1307-3p, hsa-miR-2110, hsa-miR-320c, hsa-miR-1-3p, hsa-miR-887-3p, hsa-miR-320a-3p
VPS39	15	hsa-miR-335-5p, hsa-miR-148a-3p, hsa-miR-7-5p, hsa-miR-138-5p, hsa-miR-361-5p, hsa-miR-339-5p, hsa-miR-188-5p, hsa-miR-629-5p, hsa-miR-361-3p, hsa-miR-342-5p, hsa-miR-500a-3p, hsa-miR-671-5p, hsa-miR-320c, hsa-miR-1-3p, hsa-miR-320a-3p
WDR47	15	hsa-miR-148a-3p, hsa-miR-20b-5p, hsa-miR-7-5p, hsa-miR-138-5p, hsa-miR-339-5p, hsa-miR-574-5p, hsa-miR-589-5p, hsa-miR-342-5p, hsa-miR-671-5p, hsa-miR-2110, hsa-miR-320c, hsa-miR-378i, hsa-miR-1-3p, hsa-miR-133a-3p, hsa-miR-320a-3p
WWTR1	15	hsa-miR-335-5p, hsa-miR-361-5p, hsa-miR-148a-3p, hsa-miR-20b-5p, hsa-miR-7-5p, hsa-miR-9-3p, hsa-miR-574-5p, hsa-miR-589-5p, hsa-miR-335-3p, hsa-miR-500a-3p, hsa-miR-2110, hsa-miR-320c, hsa-miR-3615, hsa-miR-1-3p, hsa-miR-320a-3p

XPO5	15	hsa-miR-425-3p, hsa-miR-148a-3p, hsa-miR-20b-5p, hsa-miR-335-5p, hsa-miR-7-5p, hsa-miR-138-5p, hsa-miR-361-5p, hsa-miR-589-5p, hsa-miR-29c-5p, hsa-miR-500a-3p, hsa-miR-671-5p, hsa-miR-320c, hsa-miR-378i, hsa-miR-320a-3p, hsa-miR-147b-3p
XPO6	15	hsa-miR-1-3p, hsa-miR-148a-3p, hsa-miR-20b-5p, hsa-miR-7-5p, hsa-miR-574-5p, hsa-miR-769-5p, hsa-miR-320d, hsa-miR-335-3p, hsa-miR-500a-3p, hsa-miR-671-5p, hsa-miR-2110, hsa-miR-320c, hsa-miR-133a-3p, hsa-miR-320a-3p, hsa-miR-147b-3p
YAP1	15	hsa-miR-138-5p, hsa-miR-335-3p, hsa-miR-20b-5p, hsa-miR-335-5p, hsa-miR-7-5p, hsa-miR-9-3p, hsa-miR-574-5p, hsa-miR-629-5p, hsa-miR-342-5p, hsa-miR-425-3p, hsa-miR-500a-3p, hsa-miR-671-5p, hsa-miR-320c, hsa-miR-1-3p, hsa-miR-320a-3p
YOD1	15	hsa-miR-20b-5p, hsa-miR-7-5p, hsa-miR-9-3p, hsa-miR-361-5p, hsa-miR-320d, hsa-miR-320c, hsa-miR-148a-3p, hsa-miR-335-5p, hsa-miR-138-5p, hsa-miR-574-5p, hsa-miR-589-5p, hsa-miR-335-3p, hsa-miR-500a-3p, hsa-miR-671-5p, hsa-miR-320a-3p
YWHAH	15	hsa-miR-148a-3p, hsa-miR-20b-5p, hsa-miR-7-5p, hsa-miR-9-3p, hsa-miR-138-5p, hsa-miR-361-5p, hsa-miR-320d, hsa-miR-629-5p, hsa-miR-361-3p, hsa-miR-335-3p, hsa-miR-1307-3p, hsa-miR-320c, hsa-miR-1-3p, hsa-miR-320a-3p, hsa-miR-147b-3p
YWHAQ	15	hsa-miR-1-3p, hsa-miR-148a-3p, hsa-miR-20b-5p, hsa-miR-335-5p, hsa-miR-7-5p, hsa-miR-361-5p, hsa-miR-361-3p, hsa-miR-342-5p, hsa-miR-335-3p, hsa-miR-500a-3p, hsa-miR-671-5p, hsa-miR-3615, hsa-miR-320a-3p, hsa-miR-147b-3p, hsa-miR-190b-5p
ZBED4	15	hsa-miR-148a-3p, hsa-miR-20b-5p, hsa-miR-335-5p, hsa-miR-7-5p, hsa-miR-9-3p, hsa-miR-138-5p, hsa-miR-339-5p, hsa-miR-769-5p, hsa-miR-342-5p, hsa-miR-500a-3p, hsa-miR-671-5p, hsa-miR-1307-3p, hsa-miR-2110, hsa-miR-320c, hsa-miR-320a-3p
ZDHHC20	15	hsa-miR-20b-5p, hsa-miR-335-5p, hsa-miR-574-5p, hsa-miR-148a-3p, hsa-miR-7-5p, hsa-miR-9-3p, hsa-miR-138-5p, hsa-miR-320d, hsa-miR-1275, hsa-miR-361-3p, hsa-miR-335-3p, hsa-miR-500a-3p, hsa-miR-3168, hsa-miR-874-3p, hsa-miR-1-3p
ZFAND5	15	hsa-miR-4516, hsa-miR-148a-3p, hsa-miR-138-5p, hsa-miR-361-5p, hsa-miR-339-5p, hsa-miR-574-5p, hsa-miR-589-5p, hsa-miR-320d, hsa-

		miR-29c-5p, hsa-miR-342-5p, hsa-miR-335-3p, hsa-miR-500a-3p, hsa-miR-1-3p, hsa-miR-320a-3p, hsa-miR-190b-5p
ZFR	15	hsa-miR-148a-3p, hsa-miR-20b-5p, hsa-miR-7-5p, hsa-miR-138-5p, hsa-miR-361-5p, hsa-miR-629-5p, hsa-miR-335-3p, hsa-miR-425-3p, hsa-miR-500a-3p, hsa-miR-671-5p, hsa-miR-2110, hsa-miR-378i, hsa-miR-1-3p, hsa-miR-320a-3p, hsa-miR-147b-3p
ZFYVE26	15	hsa-miR-148a-3p, hsa-miR-20b-5p, hsa-miR-7-5p, hsa-miR-138-5p, hsa-miR-339-5p, hsa-miR-589-5p, hsa-miR-769-5p, hsa-miR-342-5p, hsa-miR-335-3p, hsa-miR-425-3p, hsa-miR-500a-3p, hsa-miR-2110, hsa-miR-1-3p, hsa-miR-320a-3p, hsa-miR-147b-3p
ZNF367	15	hsa-miR-148a-3p, hsa-miR-20b-5p, hsa-miR-7-5p, hsa-miR-361-5p, hsa-miR-339-5p, hsa-miR-574-5p, hsa-miR-589-5p, hsa-miR-320d, hsa-miR-335-3p, hsa-miR-671-5p, hsa-miR-320c, hsa-miR-3615, hsa-miR-1-3p, hsa-miR-320a-3p, hsa-miR-147b-3p
ZNF431	15	hsa-miR-769-5p, hsa-miR-1307-3p, hsa-miR-20b-5p, hsa-miR-335-5p, hsa-miR-7-5p, hsa-miR-9-3p, hsa-miR-138-5p, hsa-miR-320d, hsa-miR-629-5p, hsa-miR-335-3p, hsa-miR-500a-3p, hsa-miR-671-5p, hsa-miR-2110, hsa-miR-320a-3p, hsa-miR-147b-3p
ZNF503	15	hsa-miR-335-5p, hsa-miR-148a-3p, hsa-miR-20b-5p, hsa-miR-7-5p, hsa-miR-9-3p, hsa-miR-339-5p, hsa-miR-574-5p, hsa-miR-769-5p, hsa-miR-320d, hsa-miR-671-5p, hsa-miR-1307-3p, hsa-miR-320c, hsa-miR-378i, hsa-miR-1-3p, hsa-miR-320a-3p
ZRANB2	15	hsa-miR-887-3p, hsa-miR-148a-3p, hsa-miR-20b-5p, hsa-miR-7-5p, hsa-miR-361-5p, hsa-miR-574-5p, hsa-miR-769-5p, hsa-miR-320d, hsa-miR-629-5p, hsa-miR-335-3p, hsa-miR-500a-3p, hsa-miR-2110, hsa-miR-320c, hsa-miR-1-3p, hsa-miR-320a-3p
